# Supplementary material for: Structural and Comparative Analyses of Insects Suggest the Presence of an Ultra-Conserved Regulatory Element of the Genes Encoding Vacuolar-Type ATPase Subunits and Assembly Factors
Source: Biology (Basel). 2023 Aug 13;12(8):1127. doi: 10.3390/biology12081127 (PMC10452791; doi:10.3390/biology12081127)
Supplement: Supplementary file 1 [file biology-12-01127-s001.zip › Figure S1.pdf]

Supplementary Figure S1. DNA Logo and DNA Positional Matrix describing the dCLEAR motif in Drosophilidae species. The sequences used to build the matrix are reported.

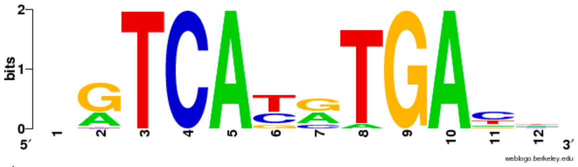

A	264	253	0	0	832	1	323	47	0	832	99	244
C	171	21	0	832	0	285	137	0	0	0	438	209
G	182	534	1	0	0	115	372	0	832	0	85	97
T	215	24	831	0	0	431	0	785	0	0	210	282

>Dmel\Atp6v0a2
TATCATGTGATC
>Dsim\Atp6v0a2
TATCATGTGATC
>Dsec\Atp6v0a2
TATCATGTGATC
>Dyak\Atp6v0a2
CATCATGTGATC
>Dere\Atp6v0a2
CATCATGTGATC
>Dbia\Atp6v0a2
TATCATGTGATC
>Dtak\Atp6v0a2
TATCATGTGATC
>Deug\Atp6v0a2
TATCATGTGATC
>Dele\Atp6v0a2
TATCATGTGATC
>Drho\Atp6v0a2
TATCATGTGATC
>Dfic\Atp6v0a2
TATCATGTGATC
>Dkik\Atp6v0a2
TATCATGTGATC
>Dbip\Atp6v0a2
TATCATGTGATC
>Dana\Atp6v0a2
TATCATGTGATA
>Dpse\Atp6v0a2
TATCATGTGATC
>Dper\Atp6v0a2
TATCATGTGATC
>Dwil\Atp6v0a2
CGTCATGTGATC
>Dmoj\Atp6v0a2
TATCATGTGACC
>Dvir\Atp6v0a2
TATCATGTGACC
>Dgri\Atp6v0a2
CATCATGTGATC

>Dmel\Atp6v0b
CGTCATGTGATT
>Dsim\Atp6v0b
CGTCATGTGATT
>Dsec\Atp6v0b

CGTCATGTGATT
>Dyak\Atp6v0b
CGTCATGTGATT
>Dere\Atp6v0b
CGTCATGTGATT
>Dbia\Atp6v0b
CGTCATGTGATT
>Dsuz\Atp6v0b
CGTCATGTGATT
>Dtak\Atp6v0b
CGTCATGTGATT
>Deug\Atp6v0b
CGTCATGTGATT
>Dele\Atp6v0b
CGTCATGTGATT
>Drho\Atp6v0b
CGTCATGTGATT
>Dfic\Atp6v0b
CGTCATGTGATT
>Dkik\Atp6v0b
CGTCATGTGATT
>Dana\Atp6v0b
CGTCATGTGATT
>Dbip\Atp6v0b
CGTCATGTGATT
>Dpse\Atp6v0b
CGTCATGTGATT
>Dper\Atp6v0b
CGTCATGTGATT
>Dmir\Atp6v0b
CGTCATGTGATT
>Dwil\Atp6v0b
AATCATGTGATT
>Dalb\Atp6v0b
CGTCATGTGATT
>Dmoj\Atp6v0b
CGTCATGTGATT
>Dvir\Atp6v0b
CGTCATGTGATT
>Dgri\Atp6v0b
CGTCATGTGATT

>Dmel\Atp6v0c1
AATCACGTGAGT
>Dmel\Atp6v0c2
GGTCATGTGAGG
>Dmel\Atp6v0c3
AGTCACAAGACC
>Dmel\Atp6v0c6
GATCACATGAGT
>Dmel\Atp6v0c7
AATCACATGACT
>Dmel\Atp6v0c8
CGTCACATGAAA
>Dmel\Atp6v0c9
GATCATGTGACT
>Dsim\Atp6v0c1
AATCACGTGAGT
>Dsim\Atp6v0c2
GGTCATGTGAGG
>Dsim\Atp6v0c3
AGTCACAAGACC
>Dsim\Atp6v0c6

GATCACATGAGT
>Dsim\Atp6v0c7
AATCACATGACT
>Dsim\Atp6v0c8
CGTCACATGAAA
>Dsim\Atp6v0c9
GATCATGTGACT
>Dsec\Atp6v0c1
AATCACGTGAGT
>Dsec\Atp6v0c2
GGTCATGTGAGG
>Dsec\Atp6v0c3
AGTCACAAGACC
>Dsec\Atp6v0c6
GATCACATGAGT
>Dsec\Atp6v0c7
AATCACATGACT
>Dsec\Atp6v0c8
CGTCACATGAAA
>Dsec\Atp6v0c9
GATCATGTGACT
>Dyak\Atp6v0c1
AATCACGTGAGT
>Dyak\Atp6v0c2
GGTCATGTGAGG
>Dyak\Atp6v0c3
AGTCACAAGACC
>Dyak\Atp6v0c5
GATCACATGAGT
>Dyak\Atp6v0c6
AATCACATGACT
>Dyak\Atp6v0c7
CGTCATATGAAA
>Dyak\Atp6v0c8
GATCATGTGACT
>Dere\Atp6v0c1
AATCACGTGAGT
>Dere\Atp6v0c2
GGTCATGTGAGG
>Dere\Atp6v0c3
AGTCACAAGACC
>Dere\Atp6v0c4
GATCACATGAGC
>Dere\Atp6v0c5
AATCACATGACT
>Dere\Atp6v0c6
CGTCATATGAAA
>Dere\Atp6v0c7
GATCATGTGACT
>Dbia\Atp6v0c1
AATCACGTGAGT
>Dbia\Atp6v0c2
GGTCATGTGAGC
>Dbia\Atp6v0c3
AGTCACAAGACC
>Dbia\Atp6v0c4
GATCACATGAGG
>Dbia\Atp6v0c5
CATCACATGACA
>Dbia\Atp6v0c6
GATCATGTGACT
>Dsuz\Atp6v0c1
AATCACGTGAGT

>Dsuz\Atp6v0c2
GGTCATGTGAGG
>Dsuz\Atp6v0c3
AGTCACAAGACT
>Dsuz\Atp6v0c4
GATCATGTGACT
>Dtak\Atp6v0c1
AATCACGTGAGT
>Dtak\Atp6v0c2
GGTCATGTGAGG
>Dtak\Atp6v0c3
AGTCACAAGACT
>Dtak\Atp6v0c4
GATCACATGAGG
>Dtak\Atp6v0c5
AATCACATGACA
>Dtak\Atp6v0c6
GATCATGTGACT
>Deug\Atp6v0c1
AATCACGTGAGT
>Deug\Atp6v0c2
GGTCATGTGAGG
>Deug\Atp6v0c3
AGTCACAAGACC
>Deug\Atp6v0c4
GATCACATGAGT
>Deug\Atp6v0c5
AATCACATGACA
>Deug\Atp6v0c6
GATCATGTGACT
>Dele\Atp6v0c1
AATCACGTGAGT
>Dele\Atp6v0c2
GGTCATGTGAGG
>Dele\Atp6v0c3
GGTCACAAGACC
>Dele\Atp6v0c4
GATCACATGAGT
>Dele\Atp6v0c5
AATCACATGATG
>Dele\Atp6v0c6
GATCATGTGACT
>Drho\Atp6v0c1
AATCACGTGAGT
>Drho\Atp6v0c2
GGTCATGTGAGG
>Drho\Atp6v0c3
AGTCACAAGACC
>Drho\Atp6v0c4
GATCACATGAGT
>Drho\Atp6v0c5
AATCACATGATG
>Drho\Atp6v0c6
GATCATGTGACT
>Dfic\Atp6v0c1
AATCACGTGAGT
>Dfic\Atp6v0c2
GGTCATGTGAGG
>Dfic\Atp6v0c3
AGTCACAAGACC
>Dfic\Atp6v0c4
GATCACAAGAGA
>Dfic\Atp6v0c5

```
GATCACATGATG
>Dfic\Atp6v0c6
GATCATGTGACT
>Dkik\Atp6v0c1
AATCACGTGAGT
>Dkik\Atp6v0c2
CGTCATGTGAGC
>Dkik\Atp6v0c3
AGTCACAAGACC
>Dkik\Atp6v0c4
GATCACATGAGC
>Dkik\Atp6v0c5
AGTCACATGACG
>Dkik\Atp6v0c6
GATCATGTGACT
>Dbip\Atp6v0c1
AATCACGTGAGT
>Dbip\Atp6v0c2
GGTCATGTGAGA
>Dbip\Atp6v0c3
AGTCACAAGACC
>Dbip\Atp6v0c4
GATCACATGAGT
>Dbip\Atp6v0c5
AGTCACATGACC
>Dbip\Atp6v0c6
GATCATGTGACT
>Dana\Atp6v0c1
AATCACGTGAGT
>Dana\Atp6v0c2
GGTCATGTGAGA
>Dana\Atp6v0c3
AGTCACAAGACC
>Dana\Atp6v0c4
GATCACATGAGT
>Dana\Atp6v0c5
AGTCACATGACT
>Dana\Atp6v0c6
GATCATGTGACT
>Dpse\Atp6v0c1
AATCACGTGAGT
>Dpse\Atp6v0c2
GGTCATATGAGG
>Dpse\Atp6v0c3
AGTCACAAGACC
>Dpse\Atp6v0c4
GATCACATGAGC
>Dpse\Atp6v0c5
AATCACATGACT
>Dpse\Atp6v0c6
GATCATGTGACT
>Dper\Atp6v0c1
AATCACGTGAGT
>Dper\Atp6v0c2
GGTCATATGAGG
>Dper\Atp6v0c5
AATCACATGACT
>Dper\Atp6v0c6
GATCATGTGACT
>Dmir\Atp6v0c1
AATCACGTGAGT
>Dmir\Atp6v0c2
GGTCATATGAGG
```

>Dmir\Atp6v0c3
AGTCACAAGACC
>Dmir\Atp6v0c4
GATCACATGAGC
>Dmir\Atp6v0c5
AATCACATGACT
>Dmir\Atp6v0c6
GATCATGTGACT
>Dwil\Atp6v0c1
AATCACGTGAGT
>Dwil\Atp6v0c2
AGTCACATGAGT
>Dwil\Atp6v0c3
AGTCACAAGACC
>Dwil\Atp6v0c4
GATCACATGAGT
>Dwil\Atp6v0c5
AATCACATGACC
>Dwil\Atp6v0c6
GATCATGTGACT
>Dalb\Atp6v0c1
AATCACGTGAGT
>Dalb\Atp6v0c2
AGTCATATGAGG
>Dalb\Atp6v0c3
AGTCACAAGACC
>Dalb\Atp6v0c5
AATCACATGACT
>Dalb\Atp6v0c6
AGTCATGTGACG
>Dmoj\Atp6v0c1
AATCACGTGAGT
>Dmoj\Atp6v0c2
AGTCATGTGAGG
>Dmoj\Atp6v0c3
AGTCACAAGACC
>Dmoj\Atp6v0c4
GATCACAAGAAG
>Dmoj\Atp6v0c5
AGTCACATGACA
>Dmoj\Atp6v0c6
GGTCATATGAAA
>Dmoj\Atp6v0c7
GGTCATGTGACC
>Dvir\Atp6v0c1
AATCACGTGAGT
>Dvir\Atp6v0c2
AGTCATATGAGG
>Dvir\Atp6v0c3
AGTCACAAGACC
>Dvir\Atp6v0c4
GATCAAATGAAC
>Dvir\Atp6v0c5
TGTCACATGACA
>Dvir\Atp6v0c6
CGTCATATGAAA
>Dvir\Atp6v0c7
GGTCATGTGACC
>Dgri\Atp6v0c1
AATCACGTGAGT
>Dgri\Atp6v0c2
AGTCATATGAGG
>Dgri\Atp6v0c3

AGTCACAAGACC
>Dgri\Atp6v0c4
GATCACATGATC
>Dgri\Atp6v0c5
AATCACATGACA
>Dgri\Atp6v0c6
CGTCATATGAAA
>Dgri\Atp6v0c7
GGTCATGTGATT

>Dmel\Atp6v0d1
AATCATATGACT
>Dmel\Atp6v0d2
GGTCACATGACC
>Dsim\Atp6v0d1
AATCATATGACT
>Dsim\Atp6v0d2
GGTCACATGACC
>Dsec\Atp6v0d1
AATCATATGACT
>Dsec\Atp6v0d2
GGTCACATGACC
>Dyak\Atp6v0d1
AATCATATGACT
>Dyak\Atp6v0d2
GGTCACATGACC
>Dere\Atp6v0d1
AATCATATGACT
>Dere\Atp6v0d2
GGTCACATGACC
>Dbia\Atp6v0d1
AATCATATGACT
>Dbia\Atp6v0d2
GGTCACATGACC
>Dsuz\Atp6v0d1
AATCATATGACT
>Dsuz\Atp6v0d2
GGTCACATGACC
>Dtak\Atp6v0d1
AATCATATGACT
>Dtak\Atp6v0d2
GGTCACATGACC
>Deug\Atp6v0d1
AATCATATGACT
>Deug\Atp6v0d2
GGTCACATGACC
>Dele\Atp6v0d1
AATCATATGACT
>Dele\Atp6v0d2
GGTCACATGACC
>Drho\Atp6v0d1
AATCATATGACT
>Drho\Atp6v0d2
GGTCACATGACC
>Dfic\Atp6v0d1
AATCATATGACT
>Dfic\Atp6v0d2
GGTCACATGACC
>Dkik\Atp6v0d1
AATCATATGACT

>Dkik\Atp6v0d2
GGTCACATGACC
>Dbip\Atp6v0d1
AATCATATGACT
>Dbip\Atp6v0d2
AATCACATGACC
>Dana\Atp6v0d1
AATCATATGACT
>Dana\Atp6v0d2
CCTCACATGACC
>Dpse\Atp6v0d1
AATCATATGACT
>Dpse\Atp6v0d2
TGTCATGTGACC
>Dper\Atp6v0d1
AATCATATGACT
>Dper\Atp6v0d2
TGTCATGTGACC
>Dmir\Atp6v0d1
AATCATATGACT
>Dmir\Atp6v0d2
TGTCATGTGACC
>Dwil\Atp6v0d1
AGTCATATGACT
>Dwil\Atp6v0d2
AGTCATGTGACG
>Dalb\Atp6v0d1
AATCATATGACT
>Dalb\Atp6v0d2
AGTCACATGACG
>Dmoj\Atp6v0d1
AATCATATGACT
>Dmoj\Atp6v0d2
AGTCACATGACG
>Dvir\Atp6v0d1
AATCATATGACT
>Dvir\Atp6v0d2
GGTCACATGACT
>Dgri\Atp6v0d1
AATCATATGACT
>Dgri\Atp6v0d2
AGTCACATGACA

>Dmel\Atp6v0e
CGTCATGTGATA
>Dsim\Atp6v0e
CGTCATGTGATA
>Dsec\Atp6v0e
CGTCATGTGATA
>Dyak\Atp6v0e
CGTCATGTGATA
>Dere\Atp6v0e
CGTCATGTGATA
>Dbia\Atp6v0e
TGTCATGTGATA
>Dsuz\Atp6v0e
CGTCATGTGATA
>Dtak\Atp6v0e
CGTCATGTGATA
>Deug\Atp6v0e
CGTCATGTGATA
>Dele\Atp6v0e
CGTCATGTGATA

>Drho\Atp6v0e
CGTCATGTGATA
>Dfic\Atp6v0e
CGTCATGTGATA
>Dkik\Atp6v0e
TGTCATGTGATA
>Dbip\Atp6v0e
TGTCATGTGATA
>Dana\Atp6v0e
TGTCATGTGATA
>Dpse\Atp6v0e
TGTCATGTGATA
>Dper\Atp6v0e
TGTCATGTGATA
>Dmir\Atp6v0e
TGTCATGTGATA
>Dwil\Atp6v0e
TGTCATGTGATT
>Dalb\Atp6v0e
TGTCATGTGATA
>Dvir\Atp6v0e
TGTCATGTGATA
>Dmoj\Atp6v0e
TGTCATGTGATA
>Dgri\Atp6v0e
TGTCATGTGATA

>Dmel\Atp6v1A1
TGTCAGCTGACC
>Dmel\Atp6v1A2
TGTCAGCTGACG
>Dmel\Atp6v1A3
TGTCACGTGAAA
>Dmel\Atp6v1A4
TGTCAGCTGACA
>Dsim\Atp6v1A1
TGTCAGCTGACC
>Dsim\Atp6v1A2
TGTCAGCTGACG
>Dsim\Atp6v1A3
TGTCACGTGAAA
>Dsim\Atp6v1A4
TGTCAGCTGACA
>Dsec\Atp6v1A1
TGTCAGCTGACC
>Dsec\Atp6v1A2
TGTCAGCTGACG
>Dsec\Atp6v1A3
TGTCACGTGAAA
>Dsec\Atp6v1A4
TGTCAGCTGACA
>Dyak\Atp6v1A1
TGTCAGCTGACC
>Dyak\Atp6v1A2
TGTCAGCTGACG
>Dyak\Atp6v1A3
TGTCACGTGAAG
>Dyak\Atp6v1A4
TGTCAGCTGACA
>Dere\Atp6v1A1
TGTCAGCTGACC
>Dere\Atp6v1A2
TGTCAGCTGACG

>Dere\Atp6v1A3
CGTCACGTGAAA
>Dere\Atp6v1A4
TGTCAGCTGACA
>Dbia\Atp6v1A1
TGTCAGCTGACC
>Dbia\Atp6v1A2
TGTCAGCTGACG
>Dbia\Atp6v1A3
TGTCACGTGAAC
>Dbia\Atp6v1A4
CGTCAGCTGACG
>Dtak\Atp6v1A1
TGTCAGCTGACC
>Dtak\Atp6v1A2
TGTCAGCTGACG
>Dtak\Atp6v1A3
TGTCACGTGAAA
>Dtak\Atp6v1A4
TGTCAGCTGACA
>Deug\Atp6v1A1
TGTCAGCTGACC
>Deug\Atp6v1A2
TGTCAGCTGACG
>Deug\Atp6v1A3
TGTCACGTGAAA
>Deug\Atp6v1A4
TGTCAGCTGACA
>Dele\Atp6v1A1
TGTCAGCTGACC
>Dele\Atp6v1A2
TGTCAGCTGACG
>Dele\Atp6v1A3
TGTCACGTGAAA
>Dele\Atp6v1A4
TGTCAGCTGACA
>Drho\Atp6v1A1
TGTCAGCTGACC
>Drho\Atp6v1A2
TGTCAGCTGACG
>Drho\Atp6v1A3
TGTCACGTGAAA
>Drho\Atp6v1A4
TGTCAGCTGACA
>Dfic\Atp6v1A1
TGTCAGCTGACC
>Dfic\Atp6v1A2
CGTCAGCTGACG
>Dfic\Atp6v1A3
TGTCACGTGAAA
>Dfic\Atp6v1A4
TGTCAGCTGACA
>Dkik\Atp6v1A1
TGTCAGCTGACC
>Dkik\Atp6v1A2
TGTCAGCTGACG
>Dkik\Atp6v1A3
TGTCACGTGAAA
>Dkik\Atp6v1A4
TGTCAGCTGACA
>Dbip\Atp6v1A1
TGTCAGCTGACC
>Dbip\Atp6v1A2

TGTCAGCTGACA
>Dbip\Atp6v1A3
TGTCACGTGAAC
>Dbip\Atp6v1A4
CGTCAGCTGACA
>Dana\Atp6v1A1
TGTCAGCTGACC
>Dana\Atp6v1A2
TGTCAGCTGACA
>Dana\Atp6v1A3
TGTCACGTGAAC
>Dana\Atp6v1A4
CGTCAGCTGACA
>Dpse\Atp6v1A1
TGTCAGCTGACC
>Dpse\Atp6v1A2
TGTCAGCTGACG
>Dpse\Atp6v1A3
TGTCACGTGAAA
>Dpse\Atp6v1A4
TGTCAGCTGACA
>Dper\Atp6v1A1
TGTCAGCTGACC
>Dper\Atp6v1A2
TGTCAGCTGACG
>Dper\Atp6v1A3
TGTCACGTGAAA
>Dper\Atp6v1A4
TGTCAGCTGACA
>Dmir\Atp6v1A1
TGTCAGCTGACC
>Dmir\Atp6v1A2
TGTCAGCTGACG
>Dmir\Atp6v1A3
TGTCACGTGAAA
>Dmir\Atp6v1A4
TGTCAGCTGACA
>Dwil\Atp6v1A1
TGTCAGCTGACC
>Dwil\Atp6v1A2
TGTCAGCTGACA
>Dwil\Atp6v1A3
TGTCACGTGAAA
>Dalb\Atp6v1A1
AGTCAGCTGACC
>Dalb\Atp6v1A2
TGTCAGCTGACA
>Dalb\Atp6v1A4
TGTCAGCTGACA
>Dmoj\Atp6v1A1
AGTCAGCTGACC
>Dmoj\Atp6v1A2
TGTCAGCTGACA
>Dmoj\Atp6v1A3
TGTCACGTGAAA
>Dmoj\Atp6v1A4
TGTCAGCTGACA
>Dvir\Atp6v1A1
AGTCAGCTGACC
>Dvir\Atp6v1A2
CGTCAGCTGACA
>Dvir\Atp6v1A3
TGTCACGTGAAA

>Dvir\Atp6v1A4
TGTCAGCTGACA
>Dgri\Atp6v1A1
AGTCAGCTGACC
>Dgri\Atp6v1A2
TGTCAGCTGACA
>Dgri\Atp6v1A3
TGTCACGTGAAA
>Dgri\Atp6v1A4
TGTCAGCTGACA

>Dmel\Atp6v1B1
GGTCATGTGACG
>Dmel\Atp6v1B2
CGTCACATGACC
>Dmel\Atp6v1B3
AGTCAGCTGACA
>Dmel\Atp6v1B4
TTTCATGTGAGC
>Dsim\Atp6v1B1
GGTCATGTGACG
>Dsim\Atp6v1B2
CGTCACATGACC
>Dsim\Atp6v1B3
AGTCAGCTGACA
>Dsim\Atp6v1B4
TTTCATGTGAGC
>Dsec\Atp6v1B1
GGTCATGTGACG
>Dsec\Atp6v1B2
CGTCACATGACC
>Dsec\Atp6v1B3
AGTCAGCTGACA
>Dsec\Atp6v1B4
TTTCATGTGAGC
>Dyak\Atp6v1B1
GGTCATGTGACG
>Dyak\Atp6v1B2
CGTCACATGACC
>Dyak\Atp6v1B3
AGTCAGCTGACA
>Dyak\Atp6v1B4
TTTCATGTGAGC
>Dere\Atp6v1B1
GGTCATGTGACG
>Dere\Atp6v1B2
CGTCACATGACC
>Dere\Atp6v1B3
AGTCAGCTGACA
>Dere\Atp6v1B4
TTTCATGTGAGC
>Dbia\Atp6v1B1
GGTCATGTGATG
>Dbia\Atp6v1B2
CGTCACATGACC
>Dbia\Atp6v1B3
AGTCAGCTGACA
>Dbia\Atp6v1B4
TTTCATGTGAGC
>Dsuz\Atp6v1B1
GGTCATGTGATG
>Dsuz\Atp6v1B2
CGTCACATGACC

>Dsuz\Atp6v1B3
AGTCAGCTGACA
>Dsuz\Atp6v1B4
TTTCATGTGAGC
>Dtak\Atp6v1B1
GGTCATGTGATG
>Dtak\Atp6v1B2
CGTCACATGACC
>Dtak\Atp6v1B3
AGTCAGCTGACA
>Dtak\Atp6v1B4
TTTCATGTGAGC
>Deug\Atp6v1B1
GGTCATGTGACG
>Deug\Atp6v1B2
CGTCACATGACC
>Deug\Atp6v1B3
AGTCAGCTGACA
>Deug\Atp6v1B4
TTTCATGTGAGC
>Dele\Atp6v1B1
GGTCATGTGACG
>Dele\Atp6v1B2
CGTCACATGACC
>Dele\Atp6v1B3
AGTCAGCTGACA
>Dele\Atp6v1B4
TTTCATGTGAGC
>Drho\Atp6v1B1
GGTCATGTGACG
>Drho\Atp6v1B2
CGTCACATGACC
>Drho\Atp6v1B3
AGTCAGCTGACA
>Drho\Atp6v1B4
TTTCATGTGAGC
>Dfic\Atp6v1B1
GGTCATGTGACG
>Dfic\Atp6v1B2
CGTCACATGACC
>Dfic\Atp6v1B3
AGTCAGCTGACA
>Dfic\Atp6v1B4
TTTCATGTGAGC
>Dkik\Atp6v1B1
AGTCATGTGACG
>Dkik\Atp6v1B2
CGTCACATGACC
>Dkik\Atp6v1B3
AGTCAGCTGACA
>Dkik\Atp6v1B4
TTTCATGTGAGC
>Dbip\Atp6v1B1
TGTCATGTGACG
>Dbip\Atp6v1B2
CGTCACATGACC
>Dbip\Atp6v1B3
AGTCAGCTGACA
>Dbip\Atp6v1B4
TTTCATGTGAGC
>Dana\Atp6v1B1
TGTCATGTGACG
>Dana\Atp6v1B2

CGTCACATGACC
>Dana\Atp6v1B3
AGTCAGCTGACA
>Dana\Atp6v1B4
TTTCATGTGAGC
>Dpse\Atp6v1B1
AGTCATGTGATG
>Dpse\Atp6v1B2
CGTCACATGACT
>Dpse\Atp6v1B3
AGTCAGCTGACA
>Dpse\Atp6v1B4
GTTCATGTGAGC
>Dper\Atp6v1B1
AGTCATGTGATG
>Dper\Atp6v1B2
CGTCACATGACT
>Dper\Atp6v1B3
AGTCAGCTGACA
>Dper\Atp6v1B4
GTTCATGTGAGC
>Dmir\Atp6v1B1
AGTCATGTGATG
>Dmir\Atp6v1B2
CGTCACATGACT
>Dmir\Atp6v1B3
AGTCAGCTGACA
>Dmir\Atp6v1B4
GTTCATGTGAGC
>Dwil\Atp6v1B1
GGTCATGTGATG
>Dwil\Atp6v1B2
TGTCACATGACT
>Dwil\Atp6v1B3
AGTCAGCTGACA
>Dwil\Atp6v1B4
TTTCATGTGAGC
>Dalb\Atp6v1B1
AGTCATGTGATG
>Dalb\Atp6v1B2
TGTCACATGACT
>Dalb\Atp6v1B3
AGTCAGCTGACT
>Dalb\Atp6v1B4
TTTCATGTGAGC
>Dmoj\Atp6v1B1
AGTCATGTGACG
>Dmoj\Atp6v1B2
TGTCACATGACT
>Dmoj\Atp6v1B3
TGTCAGCTGACT
>Dmoj\Atp6v1B4
ATTCATGTGAAG
>Dvir\Atp6v1B1
AGTCATGTGACG
>Dvir\Atp6v1B2
TGTCACATGACT
>Dvir\Atp6v1B3
AGTCAGCTGACT
>Dvir\Atp6v1B4
ATTCATGTGAGC
>Dgri\Atp6v1B1
AGTCATGTGACG

>Dgri\Atp6v1B2
TGTCACATGACT
>Dgri\Atp6v1B3
AGTCAGCTGACT
>Dgri\Atp6v1B4
GTGCATGTGAGC

>Dmel\Atp6v1C1
CATCACGTGAAT
>Dmel\Atp6v1C2
TGTCATGTGACT
>Dsim\Atp6v1C1
CATCACGTGAAT
>Dsim\Atp6v1C2
TGTCATGTGACT
>Dsec\Atp6v1C1
CATCACGTGAAT
>Dsec\Atp6v1C2
TGTCATGTGACT
>Dyak\Atp6v1C1
CATCACGTGAAT
>Dyak\Atp6v1C2
TGTCATGTGACT
>Dere\Atp6v1C1
CATCACGTGAAT
>Dere\Atp6v1C2
TGTCATGTGACT
>Dbia\Atp6v1C1
CATCACGTGAAT
>Dbia\Atp6v1C2
TGTCATGTGACT
>Dtak\Atp6v1C1
CATCACGTGAAT
>Dtak\Atp6v1C2
TGTCATGTGACT
>Deug\Atp6v1C1
CATCACGTGAAT
>Deug\Atp6v1C2
TGTCATGTGACT
>Dele\Atp6v1C1
TATCACGTGAAT
>Dele\Atp6v1C2
TGTCATGTGACT
>Drho\Atp6v1C1
TATCACGTGAAT
>Drho\Atp6v1C2
TGTCATGTGACT
>Dfic\Atp6v1C1
CATCACGTGAAT
>Dfic\Atp6v1C2
TGTCATGTGACT
>Dkik\Atp6v1C1
TATCACGTGAAT
>Dkik\Atp6v1C2
TGTCATGTGACT
>Dbip\Atp6v1C1
CATCACGTGAAA
>Dbip\Atp6v1C2
TGTCATGTGACT
>Dana\Atp6v1C1
CATCACGTGAAT
>Dana\Atp6v1C2
TGTCATGTGACT

>Dpse\Atp6v1C1
CATCACGTGAAT
>Dpse\Atp6v1C2
TGTCATGTGACT
>Dper\Atp6v1C1
CATCACGTGAAT
>Dper\Atp6v1C2
TGTCATGTGACT
>Dmir\Atp6v1C1
CATCACGTGAAT
>Dmir\Atp6v1C2
TGTCATGTGACT
>Dwil\Atp6v1C1
TATCACGTGAAT
>Dwil\Atp6v1C2
TGTCATGTGACA
>Dmoj\Atp6v1C1
TATCACGTGAAT
>Dmoj\Atp6v1C2
TGTCATGTGACT
>Dvir\Atp6v1C1
TATCACGTGAAT
>Dvir\Atp6v1C2
TGTCATGTGACT
>Dgri\Atp6v1C1
TATCACGTGAAT
>Dgri\Atp6v1C2
TGTCATGTGACT

>Dmel\Atp6v1D1
GGTCATCTGACT
>Dmel\Atp6v1D2
AGTCACATGACA
>Dsim\Atp6v1D1
GGTCATCTGACT
>Dsim\Atp6v1D2
AGTCACATGACA
>Dsec\Atp6v1D1
GGTCATCTGACT
>Dsec\Atp6v1D2
AGTCACATGACA
>Dyak\Atp6v1D1
GGTCATCTGACT
>Dyak\Atp6v1D2
AGTCACATGACA
>Dere\Atp6v1D1
GGTCATCTGACT
>Dere\Atp6v1D2
AGTCACATGACA
>Dbia\Atp6v1D1
GGTCATCTGACT
>Dbia\Atp6v1D2
GGTCACATGACA
>Dtak\Atp6v1D1
GGTCATCTGACT
>Dtak\Atp6v1D2
GGTCACATGACA
>Deug\Atp6v1D1
GGTCATCTGACT
>Deug\Atp6v1D2
AGTCACATGACA
>Dele\Atp6v1D1
GGTCATCTGACT

>Dele\Atp6v1D2
AGTCACATGACA
>Drho\Atp6v1D1
GGTCATCTGACT
>Drho\Atp6v1D2
GGTCACATGACA
>Dfic\Atp6v1D1
GGTCATCTGACT
>Dfic\Atp6v1D2
AGTCACATGACA
>Dkik\Atp6v1D1
GGTCATCTGACT
>Dkik\Atp6v1D2
AGTCACATGACA
>Dbip\Atp6v1D1
GGTCATCTGACT
>Dbip\Atp6v1D2
GGTCACATGACA
>Dana\Atp6v1D1
GGTCATCTGACT
>Dana\Atp6v1D2
GGTCACATGACA
>Dpse\Atp6v1D1
AGTCATCTGACT
>Dpse\Atp6v1D2
GGTCACATGACT
>Dper\Atp6v1D1
AGTCATCTGACT
>Dper\Atp6v1D2
GGTCACATGACT
>Dmir\Atp6v1D1
AGTCATCTGACT
>Dmir\Atp6v1D2
GGTCACATGACT
>Dwil\Atp6v1D1
GGTCATCTGACT
>Dwil\Atp6v1D2
AATCACATGACT
>Dalb\Atp6v1D1
GGTCATCTGACT
>Dalb\Atp6v1D2
AGTCATGTGACA
>Dmoj\Atp6v1D1
GGTCATCTGACT
>Dmoj\Atp6v1D2
AGTCATGTGACA
>Dvir\Atp6v1D1
GGTCATCTGACT
>Dvir\Atp6v1D2
AGTCATGTGACA
>Dgri\Atp6v1D1
GGTCATCTGACT
>Dgri\Atp6v1D2
AGTCATGTGACA

>Dmel\Atp6v1E1
AATCATATGATC
>Dmel\Atp6v1E2
GGTCATGTGAAC
>Dmel\Atp6v1E3
GGTCATGTGACA
>Dmel\Atp6v1E4
TATCATGTGACA

>Dsim\Atp6v1E1
AATCATATGATC
>Dsim\Atp6v1E2
GGTCATGTGAAC
>Dsim\Atp6v1E3
GGTCATGTGACA
>Dsim\Atp6v1E4
TATCATGTGACA
>Dsec\Atp6v1E1
AATCATATGATC
>Dsec\Atp6v1E2
GGTCATGTGAAC
>Dsec\Atp6v1E3
GGTCATGTGACA
>Dsec\Atp6v1E4
TATCATGTGACA
>Dyak\Atp6v1E1
AATCATATGATC
>Dyak\Atp6v1E2
GGTCATGTGAAC
>Dyak\Atp6v1E3
GGTCATGTGACA
>Dyak\Atp6v1E4
TATCATGTGACA
>Dere\Atp6v1E1
AATCATATGATC
>Dere\Atp6v1E2
GGTCATGTGAAC
>Dere\Atp6v1E3
GGTCATGTGACA
>Dere\Atp6v1E4
TATCATGTGACA
>Dbia\Atp6v1E1
AATCATATGATC
>Dbia\Atp6v1E2
GGTCATGTGAAC
>Dbia\Atp6v1E3
GGTCATGTGACA
>Dbia\Atp6v1E4
TATCATGTGACG
>Dsuz\Atp6v1E1
AATCATATGATC
>Dsuz\Atp6v1E2
AGTCATGTGAAC
>Dsuz\Atp6v1E3
GGTCATGTGACA
>Dsuz\Atp6v1E4
TATCATGTGACG
>Dtak\Atp6v1E1
AATCATATGATC
>Dtak\Atp6v1E2
AGTCATGTGAAC
>Dtak\Atp6v1E3
GGTCATGTGACA
>Dtak\Atp6v1E4
TATCATGTGACG
>Deug\Atp6v1E1
AATCATATGATC
>Deug\Atp6v1E2
AGTCATGTGAAC
>Deug\Atp6v1E3
GGTCATGTGACA
>Deug\Atp6v1E4

AATCATGTGACA
>Dele\Atp6v1E1
AATCATATGATC
>Dele\Atp6v1E2
AGTCATGTGAAC
>Dele\Atp6v1E3
GGTCATGTGACA
>Dele\Atp6v1E4
TATCATGTGACA
>Drho\Atp6v1E1
AATCATATGATC
>Drho\Atp6v1E2
AGTCATGTGAAC
>Drho\Atp6v1E3
GGTCATGTGACA
>Drho\Atp6v1E4
TATCATGTGACG
>Dfic\Atp6v1E1
AATCATATGATC
>Dfic\Atp6v1E2
AGTCATGTGAAC
>Dfic\Atp6v1E3
AGTCATGTGACA
>Dfic\Atp6v1E4
AATCATGTGACA
>Dkik\Atp6v1E1
AATCATATGATC
>Dkik\Atp6v1E2
AGTCATGTGAAC
>Dkik\Atp6v1E3
GGTCATGTGACA
>Dkik\Atp6v1E4
AGTCATGTGACG
>Dbip\Atp6v1E1
AATCATATGATC
>Dbip\Atp6v1E2
AGTCATGTGAAT
>Dbip\Atp6v1E3
GGTCATGTGACA
>Dbip\Atp6v1E4
TATCATGTGATG
>Dana\Atp6v1E1
AATCATATGATC
>Dana\Atp6v1E2
AGTCATGTGAAC
>Dana\Atp6v1E3
GGTCATGTGACA
>Dana\Atp6v1E4
AATCATGTGATG
>Dpse\Atp6v1E1
AATCATATGATC
>Dpse\Atp6v1E2
AGTCATGTGAAT
>Dpse\Atp6v1E3
GGTCATGTGACA
>Dper\Atp6v1E1
AATCATATGATC
>Dper\Atp6v1E2
AGTCATGTGAAT
>Dper\Atp6v1E3
GGTCATGTGACA
>Dmir\Atp6v1E1
AATCATATGATC

>Dmir\Atp6v1E2
AGTCATGTGAAT
>Dmir\Atp6v1E3
GGTCATGTGACA
>Dwil\Atp6v1E1
CATCATATGATT
>Dwil\Atp6v1E2
AGTCATGTGATA
>Dwil\Atp6v1E3
GGTCATGTGACA
>Dalb\Atp6v1E1
AATCATATGATC
>Dalb\Atp6v1E2
AGTCATGTGAAT
>Dalb\Atp6v1E3
GGTCATGTGACA
>Dmoj\Atp6v1E1
AATCATATGATC
>Dmoj\Atp6v1E2
AGTCATGTGAAT
>Dmoj\Atp6v1E3
GGTCATGTGACA
>Dmoj\Atp6v1E4
AATCATGTGATT
>Dvir\Atp6v1E1
AATCATATGATC
>Dvir\Atp6v1E2
AGTCATGTGAAT
>Dvir\Atp6v1E3
GGTCATGTGACA
>Dvir\Atp6v1E4
AATCATGTGATT
>Dgri\Atp6v1E1
AATCATATGATC
>Dgri\Atp6v1E2
AGTCATGTGAAT
>Dgri\Atp6v1E3
GGTCATGTGACA
>Dgri\Atp6v1E4
AATCATGTGATT

>Dmel\Atp6v1F
TGTCATGTGATA
>Dsim\Atp6v1F
TGTCATGTGATA
>Dsec\Atp6v1F
TGTCATGTGATA
>Dyak\Atp6v1F
TGTCATGTGATA
>Dere\Atp6v1F
TGTCATGTGATA
>Dbia\Atp6v1F
GGTCATGTGATA
>Dsuz\Atp6v1F
GGTCATGTGATA
>Dtak\Atp6v1F
GGTCATGTGACA
>Deug\Atp6v1F
GGTCATGTGATA
>Dele\Atp6v1F
GGTCATGTGATA
>Drho\Atp6v1F
GGTCATGTGATA

>Dfic\Atp6v1F
GGTCATGTGATA
>Dkik\Atp6v1F
GTTCATGTGAAG
>Dbip\Atp6v1F
TGTCATGTGATA
>Dana\Atp6v1F
TGTCATGTGATA
>Dpse\Atp6v1F
GGTCATGTGATA
>Dper\Atp6v1F
GGTCATGTGATA
>Dmir\Atp6v1F
GGTCATGTGATA
>Dwil\Atp6v1F
TGTCAGCTGACC
>Dmoj\Atp6v1F
CGTCAGCTGACA
>Dvir\Atp6v1F
CGTCAGCTGACA
>Dgri\Atp6v1F
AGTCAGCTGACA

>Dmel\Atp6v1G1
CGTCATATGACA
>Dmel\Atp6v1G2
GGTCACATGATG
>Dsim\Atp6v1G1
CGTCATATGACA
>Dsim\Atp6v1G2
GGTCACATGATG
>Dsec\Atp6v1G1
CGTCATATGACA
>Dsec\Atp6v1G2
GGTCACATGATG
>Dyak\Atp6v1G1
CGTCATATGACA
>Dyak\Atp6v1G2
GGTCACATGATG
>Dere\Atp6v1G1
CGTCATATGACA
>Dere\Atp6v1G2
GGTCACATGATG
>Dbia\Atp6v1G1
CGTCATATGACA
>Dbia\Atp6v1G2
GGTCACATGATG
>Dsuz\Atp6v1G1
CGTCATATGACA
>Dsuz\Atp6v1G2
GGTCACATGATG
>Dtak\Atp6v1G1
CGTCATATGACA
>Dtak\Atp6v1G2
GGTCACATGATG
>Deug\Atp6v1G1
CGTCATATGACA
>Deug\Atp6v1G2
GGTCACATGATG
>Dele\Atp6v1G1
CGTCATATGACA
>Dele\Atp6v1G2
GGTCACATGATG

>Drho\Atp6v1G1
CGTCATATGACA
>Drho\Atp6v1G2
GGTCACATGATG
>Dfic\Atp6v1G1
CGTCATATGACA
>Dfic\Atp6v1G2
GGTCACATGATG
>Dkik\Atp6v1G1
CGTCATATGACA
>Dkik\Atp6v1G2
GGTCACATGATG
>Dbip\Atp6v1G1
CGTCATATGACA
>Dbip\Atp6v1G2
GGTCACATGATG
>Dana\Atp6v1G1
CGTCATATGACA
>Dana\Atp6v1G2
GGTCACATGATG
>Dpse\Atp6v1G1
AGTCATATGACA
>Dpse\Atp6v1G2
GGTCACATGACT
>Dper\Atp6v1G1
AGTCATATGACA
>Dper\Atp6v1G2
GGTCACATGACT
>Dmir\Atp6v1G1
AGTCATATGACA
>Dmir\Atp6v1G2
GGTCACATGACT
>Dwil\Atp6v1G1
TGTCACATGATA
>Dwil\Atp6v1G2
AGTCACATGATT
>Dalb\Atp6v1G1
CGTCATATGACA
>Dalb\Atp6v1G2
AGTCACATGATG
>Dmoj\Atp6v1G1
CGTCATATGACA
>Dmoj\Atp6v1G2
AGTCACATGATT
>Dvir\Atp6v1G1
CGTCATATGACA
>Dvir\Atp6v1G2
AGTCACATGATT
>Dgri\Atp6v1G1
CGTCATATGACA
>Dgri\Atp6v1G2
AGTCACATGATG

>Dmel\Atp6v1H1
AGTCACAAGACC
>Dmel\Atp6v1H2
AGTCAGCTGAAT
>Dsim\Atp6v1H1
AGTCACAAGACC
>Dsim\Atp6v1H2
AGTCAGCTGAAT
>Dsec\Atp6v1H1
AGTCACAAGACC

>Dsec\Atp6v1H2
AGTCAGCTGAAT
>Dyak\Atp6v1H1
AGTCACAAGACC
>Dyak\Atp6v1H2
AGTCAGCTGAAT
>Dere\Atp6v1H1
AGTCACAAGACC
>Dere\Atp6v1H2
AGTCAGCTGAAT
>Dbia\Atp6v1H1
AGTCACAAGACC
>Dbia\Atp6v1H2
AGTCAGCTGAAT
>Dsuz\Atp6v1H1
AGTCACAAGACC
>Dsuz\Atp6v1H2
AGTCAGCTGAAT
>Dtak\Atp6v1H1
AGTCACAAGACC
>Dtak\Atp6v1H2
AGTCAGCTGAAT
>Deug\Atp6v1H1
AATCACAAGACC
>Deug\Atp6v1H2
AGTCAGCTGAAT
>Dele\Atp6v1H1
AGTCACAAGACC
>Dele\Atp6v1H2
AGTCAGCTGAAT
>Drho\Atp6v1H1
AGTCACAAGACC
>Drho\Atp6v1H2
AGTCAGCTGAAT
>Dfic\Atp6v1H1
AGTCACAAGACC
>Dfic\Atp6v1H2
AGTCAGCTGAAT
>Dkik\Atp6v1H1
AGTCACAAGACC
>Dkik\Atp6v1H2
AGTCAGCTGAAT
>Dbip\Atp6v1H1
AGTCACAAGACC
>Dbip\Atp6v1H2
AGTCAGCTGAAT
>Dana\Atp6v1H1
AGTCACAAGACC
>Dana\Atp6v1H2
AGTCAGCTGAAT
>Dper\Atp6v1H1
AGTCACAAGACC
>Dper\Atp6v1H2
AGTCAGCTGAAT
>Dper\Atp6v1H1
AGTCACAAGACC
>Dper\Atp6v1H2
AGTCAGCTGAAT
>Dmir\Atp6v1H1
AGTCACAAGACC
>Dmir\Atp6v1H2
AGTCAGCTGAAT
>Dwil\Atp6v1H1

AGTCACAAGACC
>Dwil\Atp6v1H2
AGTCAGCTGAAT
>Dalb\Atp6v1H1
AGTCACAAGACC
>Dalb\Atp6v1H2
AGTCAGCTGAAT
>Dmoj\Atp6v1H1
AGTCACAAGACC
>Dmoj\Atp6v1H2
AGTCAGCTGAAT
>Dvir\Atp6v1H1
AATCACAAGACC
>Dvir\Atp6v1H2
AGTCAGCTGAAT
>Dgri\Atp6v1H1
AGTCACAAGACC
>Dgri\Atp6v1H2
AGTCAGCTGAAT

>Dmel\Atp6ap1
CATCACATGACC
>Dsim\Atp6ap1
CATCACATGACC
>Dsec\Atp6ap1
CATCACATGACC
>Dyak\Atp6ap1
CATCACATGACC
>Dere\Atp6ap1
CATCACATGACC
>Dbia\Atp6ap1
CATCACATGACC
>Dsuz\Atp6ap1
CATCACATGACC
>Dtak\Atp6ap1
CATCACATGACC
>Deug\Atp6ap1
CATCACATGACC
>Dele\Atp6ap1
CATCACATGACC
>Drho\Atp6ap1
CATCACATGACC
>Dfic\Atp6ap1
CATCACATGACC
>Dkik\Atp6ap1
CATCACATGACC
>Dbip\Atp6ap1
CATCACATGACC
>Dana\Atp6ap1
CATCACATGACC
>Dpse\Atp6ap1
GATCACATGATT
>Dper\Atp6ap1
GATCACATGATT
>Dmir\Atp6ap1
GATCACATGATT
>Dwil\Atp6ap1
AATCACATGACC
>Dmoj\Atp6ap1
CATCACATGACC
>Dvir\Atp6ap1
CATCACATGACC

>Dgri\Atp6ap1
CATCACATGACA

>Dmel\Atp6ap2a
CATCACATGATA
>Dmel\Atp6ap2b
CGTCATATGATT
>Dsim\Atp6ap2a
CATCACATGATA
>Dsim\Atp6ap2b
CGTCATATGATT
>Dsec\Atp6ap2a
CATCACATGATA
>Dsec\Atp6ap2b
CGTCATATGATT
>Dyak\Atp6ap2a
CATCACATGATA
>Dyak\Atp6ap2b
CGTCATATGATT
>Dere\Atp6ap2a
CATCACATGATA
>Dere\Atp6ap2b
CGTCATATGATT
>Dbia\Atp6ap2a
CATCACATGATA
>Dbia\Atp6ap2b
CGTCATATGATT
>Dsuz\Atp6ap2a
CATCACATGATA
>Dsuz\Atp6ap2b
CGTCATATGATT
>Dtak\Atp6ap2a
CATCACATGATA
>Dtak\Atp6ap2b
CGTCATATGATT
>Deug\Atp6ap2a
CATCACATGATA
>Deug\Atp6ap2b
CGTCATATGATT
>Dele\Atp6ap2a
CATCACATGATA
>Dele\Atp6ap2b
CGTCATATGATT
>Drho\Atp6ap2a
CATCACATGATA
>Drho\Atp6ap2b
CGTCATATGATT
>Dfic\Atp6ap2a
CATCACATGATA
>Dfic\Atp6ap2b
CGTCATATGATT
>Dkik\Atp6ap2a
CATCACATGATA
>Dkik\Atp6ap2b
TGTCATATGATT
>Dbip\Atp6ap2a
CATCACATGATA
>Dbip\Atp6ap2b
CGTCATATGATT
>Dana\Atp6ap2a
CATCACATGATA
>Dana\Atp6ap2b
CGTCATATGATT

>Dpse\Atp6ap2a
CATCACATGATA
>Dpse\Atp6ap2b
CGTCATATGATT
>Dper\Atp6ap2a
CATCACATGATA
>Dper\Atp6ap2b
CGTCATATGATT
>Dmir\Atp6ap2a
CATCACATGATA
>Dmir\Atp6ap2b
CGTCATATGATT
>Dwil\Atp6ap2a
TGTCACATGACA
>Dwil\Atp6ap2b
TGTCATATGATT
>Dalb\Atp6ap2a
CATCACATGACA
>Dalb\Atp6ap2b
CGTCATATGATT
>Dmoj\Atp6ap2a
CATCACATGACA
>Dmoj\Atp6ap2b
CGTCATATGATT
>Dvir\Atp6ap2a
CATCACATGACA
>Dvir\Atp6ap2b
CGTCATATGATT
>Dgri\Atp6ap2a
CATCACATGACA
>Dgri\Atp6ap2b
CGTCATATGATT

>Dmel\Tmem199
TCTCATGTGACA
>Dsec\Tmem199
TCTCATGTGACA
>Dsim\Tmem199
TCTCATGTGACA
>Dyak\Tmem199
TCTCATGTGACA
>Dere\Tmem199
TCTCATGTGACA
>Dbia\Tmem199
TCTCATGTGACA
>Dsuz\Tmem199
TCTCATGTGACA
>Dtak\Tmem199
TCTCATGTGACA
>Deug\Tmem199
TCTCATGTGACA
>Dele\Tmem199
CCTCATGTGACA
>Drho\Tmem199
GCTCATGTGACA
>Dfic\Tmem199
CCTCATGTGACA
>Dkik\Tmem199
ACTCATGTGACA
>Dbip\Tmem199
ACTCATGTGACA
>Dana\Tmem199

ACTCATGTGACA
>Dpse\Tmem199
AGTCATGTGACA
>Dper\Tmem199
AGTCATGTGACA
>Dmir\Tmem199
AGTCATGTGACA
>Dwil\Tmem199
GCTCATGTGATG
>Dalb\Tmem199
ACTCACATGACG
>Dmoj\Tmem199
ACTCATGTGACA
>Dvir\Tmem199
ACTCATGTGACA
>Dgri\Tmem199
ACTCATGTGACA
>Dmel\Vma21
TATCATGTGATT
>Dsim\Vma21
TATCATGTGATT
>Dsec\Vma21
TATCATGTGATT
>Dyak\Vma21
TATCATGTGATT
>Dere\Vma21
TATCATGTGATT
>Dbia\Vma21
TATCATGTGATT
>Dsuz\Vma21
TATCATGTGATT
>Dtak\Vma21
TATCATGTGATT
>Dfic\Vma21
TATCATGTGATT
>Deug\Vma21
TATCATGTGATT
>Dele\Vma21
TATCATGTGACT
>Drho\Vma21
TATCATGTGATT
>Dkik\Vma21
TATCATGTGATT
>Dbip\Vma21
TATCATGTGATT
>Dana\Vma21
TATCATGTGATT
>Dpse\Vma21
TATCATGTGATT
>Dper\Vma21
TATCATGTGATT
>Dmir\Vma21
TATCATGTGATT
>Dwil\Vma21
CATCATGTGATT
>Dalb\Vma21
TATCATGTGATT
>Dmoj\Vma21
TATCATGTGATT
>Dvir\Vma21
TATCATGTGATT
>Dgri\Vma21
TATCATGTGATT
