# Supplementary material for: Structural and Comparative Analyses of Insects Suggest the Presence of an Ultra-Conserved Regulatory Element of the Genes Encoding Vacuolar-Type ATPase Subunits and Assembly Factors
Source: Biology (Basel). 2023 Aug 13;12(8):1127. doi: 10.3390/biology12081127 (PMC10452791; doi:10.3390/biology12081127)
Supplement: Supplementary file 1 [file biology-12-01127-s001.zip › Supplemental Table S1.pdf]

Supplementary Table S1. Distribution of the conserved CLEAR elements in the transcriptional unit of V-type ATPase genes in *D. melanogaster*.

Gene cluster	Gene name	Predicted function	CLEAR element^	Position*
Atp6v1A	Vha68-2 (CG3762)	subunit A	cttgTCAGCTGAcccc	470
			tttgTCAGCTGAcggc	691
			tctgTCACGTGAaacg	981
			cttgTCAGCTGAcaga	1177
Atp6v1B	Vha55 (CG17369)	subunit B	cgggTCATGTGAcggc	45
			gccgTCACATGAccgt	701
			acagTCAGCTGAcata	1052
			acttTCATGTGAgcat	1141
Atp6v1C	Vha44 (CG8048)	subunit C	gccaTCACGTGAatct	-40
			actgTCATGTGActgt	565
Atp6v1D	Vha36-1 (CG8186)	subunit D	agggTCATCTGActtg	1142
			acagTCACATGAcagc	1193
Atp6v1E	Vha26 (CG1088)	subunit E	gaaaTCATATGAtcga	48
			caggTCATGTGAactt	447
			ctggTCATGTGAcacg	614
			gttaTCATGTGAcaca	794
Atp6v1F	Vha14-1 (CG8210)	subunit F	tgtgTCATGTGAtaga	-3
Atp6v1G	Vha13 (CG6213)	subunit G	ttcgTCATATGAcatt	9
			tgggTCACATGAtgtg	317
Atp6v1H	VhaSFD (CG17332)	subunit H	ccagTCAGCTGAattt	36
Atp6v0a	Vha100-2 (CG18617)	subunit a	tctaTCATGTGAtctt	685
Atp6v0b	VhaPPA1-1 (CG7007)	subunit b	tacgTCATGTGAttgt	47
Atp6v0c	Vha16-1 (CG3161)	subunit c	ctaaTCACGTGAgtga	130
			gcggTCATGTGAggct	165
			acgaTCACATGAgtgc	2528
			cgaaTCACATGActcg	4381
			accgTCACATGAaact	4527
			gcgaTCATGTGActgc	4906
Atp6v0d	VhaAC39-1 (CG2934)	subunit d	aaaaTCATATGActtg	47
			tgggTCACATGAccag	437
Atp6v0e	VhaM9.7-b (CG7625)	subunit e	tccgTCATGTGAtatt	38
Atp6ap1	VhaAC45 (CG8029)	accessory protein 1	cgcaTCACATGAccgt	-4
Atp6ap2	VhaM8.9 (CG8444)	accessory protein 2	ttcaTCACATGAtagt	-1
			ttcgTCATATGAttaa	52
Vma21	CG5969	assembly integral membrane	tttaTCATGTGAtttg	-11
Tmem199	CG7071	vacuolar ER assembly factor, Vma12	tttcTCATGTGAcagc	6
CCDC115	CG14671	V-type ATPase complex assemblyCCDC115	gcgaTCATATGAttga	-12

^Only canonical CLEAR elements are reported. *Position refers to the transcriptional start site
