# Supplementary material for: Structural and Comparative Analyses of Insects Suggest the Presence of an Ultra-Conserved Regulatory Element of the Genes Encoding Vacuolar-Type ATPase Subunits and Assembly Factors
Source: Biology (Basel). 2023 Aug 13;12(8):1127. doi: 10.3390/biology12081127 (PMC10452791; doi:10.3390/biology12081127)
Supplement: Supplementary file 1 [file biology-12-01127-s001.zip › Supplementary Files S20-S37/Supp_File_S20_Atp6v1A_INS.pdf]

Gene Cluster ID	Atp6v1A
**Drosophila melanogaster gene**	**Vha68-2 (CG3762)**
FlyBase ID	FBgn0263598
**Predicted function**	**ATPase, H+ transporting, lysosomal 70kDa, V1 subunit A**

Atp6v1A CLEAR element conservation:

```
         ____5'/intron1__   ____5'/intron1_   ____intron1_____   ____intron1_____
   Dmel\ CTTGTCAGCTGACCCC   TTTGTCAGCTGACGGC  TCTGTCACGTGAAACG   CTTGTCAGCTGACAGA
   Mdom\ AAAATCATGTGAAACA   ----------------  AATGTCACGTGAAATG   AAAGTCAGCTGACAAG
   Ccap\ CATGTCAGCTGACCCC   GCCGTCAGCTGACACT  TTGGTCACGTGAATAA   GCCGTCAGCTGACTTA

         ____5'/5'UTR____   ____intron______
   Gmor\ AATGTCACGTGAAATA   AAAGTCAGCTGACAAG
   Llon\ TCTGTCAGCTGAGGTC   CAAGTCATGTGATCTG
   Ppap\ CCTGTCAGCTGAGAGT   AGAGTCATGTGATCAG
```

```
         ____intron1___ _   ____intron1_____  ____intron2_____   ___________intron2_________   ____intron2___ _
   Agam\ CAGGTCACATGACCCG   CTGATCAGCTGACCTT  GAAGTCACATGACGTG   TTATGAATCGGTTCATGTGATCCA   TGGGTCAGATGATGGT
   Aara\ CAGGTCACATGACCCG   CTGATCAGCTGACCTT  GAAGTCACATGACGTG   TTATGAATCGGTTCATGTGATCCA   TGGGTCAGATGATGGT
   Aqan\ CAGGTCACATGACCCG   CTGATCAGCTGACCTT  GAAGTCACATGACGTG   TTATGAATCGGTTCATGTGATCCA   TGGGTCAGATGATGGT
   Achr\ CAGGTCACATGACCTG   CGTATCAGCTGACCTT  GCAGTCACATGACGTG   TTATGAATCGGTTCATGTGATCTA   TGGGTCAGATGATGGG
   Aepi\ CAGGTCACATGACCTG   CGGATCAGCTGACCTT  GCAGTCACATGACGTG   TTATGAATCGGTTCATGTGATCCA   TGGGTCAGATGATGGG
   Amin\ CTGGTCACATGACCTG   CTGATCAGCTGACCTT  CCAGTCACATGACGTG   ATATGAATCGGTTCATGTGATCTG   TTGGTCAGATGATGGA
   Afun\ CAGGTCACATGACCTG   TTGATCAGCTGACCTT  GCAGTCACATGACGTG   TTATGAATCGGTTCATGTGATCTG   TTGGTCAGATGATGAT
   Aste\ CAGGTCACATGACCCG   CTGATCAGCTGACCAT  GCAGTCACATGACGTG   ACATGAATCGGTTCATGTGATCTG   TCGGTCAGATGATGGG
   Adir\ CAGGTCACATGACCTG   TTGATCAGCTGACCTT  CCAGTCACATGACGTG   AGATGAATCGGTTCATGTGATCCC   GGGGTCAGATGATGCG
   Anil\ CGGGTCACATGACCAA   TTGATCAGCTGATCTC  GAAGTCACATGACGTG   TTTTGATGTTATTCATGTGATCGC   TTGGTCAGATGACATG
   Aalb\ CGGGTCACATGACCAC   ----------------  GAAGTCACATGACGAC   ------------------------   GTGGTCAGATGACACC
   Adar\ CGGGTCACATGACCAC   ----------------  GAAGTCACATGACGAC   ------------------------   GTGGTCAGATGACTCC
   Cqui\ TTGGTCACATGACCAG   ----------------  TCAGTCACATGACTTG   ------------------------   ----------------
   Aaeg\ TTGGTCACATGACCTC   ----------------  CCAGTCACATGACTTG   ------------------------   ----------------

   Mdes\ CAAGTCATGTGACTTG
   Mdes\ ACGTTCATGTGACACC
   Mdes\ AAATTCATGTGACTCA

         ____5'/5'UTR____   ____intron1_____  ____intron1_____
   Bmor\ GGGGTCAGCTGACTTC   GGTATCATGTGACTGT  ----------------
   Msex\ TAGGTCAGCTGACTTA   TTTATCATGTGACCGA  GTAATCATGTGACTAT
   Dple\ CAGGTCAGCTGACATA   TTCATCATGTGACTGA  ACTGTCAGCTGACTTC
   Hmel\ CGGGTCAGCTGACACT   ACCATCATGTGACTCA  ----------------
   Pxyl\ CGGGTCAGCTGACTAA   CCTATCATGTGACTGG  ----------------

   Tcas\ CGTGTCAGCTGACCGT   AAATTCACGTGACGGT  GTTTTCACGTGACTTT
   Dpon\ ATGGTCAGCTGACTGT   GCATTCACGTGACTGC

         ____5'/5'UTR____   ____intron1_____  ____intron1_____
   Amel\ TCAGTCAGCTGACCTG   CTGCTCACGTGAATTT
   Aflo\ TCAGTCAGCTGACCTG   CTGCTCACGTGAATTT
   Bter\ TCAGTCAGCTGACCTG   CTGCTCACGTGAATTT
   Bimp\ TCAGTCAGCTGACCTG   CTGCTCACGTGAATTT
   Mrot\ TCAGTCAGCTGACCTG   CTGCTCACGTGAATTT
   Cflo\ TCAGTCAGCTGACCTG   CTGCTCACGTGAATCG
   Aech\ ----------------   ----------------  CTTCTCACATGAATTT
   Acep\ CGAGTCAGCTGACCTG   CTGCTCACGTGAATCG  CTTCTCACATGAATTT
   Sinv\ ----------------   ----------------  CTTTTCACGTGAATTT
   Lhum\ TCAGTCAGCTGACCTG   AATTTCACGTGAGCGA  TTGCTCACGTGAATCG
   Pbar\ TTAGTCAGCTGACCTG   TTGCTCACGTGAATCG
   Hsal\ TCAGTCAGCTGACCTG   CTGCTCACGTGAATCG
   Ngir\ GCAGTCAGCTGACCAG   CCGCTCACGTGAGAAC  CGCGTCACGTGATTTT
   Nvit\ GCAGTCAGCTGACCAG   CCGCTCACGTGAGAAC  CGCGTCACGTGATTTT
   Nlon\ GCAGTCAGCTGACCAG   CCGCTCACGTGAGAAC  CGCGTCACGTGATTTT
   Lful\ AGTGTCAGCTGACCAG

         __________5'/5'UTR?/intron1?____   ___________5'/5'UTR?/intron1______   ___________5'/5'UTR?/intron1______
   Apis\ CCAGTAGTACAATTCATATGAAAAAGTACAAT   GACGTCTTACAGTCAGCTGATTAGAGTTCCAC
   Dpul\ ATACATAGGGCTTCAGCTGAAACAAGAAAATA   TCATGTGACTGCTCACTCAGCTGACTCTGACA
   Isca\ GATTTAGTCGCTTCAGCTGATTGATGTTGTGG   TCCTGCAACCGATCATGTGATTCTTCTCGCAG   TCATGTGACAGTCGCTTCTCATATGATGACGC
   Phum\ GTGTCTTCCATGTCAGCTGACAGGTCTTGTGA
   Hsap\ CGGAGCTCGACGTCATGTGACTGGTTGGGCCC
```

Atp6v1A gene structure comparison:

Key: UTR region, CDS region **INTRON**

Order	Species	Atp6v1A orthologs	5'exon	intron	exon		intron	exon	intron	exon	intron	exon3'		Extended CLEAR region	Position	bps from TSS
Diptera (Drosophilidae)	*Drosophila_melanogaster*	Dmel\Vha68-2	124	1170	23	76	413	698	108	978	67	93	546	tattttgccttgTCAGCTGAcccccttccccg<br>tttttcatttgTCAGCTGAcggcaatgacag<br>ttcaaagctctgTCACGTGAaacgaaagctct<br>acctgccccttgTCAGCTGAcagagggctgag	intron1<br>intron1<br>intron1<br>intron1	470<br>691<br>981<br>1177
Diptera (Muscidae)	*Musca_domestica*	Mdom\Atp6v1A-1			ND	76	74	698	83	978	70	93		atttagcaaaaTCATGTGAaacatctcaaca<br>aacttccaaatgTCACGTGAaatgaaagcttt<br>taaaacgcaaagTCAGCTGAcaagagacgacg	intron1<br>intron1<br>intron1<br>intron1	470<br>691<br>981<br>1177

		Mdom\Atp6v1A-2			ND	76	66	698			75	978			61	93		ND		
		Mdom\Atp6v1A-3			ND	1932												ND		
Diptera (Tephritidae)	*Ceratitis_capitata*	Ccap\Atp6v1A-1	81	621	22	76	69	698			77	978			70	93		tgagcaaacatgTCAGCTGAccccgaatgtct<br>tgtaatcagccgTCAGCTGAcacttagcggat<br>ctaagctgttggTCACGTGAataaaaagcttt<br>gagaataagccgTCAGCTGActtatcacctgt	5'<br>5'<br>intron1<br>intron1	-284<br>-63<br>230<br>594
		Ccap\Atp6v1A-2			ND	76	222	698			61	978			174	93		ND		
Diptera (Glossinidae)	*Glossina_morsitans*	Gmor\Atp6v1A-1<br>TMP011178	133	274	55	76	93	698			60	978			73	93	144	caattccaaatgTCACGTGAaataaaagcttt<br>ggaagggaaaagTCAGCTGAcaagtgaaaaaa	5'UTR?<br>intron1	-35<br>254
		Gmor\Atp6v1A-2<br>TMP002752			ND	73	70	698			59	978			73	93		ND		
		Gmor\Atp6v1A-3<br>TMP007159			ND	1932												ND		
Diptera (Psychodidae)	*Lutzomyia_longipalpis*	Llon\Atp6v1A	93	490	23	76	71	195	2085	1084			65	397	87	93	76	tcacaagatctgTCAGCTGAggtcttgtgatt<br>atgtaactcaagTCATGTGAtctgaaaaaaat	5'UTR<br>intron1	8<br>412
	Phlebotomus_papatasi	Ppap\Atp6v1A	38	559	23	76	67	195	ND	578	ND	506	ND	397	66	93		tcacaagacctgTCAGCTGAgagtcttgtgat<br>catcggggagagTCATGTGAtcagtgaaaatt	5'UTR?<br>intron1	-41<br>414
Diptera (Culicidae)	*Anopheles_gambiae*	Agam\Atp6v1A<br>AGAP003153	30	574	67	76	735	195	75	1084			74	397	100	93		gaaccaaacaggTCACATGAcccgcagcacct<br>tttgcacactgaTCAGCTGAcctttctttgag<br>ggtcgccagaagTCACATGAcgtgtgaagtgg<br>ttatgaatcggtTCATGTGAtccactgctgcc<br>tggtggaatgggTCAGATGAtggtgtacgcca	intron1<br>intron1<br>intron2<br>intron2<br>intron2	405<br>514<br>927<br>1020<br>1248
	Anopheles_arabiensis	Ara\Atp6v1A			ND	76	724	195	75	1084			75	397	122	93		gaaccaaacaggTCACATGAcccgcagcacct<br>tttgcacactgaTCAGCTGAcctttctttgag<br>ggtcgccagaagTCACATGAcgtgtgaagtgg<br>ttatgaatcggtTCATGTGAtccactgctgcc<br>tggtggaatgggTCAGATGAtggtgtacgcca	intron1?<br>intron1?<br>intron2?<br>intron2?<br>intron2?	> -262<br>> -149<br>> 256<br>> 351<br>> 579
	Anopheles_quadriannulatus	Aqan\Atp6v1A			ND	76	731	195	75	1084			90	397	121	93		gaaccaaacaggTCACATGAcccgcagcacct<br>tttgcacactgaTCAGCTGAcctttctttgag<br>ggtcgccagaagTCACATGAcgtgtgaagtgg<br>ttatgaatcggtTCATGTGAtccactgctgcc<br>tggtggaatgggTCAGATGAtggtgtgcgcca	intron1?<br>intron1?<br>intron2?<br>intron2?<br>intron2?	>-262<br>>-149<br>>261<br>>354<br>>576
	Anopheles_christyi	Achr\Atp6v1A			ND	76	693	195	70	1084*			90	397	75	93		gagccaaacaggTCACATGAcctgcagcagca<br>ttttcactcgtaTCAGCTGAcctttctttgag<br>ggtcgccagcagTCACATGAcgtgtgaagtgg<br>ttatgaatcggtTCATGTGAtctaccactctc<br>atctggaatgggTCAGATGAtggggtggtgat	intron1?<br>intron1?<br>intron2?<br>intron2?<br>intron2?	>-252<br>>-161<br>>246<br>>321<br>>530
	Anopheles_epiroticus	Aepi\Atp6v1A			ND	76	674	195	68	1084			93	397	97	93		aaaccaaacaggTCACATGAcctgcagcagca<br>tcttcacacggaTCAGCTGAcctttctttgag<br>cgctggtcgcagTCACATGAcgtgtgaagtgg<br>ttatgaatcggtTCATGTGAtccactgtttgg<br>atctagattgggTCAGATGAtggggtgctgtt	intron1?<br>intron1?<br>intron2?<br>intron2?<br>intron2?	>-244<br>>-153<br>>228<br>>309<br>>511
	Anopheles_minimus	Amin\Atp6v1A			ND	76	606	195	65	1084			76	397	72	93		gaaccgaactggTCACATGAcctgcggttccg<br>aggcttctctgaTCAGCTGAcctttctttgag<br>gtcgctagccagTCACATGAcgtgtgaaatgg<br>atatgaatcggtTCATGTGAtctgtttgacga<br>ttctggaattggTCAGATGAtggaaggaggtg	intron1?<br>intron1?<br>intron2?<br>intron2?<br>intron2?	>-217<br>>-144<br>>238<br>>312<br>>509
	Anopheles_funestus	Afun\Atp6v1A			ND	76	616	195	67	1084			83	397	78	93		gaacctaacaggTCACATGAcctgcggttccg<br>agccttctttgaTCAGCTGAcctttcttttag<br>gtgggctagcagTCACATGAcgtgtgaaatgg<br>ttatgaatcggtTCATGTGAtctgtttgacga<br>ttttgaaattggTCAGATGAtgatgatggaag	intron1?<br>intron1?<br>intron2?<br>intron2?<br>intron2?	>-219<br>>-146<br>>233<br>>307<br>>506
	Anopheles_stephensi	Aste\Atp6v1A			ND	76	628	195	69	1084			75	397	82	93		gtaccgaacaggTCACATGAcccgcggttccg<br>cttcatcactgaTCAGCTGAccattccttcat<br>ggtcgctggcagTCACATGAcgtgtgaaatgg<br>acatgaatcggtTCATGTGAtctgtttgtgag<br>atctgaagtcggTCAGATGAtgggggagactc	intron1?<br>intron1?<br>intron2?<br>intron2?<br>intron2?	>-231<br>>-152<br>>244<br>>319<br>>521
	Anopheles_dirus	Adir\Atp6v1A			ND	76	603	195	73	1084			78	397	111	93		gtaccctacaggTCACATGAcctgcggtgatg<br>ggtcgtttttgaTCAGCTGAcctttcttgtgg<br>gtcgccagccagTCACATGAcgtgtgaagtgg<br>agatgaatcggtTCATGTGAtcccgtggttgt<br>atctggttggggTCAGATGAtgcggtttactt	intron1?<br>intron1?<br>intron2?<br>intron2?<br>intron2?	>-231<br>>-142<br>>213<br>>296<br>>485
	Anopheles_nili	Anil\Atp6v1A			ND	76	645	195	80	1084			83	397	88			aaatcaaacgggTCACATGAccaacaagctca<br>ccatcgctttgaTCAGCTGAtctcttttgaaa<br>tctctcaagaagTCACATGAcgtgtgaagtgc<br>ttttgatgttatTCATGTGAtcgctctcgagc<br>atgtgattttggTCAGATGAcatgatgatttt	intron1?<br>intron1?<br>intron2?<br>intron2?<br>intron2?	>-210<br>>-140<br>>241<br>>373<br>>570
	Anopheles_albimanus	Aalb\Atp6v1A			ND	76	644	1279					77	397	83	93		gaaacccgcgggTCACATGAccaccacctccc<br>ttgtggtcgaagTCACATGAcgacgtgtgaac<br>gctgctgggtggTCAGATGAcacccacggctg	intron1?<br>intron2?<br>intron2?	>-228<br>>191<br>>521
	Anopheles_darlingi	Adar\Atp6v1A			ND	76	646	1279					75	397	82	93		gaaacccgcgggTCACATGAccaccacctccc<br>ttgtggtcgaagTCACATGAcgacgtgtgaat<br>gcggctgggtggTCAGATGActccccctgctg	intron1?<br>intron2?<br>intron2?	>-237<br>>187<br>>512
	Aedes_aegypti	Aaeg\Atp6v1A<br>AAEL008787	127	523	48	76	9638	195	67	1084			68	397	98	93	1088	attaggatttggTCACATGAcctctaccacct<br>caaagaaaccagTCACATGActtgtatgtgta<br>agtaacatactcTCAGCTGAgcttcgttgaat	intron1 intron2<br>intron2	479<br>905<br>1004
	Culex_pipiens_qui.	Cqui\Atp6v1A<br>CPIJ007772			ND	76	5977	1279					61	397	3297	93		ttcgtaggttggTCACATGAccagcaccacct<br>acagagagtcagTCACATGActtggtggtgta	intron1?<br>intron2?	>-209<br>>207
Diptera (Cecidomyiidae)	*Mayetiola_destructor*	Mdes\Atp6v1A			ND	76	91	663			71	616	109	397 (70+137+190)	75	93		gtgaaaatcaagTCATGTGActtgtcacacag<br>agcaggtgacgtTCATGTGAcaccaagtctcc<br>ggcaattaaatTCATGTGActcacgacaattt	5'<br>5'<br>intron1?	>-709<br>>-480<br>>-256

Lepidoptera	*Bombyx_mori*	Bmor\Atp6v1A	90	2217	24	85	368	113	371	228	371	138	931	152	760	125	847	147	215	216	694	160	694	70	1031	137	939	190	353	93	285	aaatcaatgattTCAGCTGAtcatagacaaag<br>gtagagttggggTCAGCTGActtcattttaat	5'<br>5'	-117<br>-81

																																				gaaccaatggtaTCATGTGActgtaagcttaa	intron1	282
	Manduca_sexta	Msex\Atp6v1A	56	2528	19	85	164	113	482	228	238	138	237	152			286	125	254	147	194	216	364	160			319	70	222	137	179	190	400	93		catagagctaggTCAGCTGActtaatttttat<br>acgcccattttaTCATGTGAccgaaaaaacac<br>gtatctgcgtaaTCATGTGActatagatatga	5'<br>intron1<br>intron1	24<br>365<br>604
	Heliconius_melpomene	Hmel\Atp6v1A	35	2066	15	85	226	113	585	228	330	138	485	277 (152+125)					361	147	394	216	196	160			223	70	1862	137	122	190	232	93		catagactcgggTCAGCTGAcactacttttat<br>cccttgttaccaTCATGTGActcaaattagaa	5'<br>intron1	-43<br>323
	Danaus_plexippus	Dple\Atp6v1A	63	1531	22	85	97	113	160	228	88	415 (138+152+125)							115	730											84	190	71	93		catagactcaggTCAGCTGAcatattttttat<br>accacccattcaTCATGTGActgattaataaa<br>agatagcgactgTCAGCTGActtccaactcta	5'<br>intron1<br>intron1	-17<br>363<br>1329
	Plutella_xylostella	Pxyl\Atp6v1A			ND	79	1529	113	685	228	2546	138	442	152			922	125	413	147	429	216	432	160			384	70	447	137	490	121+391+69	335	93		gactacatcgggTCAGCTGActaaaattttat<br>gttaccaccctaTCATGTGActggaattttgc	5'<br>intron1	24<br>365
Strepsiptera	*Mengenilla_moldrzyki*	Mmol\Atp6v1A			ND	79	63	113	ND	366(228+138)			356	622									ND	668												ND		
Coleoptera	*Tribolium_castaneum*	Tcas\Atp6v1A	69	459	17	76	49	341 (113+228)			55	138	2071	152			51	1138																	572	gtcaagatcgtgTCAGCTGAccgtcttgtgac<br>tcactacaaaatTCACGTGAcggtgcccactc<br>aaacttctgtttTCACGTGActttttgatgga	5'<br>intron1<br>intron1	-1<br>413<br>475
	Dendroctonus_ponderosae	Dpon\Atp6v1A	16	433	15	76	91	113	ND	228	59	138	60	152 (57+95)			55	125	62	246+83+203+60+162+64+163+ND+239															189	cttgttctatggTCAGCTGActgtcttgtgat<br>atttgatcgcatTCACGTGActgccttgggaa<br>atttcaattgtgTCACATGAtacttcctattg	5'<br>intron1<br>intron3	-67<br>398<br>823
Hymenoptera	*Apis_mellifera*	Amel\Atp6v1A	144	829	34	82	84	113	110	366			72	57	60	95	91	272			113	184	73	118	62	281					79	190	86	93	1954	cacatctttcagTCAGCTGAcctgtcttgtga<br>taaatgaactgcTCACGTGAattttatgatga	5'UTR<br>intron1	55<br>519
	Apis_florea	Aflo\Atp6v1A	144?	821?	34?	82	80	113	89	366			72	57	105	95	116	272			96	184	88	118	66	281					87	190	83	93		cacatctttcagTCAGCTGAcctgtcttgtga<br>taaatgaactgcTCACGTGAattttatgatga	5'UTR<br>intron1	55<br>519
	Bombus_terrestris	Bter\Atp6v1A	>30	836	34	82	75	113	88	366			74	57	63	95	61	272			82	184	80	118	93	281					83	190	80	93	202	cacatcgttcagTCAGCTGAcctgtcttgtga<br>taaatgaactgcTCACGTGAattttctgatga	5'UTR?<br>intron1	>-51<br>>410
	Bombus_impatiens	Bimp\Atp6v1A	>30	837	34	82	75	113	88	366			74	57	63	95	60	272			82	184	80	118	94	281					83	190	75	93		cacatcgttcagTCAGCTGAcctgtcttgtga<br>taaatgaactgcTCACGTGAattttctgatga	5'UTR?<br>intron1	>-51<br>>410
	Megachile_rotundata	Mrot\Atp6v1A			ND	82	70	113	91	366			89	57	69	95	78	272			91	184	78	118	69	281					69	190	67	93		cttttaaatcagTCAGCTGAcctgtcttgtga<br>taaatgaactgcTCACGTGAattttatagtga	5'?<br>5'UTR?	>840<br>>394
	Acromyrmex_echinatior	Aech\Atp6v1A			ND	82	70	113	72	366			69	57	78	95	79	272			169	184	192	118	77	281					124	190	105	93		ggttagttcttcTCACATGAattttatcttat	intron	> 93
	Atta_cephalotes	Acep\Atp6v1A			ND	82	70	113	68	366			69	57	70	95	72	272			109	184	186	118	75	281					121	190	107	93		ctcagcaccgagTCAGCTGAcctgtcttgtga<br>tgaatggcctgcTCACGTGAatcgcacagcca<br>ggttagttcttcTCACATGAatttcaccttac	5'<br>5'<br>intron	> - 1366<br>> -907<br>> 93
	Solenopsis_invicta	Sinv\Atp6v1A			18	82	70	113	765	366			81	57	308	95	72	272			228	184	213	118	82	281					98	190	92	93	455	ggttagttctttTCACGTGAattttattctgt<br>ggtttattctttTCAGCTGAacttcttatact	intron1<br>intron2	>111<br>>320
	Camponotus_floridanus	Cflo\Atp6v1A			ND	82	70	113	80	366			85	57	93	95	68	272			119	184	311	118	81	281					120	190	100	93		cagctgtaatttTCATGTGAacttatcttaat<br>gcacgcgatcagTCAGCTGAcctgtcttgtga<br>tgaatgacctgcTCACGTGAatcgtagccagc	5'<br>5'<br>5'	>1005<br>>943<br>>502
	Harpegnathos_saltator	Hsal\Atp6v1A			ND	82	81	113	155	366			105	57	94	95	101	272			521	184	255	118	97	281					126	190	116	93		cacgcaactcagTCAGCTGAcctgtcttgtga<br>ttgaacgtctgcTCACGTGAatcgccgagagc	5'<br>5'	>-962<br>>-501
	Pogonomyrmex_barbatus	Pbar\Atp6v1A			ND	82	74	113	77	366			89	57	84	95	81	272			98	184	213	118	75	281					148	190	307	93		cacgcgacttagTCAGCTGAcctgtcttgtga<br>tgaacgacttgcTCACGTGAatcgcgcagcga	5'<br>5'	>-1420<br>> -965
	Linepithema_humile	Lhum\Atp6v1A			ND	82	73	113	83	366			87	57	79	95	80	272			90	184	188	118	69	281					114	190	89	93		cacgcgattcagTCAGCTGAcctgtcttgtga<br>tttcgccgaattTCACGTGAgcgagcgaacga<br>tgaacggcttgcTCACGTGAatcgctgccagc	5'<br>5'<br>5'	>-1476<br>>-1289<br>>-1029
	Nasonia_vitripennis	Nvit\Atp6v1A	156	396	14	73	76	113	88	366			80	57	82	367					73	184	75	118	67	281					71	190	240	93	171	cagcacgtgcagTCAGCTGAccagcgcgccag<br>tacatcagccgcTCACGTGAgaacatcccgac<br>acgtacggcgcgTCACGTGAttttcaattgag	5'<br>intron1<br>intron1	- 35<br>1147<br>1513
	Nasonia_giraulti	Ngir\Atp6v1A	153	397?	14	73	76	113	88	366			80	57	82	367					73	184	75	118	67	281					71	190	240	93		cagcacgtgcagTCAGCTGAccagcgcgccag<br>tacatcagccgcTCACGTGAgaacatcccgac<br>acgtacggcgcgTCACGTGAttttcaattgag	5'<br>intron1/5'UTR<br>intron2	-1111<br>1<br>358
	Nasonia_longicornis	Nlon\Atp6v1A	153?	378	14?	73	76	113	88	366			80	57	82	367 (95+142+130)					73	184	75	118	67	281					71	190	240	93		cagcacgtgcagTCAGCTGAccagcgcgccag<br>tacatcagccgcTCACGTGAgaacatcccgac<br>acgtacggcgcgTCACGTGAttttcaattgag	5'<br>intron1<br>intron2	-1167<br>1<br>356
Hemiptera	*Acyrthosiphon_pisum*	Apis\Atp6v1A	72+798+181	1183	17	76	89	113	77	366(228+138)			63	152			61	142	132	130	87	127	64	249			189	207			60	190	59	93	830	ccagtagtacaatTCATATGAaaaagtacaat<br>gacgtcttacagTCAGCTGAttagagttccac	intron1<br>exon2(5'UTR)	437<br>889
	Rhodnius_prolixus	Rpro\Atp6v1A			ND	73	91	113	1025	228	249	138	92	152			68	142	90	130	318	127	607	249			191	207			559	190	1179	93		ND		
Phthiraptera	*Pediculus_humanus*	Phum\Atp6v1A			ND	76	248	113	73	232	95	134	78	152			75	142	98	257(130+127)			86	249			84	207			95	190	78	93		gtgtcttccatgTCAGCTGAcaggtcttgtga	5'	>-621
Odonata	*Ladona_fulva*	Lful\Atp6v1A			ND	76	1088	113	91	228	82	138	120	152			102	142	1482	130	75	127	129	249			76	207			133	190	85	93		aggagttgagtgTCAGCTGAccagtcttgtga	5'	>-576
**Crustacea**	*Daphnia_pulex*	Dpul\Atp6v1A	74	73	9	85	250	113	66	228	62	195 (138+57)			64	95	61	272			69	127	71	249			65	207			66	190	64	93	314	atacatagggctTCAGCTGAaacaagaaaata<br>TCATGTGActgctcacTCAGCTGActctgacacccg	5'<br>5'	-1042<br>-59/-42
Ixodida	*Ixodes_scapularis*	Isca\Atp6v1A			ND	73	693	129	761	212	1869	138	ND	152			ND	163	528	109	83	123	ND	115	890	208+ND+60			5136	267			ND	93		gatttagtcgctTCAGCTGAttgatgttgtgg<br>tcctgcaaccgaTCATGTGAttcttctcgcag<br>TCATGTGAcagtcgcttcTCATATGAtgacgcagtttt	5'?<br>5'?<br>5'?	> -1623<br>> -704<br>> -111
	Homo_sapiens	ATP6V1A				82	2214	129	3045	212	257	138	1398	152			2329	163	856	109	5031	123	95	115	671	64	2303	204	5136	95	1676	172	3809	93		cggagctcgacgTCATGTGActggttgggccc	5'	-21
Cnidaria	*Nematostella_vectensis*	Nvec\Atp6v1A				79	99	44+745+85	1834	66+312+44+71+102	785	195(138+57)			525	95	550	163	962	109	439	123	ND	179			499	144+190+60	346	267			523	93		ctcggtgttagcCCATGTGAtcattgtatcat	5'	-122
Placozoa	*Trichoplax_adhaerens*	Tadh\Atp6v1A				34	118	44+630+85	1834	70+158+31+82+240			96	57	211	95	117	163	85	109	147	123		179			70	144+70+60	5136	267			132	93		ND		
