# Supplementary material for: Structural and Comparative Analyses of Insects Suggest the Presence of an Ultra-Conserved Regulatory Element of the Genes Encoding Vacuolar-Type ATPase Subunits and Assembly Factors
Source: Biology (Basel). 2023 Aug 13;12(8):1127. doi: 10.3390/biology12081127 (PMC10452791; doi:10.3390/biology12081127)
Supplement: Supplementary file 1 [file biology-12-01127-s001.zip › Supplementary Files S20-S37/Supp_File_S21_Atp6v1B_INS.pdf]

Gene Cluster ID	Atp6v1B
**Drosophila melanogaster gene**	**Vha55 (CG17369)**
FlyBase ID	FBgn0005671
**Predicted function**	**ATPase, H+ transporting, lysosomal 56/58kDa, V1 subunit B**

Atp6v1B CLEAR element conservation:

```
            ____5'/5'UTR_     ____intron_      ____intron_      ____intron_
     Dmel\  CGGGTCATGTGACGGC  GCCGTCACATGACCGT  ACAGTCAGCTGACATA  ACTTTCATGTGAGCAT
     Mdom\  TTAATCACATGACAGC  AATGTCATGTGACTTG  CGAGTCAGCTGACTTT  TCTATCATGTGAAGCA
     Ccap\  TTGGTCACATGACCGC  GCCGTCATGTGACATT  GCAGTCAGCTGACGTT
     Gmor\  TCAATCACATGACAGC  TCAGTCATGTGATTAA
     Llon\  TGTGTCACATGACATT  TTGGTCATATGACACC
     Ppap\  CCAGTCACATGACATT  TTGGTCATATGACCCT
            ____5'/5'UTR_     ____intron_      __________intron_
     Agam\  TTCATCACATGACAGA  GATGTCATGTGACGGG  GGTGTTTTGATGTCACATGACTGT
     Aqan\  TTCATCACATGACAGA  GATGTCATGTGACGAG  GGTGTTTTGATGTCACATGACTGT
     Aara\  TTCATCACATGACAGA  GATGTCATGTGACGAG  GGTGTTTTGATGTCACATGACTGT
     Achr\  TTCATCACATGACAGA  GATGTCATGTGACGAG  AGTGTTTTGATGTCACATGACTGT
     Aepi\  TTCATCACATGACAGA  GGAGTCATGTGACGTG  AGTGTTTTGATGTCACATGACTGT
     Amin\  TTCATCACATGACAGA  TGTGTCATGTGACGGT  ACTGTTTTGATGTCACATGACTGT
     Afun\  TTCATCACATGACAGA  TGTGTCATGTGACGGT  ACTGTTTTGATGTCACATGACTGT
     Aste\  TTCATCACATGACAGA  TGTGTCATGTGATATG  ACTGTTTTGATGTCACATGACTGT
     Anil\  TTCATCACATGACAGT  GTCGTCATGTGACGAT  CCTGTTTTGATGTCACATGACTGT
     Adir\  ----------------  TACGTCATGTGACGGT  ACTGTTTTGATGTCACATGACTGT
     Aalb\  ----------------  ----------------  TCTGTTTTGATGTCACATGACTGT
     Adar\  ----------------  ----------------  TCTGTTTTGATGTCACATGACTGT
     Aaeg\  TGTTTCACATGACAAC  AGTGTCATGTGATAAC  TCTGTTTTGATGTCACATGACTGT
     Cqui\  GCTGTCACATGAGAGG  TGTGTCATGTGATAAC  TCTGTTTTGATGTCACATGACACC

     Mdes\  AAAGTCACATGACAGC  CATGTCATGTGACCTA  ATAATCATATGAAAAA

            ____5'/5'UTR_             ____intron_      ____intron_      ____intron_
     Bmor\  TGGGTCACGTGACAGCACGTGAAG  TCAGTCACATGAATTT
     Msex\  TGGGTCACGTGACGGCACGTGAAG  TATATCACATGATGAA
     Dple\  TAGATCACGTGACAGCACGTGAAG  AATATCACATGATCAA
     Hmel\  TAGGTCACGTGACAGCACGTGAAG  AGGATCACATGACTTC
     Pxyl\  CGGGTCACGTGACAGCACGTGAAG  GAAATCATATGACTTA

     Tcas\  GCCGTCACGTGACGACACGTGAAT  CGGGTCATATGACCAA  GTTGTCATGTGACCGA
     Dpon\  AGCATCACATGA.GTCACATGAGA  ----------------  CATGTCATGTGACATC

     Amel\  TAAGCCACGTGATCGCACGTGCCA  ATAGTCAGCTGATTCT  TCCATCATGTGACTGG
     Aflo\  TAAACCACGTGATTGCACGTGCCA  GTAGTCAGCTGATTTT  TCTATCATGTGACTAG
     Bter\  TAAGCCACGTGATCGCACGTGCCA  GTAGTCAGCTGATTCT  TGTATCATGTGACTAT
     Bimp\  TAAGCCACGTGATCGCACGTGCCA  GTAGTCAGCTGATTCT  TGTATCATGTGACTAT
     Mrot\  CAAGCCACGTGATCGCACGTGCCA  CTAATCAGCTGATTCA  TCTGTCATGTGACTGT
     Aech\  CGGGCCACGTGATTGCACGTGCCT  GGAGTCAGCTGATCTT  AGCGTCATGTGACCAT
     Acep\  CGAGCCACGTGATTGCACGTGCCT  GGAGTCAGCTGATCTC  AGCATCATGTGATCAT
     Pbar\  CGAGCCACGTGATTGCACGTGCCT  GGGGTCAGCTGATTCC  AGCATCATGTGACTGC
     Sinv\  CCGGCCACGTGATTGCACGTGCCT  CGGGTCAGCTGATCTC  AGCGTCATGTGACTGC
     Lhum\  CGAGCCACGTGATTGCACGTGCCT  TGGGTCAGCTGATCCC  AGCGTCATGTGACTGC  AATATCACATGATTTA
     Hsal\  CGAGCCACGTGATTGCACGTGCCT  TGGGTCAGCTGATCCC  GGCGTCATGTGACTAC
     Cflo\  CAAGCCACGTGATTGCACGTGCCT  TGCATCAGCTGATCCC  CTTGTCATGTGACTGC
     Ngir\  CGAGCCACGTGATCCCACGTGCTT  CTGGTCAGCTGATCGG  TGTGTCATGTGACTGC  GGAGTCATGTGACACG
     Nlon\  CGAGCCACGTGATCCCACGTGCTT  CTGGTCAGCTGATCGG  TGTGTCATGTGACTGC  GGAGTCATGTGACACG
     Nvit\  CGAGCCACGTGATCCCACGTGCTT  CTGGTCAGCTGATCGG  TGTGTCATGTGACTGC  GGAGTCATGTGACACG

     Phum\  CTTGTCATGTGATTTCACGTGATC
     Lful\  ctggTCACGTGAatTCACGTGAtt

     Dpul\  tctgacagTCATATGAtcgagaga
     Isca\                            tggtggtagcggTCATGTGActcaggatgtca
```

Atp6v1B gene structure comparison:

Legend: UTR region, CDS region INTRON

Order	Species	Atp6v1B orthologs	5'exon		intron	exon			intron	exon3'		Extended CLEAR region	Position	bps from TSS
Diptera (Drosophilidae)	*Drosophila_melanogaster*	Dmel\Vha55	308	73	1221	1262			61	138	1005	acgcgtgacgggTCATGTGAcggcacgtgacg<br>aaattttcgccgTCACATGAccgttcgctgct<br>ccagttggacagTCAGCTGAcataatttagcc<br>agattccgacttTCATGTGAgcattgcgcgac	5'UTR<br>intron1<br>intron1<br>intron1	45<br>701<br>1052<br>1141
Diptera (Muscidae)	*Musca_domestica*	Mdom\Atp6v1B	ND	79	1522	1262 (506+756)			644	138		ggatttcgttaaTCACATGAcagcacgtgaga<br>ttttactgaatgTCATGTGActtgtttttgct<br>tttgttgacgagTCAGCTGActttattgcttt<br>tggttttatctaTCATGTGAagcattaaacat	5'<br>intron1<br>intron1<br>intron1	>-331<br>>561<br>>1320<br>>1553
Diptera (Tephritidae)	*Ceratitis_capitata*	Ccap\Atp6v1B	ND	79	926	506	71	756	102	138	ND	ctctgcctttggTCACATGAccgcacgtgaca<br>catttagtgccgTCATGTGAcattggtgttgc<br>ttcaatttgcagTCAGCTGAcgttcgttacgt	5'<br>intron1<br>intron1	>-315<br>>425<br>>805
Diptera (Glossinidae)	*Glossina_morsitans*	Gmor\Atp6v1B	265	67	599	506 (119+130+132+125)	64	756 (231+103+155+138+129)	58	138	ND	acatttattcaaTCACATGAcagcacgtgagt<br>tattcttttcagTCATGTGAttaacttcagag	5'<br>intron1	53<br>656
Diptera (Psychodidae)	*Lutzomyia_longipalpis*	Llon\Atp6v1B	ND	73	85	995	1586	405			ND	caccctgaggggTCACATGAtacgtcgtttaa<br>acgatttttgtgTCACATGAcattttgtctca<br>cttccatcttggTCATATGAcacctccatatc	5'<br>5'<br>5'	>-556<br>>-511<br>>-125
	Phlebotomus_papatasi	Ppap\Atp6v1B	145+347+27	73	599	995 (119+130+132+125+231+103+155)	ND	405 (138+129+138)			325	atccgtccccagTCACATGAcatttctcctag<br>cttccatcttggTCATATGAccctcatatctc	5'<br>intron1	> -22<br>385
Diptera (Culicidae)	*Anopheles_gambiae*	Agam\Atp6v1B<br>AGAP002884	91+96+63	97	1051	326	67	1074			361	ttcgtttcttcaTCACATGAcagaattaataa<br>gcccgcaagatgTCATGTGAcgggcggtgaag<br>ggtgttttgatgTCACATGActgtcaaaacac<br>gtggtcgcgtcgTCACATGAtagatttttctt	5'<br>intron2<br>intron2<br>intron2	-20<br>467<br>822<br>1182
	Anopheles_arabiensis	Aara\Atp6v1B	ND	97	1054	326	67	1074			ND	ttcgattcttcaTCACATGAcagaattaataa<br>gcccgctagatgTCATGTGAcgagcggtgaag<br>ggtgttttgatgTCACATGActgtcaaaacac<br>gtggtcgcgtcgTCACATGAtagatttttctt	5'<br>intron2?<br>intron2?<br>intron2?	>-271<br>>217<br>>476<br>>935
	Anopheles_quadriannulatus	Aqan\Atp6v1B	ND	97	1061	326	67	1074			ND	ttcgtttcttcaTCACATGAcagaattaataa<br>gcccgcaagatgTCATGTGAcgagcggtgaag<br>ggtgttttgatgTCACATGActgtcaaaacac<br>gtggtcgcgtcgTCACATGAtagatttttctt	5'<br>intron2?<br>intron2?<br>intron2?	>-264<br>>218<br>>572<br>>944
	Anopheles_christyi	Achr\Atp6v1B	ND	97	998	326	62	1074			ND	ttgctttcttcaTCACATGAcagaatccataa<br>gtctgtgcgatgTCATGTGAcgagcggtgaag<br>agtgttttgatgTCACATGActgtcaaaacac<br>taagccctctcgTCACATGAtgcctctcgctc	5'<br>intron2?<br>intron2?<br>3'UTR?	>-270<br>>216<br>>569<br>>2567
	Anopheles_epiroticus	Aepi\Atp6v1B	ND	97	977	326	70	1074			ND	tacctatcttcaTCACATGAcagaatccgtaa<br>cgtgagcgggagTCATGTGAcgtgcggtgaag<br>agtgttttgatgTCACATGActgtcaaaacac	5'<br>intron2?<br>intron2?	>-244<br>>221<br>>582
	Anopheles_minimus	Amin\Atp6v1B	ND	97	995	326	60	1074			ND	tgcttttcttcaTCACATGAcagaatccattg<br>ttgtgagatgtgTCATGTGAcggtgaagtgat<br>actgttttgatgTCACATGActgtcaaaacac	5'<br>intron2?<br>intron2?	>-245<br>>218<br>>572
	Anopheles_funestus	Afun\Atp6v1B	ND	97	1007	326	69	1074			ND	tgcttttcttcaTCACATGAcagaatccataa<br>gtgtgagatgtgTCATGTGAcggtgaagtgat<br>actgttttgatgTCACATGActgtcaaaacac	5'<br>intron2?<br>intron2?	>-238<br>>217<br>>582
	Anopheles_stephensi	Aste\Atp6v1B	ND	97	972	326	88	1074			ND	tgcttctcttcaTCACATGAcagaattgctaa<br>ccgtgagatgtgTCATGTGAtatggtatagtg<br>actgttttgatgTCACATGActgtcaaaacac	5'<br>intron2?<br>intron2?	>-250<br>>219<br>>567
	Anopheles_dirus	Adir\Atp6v1B	ND	97	1016	326	67	1074			ND	gcccgtggtacgTCATGTGAcggtgtgttgaa<br>actgttttgatgTCACATGActgtcaaaacac	intron1	>213<br>>556
	Anopheles_nili	Anil\Atp6v1B	ND	97	940	326	83	1074			ND	cgtttctcttcaTCACATGAcagttggcaaaa<br>aagatgtcgtcgTCATGTGAcgatggtgaagt<br>cctgttttgatgTCACATGActgtcaaaacac	5'<br>intron2?<br>intron2?	>-264<br>>192<br>>527
	Anopheles_albimanus	Aalb\Atp6v1B	ND	97	906	326	91	1074			ND	tctgttttgatgTCACATGActgtttggtgtc	intron2?	>496
	Anopheles_darlingi	Adar\Atp6v1B	ND	97	863	326	79	1074			ND	tctgttttgatgTCACATGActgtttggtgtc	intron2?	>473
	Aedes_aegypti	Aaeg\Atp6v1B<br>AAEL005798	ND	91	4094	326	63	1074			ND	ttcatgtttgttTCACATGAcaacctaatgtt<br>agctcagaagtgTCATGTGAtaaccttccaaa<br>aatttgtaaaatTCATATGAggaataagcctt<br>tctgttttgatgTCACATGActgtcaaaacac	5'<br>intron1<br>intron1<br>intron1	>-619<br>>198<br>>292<br>>572
	Culex_pipiens_qui.	Cqui\Atp6v1B<br>CPIJ003418	ND	79	ND	326	65	1074			ND	actagcgagctgTCACATGAgagggtggtgaa<br>ttttcggatgtgTCATGTGAtaacctttattt<br>tctgttttgatgTCACATGAcaccactgtcaa	5'<br>intron1<br>intron1	>-322<br>>179<br>>531
Diptera (Cecidomyiidae)	*Mayetiola_destructor*	Mdes\Atp6v1B	ND	79	737	1133 (119+130+132+125+231+103+155+138)	92	129	116	138	ND	taataccaaaagTCACATGAcagcacgtgagc<br>aacaatgacatgTCATGTGAcctaataatatg<br>tgacttcaataaTCATATGAaaaataagcaat	5'<br>intron1<br>intron1	>-177<br>>608<br>>644

Lepidoptera	*Bombyx_mori*	Bmor\Atp6v1B	ND	85	1803	119	569	130	628	132	576	125	490	231	1021	103	361	155	602	138	335	129	684	138	ND	cettgtgatgggTCACGTGAcagcacgtgaag<br>acttgtgatcagTCACATGAatttggttagtc	5UTR?<br>intron1	>-110<br>>338
	Manduca_sexta	Msex\Atp6v1B	ND	85	ND	119	ND	130	108	132	152	125	198	231	325	103	306	155	259	138	922?	129	295	138	ND	ccttgtgatgggTCACGTGAcggcacgtgaag<br>tatgtgtttataTCACATGAtgaattttatct	5UTR?<br>intron1	>-111<br>>923
	Danaus_plexippus	Dple\Atp6v1B	ND	85	1033	119	229	130	788	132	648	125	498	231	161	103	351	155	519	138	303	129	162	138	ND	tcttgtgatagaTCACGTGAcagcacgtgaag<br>atttaacaaataTCACATGAtcaaattattta	5UTR?<br>intron1	>-106<br>>590
	Heliconius_melpomene	Hmel\Atp6v1B	ND	85	960	119	1102	130	371	132	319	125	386	231 (86+145)	278	103	597	155	371	138	381	129	379	138	ND	tcttgtgataggTCACGTGAcagcacgtgaag<br>ttcttattaggaTCACATGActtctcttctac	5UTR?<br>intron1	>-107<br>>665
	Plutella_xylostella	Pxyl\Atp6v1B	ND	94	1790	119	1433	130	658	132	529	125	287	86 359 145	328	103	3466	155	1259	138	562	129	475	138	ND	ccttgtgacgggTCACGTGAcagcacgtgaag<br>atgtccttgaaaTCATATGActtagtagttac	5UTR?<br>intron1	>-102<br>>1211
Strepsiptera	*Mengenilla_moldrzyki*	Mmol\Atp6v1B	ND	73	69	381 (119+130+132)					1152	752 (125+231+103+155+138)									2681	267 (129+138)			ND	ND		
Coleoptera	*Tribolium_castaneum*	Tcas\Atp6v1B<br>LOC656208	23	91	300	288			51	506 (304+202)					2342	339 (196+143)					49	267			572	cataattcgccgTCACGTGAcgacacgtgaat<br>acacaaaacgggTCATATGAccaaaaattttc<br>ctgtttgggttgTCATGTGAccgaatctcatg	5'UTR?<br>intron1<br>intron1	>-45<br>207<br>351
	Dendroctonus_ponderosae	Dpon\Atp6v1B	138	79	219	288			311	304			61	202	55	196			59	143	355	267			257	attgatttagcaTCACATGAtaaacgtcagtt<br>gtcagttgcgtgTCACATGAgacaaaacttga<br>ggcaaatccatgTCATGTGAcatcccttatga	5'<br>5'UTR<br>intron1	-13<br>6<br>371
Hymenoptera	*Apis_mellifera*	Amel\Atp6v1B<br>GB19171	61	88	565	467							109	666							81	267			468	acttagtataagCCACGTGAtcgcacgtgcca<br>gatttaccatagTCAGCTGAttctgaatgtat<br>tagagacatccaTCATGTGActggatgcagca	5'<br>intron1<br>intron1	-267<br>266<br>421
	Apis_florea	Aflo\Atp6v1B	ND	82	590	467							125	666							86	267			ND	acttagtataaaCCACGTGAttgcacgtgcca<br>gatttatcgtagTCAGCTGAttttgaatgtgt<br>tagagacatctaTCATGTGActagatgcagca	5'<br>intron1<br>intron1	>-328<br>>199<br>>349
	Bombus_terrestris	Bter\Atp6v1B	ND	88	449	467							97	666							74	267			ND	acttagtataagCCACGTGAtcgcacgtgcca<br>atttgccagtagTCAGCTGAttctgaatatgt<br>tcgagatatgtaTCATGTGActatacatacaa	5'<br>intron1<br>intron1	>-320<br>>193<br>>343
	Bombus_impatiens	Bimp\Atp6v1B	ND	88	449	467							103	666							74	267			ND	acttagtataagCCACGTGAtcgcacgtgcca<br>atttgccagtagTCAGCTGAttctgaatatgt<br>tcgagatgtgtaTCATGTGActatacatgcaa	5'<br>intron1<br>intron1	>-320<br>>193<br>>343
	Megachile_rotundata	Mrot\Atp6v1B	ND	88	553	467							64	666							75	267			ND	tcttagtacaagCCACGTGAtcgcacgtgcca<br>atttgcaactaaTCAGCTGAttcaaagggtag<br>atgataaatctgTCATGTGActgtagacagag	5'<br>intron1<br>intron1	>-320<br>>191<br>>253
	Acromyrmex_echinatior	Aech\Atp6v1B	ND	88	865	467							180	666							152	267			ND	acctagtccgggCCACGTGAttgcacgtgcct<br>cccaacttggagTCAGCTGAtcttacttgaaa<br>gagctgccagcgTCATGTGAccatgagtgaca	5'<br>intron1<br>intron1	>-518<br>>187<br>>376
	Atta_cephalotes	Acep\Atp6v1B	ND	88	831	467							202	666							154	267			ND	acctagtccgagCCACGTGAttgcacgtgcct<br>ccgaacttggagTCAGCTGAtctcacttgaaa<br>ctcgagccagcaTCATGTGAtcatgagcaact	5'<br>intron1<br>intron1	>-390<br>>184<br>>365
	Solenopsis_invicta	Sinv\Atp6v1B	ND	88	1438	467							133	666							149	267			ND	cacgttgtccggCCACGTGAttgcacgtgcct<br>gattccctcgggTCAGCTGAtctcacctgaga<br>ctgcttcgagcgTCATGTGActgcggacgaca	5'<br>intron1<br>intron1	>-353<br>>192<br>>371
	Camponotus_floridanus	Cflo\Atp6v1B	ND	88	978	467							130	666							135	267			ND	acgtagtccaagCCACGTGAttgcacgtgcct<br>cccctgactgcaTCAGCTGAtccccgaaattc<br>agaccgagcttgTCATGTGActgcagacgaca	5'<br>intron1<br>intron1	>-363<br>>82<br>>338
	Harpegnathos_saltator	Hsal\Atp6v1B	ND	88	925	467							314	666							244	267			ND	acctagtccgagCCACGTGAttgcacgtgcct<br>tcctcaattgggTCAGCTGAtccccgaaattc<br>gagctcgcggcgTCATGTGActacggacgatt	5'<br>intron1<br>intron1	>-401<br>>182<br>>343
	Pogonomyrmex_barbatus	Pbar\Atp6v1B	ND	88	946	467							108	666							146	267			ND	acctagtccgagCCACGTGAttgcacgtgcct<br>cgacgcaggggg TCAGCTGAttccacttgaaa<br>gagccgccagcaTCATGTGActgcggacgact	5'<br>intron1<br>intron1	>-398<br>>191<br>>359
	Linepithema_humile	LhumAtp6v1B	ND	88	ND	467							115	666 (327+339)							130	267			ND	acttagttcgagCCACGTGAttgcacgtgcct<br>ccaccaaatgggTCAGCTGAtccccgaaattc<br>gagctgccagcgTCATGTGActgcagacgacg<br>gaaatatgaataTCACATGAtttagttagtag	5'<br>intron1<br>intron1<br>intron1	>-369<br>>170<br>>332<br>>579
	Nasonia_vitripennis	Nvit\Atp6v1B	ND	88	1138	467							75	327	79	339					110	267			ND	catcagcccgagCCACGTGAtcccacgtgctt<br>cccctcagctggTCAGCTGAtcggcgcttgcc<br>gtaggtcttgtgTCATGTGActgcagacgacg<br>cactcgtgggagTCATGTGAcacgctgccgtt	5'<br>intron1<br>intron1<br>intron1	>-417<br>>379<br>>543<br>>722
	Nasonia_giraulti	Ngir\Atp6v1B	ND	88	ND	467							74	327	78	339					110	267			ND	catcagcccgagCCACGTGAtcccacgtgctt<br>tccctcagctggTCAGCTGAtcggcgcttgcc<br>gtaggtcttgtgTCATGTGActgcagacgacg<br>cactcgtgggagTCATGTGAcacgctgccgtt	5'<br>intron1<br>intron1<br>intron1	>-426<br>>381<br>>546<br>>723
	Nasonia_longicornis	Nlon\Atp6v1B	ND	88	1172	467 (155+312)							ND	327 (102+98+124)	78	339 (151+83+105)					110	267			ND	catcagcccgagCCACGTGAtcccacgtgctt<br>tccctcagctggTCAGCTGAtcggcgcttgcc<br>gtaggtcttgtgTCATGTGActgcagacgacg<br>cactcgtgggagTCATGTGAcacgctgccgtt	5'<br>intron1<br>intron1<br>intron1	>-420<br>>378<br>>542<br>>719

Hemiptera	*Acyrthosiphon_pisum*	LOC100572714		88	603	155			70	312 (172+140)							72	147			521	331					69	188			74	130	71	140			ND		
	Rhodnius_prolixus	Rpro\Atp6v1B		79	205	155			73	172			1383	140			118	147			846	331					861	188			3191	130	87	149			ND		
Phthiraptera	*Pediculus_humanus*	PHUM253140		79	415	155			135	172			103	287 (140+147)							81	414 (331+83)							83	235 (105+130)			91	149			tacggtggcttgTCATGTGAttTCACGTGAtc	5'UTR?	>-32
Odonata	*Ladona_fulva*	Lful\Atp6v1B		88	323	155			101	172			1065	140			82	147			156	180+191+151					89	188			264	130	127	149			tcgagtagctggTCACGTGAatTCACGTGAttgacatcgaaa<br>attttatttcatTCATGTGAtccacccaccca<br>tgaatttaggagTCACATGAagttatgatcca	5'<br>intron<br>intron	>-23/-13<br>>1569<br>>3413
**Crustacea**	*Daphnia_pulex*	Dpul\Atp6v1B		94	86	155(56+99)			57	172			64	140			67	478(105+98+124+151)									78	188 (83+105)			63	273					gtgatctgacagTCATATGAtcgagagaaccc	5'	>-138
Ixodida	*Ixodes_scapularis*	ISCW022132	99	85	1630	56	944	99	80	94	57	78	67	140			74	105	820	98	903	358 (124+151+83)							2083	105	93	130	ND	155			tggtggtagcggTCATGTGActcaggatgtca	intron1	547
	Homo_sapiens	ATP6V1B1	87	118	7585	56	14332	99	188	94	591	78	843	140			842	102	573	98	1083	124	261	151			259	83	783	105	193	130	92	164	310		atcctccagctcTCAGGTGAcgggggcccctc	5'	>-149
		ATP6V1B2	214	135	6941	56	4893	99	814	94	129	78	530	140(82+58)			335	102	348	98	582	124	1912	151			1444	83	724	105	828	130	1980	140	1294		tcgaaatggataTCAGCTGAcgttggcctgca	5'	>-121
Cnidaria	*Nematostella_vectensis*	Nvec\Atp6v1B	ND	79	152	56	394	99	295	94	174	78	405	82	87	58	96	102	152	98	115	124	199	81	447	70	180	83	334	105	577	130	329	137			ccgttcagggctTCACGTGAtctcttcgtgag	intron1	>171
Placozoa	*Trichoplax_adhaerens*	Tadh\Atp6v1B	ND	73	213	56	740	99	344	94	101	160 (78+82)			73	58	85	102	158	98	99	124	72	81	70	70	110	83	79	105	118	130	92	149			cttgaatgactaTCACATGAccatgcgtatac	5'	>-290
