# Supplementary material for: Structural and Comparative Analyses of Insects Suggest the Presence of an Ultra-Conserved Regulatory Element of the Genes Encoding Vacuolar-Type ATPase Subunits and Assembly Factors
Source: Biology (Basel). 2023 Aug 13;12(8):1127. doi: 10.3390/biology12081127 (PMC10452791; doi:10.3390/biology12081127)
Supplement: Supplementary file 1 [file biology-12-01127-s001.zip › Supplementary Files S20-S37/Supp_File_S22_Atp6v1C_INS.pdf]

Gene Cluster ID	Atp6v1C
**Drosophila melanogaster gene**	**Vha44 (CG8048)**
**Predicted function**	**ATPase, H+ transporting, lysosomal 42kDa, V1 subunit C**

Atp6v1C CLEAR element conservation:

```
              _____________5'_________________    __________5'/intron1__________

    Dmel\  ACACTGGCGCCATCACGTGAATCTCGTGGGCA  ATCGAAAAACTGTCATGTGACTGTCAACGAAC
    Mdom\  CTGTGTCACTTATCACGTGAATTTCTTCTCCT  GATTCAAGCTTGTCATGTGACTGTCATCTGTC
    Ccap\  CTGCGAATGTTATCACGTGAATTTTTGTACAT  GACCCAAAGCTGTCATGTGACTGTCATCAAAC
    Gmor\  --------------------------------  CGATCCAAACTGTCATGTGACTTATCATCAAA
    Llon\  --------------------------------  TAGGAAAAATTTTCATGTGAAATTCATTTCTT
    Ppap\  --------------------------------  AAGATGGTATGTTCATGTGACGTCAAAAAACT

    Agam\  CACCGAAAGCAATCACGTGAATACGTTGTAAT  AAAAGGTAGATGTCATGTGACTTATCAGTTCA
    Aara\  CACCGAAAGCAATCACGTGAATACGTTGTAAT  AAAAGGTAGATGTCATGTGACTTATCAGTTCA
    Aqan\  CACCGAAAGCAATCACGTGAATACGTTGTAAT  AAAAGGTAGATGTCATGTGACTTATCAGTTCA
    Achr\  CTCCGAAAGCAATCACGTGAATACGTTGTAAT  AAAAGGTAGATGTCATGTGACTTATCAGTTCA
    Aepi\  CTCCGAAAGCAATCACGTGAATACGTTGTAAT  AAAAGGTGGATGTCATGTGACTTTTATCAATT
    Amin\  CTCCGAAAGCAATCACGTGAATACTGTAATGG  AAAGGTAGACTGTCATGTGACTTATCAGTTCA
    Afun\  CTCCGAAAGCAATCACGTGAATACTGTAATGG  AAAAGGTAGATGTCATGTGACTTATCAGTTCA
    Aste\  CTCCGAAAGCAATCACGTGAATACTGTGTAAT  AAAAGGTAGATGTCATGTGACTTATCAGTTCA
    Adir\  GCTCGGAAGCAATCACGTGAATACTTGTAATG  AAAAGGTAGATGTCATGTGACTTATCAGTTCA
    Anil\  AGAGGCTTGCAATCACGTGAATAATATGGTAA  AAAAGGTAGATGTCATGTGACTTATCATTTCA
    Aalb\  ACGAGCCGCGAATCACGTGAATTTCTGTGAAG  GAAAAGATAGTGTCATGTGACTTATCACACAA
    Adar\  CTTCTCGTGCAATCACGTGAATCGAATTCGGA  GAAAAGATAGTGTCATGTGACTTATCACACAA
    Aaeg\  ACGCAGAGACTGTCACGTGAATTGCAATGGTG  AGGAAAAATATGTCATGTGACTTATCATTCAA
    Cqui\  ACACTTTGGCTGTCACGTGAATTGCAATGGTG  TTGAAAAATATGTCATGTGACTTATCAGTCAA

    Mdes\  TTATGTGAAATGTCACATGACACAAATGTTAA  CAATGGTAAATGTCATGTGACTCACAACAAAA

    Bmor\  GGCAGCACAGCGTCACGTGAATTTTCATGGCG
    Msex\  GGCAGCACCGCATCACGTGAATTTTCATGGCG
    Dple\  GGCAGCACGGCATCACGTGAATTTTCATGGCG
    Hmel\  GGCAGCACAGCATCACGTGAATTTTCATGGCG
    Pxyl\  GGCAGCACCGCATCACGTGAATTTTCATGGCG

    Tcas\  CAACATGTCAAATCACGTGAAACCCCACTAAG  TGTCAATTGAAGTCACATGATCGCCTTCAATT
    Dpon\  ACAATTTGACAGTCACGTGAAAATAACGATCA  AGTTTAGTGTAGTCATGTGACTGTAGTAATGT

    Amel\  AACTGTTCCAAATCAGCTGATATTCCCTTTGG  ACACAATTGTCATCACGTGAG--GTCACAATG
    Bter\  TCTGGTTCCAAATCAGCCGATGATTCTTTTAG  ACACAATTGTCATCACGTGAG--GTCACAATG
    Bimp\  TCTGGTTCCAAATCAGCTGATGATTCTTTTAG  ACACAATTGTCATCACGTGAG--GTCACAATG
    Aflo\  AACGGTTCCAAATCAGCTGATGATATTCTCTT  ACACAATTGTCATCACGTGAG--GTCACAATG
    Mrot\  GACAATTCCAAGTCAGCTGATGATACTGTGGC  ACACAATTGTCATCACGTGAG--GTCACAATG
    Lhum\  CAGGTTCTTGAGTCAGCTGATAAGTGTTTAAT  ACACAATTGTCATCACGTGAGAAGTCACGATG
    Cflo\  TCGAGTTACGAATCAGCTGACAATATTCAGTT  ACATAATTGTCATCACGTGAGGAGTCACAATG
    Aech\  TCTTGTATGCCGTCAGCTGACGGCACGGCGTT  CGCAATCTGTCATCACGTGAGCGGTCACAATG
    Acep\  TGCGTCGCGGAGTCAGCTGACGGCACGGCGTT  CGCAATCTGTCATCACGTGAGTGGTCACAATG
    Pbar\  TGTCACGCGGAGTCAGCTGACAGCACAGCGTT  CACAATCTGTCATCACGTGAGTGGTCACAATG
    Sinv\  TGTCGCACGGAGTCAGCTGACGGCACGCGGCG  CACAATCTGTCATCACGTGAGCGGTCACAATG
    Hsal\  TCGGGCGCCAAGTCAGCTGACGGCACAGTGTG  ACACAATTGTCATCACGTGAGAGGTCACAATG
    Ngir\  --------------------------------  GCAGCACTCTTATCACGTGAGAGCGTGCGTAT
    Nlon\  --------------------------------  GCAGCACTCTTATCACGTGAGAGCGTGCGTAT
    Nvit\  --------------------------------  GCAGCACTCTTATCACGTGAGAGCGTGCGTAT

    Phum\  GGCAGCACATAATCACGTGAATCGTTATGGTA
    Lful\  GTCACCACCGAGTCACGTGAATTGTAATGAAT
    Dpul\  GGAAACCTTAGATCACATGACAGTCTCCTGAC
```

Atp6v1C gene structure comparison:

Legend: UTR region, CDS region INTRON

Order	Species	Atp6v1C orthologs	5'exon	intron	exon	intron	exon	intron	exon	intron	exon	intron	exon	intron	exon	intron	exon	intron	exon3'	Extended CLEAR region	Position	bps from TSS

Diptera (Drosophilidae)	*Drosophila_melanogaster*	Dmel\Vha44-RA	148	3069	8	135	90	154			5873	187			64	99	62	256			62	98	59	139	70	99	1153	acactggcgccaTCACGTGAatctcgtgggca<br>atcgaaaaactgTCATGTGActgtcaacgaac	5'<br>intron1	-40<br>565
Diptera (Muscidae)	*Musca_domestica*	Mdom\Atp6v1C	ND	ND	11	132	68	154			13312	187			164	99	68	256			71	98	70	145	2264	99	ND	ctgtgtcacttaTCACGTGAatttcttctcct<br>gattcaagcttgTCATGTGActgtcatctgtc	5'<br>intron1?	ND<br>ND
Diptera (Tephritidae)	*Ceratitis_capitata*	Ccap\Atp6v1C	192	14748	11	132	ND	154			18961	187			2027	99	93	256			1441	98	106	142	562	99	ND	ctgcgaatgttaTCACGTGAatttttgtacat<br>gacccaaagctgTCATGTGActgtcatcaaac	5'<br>intron1	-115<br>670
Diptera (Glossinidae)	*Glossina_morsitans*	Gmor\Atp6v1C<br>TMP008641*	138	5966	11	132	72	154			10967	187			1138	99	82	256			77	98	56	139	81	99	239	cgatccaaactgTCATGTGActtatcatcaaa	intron1	531
Diptera (Psychodidae)	*Lutzomyia_longipalpis*	Llon\Atp6v1C			ND	286					ND	187			85	99	1215	493							471	96	ND	taggaaaaatttTCATGTGAaattcatttctt	intron1	ND
	Phlebotomus_papatasi	Ppap\Atp6v1C			ND	286 (132+154)					ND	187			ND	99	2288	493 (256+98+139)							ND	96	ND	aagatggtatgtTCATGTGAcgtcaaaaaact	intron1	ND
Diptera (Culicidae)	*Anopheles_gambiae*	Agam\Atp6v1C<br>AGAP005845	279	869	28	132	102	154			4455	187			74	99	71	256			75	98	72	136	77	96	345	caccgaaagcaaTCACGTGAatacgttgtaat<br>aaaaggtagatgTCATGTGActtatcagttca	5'UTR<br>intron1	25<br>658
	Anopheles_arabiensis	Aara\Atp6v1C			ND	132	101	154			4446	187			76	99	74	256			70	98	72	136	72	96	ND	caccgaaagcaaTCACGTGAatacgttgtaat<br>aaaaggtagatgTCATGTGActtatcagttca	5'UTR?<br>intron1?	>-1160<br>>-518
	Anopheles_quadriannulatus	AqanAtp6v1C			ND	132	101	154			4415	187			80	99	73	256			71	98	72	136	74	96	ND	caccgaaagcaaTCACGTGAatacgttgtaat<br>aaaaggtagatgTCATGTGActtatcagttca	5'UTR?<br>intron1?	>-1155<br>>-514
	Anopheles_christyi	Achr\Atp6v1C			ND	132	85	154			4227	187			81	99	93	256			74	98	75	136	69	96	ND	ctccgaaagcaaTCACGTGAatacgttgtaat<br>aaaaggtagatgTCATGTGActtatcagttca	5'UTR?<br>intron1?	>-1166<br>>-514
	Anopheles_epiroticus	Aepi\Atp6v1C			ND	135	69	154			4993	187			73	99	80	256			67	98	76	136	62	96	ND	ctccgaaagcaaTCACGTGAatacgttgtaat<br>aaaaggtggatgTCATGTGActtttatcaatt	5'UTR?<br>intron1?	>-1135<br>>-504
	Anopheles_minimus	Amin\Atp6v1C			ND	132	84	154			4210	187			65	99	75	256			72	98	69	136	63	96	ND	ctccgaaagcaaTCACGTGAatactgtaatgg<br>aaaggtagactgTCATGTGActtatcagttca	5'UTR?<br>intron1?	>-1137<br>>-485
	Anopheles_funestus	Afun\Atp6v1C			ND	132	93	154			4498	187			73	99	79	256			87	98	74	136	72	96	ND	ctccgaaagcaaTCACGTGAatactgtaatgg<br>aaaaggtagatgTCATGTGActtatcagttca	5'UTR?<br>intron1?	>-1108<br>>-469
	Anopheles_stephensi	Aste\Atp6v1C			ND	132	77	154			ND	187			ND	99	65	256			73	98	90	136	ND	96	ND	ctccgaaagcaaTCACGTGAatactgtgtaat<br>aaaaggtagatgTCATGTGActtatcagttca	5'UTR?<br>intron1?	ND
	Anopheles_dirus	Adir\Atp6v1C			ND	132	68	154			3912	187			68	99	73	256			57	98	347	136	71	96	ND	gctcggaagcaaTCACGTGAatacttgtaatg<br>aaaaggtagatgTCATGTGActtatcagttca	5'UTR?<br>intron1?	>-1111<br>>-478
	Anopheles_nili	Anil\Atp6v1C			ND	132	78	154			ND	187			86	99	69	256			66	98	73	136	67	96		agaggcttgcaaTCACGTGAataatatggtaa<br>aaaaggtagatgTCATGTGActtatcatttca	5'UTR?<br>intron1?	>-1108<br>>-500
	Anopheles_albimanus	Aalb\Atp6v1C			ND	132	79	154			3054	187			76	99	75	256			67	98	76	136	77	96	ND	acgagccgcgaaTCACGTGAatttctgtgaag<br>gaaaagatagtgTCATGTGActtatcacacaa	5'UTR?<br>intron1?	>-923<br>>-337
	Anopheles_darlingi	Adar\Atp6v1C			ND	132	78	154			3189	187			73	99	70	256			73	98	71	136	77	96	ND	cttctcgtgcaaTCACGTGAatcgaattcgga<br>gaaaagatagtgTCATGTGActtatcacacaa	5'UTR?<br>intron1?	>-946<br>>-367
	Aedes_aegypti	Aaeg\Atp6v1C<br>AAEL005173	215	7535	60	132	65	154			9933	187			56	99	55	256			62	98	66	136	10450	96	593	acgcagagactgTCACGTGAattgcaatggtg<br>aggaaaaatatgTCATGTGActtatcattcaa	5'UTR<br>intron1	7<br>632
	Culex_pipiens_qui.	Cqui\Atp6v1C<br>CPIJ002067			58	132	55	154			2686	187			63	99	220	256 (124+132)			56	98	64	136	64	96	ND	acactttggctgTCACGTGAattgcaatggtg<br>ttgaaaaatatgTCATGTGActtatcagtcaa	5'UTR?<br>intron1?	ND<br>ND
Diptera (Cecidomyiidae)	*Mayetiola_destructor*	Mdes\Atp6v1C	121	3731	186	132	ND	154			14525	187			100	99	76	124	96	132	173	98	128	136	721	96	ND	ttatgtgaaatgTCACATGAcacaaatgttaa<br>caatggtaaatgTCATGTGActcacaacaaaa	5'UTR?<br>intron1	-66<br>949
Lepidoptera	*Bombyx_mori*	Bmor\Atp6v1C			250	129	1316	102	744	52	7172	98	1143	89	556	99	1370	124	853	132	1681	98	1369	139	940	99	31	ggcagcacagcgTCACGTGAattttcatggcg<br>atctgtaacctcTCACATGAgaagaacgtgtg	5'<br>intron1	-286<br>1267
	Manduca_sexta	Msex\Atp6v1C			ND	129	1581	102	328	52	ND	98	266	89	267	99	99	124	ND	132	321	98	250	139	505	96	ND	ggcagcaccgcaTCACGTGAattttcatggcg	5'	>-541
	Danaus_plexippus	Dple\Atp6v1C			ND	129	996	102	427	52	3233	98	346	89	531	99	415	124	339	132	286	98	586	139	251	93	ND	ggcagcacggcaTCACGTGAattttcatggcg	5'	>-537
	Heliconius_melpomene	Hmel\Atp6v1C			ND	129	853	102	481	52	5651	98	874	89	240	99	120	124	837	132	321	98	425	139	1281	96	ND	ggcagcacagcaTCACGTGAattttcatggcg	5'	>-544
	Plutella_xylostella	Pxyl\Atp6v1C			ND	129	414	102	430	52	6248	98	363	89	513	99	508	124	711	132	596	98	693	136	721	96	ND	ggcagcaccgcaTCACGTGAattttcatggcg	5'	>-568
Strepsiptera	*Mengenilla_moldrzyki*	Mmol\Atp6v1C			ND	132	ND	154			1478	187			ND	99	59	256			994	240			52	96	ND	ND		
Coleoptera	*Tribolium_castaneum*	Tcas\Atp6v1C<br>LOC661302	81	414	33	132	46	154			2617	187			50	355 (99+124+132)					54	98	75	136	49	99	572	caacatgtcaaaTCACGTGAaaccccactaag<br>tgtcaattgaagTCACATGAtcgccttcaatt	5'UTR?<br>intron1	1<br>418
	Dendroctonus_ponderosae	Dpon\Atp6v1C	78	601	46	132	871	154			ND	187			84	99	65	124	69	132	588	98	61	136	513	99	182	acaatttgacagTCACGTGAaaataacgatca<br>agtttagtgtagTCATGTGActgtagtaatgt	5'UTR?<br>intron1	-5<br>497
Hymenoptera	*Apis_mellifera*	Amel\Atp6v1C	170	1039	36	286					6348	187			120	99	71	209			149	145	108	145	105	96	ND	aactgttccaaaTCAGCTGAtattccctttgg<br>acacaattgtcaTCACGTGAggtcacaatggt	5'<br>5'UTR	-22<br>42

		GB11380*																																
	Apis_florea	Aflo\Atp6v1C	170	1094	36	286							ND	187			121	99	74	209					149	145?	86	145	122	96?	ND	aacggttccaaaTCAGCTGAtgatattctctt<br>acacaattgtcaTCACGTGAggtcacaatggt	5'<br>5'UTR	-26<br>42
	Bombus_terrestris	Bter\Atp6v1C<br>LOC100644844*	118	980	36	286							5976	187			113	99	76	209					150	145	67	145	114	96	ND	tctggttccaaaTCAGCCGAtgattcttttag<br>acacaattgtcaTCACGTGAggtcacaatggt	5'<br>5'	-80<br>-9
	Bombus_impatiens	Bimp\Atp6v1C<br>LOC100741901*	118	983	36	286							5985	187			113	99	76	209					150	145	67	145	100	96	ND	tctggttccaaaTCAGCTGAtgattcttttag<br>acacaattgtcaTCACGTGAggtcacaatggt	5'<br>5'	-79<br>-8
	Megachile_rotundata	Mrot\Atp6v1C			ND	286							6046	187			137	99	81	209					82	145	71	139	143	96	ND	gacaattccaagTCAGCTGAtgatactgtggc<br>acacaattgtcaTCACGTGAggtcacaatggt	5'<br>5'	>-1550<br>>-1475
	Acromyrmex_echinatior	Aech\Atp6v1C			ND	286							6067	187			81	99	116	209					159	145	93	139	352	96	ND	tcttgtatgccgTCAGCTGAcggcacggcgtt<br>cgcaatctgtcaTCACGTGAgcggtcacaatg	5'<br>5'	>-1408<br>>-1320
	Atta_cephalotes	Acep\Atp6v1C			ND	286							ND	187			77	99	114	209					145	145	154	139	227	96	ND	tgcgtcgcggagTCAGCTGAcggcacggcgtt<br>cgcaatctgtcaTCACGTGAgtggtcacaatg	5'<br>5'	>-1418<br>>-1337
	Solenopsis_invicta	Sinv\Atp6v1C			ND	286							ND	187			86	99	124	209					ND	145	83	139	457	96	ND	tgtcgcacggagTCAGCTGAcggcacgcggcg<br>cacaatctgtcaTCACGTGAgcggtcacaatg	5'<br>5'	>-1710<br>>-1608
	Camponotus_floridanus	Cflo\Atp6v1C			ND	286							14599	187			82	99	133	209					108	145	532	142	180	96	ND	tcgagttacgaaTCAGCTGAcaatattcagtt<br>acataattgtcaTCACGTGAggagtcacaatg	5'<br>5'	ND<br>ND
	Harpegnathos_saltator	Hsal\Atp6v1C			ND	286							5669	187			78	99	91	209					179	145	78	145	131	96	ND	tcgggcgccaagTCAGCTGAcggcacagtgtg<br>acacaattgtcaTCACGTGAgaggtcacaatg	5'<br>5'	>-1186<br>>-1110
	Pogonomyrmex_barbatus	Pbar\Atp6v1C			ND	286							5825	187			96	99	128	209					212	145	71	139	233	96	ND	tgtcacgcggagTCAGCTGAcagcacagcgtt<br>cacaatctgtcaTCACGTGAgtggtcacaatg	5'<br>5'	>-1866<br>>-1786
	Linepithema_humile	LhumAtp6v1C			ND	286							5806	187			91	99	117	209					211	145	83	145	186	96	ND	caggttcttgagTCAGCTGAtaagtgtttaat<br>acacaattgtcaTCACGTGAgaagtcacgatg	5'<br>5'	>-1331<br>>-1261
	Nasonia_vitripennis	Nvit\Atp6v1C	343	1105	47	286							6167	187			86	99	73	209					70	290			80	96	ND	gcagcactcttaTCACGTGAgagcgtgcgtat	5'UTR	43
	Nasonia_giraulti	Ngir\Atp6v1C			ND	286							ND	187			87	99	73	209					70	290			80	96	ND	gcagcactcttaTCACGTGAgagcgtgcgtat	5'UTR?	ND
	Nasonia_longicornis	Nlon\Atp6v1C			ND	286							ND	187			87	99	73	209					70	290			80	96	ND	gcagcactcttaTCACGTGAgagcgtgcgtat	5'UTR?	ND
Hemiptera	Acyrthosiphon_pisum	Apis\Atp6v1C<br>ACYPI006545			173	286							5645	286 (187+99)					69	162			670	94	67	98	52	142	59	96	255	ND		
	Rhodnius_prolixus	Rpro\Atp6v1C			ND	132	1627	154					1996	187			104	99	1442	162			ND	94	370	98	80	148	123	96		ND		
Phthiraptera	Pediculus_humanus	Phum\Atp6v1C<br>PHUM039540*			ND	132	176	154					1320	187			92	99	70	162			105	94	87	234			95	96		ggcagcacataaTCACGTGAatcgttatggta	5'	>-571
Odonata	Ladona_fulva	Lful\Atp6v1C			ND	132	81	154					127	187			389	99	499	162			242	94	93	98	73	133	271	96		gtcaccaccgagTCACGTGAattgtaatgaat	5'	>-235
**Crustacea**	Daphnia_pulex	Dpul\Atp6v1C			84	286							68	187			72	99	60	162(69+93)			64	94	61	98	62	130	72	102	243	ggaaaccttagaTCACATGAcagtctcctgac	5'	-24
Aracnida	Ixodes_scapularis	Isca\Atp6v1C<br>ISCW009268	103	383	19	135	61	68	102	86			542	99	1243	88	3230	99	76	69	1436	93	1102	94	2152	98	410	130	673	96	166	ND		
	Homo_sapiens	ATP6V1C1	206	19572	39	132	1371	68	6462	86			2094	95	1586	92	1061	99	1885	69	7018	93	87	94	1485	98	1509	127	2204	96		cgcgcgccgcggTCAGCTGActgctgggctgg	5'UTR	34
Cnidaria	Nematostella_vectensis	Nvec\Atp6v1C<br>v1g160000			ND	123	362	68	187	52	463	34	136	95	1017	92	701	99	81	69	236	93	819	94	219	77+123+24	77	40+147+93	545	93		ND		
Placozoa	Trichoplax_adhaerens	Tadh\Atp6v1C<br>TRIADDRAFT_19844			ND	141	1245	120			325	34	97	95	155	92	132	99	72	69	100	93	81	94	69	66+98+32	71	40+76+87	80	96		ND		
