# Supplementary material for: Structural and Comparative Analyses of Insects Suggest the Presence of an Ultra-Conserved Regulatory Element of the Genes Encoding Vacuolar-Type ATPase Subunits and Assembly Factors
Source: Biology (Basel). 2023 Aug 13;12(8):1127. doi: 10.3390/biology12081127 (PMC10452791; doi:10.3390/biology12081127)
Supplement: Supplementary file 1 [file biology-12-01127-s001.zip › Supplementary Files S20-S37/Supp_File_S23_Atp6v1D_INS.pdf]

| Gene Cluster ID | Atp6v1D |
|---|---|
| **Drosophila melanogaster gene** | **Vha36-1 (CG8186)** |
| FlyBase ID | FBgn0022097 |
| **Predicted function** | **ATPase, H+ transporting, V1 subunit D** |

**Atp6v1D CLEAR element conservation:**

```
                   ______________5'/5'UTR_____________   _________ ___intron1_____________
Mdom\Atp6v1D       AAGACCCAATAATCACATATGACCGTATTGTAGA
Ccap\Atp6v1D-1     AGCTTTTGTTGGTCATATGACATTTCTGCTGA
Ccap\Atp6v1D-1     TGCTGACATTTGTCAGCTGACCCAACGACGAC

Gmor\Atp6v1D-1     TCACAAGACAAGTCAGCTGACCTCGAAAGAAA     --------------------------------
Llon\Atp6v1D       GAACATCACAAGTCAGCTGACTTTTCTGGTAA     GGAAAAGCAGGGTCATGTGAGGGATTTCAATA
Ppap\Atp6v1D       TTGCAGTTCAAGTCAGCTGACTTTCTGTGGCA     TGGGAATCAGTGTCATGTGAGTATCTTGTTTA

Agam\Atp6v1D       GGTATGATATGGTCTTGTGACCAGAGTAATCT
Aara\Atp6v1D       GGTATGATATGGTCTTGTGACCAGAGTAATCT
Aqan\Atp6v1D       GGTATGATATGGTCTTGTGACCAGAGTAATCT
Achr\Atp6v1D       GTTGTTATATGGTCTTGTGACCAGAGCAATCT
Aepi\Atp6v1D       GTTGTTACATGGTCTTGTGATCAGTACAATCT
Amin\Atp6v1D       GTGGTTATATGGTCTTGTGATCAGTGGAACCT
Afun\Atp6v1D       GTTGTTGTATGGTCTTGTGACCAGAGAATCTG
Aste\Atp6v1D       GTTGTTATGTGGTCTTGTGACCAGAAGAGCCT
Adir\Atp6v1D       TGTGTTCTGTGGTCTTGTGACCATAATTTGCG
Anil\Atp6v1D       GTGTGTAACGAGTCTTGTGACCATAATTTGCG
Aalb\Atp6v1D       ATCACTGTAGGGTCTTGTGATCATCGGGTTTG
Adar\Atp6v1D       CATCACTGTAGGTCTTGTGACTGGGTTTGCTG
Aaeg\Atp6v1D       AAATTCATTTGATCTTGTGACCCGTTTTGCGC
Cqui\Atp6v1D       GTCTCGATTTGATCTTGTGACTTGAATTTTGG
Mdes\Atp6v1D       GCTGACTACTTCTCTTGTGACCATACACAGAA

Aaeg\Atp6v1D       GATCCATCAAAATCACATGACATGTGTCAAAC
Cqui\Atp6v1D       TCACACAGTTCGTCATGTGACATTTTGCTGCC
Mdes\Atp6v1D       TTTTCTTTAGTGTCAGCTGACTACTTCTCTTG

Bmor\Atp6v1D       TGTCTTCTGTCATCAGGTGATTTGGGTTTGGG
Msex\Atp6v1D-1     TGTCTGCTGTCATCAGGTGATTTAGGTTTTGG
Dple\Atp6v1D-1     TGTCTTCTGTCATCAGGTGATCGAGAATTAGG
Hmel\Atp6v1D-1     TGTCTTCTGTCATCAGGTGATTGAAATTGGGA
Pxyl\Atp6v1D       TGTCTTCTGTCATCAGGTGATCGTGAGCAAAA

Tcas\Atp6v1D       TATGTCACGCGGTCAGCTGACTGATCTCGTGA
Dpon\Atp6v1D       CTGATTTTCATGTCAGCTGACTAGTCTTGTGA

Amel\Atp6v1D       CGGTCTTACGAGTCAGCTGATCAATTTCATTC
Aflo\Atp6v1D       CGGTCTTACGAGTCAGCTGATCAATTTCATTC
Bter\Atp6v1D       CGGTCCTACGAGTCAGCTGATCAGTTTCATTC
Bimp\Atp6v1D       CGGTCCTACGAGTCAGCTGATCAGTTTCATTC
Mrot\Atp6v1D       CGGTCCCACAAGTCAGCTGATCAATCTTATTC
Aech\Atp6v1D       CGGTCTCTCGTGTCAGCTGATCAATTCCATTC
Acep\Atp6v1D       CGGTCTCTCATGTCAGCTGATCAATCCCGTTC
Lhum\Atp6v1D       CGGTCTCTCGTGTCAGCTGATCAATCCCGTTC
Sinv\Atp6v1D       CGGTCTCTCACGTCAGCTGATCAATCCCATTC
Pbar\Atp6v1D       --------------------------------
Hsal\Atp6v1D       CGGTCGCCCGAGTCAGCTGATCAATCCCATTC
Cflo\Atp6v1D       GGTCAGCTCGAGTCAGCTGATCAATCCCGTTC
```

```
Nvit\Atp6v1D    TTTCCGTCAAAGTCAGCTGATCAATCGTATTC
Ngir\Atp6v1D    TTTCCGTCAAAGTCAGCTGATCAATCGTATTC
Nlon\Atp6v1D    TTTCCGTCAAAGTCAGCTGATCAATCGTATTC

Rpro\Atp6v1D    tacacattataaTCAGCTGAtgtaccttccct
Phum\Atp6v1D    tggaaataatagTCAGCTGActttagctctga
Lful\Atp6v1D    tacctaattgtgTCAGCTGActttacgcctac
Dpul\Atp6v1D    cttctcttgttgTCACATGAtcagatgattct
```

## Atp6v1D gene structure comparison:

Key: UTR region, CDS region INTRON

| Order | Species | Atp6v1D orthologs | 5'exon | | intron | exon | intron | exon | intron | exon | intron | exon | intron | exon3' | | Extended CLEAR region | Position | bps from TSS |
|---|---|---|---|---|---|---|---|---|---|---|---|---|---|---|---|---|---|---|
| Diptera (Drosophilidae) | *Drosophila_melanogaster* | Dmel\Vha36-1 | 120 | 741 | | | | | | | | | | | 221 | ttgcaaaaagggTCATCTGActtgttttatac<br>acaattgcacagTCACATGAcagcaaacatat | 3'<br>3' | 1142<br>1193 |
| | | Dmel\Vha36-2 | 73 | 525 | | | | | 75 | 597 | | | | | 101 | ND | | |
| | | Dmel\Vha36-3 | 83 | 41 | 60 | 118 | 55 | 364 | | | 61 | 227 | | | 45 | ND | | |
| Diptera (Muscidae) | *Musca_domestica* | Mdom\Atp6v1D-1 | | 41 | 110 | 118 | 96 | 364 | | | 67 | 218 | | | | aagacccaataaTCACATATGAccgtattgtaga | 5' | >-125 |
| | | Mdom\Atp6v1D-2 | | 969 | | | | | | | | | | | | ND | | |
| Diptera (Tephritidae) | *Ceratitis_capitata* | Ccap\Atp6v1D-1 | | 41 | 132 | 118 | 132 | 364 | | | 58 | 218 | | | | agcttttgttggTCATATGAcatttctgctga<br>tgctgacatttgTCAGCTGAcccaacgacgac | 5'<br>5' | >-128<br>>-102 |
| | | Ccap\Atp6v1D-2 | | 951 | | | | | | | | | | | | ND | | |
| Diptera (Glossinidae) | *Glossina_morsitans* | Gmor\Atp6v1D-1 TMP011652 | 124 | 41 | 73 | 118 | 69 | 364 | | | 3174 | 218 (95+123) | | | 56 | tcacaagacaagTCAGCTGAcctcgaaagaaa | 5'UTR | 3 |
| | | Gmor\Atp6v1D-2 TMP010970 | | 1062 | | | | | | | | | | | | ND | | |
| Diptera (Psychodidae) | *Lutzomyia_longipalpis* | Llon\Atp6v1D | | 41 | 123 | 700 (118+364+95+123) | | | | | | | | | | gaacatcacaagTCAGCTGActtttctggtaa<br>ggaaaagcagggTCATGTGAgggatttcaata | 5'<br>intron1 | >-47<br>>138 |
| | *Phlebotomus_papatasi* | Ppap\Atp6v1D | | 41 | 135 | 459 | | | | 63 | 241 | | | | | ttgcagttcaagTCAGCTGActttctgtggca<br>tgggaatcagtgTCATGTGAgtatcttgttta | 5'<br>intron1 | >-48<br>>147 |
| | | | | | | | | | | | | | | | | | | |
| Diptera (Culicidae) | *Anopheles_gambiae* | Agam\Atp6v1D AGAP010298 | 224 | 41 | 83 | 118 | 75 | 364 | | | 67 | 95 | 82 | 123 | 250 | ggtatgatatggTCTTGTGAccagagtaatct | 5'UTR | 108 |
| | *Anopheles_arabiensis* | Aara\Atp6v1D | | 41 | 83 | 118 | 75 | 364 | | | 67 | 95 | 82 | 123 | | ggtatgatatggTCTTGTGAccagagtaatct | 5' | >-110 |
| | *Anopheles_quadriannulatus* | Aqan\Atp6v1D | | 41 | 83 | 118 | 75 | 364 | | | 67 | 95 | 82 | 123 | | ggtatgatatggTCTTGTGAccagagtaatct | 5' | >-110 |
| | *Anopheles_christyi* | Achr\Atp6v1D | | 41 | 86 | 118 | 77 | 364 | | | 72 | 95 | 82 | 123 | | gttgttatatggTCTTGTGAccagagcaatct | 5' | >-108 |
| | *Anopheles_epiroticus* | Aepi\Atp6v1D | | 41 | 84 | 118 | 74 | 364 | | | 68 | 95 | 65 | 123 | | gttgttacatggTCTTGTGAtcagtacaatct | 5' | >-104 |
| | *Anopheles_minimus* | Amin\Atp6v1D | | 41 | 79 | 118 | 92 | 364 | | | 64 | 95 | 79 | 123 | | gtggttatatggTCTTGTGAtcagtggaacct | 5' | >-110 |
| | *Anopheles_funestus* | Afun\Atp6v1D | | 41 | 78 | 118 | 74 | 364 | | | 64 | 95 | 64 | 123 | | gttgttgtatggTCTTGTGAccagagaatctg | 5' | >-109 |
| | *Anopheles_stephensi* | Aste\Atp6v1D | | 41 | 79 | 118 | 80 | 364 | | | 71 | 95 | 76 | 123 | | gttgttatgtggTCTTGTGAccagaagagcct | 5' | >-110 |
| | *Anopheles_dirus* | Adir\Atp6v1D | 98 | 41 | 73 | 118 | 73 | 364 | | | 294 | 95 | 71 | 123 | | tgtgttctgtggTCTTGTGAccataatttgcg | 5' | >-108 |

| | | | | | | | | | | | | | | | | | | | |
|---|---|---|---|---|---|---|---|---|---|---|---|---|---|---|---|---|---|---|---|
| | *Anopheles_nili* | Anil\Atp6v1D | | 41 | 64 | 118 | 68 | 364 | | | 87 | 95 | 87 | 123 | | | gtgtgtaacgagTCTTGTGAccataatttgcg | 5' | >-90 |
| | *Anopheles_albimanus* | Aalb\Atp6v1D | 98 | 41 | 66 | 118 | 71 | 364 | | | 58 | 95 | 87 | 123 | | | atcactgtagggTCTTGTGAtcatcgggtttg | 5' | >-74 |
| | *Anopheles_darlingi* | Adar\Atp6v1D | | 41 | 68 | 118 | 71 | 364 | | | 77 | 95 | 87 | 123 | | | catcactgtaggTCTTGTGActgggtttgctg | 5' | >-79 |
| | *Aedes_aegypti* | Aaeg\Atp6v1D<br>AAEL009808 | 98 | 41 | 68 | 118 | 19182 | 364 | | | 8305 | 95 | 58 | 123 | 301 | | gatccatcaaaaTCACATGAcatgtgtcaaac<br>aaattcatttgaTCTTGTGAcccgttttgcgc | 5'<br>5'UTR | -15<br>27 |
| | *Culex_pipiens_qui.* | Cqui\Atp6v1D-1<br>CPIJ015086 | 84 | 741 | | | | | | | | | | | | | tcacacagttcgTCATGTGAcattttgctgcc<br>gtctcgatttgaTCTTGTGActtgaattttgg | 5'<br>5'UTR | -46<br>29 |
| | | Cqui\Atp6v1D-2<br>CPIJ009338 | | 741 | | | | | | | | | | | | | ND | | |
| Diptera (Cecidomyiidae) | *Mayetiola_destructor* | Mdes\Atp6v1D | | 41 | 282 | 118 | 103 | 180 | 72 | 184 | 94 | 95 | 85 | 123 | | | ttttctttagtgTCAGCTGActacttctcttg<br>gctgactacttcTCTTGTGAccatacacagaa | 5'<br>5' | >-103<br>>-89 |
| | | | | | | | | | | | | | | | | | | | |
| Lepidoptera | *Bombyx_mori* | Bmor\Atp6v1D<br>LOC692984 | 57 | 41 | 87 | 118 | 281 | 148 | 1308 | 216 | 1472 | 131 | 993 | 90 | 544 | | tgtcttctgtcaTCAGGTGAtttgggtttggg | 5'UTR | -5 |
| | *Manduca_sexta* | Msex\Atp6v1D-1 | | 41 | 100 | 118 | ND | 148 | 272 | 216 | 866 | 131 | 212 | 87 | | | tgtctgctgtcaTCAGGTGAtttaggttttgg | 5' | >-65 |
| | *Danaus_plexippus* | Dple\Atp6v1D-1 | | 41 | 77 | 118 | 666 | 148 | 1020 | 216 | 509 | 131 | 450 | 96 | | | tgtcttctgtcaTCAGGTGAtcgagaattagg | 5' | >-59 |
| | *Heliconius_melpomene* | Hmel\Atp6v1D-1 | | 41 | 96 | 118 | 857 | 148 | 382 | 216 | 933 | 131 | 573 | 93 | | | tgtcttctgtcaTCAGGTGAttgaaattggga | 5' | >-61 |
| | *Plutella_xylostella* | Pxyl\Atp6v1D | | 41 | 99 | 118 | 367 | 148 | 1515 | 216 | 571 | 131 | 432 | 87 | | | tgtcttctgtcaTCAGGTGAtcgtgagcaaaa | 5' | >-56 |
| | *Manduca_sexta* | Msex\Atp6v1D-2 | | 267 | | | 524 | 239 | 91 | 82 | 67 | 100 | 513 | 170? | | | tgtctgctgtcaTCAGGTGAtttaggttttgg | 5'? | ND |
| | *Danaus_plexippus* | Dple\Atp6v1D-2 | | 267 | | | 86 | 239 | 72 | 82 | 83 | 106 | 114 | 155 | | | tgtcttctgtcaTCAGGTGAtcgagaattagg | 5'? | ND |
| | *Heliconius_melpomene* | Hmel\Atp6v1D-2 | | 267 | | | 89 | 239 | 134 | 82 | 77 | 115 | 608 | 155 | | | tgtcttctgtcaTCAGGTGAttgaaattggga | 5' ? | ND |
| | | | | | | | | | | | | | | | | | | | |
| Strepsiptera | *Mengenilla_moldrzyki* | Mmol\Atp6v1D | | 41 | | 118 | 86 | 364 | | | ND | 227 | | | | | ND | | |
| Coleoptera | *Tribolium_castaneum* | Tcas\Atp6v1D<br>LOC660332 | | 41 | 108 | 118 | 46 | 148 | 57 | 216 | 46 | 215 (131 + 84) | | | | | tatgtcacgcggTCAGCTGActgatctcgtga | 5' | >-37 |
| | *Dendroctonus_ponderosae* | Dpon\Atp6v1D | | 41 | 204 | 118 | 59 | 148 | 60 | 216 | 59 | 224 | | | | | ggcaaaataaggTCAGCTGAtttcatgtcag<br>ctgattttcatgTCAGCTGActagtcttgtga | 5' | >-113<br>>-97 |
| | | | | | | | | | | | | | | | | | | | |
| Hymenoptera | *Apis_mellifera* | Amel\Atp6v1D<br>GB15226 | 44 | 41 | 342 | 118 | 109 | 486 | | | | | 74 | 93 | 572 | | cggtcttacgagTCAGCTGAtcaatttcattc | 5'UTR | 6 |
| | *Apis_florea* | Aflo\Atp6v1D | | 41 | 331 | 118 | 122 | 486 | | | | | 72 | 93 | | | cggtcttacgagTCAGCTGAtcaatttcattc | 5' | >-38 |
| | *Bombus_terrestris* | Bter\Atp6v1D<br>LOC100643813 | 47 | 41 | 349 | 118 | 72 | 486 | | | | | 80 | 96 | 748 | | cggtcctacgagTCAGCTGAtcagtttcattc | 5'UTR | 2 |
| | *Bombus_impatiens* | Bimp\Atp6v1D | | 41 | 741 | 118 | 72 | 486 | | | | | 82 | 96 | | | cggtcctacgagTCAGCTGAtcagtttcattc | 5' | >-38 |
| | *Megachile_rotundata* | Mrot\Atp6v1D | | 41 | 371 | 118 | 79 | 486 | | | | | 76 | 93 | | | cggtcccacaagTCAGCTGAtcaatcttattc | 5' | >-36 |
| | *Acromyrmex_echinatior* | Aech\Atp6v1D | | 41 | 547 | 118 | 185 | 486 | | | | | 192 | 93 | | | cggtctctcgtgTCAGCTGAtcaattccattc | 5' | >-39 |
| | *Atta_cephalotes* | Acep\Atp6v1D | | 41 | 555 | 118 | 173 | 486 | | | | | 180 | 93 | | | cggtctctcatgTCAGCTGAtcaatcccgttc | 5' | >-39 |
| | *Solenopsis_invicta* | Sinv\Atp6v1D | | 41 | 526 | 118 | 961 | 486 | | | | | 131 | 93 | | | cggtctctcacgTCAGCTGAtcaatcccattc | 5' | >-38 |

| | | | | | | | | | | | | | | | | | | | | | | | | | |
|---|---|---|---|---|---|---|---|---|---|---|---|---|---|---|---|---|---|---|---|---|---|---|---|---|---|
| | *Pogonomyrmex_barbatus* | Pbar\Atp6v1D | | ND | ND | 118 | 167 | 486 | | | | | | | | | | | 129 | 93 | | | ND | | |
| | *Camponotus_floridanus* | Cflo\Atp6v1D | | 41 | ND | 118 | 170 | 486 | | | | | | | | | | | 70 | 93 | | | ggtcagctcgagTCAGCTGAtcaatcccgttc | 5' | >-41 |
| | *Harpegnathos_saltator* | Hsal\Atp6v1D | | 41 | 371 | 118 | 221 | 486 | | | | | | | | | | | 620 | 93 | | | cggtcgcccgagTCAGCTGAtcaatcccattc | 5' | >-4 |
| | *Linepithema_humile* | Lhum\Atp6v1D | | 41 | 512 | 118 | 96 | 486 | | | | | | | | | | | 162 | 93 | | | cggtctctcgtgTCAGCTGAtcaatcccgttc | 5' | >-39 |
| | *Nasonia_vitripennis* | Nvit\Atp6v1D NV16729 | 25 | 41 | 375 | 118 | 100 | 486 | | | | | | | | | | | 82 | 102 | 1002 | | tttccgtcaaagTCAGCTGAtcaatcgtattc | 5' | -12 |
| | *Nasonia_giraulti* | Ngir\Atp6v1D | | 41 | 377 | 118 | 100 | 486 | | | | | | | | | | | 82 | 102 | | | tttccgtcaaagTCAGCTGAtcaatcgtattc | 5' | >-37 |
| | *Nasonia_longicornis* | Nlon\Atp6v1D | | 41 | 377 | 118 | 100 | 486 | | | | | | | | | | | 82 | 102 | | | tttccgtcaaagTCAGCTGAtcaatcgtattc | 5' | >-37 |
| Hemiptera | *Acyrthosiphon_pisum* | Apis\Atp6v1D ACYPI000083 | 260 | 41 | 328 | 118 | 93 | 148 | | | 62 | 216 | | | | | 94 | 79 | 427 | 130 | 840 | | ND | | |
| | *Rhodnius_prolixus* | Rpro\Atp6v1D | | 41 | 118 | 118 | 1609 | 148 | | | ND | 216 | | | | | 506 | 79 | 1637 | 121 | | | tacacattataaTCAGCTGAtgtaccttccct | 5' | >-129 |
| Phthiraptera | *Pediculus_humanus* | Phum\Atp6v1D PHUM603950 | | 41 | 251 | 266 (118+148) | | | | | 108 | 216 | | | | | 207 | 79 | 74 | 127 | | | tggaaataatagTCAGCTGActttagctctga | 5' | >-38 |
| Odonata | *Ladona_fulva* | Lful\Atp6v1D | | 41 | 76 | 118 | 72 | 148 | | | 72 | 216 | | | | | 115 | 79 | 230 | 148 | | | tacctaattgtgTCAGCTGActttacgcctac | 5' | >-35 |
| **Crustacea** | *Daphnia_pulex* | Dpul\Atp6v1D | | 41 | 107 | 118 | 62 | 148 | | | 61 | 216 (45+171) | | | | | 78 | 79 | 62 | 142 | | | cttctcttgttgTCACATGAtcagatgattct | 5' | >-106 |
| Ixodida | *Ixodes_scapularis* | Isca\Atp6v1D | | 41 | 148 | 118 | ND | 148 (80+68) | | | 920 | 45 | 2332 | 171 (104+67) | | | ND | 79 | 2484 | 145 | | | ND | | |
| | *Homo_sapiens* | ATP6V1D | 305 | 41 | 6617 | 118 | 2232 | 80 | 1506 | 68 | 1583 | 45 | 1537 | 104 | 2329 | 67 | 2853 | 79 | 1677 | 142(86+56) | 756 | | caacaagaggggTCAAGTGAcacaaccagctgac | 5'UTR | +191 |
| Cnidaria | *Nematostella_vectensis* | Nvec\Atp6v1D v1g104847 | 43 | 38 | 117 | 118 | 287 | 80 | 618 | 113 (68+45) | | | 273 | 104 | 126 | 67 | 320 | 79 | 99 | 86+446+56 | 98 | | ND | | |
| Placozoa | *Trichoplax_adhaerens* | Tadh\Atp6v1D | 35 | 41 | 152 | 118 | 515 | 80 | 135 | 68 | 373 | 45 | 132 | 104 | 93 | 146 (67+79) | | | 62 | 68+83+65 | | | tccgtgatcaTCAAGTGACacagccagg | 5' | -29 |
