# Supplementary material for: Structural and Comparative Analyses of Insects Suggest the Presence of an Ultra-Conserved Regulatory Element of the Genes Encoding Vacuolar-Type ATPase Subunits and Assembly Factors
Source: Biology (Basel). 2023 Aug 13;12(8):1127. doi: 10.3390/biology12081127 (PMC10452791; doi:10.3390/biology12081127)
Supplement: Supplementary file 1 [file biology-12-01127-s001.zip › Supplementary Files S20-S37/Supp_File_S24_Atp6v1E_INS.pdf]

Gene Cluster ID	Atp6v1E
**Drosophila melanogaster gene**	**Vha26 (CG1088)**
FlyBase ID	FBgn0015324
**Predicted function**	**ATPase, H+ transporting, V1 subunit E**

Atp6v1E CLEAR element conservation:

```
                 5'/5'UTR                          intron1_                          intron1_                          intron1_
Dmel\    ACTTTCGTGAAATCATATGATCGATTTGCAGT  GGATCTCCCAGGTCATGTGAACTTTTCACCGC  AATTCGTGCTGGTCATGTGACACGGCCCCCGC  CATGTCCAGTTATCATGTGACACACAGGCAAC

Mdom\    AAAATTTCCCTTTCACATGATGGGATATTTAC  TCATGGGATATGTCATGTGAATATTTGTTGTC  CATTAACAGAAGTCATGTGATCAAAATGTAGG
Ccap\    AAATTTCCCCAATCATATGATAAAAAAAAAAG  TCATGGGATGTGTCATGTGAATACGAAGTGTA  CAATGGGGCTTGTCATGTGACCAACCGAACGT  GCTTACAACTAATCATGTGATTAAAGTTTGGA
Gmor\    TAACCCTGTTTATCATATGACCAAGAGAAAAA  AGATTCGAGTAGTCATGTGATCATTGGTTGAA
Llon\    TACAACTCCAAATCATATGACTGACTTTGGTC  TTTTCCTTGAAGTCATGTGACTCTTCGCCATG
Ppap\    TAAATACACGAATCATATGATCACCTTGGACA  TTTTCCTCCAAGTCATGTGACT--TCGCCATG


Agam\    --------------------------------  CGGGTCACGGGACCT--GTCATGTGAAAAGTG  ATGCAATATGTGTCACATGACCGACTCTCCCC  TACTGCTGGTAGTCATATGACGGGCTTACAAG
Aara\    --------------------------------  CGGGTCACGGGACCT--GTCATGTGAAAAGTG  ATGCAATATGTGTCACATGACCGACTCTCCCC  TACTGCTGGGAGTCATATGACGGGCTTACATG
Aqan\    --------------------------------  CGGGTCACGGGACCT--GTCATGTGAAAAGTG  ATGCAATATGTGTCACATGACCGACTCTCCCC  TACTGCTGGGAGTCATATGACGGGCTTACATG
Achr\    --------------------------------  CAGGTCACGGGACCT--GTCATGTGAAAAGTG  ATGCAATATGTGTCACATGACCGACTCCCTCC  TGCTGCTGGGAGTCATATGACAGGCTTATGTT
Aepi\    --------------------------------  CGGGTCACGGGACCT--GTCATGTGAAAAGTG  ATGCAATATGTGTCACATGACCAACTCCCCCC  ATCGTACTTGTGTCATATGACGGGCTTATGTT
Amin\    --------------------------------  CAGGTCACGGGACCC--GTCATGTGAAAAGTG  ATGCAATATGTGTCACATGACCCTCACGGGCA  CGTACAGGGGTGTCATATGACATGCTCTCGAT
Afun\    --------------------------------  CAGGTCACGGGACCC--GTCATGTGAAAAGTG  ATGCAATATGTGTCACATGACCCTCACGGCGA  AGGAGGGGGCGGTCATATGACATGCTCACGAT
Aste\    --------------------------------  TGGGTCACGGGACCC--GTCATGTGAAAAGTG  ATGCAATATGTGTCACATGACCTCCTCCGACG  CCTATGCTGGTGTCATATGACGTGCTTAGAAC
Adir\    --------------------------------  CGGGTCACGGGACCTCCGTCATGTGAAAAGTG  ACGAAATATGTGTCACATGACCCTCGGTGGAT  TTCGTGCTGGTGTCATATGACATGCTCAGGAG
Aalb\    --------------------------------  TGGGTCACGGGACCT--GTCATGTGAAAAGTG  AAAATATGTTGGTCACATGACTTCCTCCTCCT  CGGCGGCAAGTGTCATATGACATGCCGAAGAG
Adar\    --------------------------------  TGGGTCACGGGACCT--GTCATGTGAAAAGTG  AAAATATGTTGGTCACATGATCTGCGCCTCCT  CGGCGGCAAGTGTCATATGACATGGCGAGGGG
Cqui\    TTTTACCTTTGATCATGTGAGAAGTTGCTCAT  GTGGTCACGGGACCT--GTCATGTGAAGTGAC  CAAAAAAGAGAGTCACATGACCACTAGCATCG  --------------------------------
Aaeg\    CTCTTCTGTCTGTCATGTGAGATATTTTCCTT  GAGGTCACGGGACCT--GTCATGTGAAGCAAG  AAACAAAAAGTGTCACATGACCACTAGTGGCG  --------------------------------

Mdes\    CGAATGATAGAGTCATATGACTTATCAGACTA  TTGTTTCAAATGTCATGTGAATGAAATGATAT  TGTGTGCGAACGTCACATGATCAACATTTCAA  --------------------------------

                 5'/5'UTR                          intron2_
Bmor\    GCAACACTAAGATCATATGATTGTTCAATAAA  ACGAACGAACAATCATGTGACCAAGCAGTATT
Dple\    TAACATTAAAGATCATATGATTGTTCAATGAA  TATGTAATTATATCACATGACATCTTAGTATC
Msex\    GCAACACTGGGATCATATGATTGTTCAATGAA  GGTGTAGGTATGTCACATGAGAATTTTGCACA
Hmel\    CAACATTAATGATCATATGATTGTTCAATGAA  --------------------------------
Pxyl\    CAACACCAAAAATCATATGATCGTTCAATGAA  GTTGAATAGTGGTCATGTGATTATGTTCAAGG

Tcas\    ATAAGGTCAAAATCATATGATCAATCAGTCTG  TGCCTTTTCATGTCATGTGATCTATTGCAGTC
Dpon     CACCTGCTCCCATCATATGATCAATCTACAAC  ATCTCATGCTGGTCATGTGATTCGCATTTGAC

Amel\    CACACTACTGTGTCATATGATCGTCATTAACT
Aflo\    CACACTACTGTGTCATATGATCGTCATTAACT
Bter\    ACACTACCTGTGTCATATGATCGTCATTAACT
Bimp\    ACACTACCTGTGTCATATGATCGTCATTAACT
Mrot\    ACTAGTCTTGTGTCATATGATCATCATTAACT
Aech\    TCACACTTCGGGTCATATGACCGACGTTACGC
Pbar\    TCACACTTCGGGTCATATGACCGACGTTACGC
Hsal\    TCACATTTCGGGTCATATGATCGACGTTACGC
Acep\    TCATGCTTCGGGTCATATGACCGACGTTACGC
Lhum\    TCACGCTTTGGGTCATATGACCGACGTTACGC
```

```
Sinv\    TCACACTTTAGGTCATATGACCGACGTTACGC
Cflo\    CACATTTTCGAGTCATATGACCGACGTTACGC
Ngir\    TAACTCGGCCGGTCATATGATCGTCATGTGAG
Nlon\    TAACTCGGCCGGTCATATGATCGTCATGTGAG
Nvit\    TAACTCGGCTGGTCATATGATCGTCATGTGAG
Lful\    CGTTGTACGATATCATATGATCAACTCGTAAC
Phum\    TATGTGAATTGATCATATGATCGTCTGCAAAG

Dpul\    CACCCACTGCTGTCACATGACAAACCAAATCAGCTGACACTAGTAAGGA
Isca\    ACTCGGCGGACATCACATGACGGCTGTGCGGC
```

Atp6v1E gene structure comparison:

Key: UTR region, CDS region INTRON

Order	Species	Atp6v1E orthologs	5'exon		int	exon	int	exon			int	exon3'		Extended CLEAR region	Position	bps from TSS
Diptera (Drosophilidae)	*Drosophila_melanogaster*	Dmel\Vha26	275	33	529	213	78	327			61	108	1353	actttcgtgaaaTCATATGAtcgatttgcagt<br>ggatctcccaggTCATGTGAacttttcaccgc<br>aattcgtgctggTCATGTGAcacggcccccgc<br>catgtccagttaTCATGTGAcacacaggcaac	5'UTR<br>intron1<br>intron1<br>intron1	48<br>447<br>614<br>794
Diptera (Muscidae)	*Musca_domestica*	Mdom\Atp6v1E		33	1069	213	74	330			1462	108		aaaatttcccttTCACATGAtgggatatttac<br>tacaactaccaaTCACTTGActtgaaagttaa<br>tcatgggatatgTCATGTGAatatttgttgtc<br>cattaacagaagTCATGTGAtcaaaatgtagg	5'UTR?<br>5'UTR?<br>intron1<br>intron1	>-294<br>>-265<br>>231<br>>457
Diptera (Tephritidae)	*Ceratitis_capitata*	Ccap\Atp6v1E		33	797	213	62	327			142	108		aaatttccccaaTCATATGAtaaaaaaaaaag<br>tcatgggatgtgTCATGTGAatacgaagtgta<br>caatggggcttgTCATGTGAccaaccgaacgt<br>gcttacaactaaTCATGTGAttaaagtttgga	5'UTR?<br>intron1<br>intron1<br>intron1	>-305<br>>198<br>>390<br>>717
Diptera (Glossinidae)	*Glossina_morsitans*	Gmor\Atp6v1E	106+88	33	1629	213	64	327 (132+195)			858	108	223	taaccctgtttaTCATATGAccaagagaaaaa<br>agattcgagtagTCATGTGAtcattggttgaa	5'UTR?<br>intron1	-4<br>517
Diptera (Psychodidae)	*Lutzomyia_longipalpis*	Llon\Atp6v1E	ND	33	669	213	59	132	60	303			ND	tacaactccaaaTCATATGActgactttggtc<br>ttttccttgaagTCATGTGActcttcgccatg	5'UTR?<br>intron1	>-125<br>>442
	Phlebotomus_papatasi	Ppap\Atp6v1E	ND	33	609	213	ND	132	69	303 (195+108)			ND	taaatacacgaaTCATATGAtcaccttggaca<br>taaaaatcgacgTCATGTGAattcaattgatt<br>ttttcctccaagTCATGTGActtcgccatgtt	5'UTR?<br>intron1<br>intron1	>-122<br>>189<br>>411
Diptera (Culicidae)	*Anopheles_gambiae*	Agam\Atp6v1E<br>AGAP002401	226+147+33	33	826	345			79	195	74	108	ND	tcacgggacctgTCATGTGAaaagtgtcaaac<br>atgcaatatgtgTCACATGAccgactctcccc<br>tactgctggtagTCATATGAcgggcttacaag	intron1<br>intron1<br>intron1	440<br>717<br>974
	Anopheles_arabiensis	Aara\Atp6v1E	ND	33	830	345			79	195	74	108	ND	tcacgggacctgTCATGTGAaaagtgtcaaac<br>atgcaatatgtgTCACATGAccgactctcccc<br>tactgctgggagTCATATGAcgggcttacatg	intron1<br>intron1<br>intron1	>197<br>>476<br>>732
	Anopheles_quadriannulatus	Aqan\Atp6v1E	ND	33	829	345			80	195	74	108	ND	tcacgggacctgTCATGTGAaaagtgtcaaac<br>atgcaatatgtgTCACATGAccgactctcccc<br>tactgctgggagTCATATGAcgggcttacatg	intron1<br>intron1<br>intron1	>197<br>>475<br>>731
	Anopheles_christyi	Achr\Atp6v1E	ND	33	805	345			81	195	85	108	ND	tcacgggacctgTCATGTGAaaagtgtcaaac<br>atgcaatatgtgTCACATGAccgactccctcc<br>tgctgctgggagTCATATGAcaggcttatgtt	intron1<br>intron1<br>intron1	>194<br>>468<br>>696
	Anopheles_epiroticus	Aepi\Atp6v1E	ND	33	809	345			88	195	76	108	ND	tcacgggacctgTCATGTGAaaagtgtcaaac<br>atgcaatatgtgTCACATGAccaactcccccc<br>atcgtacttgtgTCATATGAcgggcttatgtt	intron1<br>intron1<br>intron1	>186<br>>442<br>>680

	Anopheles_minimus	Amin\Atp6v1E	ND	33	808	345			62	195	65	108	ND	tcacgggacccgTCATGTGAaaagtgtcaaac<br>atgcaatatgtgTCACATGAccctcacgggca<br>cgtacaggggtgTCATATGAcatgctctcgat	intron1<br>intron1<br>intron1	>190<br>>463<br>>695
	Anopheles_funestus	Afun\Atp6v1E	ND	33	796	345			74	195	62	108	ND	tcacgggacccgTCATGTGAaaagtgtcaaac<br>atgcaatatgtgTCACATGAccctcacggcga<br>aggagggggcggTCATATGAcatgctcacgat	intron1<br>intron1<br>intron1	>193<br>>459<br>>674
	Anopheles_stephensi	Aste\Atp6v1E	ND	33	789	345			89	195	74	108	ND	tcacgggacccgTCATGTGAaaagtgtcaaac<br>atgcaatatgtgTCACATGAcctcctccgacg<br>cctatgctggtgTCATATGAcgtgcttagaac	intron1<br>intron1<br>intron1	>194<br>>465<br>>681
	Anopheles_dirus	Adir\Atp6v1E	ND	33	820	345			69	195	64	108	ND	acgggacctccgTCATGTGAaaagtgtcaaac<br>acgaaatatgtgTCACATGAccctcggtggat<br>ttcgtgctggtgTCATATGAcatgctcaggag	intron1<br>intron1<br>intron1	>190<br>>459<br>>673
	Anopheles_nili	Anil\Atp6v1E	ND	ND	ND	ND			ND	ND	ND	108	ND	ND		
	Anopheles_albimanus	Aalb\Atp6v1E	ND	33	895	345			76	195	80	108	ND	tcacgggacctgTCATGTGAaaagtgacaatc<br>aaaatatgttggTCACATGActtcctcctcct<br>cggcggcaagtgTCATATGAcatgccgaagag	intron1<br>intron1<br>intron1	>315<br>>596<br>>828
	Anopheles_darlingi	Adar\Atp6v1E	ND	33	904	345 (213 + 132)			74	195	78	108	ND	tcacgggacctgTCATGTGAaaagtgacaatc<br>aaaatatgttggTCACATGAtctgcgcctcct<br>cggcggcaagtgTCATATGAcatggcgagggg	intron1<br>intron1<br>intron1	>317<br>>596<br>>831
	Aedes_aegypti	Aaeg\Atp6v1E<br>AAEL012035	287	33	7946	213	62	132	66	195	118	108	1928	ctcttctgtctgTCATGTGAgatattttcctt<br>tcacgggacctgTCATGTGAagcaagtgtatt<br>aaacaaaaagtgTCACATGAccactagtggcg	5'<br>intron1<br>intron1	-8<br>671<br>1037
	Culex_pipiens_qui.	Cqui\Atp6v1E<br>CPIJ006751	64+133+32	33	3758	213	66	132	55	195	65	108	537	ttttacctttgaTCATGTGAgaagttgctcat<br>tcacgggacctgTCATGTGAagtgacatcgcg<br>caaaaaagagagTCACATGAccactagcatcg<br>cctttgacaaacTCACGTGAaattttcttctc	5'<br>intron1<br>intron1<br>intron1	-27<br>>567<br>>870<br>>1485
Diptera (Cecidomyiidae)	*Mayetiola_destructor*	Mdes\Atp6v1E	ND	33	548	213	72	327 (132 + 195)			83	108	ND	cgaatgatagagTCATATGActtatcagacta<br>ttgtttcaaatgTCATGTGAatgaaatgatat<br>tgtgtgcgaacgTCACATGAtcaacatttcaa	5'<br>intron1<br>intron1	>-341<br>>191<br>>383
Lepidoptera	*Bombyx_mori*	Bmor\Atp6v1E<br>LOC732990	ND	33	186	176	2169	169	462	195	1422	108	ND	gcaacactaagaTCATATGAttgttcaataaa<br>acgaacgaacaaTCATGTGAccaagcagtatt	5'<br>intron2	>-119<br>>457
Lepidoptera	*Manduca_sexta*	Msex\Atp6v1E	ND	33	195	176	1821	169	618	195	510	108	ND	gcaacactgggaTCATATGAttgttcaatgaa<br>ggtgtaggtatgTCACATGAgaattttgcaca<br>ataaagttgatgTCATGTGActtcaaggttct	5'<br>intron2<br>intron2	>-121<br>>572<br>>832
Lepidoptera	*Danaus_plexippus*	Dple\Atp6v1E	ND	33	201	176	434	169	348	195	149	108	ND	taacattaaagaTCATATGAttgttcaatgaa<br>tatgtaattataTCACATGAcatcttagtatc	5'<br>intron2	>-120<br>>440
Lepidoptera	*Heliconius_melpomene*	Hmel\Atp6v1E	ND	33	193	176	909	169	308	195	319	108	ND	caacattaatgaTCATATGAttgttcaatgaa	5'	>-121
Lepidoptera	*Plutella_xylostella*	Pxyl\Atp6v1E	ND	33	184	176	2072	169	435	195	628	108	ND	caacaccaaaaaTCATATGAtcgttcaatgaa<br>gttgaatagtggTCATGTGAttatgttcaagg	5'<br>intron2	>-119<br>>665
Strepsiptera	*Mengenilla_moldrzyki*	Mmol\Atp6v1E	ND	33	54	176	970	169	819	303			ND	ND--		
Coleoptera	*Tribolium_castaneum*	Tcas\Atp6v1E<br>LOC659201	99	33	111	176	53	169	3968	303			ND	ataaggtcaaaaTCATATGAtcaatcagtctg<br>tgccttttcatgTCATGTGAtctattgcagtc	5'<br>intron2	-16<br>454
Coleoptera	*Dendroctonus_ponderosae*	Dpon\Atp6v1E	99	33	117	176	615	169	226	303 (184 +119)			ND	ataaaatggtggTCACATGAcagccaaatgtc<br>cacctgctcccaTCATATGAtcaatctacaac<br>atctcatgctggTCATGTGAttcgcatttgac	5'<br>5'<br>intron2	>-149<br>>-118<br>>381

Hymenoptera	*Apis_mellifera*	Amel\Atp6v1E<br>GB12913	194	33	914	176	133	169	202	184	217	119	301	cacactactgtgTCATATGAtcgtcattaact	5'UTR	64
	Apis_florea	Aflo\Atp6v1E	ND	33	1021	176	130	169	147	184	181	119	ND	cacactactgtgTCATATGAtcgtcattaact	5'UTR?	>-124
	Bombus_terrestris	Bter\Atp6v1E<br>LOC100648858	164	33	617	176	126	169	136	184	138	119	1377	acactacctgtgTCATATGAtcgtcattaact	5'UTR	16
	Bombus_impatiens	Bimp\Atp6v1E	ND	33	1413	176	129	169	136	184	138	119	ND	acactacctgtgTCATATGAtcgtcattaact	5'UTR?	>-140
	Megachile_rotundata	Mrot\Atp6v1E	ND	33	514	176	104	169	86	184	157	119	ND	actagtcttgtgTCATATGAtcatcattaact	5'UTR?	>-132
	Acromyrmex_echinatior	Aech\Atpv1E<br>AECH19756	ND	33	451	176	5279	169	101	184	195	119	ND	tcacacttcgggTCATATGAccgacgttacgc	5'	>-134
	Atta_cephalotes	Acep\Atp6v1E<br>ACEP15444	ND	33	469	176	4706	169	103	184	291	119	ND	tcatgcttcgggTCATATGAccgacgttacgc	5'	>-126
	Solenopsis_invicta	Sinv\Atp6v1E	88	33	590	176	194	169	97	184	140	119	486	tcacactttaggTCATATGAccgacgttacgc	5'	-46
	Camponotus_floridanus	Cflo\Atp6v1E	ND	33	555	176	169	169	141	184	195	119	ND	cacattttcgagTCATATGAccgacgttacgc	5'	>-136
	Harpegnathos_saltator	Hsal\Atp6v1E	ND	33	563	176	208	169	129	184	245	119	ND	tcacatttcgggTCATATGAtcgacgttacgc	5'	>-144
	Pogonomyrmex_barbatus	Pbar\Atp6v1E	ND	33	512	176	199	169	95	184	215	119	ND	tcacacttcgggTCATATGAccgacgttacgc	5'	>-132
	Linepithema_humile	LhumAtp6v1E	ND	33	566	176	174	169	129	184	74	119	ND	tcacgctttgggTCATATGAccgacgttacgc	5'	>-147
	Nasonia_vitripennis	Nvit\Atp6v1E<br>NV10403	>127	33	333	176	86	169	80	184	78	119	539	taactcggctggTCATATGAtcgtcatgtgag	5'	144
	Nasonia_giraulti	Ngir\Atp6v1E	ND	33	333	176	84	169	80	184	78	119	ND	taactcggccggTCATATGAtcgtcatgtgag	5'	ND
	Nasonia_longicornis	Nlon\Atp6v1E	ND	33	333	176	84	169	80	184	78	119	ND	taactcggccggTCATATGAtcgtcatgtgag	5'	ND
Hemiptera	*Acyrthosiphon_pisum*	Apis\Atp6v1E	ND	33	156	176	705	169	1177	303			ND	ND		
	Rhodnius_prolixus	Rpro\Atp6v1E	175	33	2126	176	1220	169	94	303			75	ND		
Phthiraptera	*Pediculus_humanus*	Phum\Atp6v1E<br>PHUM598510	ND	33	279	176	87	169	88	303 (152 + 151)			ND	tatgtgaattgaTCATATGAtcgtctgcaaag	5'	>-94
Odonata	*Ladona_fulva*	Lful\Atp6v1E		33	156	176	384	169	188	152	329	151		aacagaaatcatTCATATGAaccacttcgttg<br>cgttgtacgataTCATATGAtcaactcgtaac	5'<br>5'	>-141<br>>-114
**Crustacea**	*Daphnia_pulex*	Dpul\Atp6v1E	128	33	72	176(66+110)	64	169	59	152	71	151 (88+63)	275	cacccactgctgTCACATGAcaaaccaaaTCAGCTGAcactagtaagga	5'/5'UTR	-16/2
Ixodida	*Ixodes_scapularis*	Isca\Atp6v1E	98	33	122	66 244 110	ND	184+2187+276					93	actcggcggacaTCACATGAcggctgtgcggc	5'	-1
	Homo_sapiens	ATP6V1E1	127	33	9076	66 6140 110	332	67+11630+90	996	69+1738+95	3577	88+1792+63		ttgccccgcagcTCATGTGActtttgctcggg	intron1-2	333
Cnidaria	*Nematostella_vectensis*	Nvec/Atp6v1E	65	33	158	66 543 110	245	67+562+90	193	69+1053+95	875	88+875+63	409	ccaatatttccaTCACATGAaaatttatttac	5'UTR	19
Placozoa	*Trichoplax_adhaerens*	Tadh\Atp6v1E	129	33	115	66 98 110	206	67+155+90	83	69+146+95	193	151	405	tacaatttatctTCACGTGAcatgcgcttgat	5'	-21
