# Supplementary material for: Structural and Comparative Analyses of Insects Suggest the Presence of an Ultra-Conserved Regulatory Element of the Genes Encoding Vacuolar-Type ATPase Subunits and Assembly Factors
Source: Biology (Basel). 2023 Aug 13;12(8):1127. doi: 10.3390/biology12081127 (PMC10452791; doi:10.3390/biology12081127)
Supplement: Supplementary file 1 [file biology-12-01127-s001.zip › Supplementary Files S20-S37/Supp_File_S25_Atp6v1F_INS.pdf]

Gene Cluster ID	Atp6v1F
**Predicted function**	**ATPase, H+ transporting, lysosomal 14kDa, V1 subunit F**
**Drosophila melanogaster gene**	**Vha14-1 (CG8210)**
FlyBase ID	FBgn0262512

Atp6v1F CLEAR element conservation:

```
                5'/5'UTR
Dmel\    ACCGCAACTGTGTCATGTGATAGAGACCAGCT
Dwil\    TCGTGACAGATGTCAGCTGACCACTTTTTTCT
Mdom\    TTTTGACGTTTGTCATCTGACCAAAGAAGAAA
Ccap\    ATTTGACGTATGTCAGCTGACCATTTGCGAAC
Gmor\    TTTTGACATAAGTCATGAGACTTAAATTTCTA
Llon\    GTGCCCCAAAAGTCAGCTGATCTCTCAGAAAA
Ppap\    CACGTCGCAAAGTCAGCTGACACTTCTTGTTT

Agam\    GTTGTAAGTCAGTCAGGTGATGTTTTTCTCTT
Aara\    GTTGTAAGTCAGTCAGGTGATGTTTTTCTCTT
Aqan\    GTTGTAAGTCAGTCAGGTGATGTTTTTCTCTT
Achr\    GGTTGTAATCAGTCAGGTGATGTTTTTCGCAT
Aepi\    GGTTGTAGTCAGTCAGGTGATGTTTTTCTCTT
Amin\    GATCGTATTCAGTCAGGTGATTTTTTCTTCCC
Afun\    GGTCGTATTCAGTCAGGTGATATTTTTCTTCT
Aste\    GATTGTTTTCAGTCAGGTGACATTTTTCTCCA
Adir\    GGTTGTAGTCAGTCAGGTGATTGGTTTCTCAG
Aalb\    CCCCTTCTTCGGTCAGGTGACGTTTTTCTCAT
Adar\    CCCCCTCGTCGGTCAGGTGACGTTTTTCTCAA
Aaeg\    ATCAATTTTTGATCAGGTGACATATTTTCCTT
Cqui\    TTTTGCTTTTGATCAGGTGACATATTTTTCCT

Mdes\    TTGTGACACCAGTCAGGTGACAGCTTAGGAGA
Tcas\    AGGTGCTTTAAGTCAGGTGACAGTCTTGAATA
Dpon     CAGCTGATCTTGTCAGGTGACAATAATTAAAT
Bmor\    GTCAAAATTGAGTCAGGTGACGAGCCCTTGTG
Msex\    ACAAATTCGTAGTCAGGTGACGAGCCCTTGTG
Dple\    AAAAATCTATAGTCAGGTGATGATTTCTTGTG
Hmel\    AAAAATCTATAGTCAGGTGATGATTTCTTGTG
Pxyl\    AAAAATCCATAGTCAGGTGACGATCCCGTGTG

Amel\    AGGTGTGACTTGTCAGCTGATATATTATCAAG
Aflo\    AGGTGTGACTTGTCAGCTGATATATTATCAAG
Bter\    AGGTGTGACTTGTCAGCTGATGTATCGTCAGG
Bimp\    AGGTGTGACTTGTCAGCTGATGTATCGTCAAG
Mrot\    AGGCGTGACTTGTCAGCTGATGCATTAGCTGT
Aech\    CTATACGTTTCGTCAGCTGACGTGCAAGCGAC
```

```
Acep\   CTGTGGTATACATCAGCTGACGTGCAAGCGAC
Hsal\   AGGTGTGACTAGTCAGCTGATGTCCAACATAA
Lhum\   TAGCGTAACTTGTCAGCTGATGTTCGATAAAA
Sinv\   TGGTGTGGTTCGTCAGCTGATGTCTTACTGTT
Cflo\   TGGTGTGGCTCGTCAGCTGATGCGCGACAGAT
Pbar\   CTGACCACGTGATCACGTGACATTTGTCTCCC
Ngir\   GACGGTCGGAGGTCAGCTGATTTCTGCTAGCA
Nvit\   GACGGTCGGAGGTCAGCTGATTTCTGCTAGCA
Nlon\   GACGGTCGGAGGTCAGCTGATTTCTGCTAGCA

Phum\   tcagtaaacaaaTCATCTGAtatttcatcaat
Phum\   atttagtttaaaTCACCTGActaaatcagatt
Lful\   ACCCACAATAAAGCAGCTGAGGTTTTTAAATA
Dpul\   ttttttcacgagTCATGTGAcagttcccttttt
Isca\   ctgatcggactttTCAGCTGAgcgtacgggtgt
```

Atp6v1F gene structure comparison:

Key: UTR region, CDS region INTRON

Order	Species	Atp6v1F orthologs	5'exon	intron	exon	intron	exon	intron	exon	intron	exon3'		Extended CLEAR region	Position	bps from TSS
Diptera (Drosophilidae)	*Drosophila_melanogaster*	Dmel\Vha14-1	98	375							182		accgcaactgtgTCATGTGAtagagaccagct	5'	-3
	Drosophila_willistoni	Dwil\GK12172	54	407	82	90	206			60	33		tcgtgacagatgTCAGCTGAccactttttct	5'	
Diptera (Muscidae)	*Musca_domestica*	Mdom\Atp6v1F	54	67	82	61	206			72	33		ttttgacgtttgTCATCTGAccaaagaagaaa	5'	>-54
Diptera (Tephritidae)	*Ceratitis_capitata*	Ccap\Atp6v1F	51	59	82	63	206			60	30 122		atttgacgtatgTCAGCTGAccatttgcgaac	5'	>-86
Diptera (Glossinidae)	*Glossina_morsitans*	Gmor\Atp6v1F	53 54	63	82	63	206			57	33 122		ttttgacataagTCATGAGActtaaatttcta	5'	16
Diptera (Psychodidae)	*Lutzomyia_longipalpis*	Llon\Atp6v1F	54	57	82	68	206			59	45		gtgccccaaaagTCAGCTGAtctctcagaaaa	5'	>-41
	Phlebotomus_papatasi	Ppap\Atp6v1F	54	57	82	61	206			58	45		cacgtcgcaaagTCAGCTGAcacttcttgttt	5'	>-44
Diptera (Culicidae)	*Anopheles_gambiae*	Agam\Atp6v1F AGAP002473	83 54	158	82	86	206			86	42 224		gttgtaagtcagTCAGGTGAtgtttttctctt	5'	-9
	Anopheles_arabiensis	Aara\Atp6v1F	54	159	82	86	206			86	42		gttgtaagtcagTCAGGTGAtgtttttctctt	5'	>-93
	Anopheles_quadriannulatus	Aqan\Atp6v1F	54	158	82	89	206			86	42		gttgtaagtcagTCAGGTGAtgtttttctctt	5'	>-93
	Anopheles_christyi	Achr\Atp6v1F	54	135	82	69	206			85	42		ggttgtaatcagTCAGGTGAtgtttttcgcat	5'	>-91

	Anopheles_epiroticus	Aepi\Atp6v1F		54	145	82	92	206			84	42		ggttgtagtcagTCAGGTGAtgtttttctctt	5'	>-87
	Anopheles_minimus	Amin\Atp6v1F		54	147	82	75	206			67	42		gatcgtattcagTCAGGTGAttttttcttccc	5'	>-105
	Anopheles_funestus	Afun\Atp6v1F		54	137	82	97	206			66	42		ggtcgtattcagTCAGGTGAtatttttcttct	5'	>-104
	Anopheles_stephensi	Aste\Atp6v1F		54	162	82	73	206			75	42		gattgttttcagTCAGGTGAcatttttctcca	5'	>-103
	Anopheles_dirus	Adir\Atp6v1F		54	161	82	70	206			77	42		ggttgtagtcagTCAGGTGAttggtttctcag	5'	>-124
	Anopheles_nili	Anil\Atp6v1F		54	171	82	74	206			57	42		gatctattgcagTCAGGTGAcatccgagtttt	5'	>-109
	Anopheles_albimanus	Aalb\Atp6v1F		54	113	82	98	206			68	42		ccccttcttcggTCAGGTGAcgtttttctcat	5'	>-113
	Anopheles_darlingi	Adar\Atp6v1F		54	113	82	88	206			62	42	421	ccccctcgtcggTCAGGTGAcgtttttctcaa	5'	>-109
	Aedes_aegypti	Aaeg\Atp6v1F<br>AAEL002464	128	54	102	82	108	206			64	42	421	atcaatttttgaTCAGGTGAcatattttcctt	5'UTR	2
	Culex_pipiens_qui.	Cqui\Atp6v1F<br>CPIJ016432		54	118	82	63	248 (206+42)					175	ttttgcttttgaTCAGGTGAcatatttttcct	5'	>-116
Diptera (Cecidomyiidae)	*Mayetiola_destructor*	Mdes\Atp6v1F		136 (54+82)			119	206			118	36		ttgtgacaccagTCAGGTGAcagcttaggaga	5'	>-131
Lepidoptera	*Bombyx_mori*	Bmor\Atp6v1F	98	54	121	82	663	206			595	33	375	gtcaaaattgagTCAGGTGAcgagcccttgtg	5'UTR	26
	Manduca_sexta	Msex\Atp6v1F		54	147	82	1739	206			437	33		acaaattcgtagTCAGGTGAcgagcccttgtg	5'	>-64
	Danaus_plexippus	Dple\Atp6v1F		54	127	82	77	206			275	33		aaaaatctatagTCAGGTGAtgatttcttgtg	5'	>-65
	Heliconius_melpomene	HmelAtp6v1F		54	142	82	134	206			540	33		aaaaatctatagTCAGGTGAtgatttcttgtg	5'	>-67
	Plutella_xylostella	Pxyl\Atp6v1F		54	127	82	188	206			232	33		aaaaatccatagTCAGGTGAcgatcccgtgtg	5'	>-72
Strepsiptera	*Mengenilla_moldrzyki*	Mmol\Atp6v1F		363										ND		
Coleoptera	*Tribolium_castaneum*	Tcas\Atp6v1F<br>LOC663894		54	51	82	47	206			58	30		aggtgctttaagTCAGGTGAcagtcttgaata	5'UTR?	>-40
	Dendroctonus_ponderosae	Dpon\Atp6v1F		54	57	82	115	206 (83+123)			92	30		gtcaacacacagTCAGCTGAtcttgtcaggtg<br>cagctgatcttgTCAGGTGAcaataattaaat	5'UTR?	>-57<br>>-44
Hymenoptera	*Apis_mellifera*	Amel\Atp6v1F<br>GB18107	343	54	119	82	140	83	247	123	191	30	100	aggtgtgacttgTCAGCTGAtatattatcaag	5'UTR	262
	Apis_florea	Aflo\Atp6v1F		54	88	82	78	83	266	123	188	30		aggtgtgacttgTCAGCTGAtatattatcaag	5'UTR?	>-73
	Bombus_terrestris	Bter\Atp6v1F<br>LOC100651333	96+90+6	54	66	82	181	83	308	123	354	30	707	aggtgtgacttgTCAGCTGAtgtatcgtcagg	intron1-2	110
	Bombus_impatiens	Bimp\Atp6v1F		54	66	82	178	83	294	123	402	30		aggtgtgacttgTCAGCTGAtgtatcgtcaag	5'	>-74

	Megachile_rotundata	Mrot\Atp6v1F		54	65	82	203	83	304	123	306	30			aggcgtgacttgTCAGCTGAtgcattagctgt	5'	>-69
	Acromyrmex_echinatior	Aech\Atp6v1F		54	941	82	259	83	289	123	123	30			ctatacgtttcgTCAGCTGAcgtgcaagcgac	5'	>-76
	Atta_cephalotes	Acep\Atp6v1F		54	937	82	262	83	267	123	118	30			ctgtggtatacaTCAGCTGAcgtgcaagcgac	5'	>-182
	Solenopsis_invicta	Sinv\Atp6v1F		54	210	82	249	83	154	123	ND	ND			tggtgtggttcgTCAGCTGAtgtcttactgtt	5'	>-77
	Camponotus_floridanus	Cflo\Atp6v1F		54	1431	82	3431	83	2439	123	107	30			tggtgtggctcgTCAGCTGAtgcgcgacagat	5'	>-101
	Harpegnathos_saltator	Hsal\Atp6v1F		54	297	82	494	83	408	123	114	33			aggtgtgactagTCAGCTGAtgtccaacataa	5'	>-96
	Pogonomyrmex_barbatus	Pbar\Atp6v1F		54	143	82	263	83	136	123	105	30			ctgaccacgtgaTCACGTGAcatttgtctccc	5'	>-561
	Linepithema_humile	LhumAtp6v1F		54	416	82	358	83	481	123	110	33			tagcgtaacttgTCAGCTGAtgttcgataaaa	5'	>-109
	Nasonia_giraulti	Ngir\Atp6v1F		54	116	82	113	83	85	123	ND	30			gacggtcggaggTCAGCTGAtttctgctagca	5'	>-111
	Nasonia_longicornis	Nlon\Atp6v1F		54	116	82	113	83	85	123	ND	30			gacggtcggaggTCAGCTGAtttctgctagca	5'	>-111
	Nasonia_vitripennis	Nvit\atp6v1F<br>NV14793	568	54	116	82	113	83	85	123	90	30	590		gacggtcggaggTCAGCTGAtttctgctagca	5'UTR	449
Hemiptera	*Acyrthosiphon_pisum*	Apis\Atp6v1F<br>ACYPI000082	128	54	157	82	59	83	107	123	77	27			ND		
	Rhodnius_prolixus	Rpro\Atp6v1F		54	138	82	73	83	75	123	121	30			ND		
Phthiraptera	*Pediculus_humanus*	Phum\Atp6v1F<br>PHUM390990		136 (54+82)			91	206 (83+123)			167	30			tcagtaaacaaaTCATCTGAtatttcatcaat<br>atttagtttaaaTCACCTGActaaatcagatt	5'<br>5'	>-101<br>>-55
Odonata	*Ladona_fulva*	Lful\Atp6v1F		369											acccacaataaagCAGCTGAggtttttaaata	5'	>-39
**Crustacea**	*Daphnia_pulex*	Dpul\Atp6v1F		51	73	82	70	83	60	123	60	30			ttttttcacgagTCATGTGAcagttccctttt	5'	>-83
Ixodida	*Ixodes_scapularis*	Isca\Atp6v1F	132	45	374	82	80	83	ND	123	ND	30	320		ctgatcggacttTCAGCTGAgcgtacgggtgt	5'	28
	Homo_sapiens	ATP6V1F	102	158			2314	202					271		agcccccccaccTCACATGAtcgtgtgtcatg	intron1-2	459
Cnidaria	*Nematostella_vectensis*	Nvec\Atp6v1F	18	66	72	82	622	83	246	123	1000	30	344		gatgggtgaagaCCACATGAcccatttccggt	5'	21
Placozoa	*Trichoplax_adhaerens*	Tadh\Atp6v1F		69	74	82	76	83	632	123	322	30			ataggtagagcaTCACGTGAtggacatcggat	5'	>-138
