# Supplementary material for: Structural and Comparative Analyses of Insects Suggest the Presence of an Ultra-Conserved Regulatory Element of the Genes Encoding Vacuolar-Type ATPase Subunits and Assembly Factors
Source: Biology (Basel). 2023 Aug 13;12(8):1127. doi: 10.3390/biology12081127 (PMC10452791; doi:10.3390/biology12081127)
Supplement: Supplementary file 1 [file biology-12-01127-s001.zip › Supplementary Files S20-S37/Supp_File_S26_Atp6v1G_INS.pdf]

Gene Cluster ID	Atp6v1G
**Drosophila melanogaster gene**	**Vha13 (CG6213)**
FlyBase ID	FBgn0026753
**Predicted function**	**V-type ATPase, H+ transporting, V1 subunit G**

Atp6v1G CLEAR element conservation:

```
                   5'/5'UTR                       intron1

Dmel\Vha13       TGTGTGACTTCGTCATATGACATTTTTACAGT  GTTGTGTGTGGGTCACATGATGTGGTGGAAAA
Mdom\Atp6v1G     TCCAAAGCAATGTCATATGACATTTTTCGTTT  GCTATGGTGGGGTCACATGATAACCGAGCTTA
Ccap\Atp6v1G-1   TAGGTGGTTTTTTCATGTGACTGCGTTTGTCA  CAACCCATCGGGTCATGTGATAAGCAAAAATT
Gmor\Atp6v1G     TTTCTGTCTATGTCATATGACATTTACAATCC  AGTCCACAAGAGTCACATGATAAGATTATAGT
Llon\Atp6v1G     CGAAGTTGGGAGTCACATGATTTCTCTTATCG  AAATACTGTGAGTCACATGACGATTGTAATAA
Ppap\Atp6v1G-1   TTCAACGGTTGATCACATGATTTCTGTTATCG  GGGAATACTGAGTCACATGACGATGGTGATGA

Agam\Atp6v1G-1   ACCCGGGGGTTGTCATATGATCAGCGAAGAAA  ACACCACTCCGGTCACATGACCCACCTGTTTG
Aara\Atp6v1G-1   ACCCGGGGGTTGTCATATGATCAGCGAAGAAA  ACACCACTCCGGTCACATGACCCACCTGTTTG
Aqan\Atp6v1G-1   ACCCGGGGGTTGTCATATGATCAGCGAAGAAA  ACACCACTCCGGTCACATGACCCACCTGTTTG
Achr\Atp6v1G-1   AGTGGAGGGTTGTCATATGATCAACGATGAAA  ACTGACGGACGGTCACATGACCCACCTGTTGT
Aepi\Atp6v1G-1   GGAAGGGGATTGTCATATGATCAACGAAAAAA  ACGCAGCTCCGGTCACATGACCAACCTGTTGT
Amin\Atp6v1G-1   AAAGGGGGGTTGTCATATGATCAAAGAAAAAA  ACGTAACCCTGGTCACATGACCAACCTGTTGT
Afun\Atp6v1G-1   AAAAGGGGGTTGTCATATGATCAAAGAAAAAA  ACGTAACCCTGGTCACATGACCAACCTGTTGT
Aste\Atp6v1G-1   CAAAGTGGGTTGTCATATGATCAATGAAAAAA  CGTAACTTTGGGTCACATGACCCACCTGTTGT
Adir\Atp6v1G-1   AACGAGGGGTGGTCATATGATCTCGGAAAGAA  CGTAACGCACGGTCACATGACCCACCTGTTGT
Anil\Atp6v1G-1   TTGCAGGGTTTGTCATATGATCAAATTTGAAA  GTAACCACGGGGTCACATGACCCGCTGATGTG
Aalb\Atp6v1G-1   GAAACATCGTAGTCATATGATTGAGGGGTCGG  GACTGTGGCTGGTCACATGACCCTCCTCTGCT
Adar\Atp6v1G-1   TACGAGCGTAGGTCATATGATTGTGGGGTCGG  ACTGTGAATGGGTCACATGACCCTCTTCTTCG
Aaeg\Atp6v1G-1   CAACGATTTCAGTCATATGATCCGTCCTATAT  AGTGTACGGTAGTCACATGACCCATTGGACTA
Cqui\Atp6v1G-1   CAAGTGTCAGAGTCATATGATGCTCGCGCACA  CGCGCACGGTAGTCACATGACTACCTGCTGCT

Mdes\Atp6v1G-1   ATTTTTATTGGATCATATGATCCGAAATGAAA  TATACAAAGCGGTCACATGACTACATGATTGG
Bmor\Atp6v1G     TAACTAATTAAATCATATGACTTTCGTCACGA  AATCCATGGTCGTCACATGATTTAATTCAAAT
Msex\Atp6v1G     GAACTAATGTAATCATATGACTTTCGTCACGA  GGTCTTTTATCATCACATGATTGCTTTCATAT
Dple\Atp6v1G     GAACTAAACCAATCATATGACTATTTTCGTCA  --------------------------------
Hmel\Atp6v1G     GAACTAAACCAATCATATGACTTACTTTTCGT  TTACTTTTCTTATCACATGATATATCTTAAGA
Pxyl\Atp6v1G     GAGATAAACTAATCATATGACTTTCGTCACAT
Tcas\Atp6v1G-1   CCCAACTAGCGATCATATGACATTTGCCTCAA
Dpon\Atp6v1G-1   ATCGATTGGAAGTCATATGACTGTCACTGGGT

Amel\Atp6v1G     GAAACTCTAAAGTCATATGATCGATTTGCGAT
Aflo\Atp6v1G     GAAACTCTAAAGTCATATGATCGATTTGCGAT
Bter\Atp6v1G     AAAGTCATATGATCATATGATCGATTTGCGAT
Bimp\Atp6v1G     AAAGTCATATGATCATATGATCGATTTGCGAT
Mrot\Atp6v1G     GAAACTCCAAAGTCATATGATCGATTTGTAAT
```

```
Aech\Atp6v1G     GAAGCTCCAAAGTCATATGATCGATTCGTGAC
Acep\Atp6v1G     GAAGCTCCAAAGTCATATGATCGATTCGTGAC
Pbar\Atp6v1G     GAAGCTCCAAAGTCATATGATCGATTCGTGAC
Hsal\Atp6v1G     GAAGCTCCAAAGTCATATGATCGATTCGTGAT
Cflo\Atp6v1G     GAAGCTCCAAAGTCATATGATCGATTCGTGAT
Sinv\Atp6v1G     GAAGCTCTAAAGTCATATGATCGATTCGTGAC
Lhum\Atp6v1G     GAAGCGCCAAAGTCATATGATCGATTCATGAT
Ngir\Atp6v1G     GAGGTGCCTTGATCATATGATCGACTTTTCAG
Nlon\Atp6v1G     GAGGTGCCTTGATCATATGATCGATTTTTCAG
Nvit\Atp6v1G     GAGGTGCCTTGATCATATGATCGATTTTTCAG

Phum\Atp6v1G     TGCTTGTTACTGTCACGAGATCGATAAGAATT
Lful\Atp6v1G     TCCTGTGTCTTGTCATGTGATCGATCTGTAAC
Dpul\Atp6v1G     TGGCTCCAGCAGTCATATGACAGGAAGTCACA
Isca\Atp6v1G     GTAGGTCGCCGATCACCTGACTGCGGCAGTGT
```

Atp6v1G gene structure comparison:

Key: UTR region, CDS region

Order	Species	Atp6v1G orthologs	5'exon		intron	exon	intron	exon	intron	exon3'		Extended CLEAR region	Position	bps from TSS
Diptera (Drosophilidae)	*Drosophila_melanogaster*	Dmel\Vha13	117	82	367	101	59	171		250		tgtgtgacttcgTCATATGAcatttttacagt<br>gttgtgtgtgggTCACATGAtgtggtggaaaa	5'UTR<br>intron1	9<br>317
Diptera (Muscidae)	*Musca_domestica*	Mdom\Atp6v1G		82	259	101	3128	174				tccaaagcaatgTCATATGAcatttttcgttt<br>gctatggtggggTCACATGAtaaccgagctta	5'UTR<br>intron1	>-133<br>>259
Diptera (Tephritidae)	*Ceratitis_capitata*	Ccap\Atp6v1G-1		82	173	101	110	171				taggtggtttttTCATGTGActgcgtttgtca<br>caacccatcgggTCATGTGAtaagcaaaaatt	5'UTR?<br>intron1	>-133<br>>166
		Ccap\Atp6v1G-2		82	105	101	62	171				ND		
Diptera (Glossinidae)	*Glossina_morsitans*	Gmor\Atp6v1G<br>TMP007097	94	82	121	101	70	171		341		tttctgtctatgTCATATGAcatttacaatcc<br>agtccacaagagTCACATGAtaagattatagt	5'UTR<br>intron1	63<br>220
Diptera (Psychodidae)	*Lutzomyia_longipalpis*	Llon\Atp6v1G		82	113	278						cgaagttgggagTCACATGAtttctcttatcg<br>aaatactgtgagTCACATGAcgattgtaataa	5'UTR<br>intron1	>-118<br>>155
	Phlebotomus_papatasi	Ppap\Atp6v1G-1		82	109	278						ttcaacggttgaTCACATGAtttctgttatcg<br>gggaatactgagTCACATGAcgatggtgatga	5'UTR<br>intron1	
		Ppap\Atp6v1G-2		82	67	101	3682	171 (strand[-])				ND		
Diptera (Culicidae)	*Anopheles_gambiae*	Agam\Atp6v1G-1<br>AGAP001823	132	82	204	101	82	168		457		gcgctcttgcaaTCACGTGAaccaccgtgggg<br>acccgggggttgTCATATGAtcagcgaagaaa<br>acaccactccggTCACATGAcccacctgtttg	5'<br>5'<br>intron1	-73<br>-19<br>294
	Anopheles_arabiensis	Aara\Atp6v1G-1		82	204	101	82	168				gcgctcttgcaaTCACGTGAaccaccgtgggg<br>acccgggggttgTCATATGAtcagcgaagaaa<br>acaccactccggTCACATGAcccacctgtttg	5'<br>5'<br>intron1	>-205<br>>-152<br>>163

Anopheles_quadriannulatus	Aqan\Atp6v1G-1		82	215	101	87	168			acccgggggttgTCATATGAtcagcgaagaaa<br>acaccactccggTCACATGAcccacctgtttg	5'<br>intron1	>-152<br>>163
Anopheles_christyi	Achr\Atp6v1G-1		82	214	101	82	168			aaccgagtgcaaTCACGTGAacgctgcggacg<br>agtggagggttgTCATATGAtcaacgatgaaa<br>actgacggacggTCACATGAcccacctgttgt	5'<br>5'<br>intron1	>-196<br>>-141<br>>173
Anopheles_epiroticus	Aepi\Atp6v1G-1		82	191	101	87	168			cgatgtttgcaaTCACGTGAacccgtcggttg<br>ggaaggggattgTCATATGAtcaacgaaaaaa<br>acgcagctccggTCACATGAccaacctgttgt	5'<br>5'<br>intron1	>-213<br>>-141<br>>158
Anopheles_minimus	Amin\Atp6v1G-1		82	199	101	69	168			cactgtatgctaTCACGTGAaccaacctaccg<br>aaagggggttgTCATATGAtcaaagaaaaaa<br>acgtaaccctggTCACATGAccaacctgttgt	5'<br>5'<br>intron1	>-196<br>>-139<br>>145
Anopheles_funestus	Afun\Atp6v1G-1		82	222	101	68	168			aaaagggggttgTCATATGAtcaaagaaaaaa<br>acgtaaccctggTCACATGAccaacctgttgt	5'<br>intron1	>-176<br>>151
Anopheles_stephensi	Aste\Atp6v1G-1		82	207	101	77	171			caaagtgggttgTCATATGAtcaatgaaaaaa<br>cgtaactttgggTCACATGAcccacctgttgt	5'<br>intron1	>-145<br>>170
Anopheles_dirus	Adir\Atp6v1G-1		82	213	101	71	168			cgccgaacgctaTCACGTGAgagtgggggttg<br>aacgaggggtggTCATATGAtctcggaaagaa<br>cgtaacgcacggTCACATGAcccacctgttgt	5'<br>5'<br>intron1	>-213<br>>-162<br>>169
Anopheles_nili	Anil\Atp6v1G-1		82	>126	ND	ND	ND			ctctcgcacgagTCACGTGAgcaagtgttttt<br>ttgcagggtttgTCATATGAtcaaatttgaaa<br>gtaaccacggggTCACATGAcccgctgatgtg	5'<br>5'<br>intron1	>-160<br>>-130<br>>150
Anopheles_albimanus	Aalb\Atp6v1G-1		82	228	101	107	168			gaaacatcgtagTCATATGAttgaggggtcgg<br>gactgtggctggTCACATGAccctcctctgct	5'<br>intron1	>-170<br>>146
Anopheles_darlingi	Adar\Atp6v1G-1		82	253	101	113	168			tacgagcgtaggTCATATGAttgtggggtcgg<br>actgtgaatgggTCACATGAccctcttcttcg	5'<br>intron1	>-176<br>>151
Aedes_aegypti	Aaeg\Atp6v1G-1<br>AAEL012819	140	82	191	101	7661	168	381		gcgctcttgcaaTCACGTGAaccaccgtgggg<br>caacgatttcagTCATATGAtccgtcctatat<br>agtgtacggtagTCACATGAcccattggacta	5'<br>5'<br>intron1	-73<br>-20<br>295
Culex_pipiens_qui.	Cqui\Atp6v1G-1<br>CPIJ006975		82	171	101	5527	168			caagtgtcagagTCATATGAtgctcgcgcaca<br>cgcgcacggtagTCACATGActacctgctgct	5'<br>intron1	>-253<br>>123
Anopheles_gambiae	Agam\Atp6v1G-2<br>AGAP004867	126	82	239	101	75	174	1151		ND		
Anopheles_arabiensis	Aara\Atp6v1G-2		82	238	101	75	174			ND		
Anopheles_quadriannulatus	Aqan\Atp6v1G-2		82	238	101	75	174			ND		
Anopheles_christyi	Achr\Atp6v1G-2		82	212	101	74	174			ND		
Anopheles_epiroticus	Aepi\Atp6v1G-2		82	223	101	73	174			ND		
Anopheles_minimus	Amin\Atp6v1G-2		82	203	101	85	174			ND		
Anopheles_funestus	Afun\Atp6v1G-2		82	219	101	81	174			ND		
Anopheles_stephensi	Aste\Atp6v1G-2		82	208	101	89	174			ND		

	Anopheles_dirus	Adir\Atp6v1G-2		82	198	101	86	174					ND		
	Anopheles_nili	Anil\Atp6v1G-2		82	184	101	73ß	174					ND		
	Anopheles_albimanus	Aalb\Atp6v1G-2		82	187	101	74	180					ND		
	Anopheles_darlingi	Adar\Atp6v1G-2		82	182	101	71	180					ND		
	Aedes_aegypti	Aaeg\Atp6v1G-2<br>AAEL007184	133	82	152	101	10739	180			755		ND		
		Aaeg\Atp6v1G-3<br>AAEL013302		363									ND		
	Culex_pipiens_qui.	Cqui\Atp6v1G-2<br>CPIJ013849		82	207	101	6688	180					ND		
Diptera (Cecidomyiidae)	*Mayetiola_destructor*	Mdes\Atp6v1G-1		82	296	101	76	174					atttttattggaTCATATGAtccgaaatgaaa<br>cataatgttcgaTCATGTGAgtttatttgcct<br>tatacaaagcggTCACATGActacatgattgg	5'<br>intron1<br>intron1	>-146<br>>170<br>>209
		Mdes\Atp6v1G-2		82	782	101	95	183					ND		
Lepidoptera	*Bombyx_mori*	Bmor\Atp6v1G<br>LOC692985	93	82	917	101	ND	99	203	72	154		taactaattaaaTCATATGActttcgtcacga<br>aatccatggtcgTCACATGAtttaattcaaat	5'<br>intron1	-2<br>342
	Manduca_sexta	Msex\Atp6v1G		82	562	101	1005	99	1133	72			gaactaatgtaaTCATATGActttcgtcacga<br>ggtcttttatcaTCACATGAttgcttcatat	5'<br>intron1	>-87<br>>253
	Danaus_plexippus	Dple\Atp6v1G		82	560	101	503	99	84	72			gaactaaaccaaTCATATGActattttcgtca	5'	>-87
	Heliconius_melpomene	Hmel\Atp6v1G		82	1121	101	585	99	1216	72			gaactaaaccaaTCATATGActtacttttcgt<br>ttacttttcttaTCACATGAtatatcttaaga	5'<br>intron1	>-85<br>>284
	Plutella_xylostella	Pxyl\Atp6v1G		82	1024	101	1509	99	618	78			gagataaactaaTCATATGActttcgtcacat	5'	>-87
Strepsiptera	*Mengenilla_moldrzyki*	Mmol\Atp6v1G		82	68	287									
Coleoptera	*Tribolium_castaneum*	Tcas\Atp6v1G-1<br>LOC662804	37	82	86	200			50	69	253		cccaactagcgaTCATATGAcatttgcctcaa	5'	21
		Tcas\Atp6v1G-2<br>LOC658882		82	157	200			98	90			ND		
	Dendroctonus_ponderosae	Dpon\Atp6v1G-1		82	215	200			945	69			atcgattggaagTCATATGActgtcactgggt<br>tattccgttcagTCATGTGActctctgatcaa<br>gtgctggtttgcTCACATGAtgtacaggtgat	5'<br>intron2<br>intron2	>-97<br>>531<br>>679
		Dpon\Atp6v1G-2		85	206	200 (101+99)			52	90			ND		
Hymenoptera	*Apis_mellifera*	Amel\Atp6v1G<br>GB13499	76	82	412	101	117	99	79	75	741		gaaactctaaagTCATATGAtcgatttgcgat	5'UTR	26

	Apis_florea	Aflo\Atp6v1G		82	438	101	114	99	79	75			gaaactctaaagTCATATGAtcgatttgcgat	5'UTR?	>-42
	Bombus_terrestris	Bter\Atp6v1G		82	348	101	123	99	93	75			aaagTCATATGATCATATGAtcgatttgcgat	5'UTR?	>-48
	Bombus_impatiens	Bimp\Atp6v1G		82	348	101	123	99	90	75			aaagTCATATGATCATATGAtcgatttgcgat	5'UTR?	>-48
	Megachile_rotundata	Mrot\Atp6v1G		82	353	101	93	99	77	75			gaaactccaaagTCATATGAtcgatttgtaat	5'UTR?	>-48
	Acromyrmex_echinatior	Aech\Atp6v1G		82	560	101	95	99	86	75			gaagctccaaagTCATATGAtcgattcgtgac	5'UTR?	>-39
	Atta_cephalotes	Acep\Atp6v1G		82	466	101	92	99	84	75			gaagctccaaagTCATATGAtcgattcgtgac	5'UTR?	>-39
	Solenopsis_invicta	Sinv\Atp6v1G		82	820	101	92	99	79	75			gaagctctaaagTCATATGAtcgattcgtgac	5'UTR?	>-39
	Pogonomyrmex_barbatus	Pbar\Atp6v1G*		82	576	101	94	99	81	75			gaagctccaaagTCATATGAtcgattcgtgac	5'UTR?	>-39
	Linepithema_humile	Lhum\Atp6v1G		82	538	101	104	99	84	75			gaagcgccaaagTCATATGAtcgattcatgat	5'UTR?	>-44
	Harpegnathos_saltator	Hsal\Atp6v1G		82	586	101	118	99	85	75			gaagctccaaagTCATATGAtcgattcgtgat	5'UTR?	>-44
	Camponotus_floridanus	Cflo\Atp6v1G		82	561	101	107	99	122	75			gaagctccaaagTCATATGAtcgattcgtgat	5'UTR?	>-66
	Nasonia_giraulti	Ngir\Atp6v1G		360									gaggtgccttgaTCATATGAtcgacttttcag	5'UTR?	>-172
	Nasonia_longicornis	Nlon\Atp6v1G		360									gaggtgccttgaTCATATGAtcgatttttcag	5'UTR?	>-172
	Nasonia_vitripennis	Nvit\Atp6v1G NV17706	23+91+37	360									gaggtgccttgaTCATATGAtcgatttttcag	5'UTR?	-21
Hemiptera	*Acyrthosiphon_pisum*	Apis\Atp6v1G ACYPI000034	68	82	958	101	68	183			405		ND		
	Rhodnius_prolixus	Rpro\Atp6v1G	123	82	270	101	76	99	1773	72	187		ND		
Phthiraptera	*Pediculus_humanus*	Phum\Atp6v1G		82	ND	278							ttcttactatTCAAATGAcatttatttg	5'?	> -422
Odonata	*Ladona_fulva*	Lful\Atp6v1G		82	125	101	74	99	79	87			tcctgtgtcttgTCATGTGAtcgatctgtaac	5'UTR?	>-49
**Crustacea**	*Daphnia_pulex*	Dpul\Atp6v1G	79	82	425	101	62	171			161		tggctccagcagTCATATGAcaggaagtcaca	5'	-34
Ixodida	*Ixodes_scapularis*	Isca\Atp6v1G	86	82	117	101	ND	168			95		gtaggtcgccgaTCACCTGActgcggcagtgt	5'UTR	18
	Homo_sapiens	ATP6V1G1	125	82	4631	101	4917	174			1129		gttcaagagcggTCAGCTGAtgctacgctgtg<br>tacgctgtgcggTCACGTGAcaacgggggcga	5'<br>5'UTR?	-19<br>4
		ATP6V1G2	291	82	268	101	527	174			946		ND		
Cnidaria	*Nematostella_vectensis*	v1g191954	28	82	3361?	101	591	171			606		acgTCACGTGAccgagtaccaTCATGTGAtga<br>ttttgttatcttTCATATGAttttgctactac	5'<br>intron1	-12<br>285
Placozoa	*Trichoplax_adhaerens*	Tadh\Atp6v1G	39	82	287	101	776	171 (99+72)			124		tctgtcaagaggTCATATGAtcgcagatgtca	5'	-638
