# Supplementary material for: Structural and Comparative Analyses of Insects Suggest the Presence of an Ultra-Conserved Regulatory Element of the Genes Encoding Vacuolar-Type ATPase Subunits and Assembly Factors
Source: Biology (Basel). 2023 Aug 13;12(8):1127. doi: 10.3390/biology12081127 (PMC10452791; doi:10.3390/biology12081127)
Supplement: Supplementary file 1 [file biology-12-01127-s001.zip › Supplementary Files S20-S37/Supp_File_S27_Atp6v1H_INS.pdf]

Gene Cluster ID	Atp6v1H
**Drosophila melanogaster gene**	**VhaSFD (CG17332)**
FlyBase ID	FBgn0027779
**Predicted function**	**V-type ATPase, H+ transporting, V1 subunit H**

Atp6v1H CLEAR element(s) conservation:

```
              5'/5'UTR                                              intron1

Dmel\   GCTGCAGAAAAGTCACAAGACCA---GTCAGCTGAATTTCGGTGCTG
Gmor\   GAAGCGAAAAAGTCACAAGACTC---GTCAGCTGAATTCTTCCATTA
Mdom\   TTACATCACAAGTCACAAGACCTTCTGTCAGCTGAATTCAGAGTAAA
Ccap\       TTTCTGGGTCTTGTGAC-TTTTGTCAGCTGAATTTGTTCATCG   GGTAAGTGTGTGTCACATGAACATTTTGTGCG
Llon\   CAATCGTATTTGTCACGTGAAGTCAGAAAACT
Ppap\   CCCTCCATTCTGTCACGTGAATTCGAAGGAGC

Agam\   GTTCACTTCAGGTCATATGATCACAAATATTT                  GAGCAAATTCCATCACGTGATGTTGTTTCTTT
Aara\   GTTCACTTCAGGTCATATGATCACAAATATTT                  AGCTAAATTCCATCACGTGATGTTGTTTCTTT
Aqan\   GTTCACTTCAGGTCATATGATCACAAATATTT                  AGCTAAATTCCATCACGTGATGTTGTTTCTTT
Achr\   ATTCACTTTTGGTCATATGATCACAAATATTT                  AGTTGAATTTAATCACGTGATGTTGTTTTTTC
Aepi\   ATTCACTTTTGGTCATATGATCACAAATATTT                  AGTTAAATATCATCACGTGATGTTGTTTCTTT
Amin\   ATGCACTTTTGGTCATATGATCACAAATATTT                  ATTTAATTTCCATCACGTGATGGTATTTCTTT
Afun\   CTACACTTTAGGTCATATGATCACAAATATTT                  ATTTAATTTTCATCACGTGATGATGTTTCTTT
Aste\   CACCCCTTTAGGTCATATGATCACAAATATTT                  CTTTACTTTTCATCACGTGATGGTATTTCGTT
Adir\   AAGAAGAAGAGGTCATATGATCACAATTTTTT                  CCATCACTTCTATCACGTGATGAGTTTCGTTT
Anil\   AAGAAGAAGAGGTCATATGATCACAAATATTT                  AATCTACTTCCATCACGTGACTTACTTTTTTT
Aalb\   TTATCAGCGCAGTCATATGAGTTGATTTTTCA                  CTTCTTCGTTCGTCACGTGACGTTCTACGCTT
Adar\   TATTCAGCGCAGTCATATGAGTCCCCGAGAGT                  GTTCCTCGCTCATCACGTGACGTTCTACGTTT
Cqui\   CTTTACCCCAAGTCATATGACATTCAGCTGAG                  GAAATTTTGGAATCACGTGACCTTTTTCCTTC
Aaeg\   ACCCCATGCAAGTCATATGACATTCAGCTGCG                  AGAAATTTGGAATCACGTGATGCTTTTTGTGC


Mdes\   CCGATGGAAAAGTCACAAGAC-TCTCAGCTGAGGTTTTTTCTTC
Bmor\   CATTTCCTACAGTCACAAGACCTCTCACCTGAGCAGTGTCGGAT
Msex\   CACTTTTCATAGTCACAAGACCACTCAGCTGAGGCTGTGTTCGA
Dple\   CACGTCGAGTAGTCACAAGACCTCTCAGCTGAGTTCACTGTCGC
Hmel\   CACATCGAATAGTCACAAGACCTCTCAGCTGAGGTATCTGTCTA
Pxyl\   CACTTTCAATAGTCACAAGACCTCTCAGCTGAGGTTGTGTTGGT

Tcas\   GCGTGTCAATAATCACAAGACTTGTCAGGTGT                  GGCTGAGCACACTCACGTGACACTTGAGTATC
Dpon\   tcaaaagattggTCATATGAtcaatctgccag                  taatgaaactatTCACGTGAcattggtcaagt
Dpon    aatctgccagtgTCAGCTGAttgagactacgt

Aflo\   GTTATACATCGATCTTGTGATCCAACTATTGA
Amel\   GTTATACATCGATCTTGTGATCCAACTATTGA
Bimp\   GTAATATATCAGTCTTGTGATCCAAGTGTTGA
Bter\   GTAATATATCAGTCTTGTGATCCAAGTGTTGA
Mrot\   TATGTACATTGGTCTTGTGATTTTAACAGCTG
Aech\   ATATTCGTGTGGTCTTGTGATCGAACAGCTGA
Acep\   GTATTCGCGTGATCTTGTGATCGAAGAAGCTG
Sinv\   GTATTCGCATGGTCTTGTGATCGAACAGCTGA
Pbar\   GTATTTGCGTGGTCTTGTGATCGAACAGCTGA
Cflo\   GTATCTGCACGGTCTTGTGATCGAACAGCTGA
Lhum\   GTAGTTGATTGGTCTTGTGATCGAACAGCTGA
Hsal\   GTACCCAAGTGGTCTTGTGATCGAACAGCTGA
Ngir\   ACGCTACTCCAATCTCGTGACCTGACTTGTGA
Nlon\   ACGCTACTCCAATCTCGTGACCTGACTTGTGA
Nvit\   ACGCTACACCAATCTCGTGACCTGACTTGTGA

Phum\   CCCCATAGTTAATCACGTGAAAAATCGTAGGA
Lful\   gatcatggaatgTCACGAGAcaaggcagacgg
Dpul\   TTGGAGGGCCAGTCACATGACACAACTGACAA
Isca\   GTGTCTCCTCACTCACGTGACGTCTGCTGCTG
```

Atp6v1H gene structure comparison:

Key: UTR region, CDS region INTRON

Order	Species	Atp6v1H orthologs	5'ex		int	exon					int	ex	int	exon					int	ex	int	exon3'		Extended CLEAR region	Position	bps from TSS
Diptera (Drosophilidae)	*Drosophila_melanogaster*	Dmel\VhaSFD-RA (CG17332)	159	37	141	479					2016	135	64	563 (126+437)					60	114	160	79	681	gaaaagTCACAAGAccagTCAGCTGAatttcg	5'UTR	36
Diptera (Muscidae)	*Musca_domestica*	Mdom\Atp6v1H		46	136	479					1191	135	58	126	64	437			66	114	69	79		ttacatcacaagTCACAAGAccttctgTCAGCTGAattcagagtaaa	5'UTR?	>-100
Diptera (Tephritidae)	*Ceratitis_capitata*	Ccap\Atp6v1H		46	106	479					3452	135	86	126	72	437			56	114	249	85		ttgtgacttttgTCAGCTGAatttgttcatcg<br>ggtaagtgtgtgTCACATGAacattttgtgcg	5'UTR?<br>intron	>-117<br>>58
Diptera (Glossinidae)	*Glossina_morsitans*	Gmor\Atp6v1H	149	46	85	479					2729	135	84	126	62	437			57	114	171	79	115	aaaagTCACAAGActcgTCAGCTGAattcttccatta	5'UTR	9
Diptera (Psychodidae)	*Lutzomyia_longipalpis*	Llon\Atp6v1H	130	52	104	82	75	400			2509	698 (135+126+437)							72	205				caatcgtatttgTCACGTGAagtcagaaaa	5'UTR	33
	Phlebotomus_papatasi	Ppap\Atp6v1H		52	127	82	64	400			ND	698 (135+126+437)							364	202				ccctccattctgTCACGTGAattcgaaggagc	5'UTR?	>-86
Diptera (Culicidae)	*Anopheles_gambiae*	Agam\Atp6v1H<br>AGAP009486*		49	299	82	106	406			1237	135	78	563					69	114	89	79	99	gttcacttcaggTCATATGAtcacaaatattt<br>gagcaaattccaTCACGTGAtgttgtttcttt	5'UTR?<br>intron1	6<br>433
	Anopheles_arabiensis	Aara\Atp6v1H		49	308	82	100	406			1208	135	78	563					69	114	85	79		gttcacttcaggTCATATGAtcacaaatattt<br>agctaaattccaTCACGTGAtgttgtttcttt	5'<br>intron1	>-121<br>>327
	Anopheles_quadriannulatus	Aqan\Atp6v1H		49	315	82	100	406			1221	135	75	563					68	114	88	79		gttcacttcaggTCATATGAtcacaaatattt<br>agctaaattccaTCACGTGAtgttgtttcttt	5'<br>intron1	>-121<br>>334
	Anopheles_christyi	Achr\Atp6v1H		49	316	82	64	406			1087	135	78	563					63	114	76	79		attcacttttggTCATATGAtcacaaatattt<br>agttgaatttaaTCACGTGAtgttgtttttttc<br>caaggaagatctTCACGTGAccctaacggtag	5'<br>intron1<br>intron3	>-117<br>>336<br>>1651
	Anopheles_epiroticus	Aepi\Atp6v1H		49	294	82	95	406			1121	135	62	563					69	114	64	79		attcacttttggTCATATGAtcacaaatattt<br>agttaaatatcaTCACGTGAtgttgtttcttt	5'<br>intron1	>-129<br>>313
	Anopheles_minimus	Amin\Atp6v1H		49	294	82	70	406			974	135	68	563					65	114	67	79		atgcacttttggTCATATGAtcacaaatattt<br>atttaatttccaTCACGTGAtggtatttcttt	5'<br>intron1	>-115<br>>316
	Anopheles_funestus	Afun\Atp6v1H		49	285	82	70	406			988	135	70	563					83	114	67	79		ctacactttaggTCATATGAtcacaaatattt<br>atttaattttcaTCACGTGAtgatgtttcttt	5'<br>intron1	>-115<br>>307
	Anopheles_stephensi	Aste\Atp6v1H		49	302	82	76	406			1045	135	80	563					ND	114	72	79		caccccttaggTCATATGAtcacaaatattt<br>ctttacttttcaTCACGTGAtggtatttcgtt	5'<br>intron1	>-117<br>>323
	Anopheles_dirus	Adir\Atp6v1H		49	273	82	62	406			1013	135	68	563					67	114	71	79		aagaagaagaggTCATATGAtcacaatttttt<br>ccatcacttctaTCACGTGAtgagtttcgttt<br>gaaggaagatctTCACGTGAcccttacggtag	5'<br>intron1<br>intron3	>-133<br>>286<br>>1538
	Anopheles_nili	Anil\Atp6v1H		49	273	82	59	406			ND	135	70	563					78	114	69	79		aagaagaagaggTCATATGAtcacaaatattt<br>aatctacttccaTCACGTGActtactttttt	5'<br>intron1	>-118<br>>289
	Anopheles_albimanus	Aalb\Atp6v1H		49	270	82	72	394			1032	135	71	563					70	114	69	79		ttatcagcgcagTCACCTGAgttgatttttca<br>cttcttcgttcgTCACGTGAcgttctacgctt	5'<br>intron1	>-106<br>>284
	Anopheles_darlingi	Adar\Atp6v1H		49	261	82	74	394			1026	135	87	563					70	114	67	79		tattcagcgcagTCACCTGAgtccccgagagt<br>gttcctcgctcaTCACGTGAcgttctacgttt	5'<br>intron1	>-115<br>>274
	Culex_pipiens_qui.	Cqui\Atp6v1H<br>CPIJ002428		46	232	82	64	406			936	135	2950	563					56	114	59	79		ctttaccccaagTCATATGAcatTCAGCTGAg<br>gaaattttggaaTCACGTGAcctttttccttc	5'UTR?<br>intron1	>-135<br>>245
	Aedes_aegypti	Aaeg\Atp6v1H<br>AAEL006516	169	49	246	488 (82+406)					4353	135	59	563 (374+189)					404	114	76	79		accccatgcaagTCATATGAcattcagctgcg<br>agaaatttggaaTCACGTGAtgctttttgtgc	5'UTR<br>intron1	5<br>435
Diptera (Cecidomyiidae)	*Mayetiola_destructor*	Mdes\Atp6v1H		52	414	482 (82+400)					1809	135	86	374 (186+188)			68	189	68	114	76	94		gaaaagTCACAAGActcTCAGCTGAggttttttcttc<br>gcgataaaagttTCATTTGAattcgtttgtta	5'<br>5'	>-153<br>>-61
Lepidoptera	*Bombyx_mori*	Bmor\Atp6v1H<br>LOC733031	116	64	76	82	69	181	636	213	2275	135	321	186	480	188	440	189	256	114	352	76	143	catttcctacagTCACAAGAcctcTCACCTGAgcagtgtcggat	5'UTR	16
	Manduca_sexta	Msex\Atp6v1H		64	74	82	81	181	1136	213	2509	135	495	186	115	188	501	189	691	114	557	76		cacttttcatagTCACAAGAccacTCAGCTGAggctgtgttcga	5'	>-95
	Danaus_plexippus	Dple\Atp6v1H		52	94	82	67	181	358	210	2006?	135	303	186	108	188	256	189	486	114	361	79		cacgtcgagtagTCACAAGAcctcTCAGCTGAgttcactgtcgc	5'	>-98
	Heliconius_melpomene	Hmel\Atp6v1H		64	89	82	81	181	119	210	2506	135	675	186	524	188	595	189	252	114	241	79		cacatcgaatagTCACAAGAcctcTCAGCTGAggtatctgtcta	5'	>-95
	Plutella_xylostella	Pxyl\Atp6v1H		64	93	82	255	181	728	204	2035	135	437	186	596	188	716	189	1043	114	474	76		cactttcaatagTCACAAGAcctcTCAGCTGAggttgtgttggt	5'	>-93
Strepsiptera	*Mengenilla_moldrzyki*	Mmol\Atp6v1H		ND	ND	82	58	190	237	219	ND	360			ND		335		59	184?				ND		

Coleoptera	*Tribolium_castaneum*	Tcas\Atp6v1H<br>LOC657235*	83	79	232	82	49	190	4486	219	4351	695 (135+186+188+186)	48	199																								gcgtgtcaataaTCACAAGActtgtcaggtgt<br>ggctgagcacacTCACGTGAcacttgagtatc	5'UTR?<br>intron1	1<br>291
	Dendroctonus_ponderosae	Dpon\Atp6v1H	101	73	2654	82	59	190	4486	219	4351	135	55	225	60	135	ND	200	150	198	63	55	40+125															tcaaaagattggTCATATGAtcaatctgccag<br>aatctgccagtgTCAGCTGAttgagactacgt<br>taatgaaactatTCACGTGAcattggtcaagt	5'UTR?<br>5'UTR<br>intron1	-21<br>2<br>365
Hymenoptera	*Apis_florea*	Aflo\Atp6v1H		58	440	82	94	190	616	219	1125	206	78	85	107	407	63	114	111	94																			gttatacatcgaTCTTGTGAtccaactattga	5UTR?	>-99
	Apis_mellifera	Amel\Atp6v1H<br>GB17480	119	58	452	82	102	190	583	219	1023	206	87	85	99	407	63	114	122	94	439																	gttatacatcgaTCTTGTGAtccaactattga	5UTR	14
	Bombus_impatiens	Bimp\Atp6v1H		58	491	82	838	190	378	219	1203	206	85	85	875	407	73	114	102	91																			gtaatatatcagTCTTGTGAtccaagtgttga	5UTR?	>-102
	Bombus_terrestris	Bter\Atp6v1H<br>LOC100648051		58	506	82	936	190	372	219	1204	206	85	85	856	407	73	114	102	91																			gtaatatatcagTCTTGTGAtccaagtgttga	5UTR?	>-102
	Megachile_rotundata	Mrot\Atp6v1H		58	603	82	78	190	78	219	991	206	78	85	67	407	80	114	78	94																			tatgtacattggTCTTGTGAttttaacagctg	5UTR?	>-104
	Acromyrmex_echinatior	Aech\Atp6v1H<br>AECH24129*		61	200	82	196	190	455	219	ND	206	95	85	87	407	79	114	164	94																			atattcgtgtggTCTTGTGAtcgaacagctga	5'	>-99
	Atta_cephalotes	Acep\Atp6v1H<br>ACEP26838*		61	196	82	176	190	487	219	19366	206	97	85	77	407	75	114	163	94																			gtattcgcgtgaTCTTGTGAtcgaagaagctg	5'	>-102
	Solenopsis_invicta	Sinv\Atp6v1H<br>SINV11121*		58	195	82	168	190	775	219	ND	206	84	85	82	407	77	114	171	94																			gtattcgcatggTCTTGTGAtcgaacagctga	5'	>-102
	Pogonomyrmex_barbatus	Pbar\Atp6v1H<br>PB18819*		58	191	82	73	190	112	219	ND	206	91	85	92	407	119	114	188	88																			gtatttgcgtggTCTTGTGAtcgaacagctga	5'	
	Harpegnathos_saltator	Hsal\Atp6v1H<br>HSAL13159*		58	189	82	225	190	164	219	1499	206	69	85	82	407	82	114	137	94																			gtacccaagtggTCTTGTGAtcgaacagctga	5'	>-102
	Linepithema_humile	Lhum\Atp6v1H<br>LH13415*		58	191	82	122	190	118	219	1639	206	97	85	77	407	107	114	86	94																			gtagttgattggTCTTGTGAtcgaacagctga	5'	>-101
	Camponotus_floridanus	Cflo\Atp6v1H<br>CFLO15154*		58	190	82	159	190	121	219	1525	206	104	85	96	407	117	114	208	94																			gtatctgcacggTCTTGTGAtcgaacagctga	5'	
	Nasonia_giraulti	Ngir\Atp6v1H		58	144	82	76	190	68	219	1032	291	72	407	72	114	81	94																					ttcgtgggagtaTCAGCTGAtcaatacagcct<br>aacatcgccgcTCATCTGAcgtcactgacac<br>acgctactccaaTCTCGTGAcctgacttgtga	5UTR?	>-222<br>>-190<br>>-136
	Nasonia_longicornis	Nlon\Atp6v1H		58	144	82	82	190	68	219	1020	291	79	407	72	114	81	94																					ttcgtgggagtaTCAGCTGAtcaatacagcct<br>aacgtcgccgcTCATCTGAcgtcactgacac<br>acgctactccaaTCTCGTGAcctgacttgtga	5UTR?	>-222<br>>-190<br>>-136
	Nasonia_vitripennis	Nvit\Atp6v1H<br>NV13458	37	58	144	82	82	190	68	219	1053	291 (206+85)	79	407 (179+228)	72	114	81	94																					ttcgtgggagtaTCAGCTGAtcaatacagcct<br>aacgtcgccgcTCATCTGAcgtcactgacac<br>acgctacaccaaTCTCGTGAcctgacttgtga	5UTR?	>-186<br>>-154<br>>-99
Hemiptera	*Acyrthosiphon_pisum*	Apis\Atp6v1H<br>LOC100161058*	122	58	98	82	71	184	104	219	414	206	68	85	58	179	74	228	85	114	2505	79	330															ND		
	Rhodnius_prolixus	Rpro\Atp6v1H		58	76	82	69	190	169	219	73	206	71	85	66	179	87	228	157	114	1010	88																ND		
Phthiraptera	*Pediculus_humanus*	Phum\Atp6v1H<br>PHUM375970		61	333	82	203	190	89	219	148	206 (98+108)	472	264 (85+179)	132	228	107	114	556	82																			ccccatagttaaTCACGTGAaaaatcgtagga	5'	>-789
Odonata	*Ladona_fulva*	Lful\Atp6v1H		52	228	82	ND	190	1262	219 (114+105)	ND	108+ND+98	655	85	ND	ND	ND	228 (126+102)	ND	114	ND	94																gatcatggaatgTCACGAGAcaaggcagacgg	5'	>-126
**Crustacea**	*Daphnia_pulex*	Dpul\Atp6v1H	142	61	72	82	190	190	73	354	64	71	67	492 (85+179+126+102)	74	114	68	76	75																			ttggagggccagTCACATGAcacaactgacaa	5UTR	1
Ixodida	*Ixodes_scapularis*	Isca\Atp6v1H<br>ISCW013948*	24	64	ND	82	ND	190	ND	114	444	105	1960	98	1396	108	94	85	939	179	1483	126	642	102	ND	114	ND	73	45									gtgtctcctcacTCACGTGAcgtctgctgctg	5'	-11
	Homo_sapiens	ATP6V1H	422	113		103		90		114		105		98		193		179		126		102		114		61												atcattcaagTCATATGAacccattgac<br>ctaaaacttcTCACTGAactccaaaagc	5'	-596<br>-139
Cnidaria	*Nematostella_vectensis*	Nvec\Atp6v1H<br>v1g164874	8	95		100		90		44		70		105		98		111		88		72		48		53		79		47		102		114		34		tgcttcgttaTCACGTGAcagaagtcac	5'	-108
Placozoa	*Trichoplax_adhaerens*	Tadh\Atp6v1H<br>TRIADDRAFT_36227	ND	56		106		90		44		73		105		98		108		85		72		48		53		79		47		102		114		64		ND		

GO to protein sequence alignment
