# Supplementary material for: Structural and Comparative Analyses of Insects Suggest the Presence of an Ultra-Conserved Regulatory Element of the Genes Encoding Vacuolar-Type ATPase Subunits and Assembly Factors
Source: Biology (Basel). 2023 Aug 13;12(8):1127. doi: 10.3390/biology12081127 (PMC10452791; doi:10.3390/biology12081127)
Supplement: Supplementary file 1 [file biology-12-01127-s001.zip › Supplementary Files S20-S37/Supp_File_S28_Atp6v0a-1_INS.pdf]

Gene Cluster ID	Atp6v0a-1
Protein Cluster ID	ATP6V0A-1
**Drosophila melanogaster gene**	**Vha100-1 (CG1709)**
FlyBase ID	**FBgn0028671**
**Predicted function**	**ATPase, H+ transporting, lysosomal V0 subunit a1**

Atp6v0a-1 gene structure comparisons:

Key: UTR region, CDS region , alternatively spliced exons INTRON

Order	Species	Atp6v0a1 orthologs	5' exon	intron	5'exon		intron	exon	intron	exon	intron	exon	intron	exon	intron	exon	intron	exon	intron	exon	intron	exon	intron	exon	intron	exon	intron	exon	intron	exon	intron	exon	intron	exon	intron	exon3'		Extended CLEAR region	Position	bps from TSS
Diptera (Drosophilidae)	*Drosophila_melanogaster*	Dmel\Vha100-1	129	414	23	117	300	177	73	129	767	92 (158)	431	101+595+42	450	127	86	1156											ND	218	376	211	65	172	60	103	294	ND		
Diptera (Muscidae)	*Musca_domestica*	Mdom\Vha100-1				117	62	177	63	129	3674	95	4023	ND	ND	127	62	1159											58	218	305	205	1085	172	65	112		ND		
Diptera (Tephritidae)	*Ceratitis_capitata*	Ccap\Vha100-1				117	96	177	65	129	3674	95	4084	ND	ND	127	2346	1159											106	218	586	223	106	172	63	112		ND		
Diptera (Glossinidae)	*Glossina_morsitans*	Gmor\Atp6v0a1			40	117	64	177	52	129	3802	92	548	98+ND+ND	2108	127	57	1159											54	218	1184	208	729	172	60	112		ND		
Diptera (Psychodidae)	*Lutzomyia_longipalpis*	Llon\Atp6v0a1				117	79	177	172	129	ND	ND	ND	ND	ND	127	63	1902?																				ND		
	Phlebotomus_papatasi	Ppap\Atp6v0a1				117	70	177	62	129	ND	ND	ND	ND	ND	127	ND	1890																				ND		
Diptera (Culicidae)	*Anopheles_gambiae*	Agam\Atp6v0a1 AGAP003711	76	905	63	117	96	306			1909 (1858)	101 (152)	1969	95+438+42	1618	127	71	1380												79	250	88	172	95	103	614	gccctgccctgcTCATGTGAtgcgaggaaatc	5'	617
	Anopheles_arabiensis	Aara\Atp6v0a1				117	106	306			1913 (1862)	101 (152)	1997	95+432+42	1623	127	71	1380												79	250	87	172	95	103		gccctgccctgcTCATGTGAtgcgaggaaggc	5'	> -467
	Anopheles_quadriannulatus	Aqan\Atp6v0a1				117	98	306			1919 (1868)	101 (152)	1985	95+435+42	1633	127	71	1380												79	250	87	172	95	103		gccctgccctgcTCATGTGAtgcgaggaaatc aaaaaaactaaaTCACATGAaaaaatgttaaa	5'	> -471
	Anopheles_christyi	Achr\Atp6v0a1				117	95	306			1855 (1804)	101 (152)	1922	95+422+42	1567	127	87	1380												82	259	67	172	72	103		gccctgccctacTCATGTGAtgttagcaatcc tgaaatccaaccTCACATGAgttggggaatca	5'	> -411 > -370
	Anopheles_epiroticus	Aepi\Atp6v0a1				117	94	306			2040 (1997)	101 (152)	2041	95+411+42	1665	127	88	1380												68	256	76	172	103	103		tgtcctctttacTCATGTGAtgccagagaaat	5'	> -461
	Anopheles_minimus	Amin\Atp6v0a1				117	69	306			2304 (2253)	101 (152)	1830	95+429+42	1359	127	68	1380												89	256	94	172	84	103		gccttgagccacTCATGTGAtgttagaaaatc	5'	> -406
	Anopheles_funestus	Afun\Atp6v0a1				117	70	306			2268 (2217)	101 (152)	1818	95+432+42	1525	127	75	1377												77	256	80	172	89	103		gccttgcactacTCATGTGAtgttagaaaatc	5'	> -408
	Anopheles_stephensi	Aste\Atp6v0a1				117	98	305*(A base deletion at 95)			2438 (2387)	101 (152)	ND	ND	ND	127	80	1377												118	250	71	172	88	103		attatccgccacTCATGTGAtgatggtggtga	5'	> -436
	Anopheles_dirus	Adir\Atp6v0a1				117	99	306			1968 (1917)	101 (152)	1852	95+423+42	1449	127	81	1380												69	250	86	172	76	103		ND		
	Anopheles_nili	Anil\Atp6v0a1				117	66	306			ND	101 (152)	1669	95+417+42	ND	127	80	1380?												84	ND	ND	ND	ND	ND		ND		
	Anopheles_albimanus	Aalb\Atp6v0a1				117	113	306			2060 (2009)	101 (152)	1998	95+368+42	1282	127	98	1380												82	247	95	172	117	103		ND		
	Anopheles_darlingi	Adar\Atp6v0a1				117	98	306			2127 (2076)	101 (152)	2011	95+416+42	1250	127	91	1380												89	247	79	172	115	103		ND		
	Aedes_aegypti	Aaeg\Atp6v0a1 AAEL003743	119	21603	73	117	61	306			8727 (8673)	101 (155)	10223	98+373+42	865	127	10180	1374												171	247	1170	172	63	103	423	ND		
	Culex_pipiens_qui.	Cqui\Atp6v0a1 CPIJ005774				117	68	306			4573 (4519)	101 (155)	1694	95+510+42	912	127	1676	1618															78	172	63	103		ND		
Diptera (Cecidomyiidae)	*Mayetiola_destructor*	Mdes\Atp6v0a1				117	619	177	97	129	ND ND	89 (155)	ND	ND	ND	127	77	390			91	984								89	223	634	172	96	103		ND		
Lepidoptera	*Bombyx_mori*	Bmor\Atp6v0a1			183	117	2623	180	464	129	1376	86	18949	ND	ND	127	888	177	2018	213	219	151	433	140	885	237+487+113	1030	149?	ND	137?+ND+87	505	114+170+127	1170	133	1024	100	207	accgaataaatgTCATGTGActgattaaaaaa	intron1	1783
	Manduca_sexta	Msex\Atp6v0a1				117	4147	180	997	129	892	86	17147	ND	ND	127	522	177	2679	213	495	151	539	140	231	237+369+113	329	149	321	137+449+87	552	114+495+127	460	133	436	100		acttgtgaccagTCACATGActgattaaaaca	intron1	>1612
	Danaus_plexippus	Dple\Atp6v0a1			153	117	2089	180	794	129	626	86	698	89	5127	127	223	177	771	213	459	151	86	140	164	237+129+113	327	149	237	131+82+87	569	114+77+127	260	133	643	100		ttatcatcagaaTCATGTGAttgtaatataat	intron1	1463
	Heliconius_melpomene	Hmel\Atp6v0a1				117	3423	180	308	129	962	86	1001	89	ND	127	1367	177	ND	213	378	151	401	140	194	237+217+113	108	143	207	137+304+87	316	114+310+127	371	133	280	100		atcacagttaaaTCACATGAtttgattaatat	intron1	>1901
	Plutella_xylostella	Pxyl\Atp6v0a1			145	117	1777	180	1025	129	1488	86	878	89	ND	127	821	177	1952	213	277	151	297	140	2945	237+368+113	403	143	426	137+316+87	584	114+408+127	458	133	2598	100	64			
Strepsiptera	*Mengenilla_moldrzyki*	Mmol\Atp6v0a1				117	ND	177	ND	129	ND	ND	ND	ND	ND	127	48	390			459	291			540	350 (237+113)	1459	131	1723	239	989	217	848	172	3847	103		ND		
Coleoptera	*Tribolium_castaneum*	Tcas\Atp6v0a1			139	117	99	177	51	129	3195	86 (110)	1907	ND	ND	127	48	177	52	504 (213+151+140)					46	350 (237+113)	48	128	402	230	70	202	243	172	48	103		ND		
	Dendroctonus_ponderosae	Dpon\Atp6v0a1			132	117	3290	177	55	129	4022	86 (110)	7037	ND	ND	127	472	177	54	213	53	291			84	205+52+148	59	128	745	236	51	229	70	172	61	103	134	cgtcagcgttggTCACGTGAccacgccaaaat	5'	-332
Hymenoptera	*Apis_mellifera*	Amel\Atp6v0a1			199	117	365	306			ND	86 (113)	ND	101	ND	127	126	177	ND	213	78	769							158	230	632	220	173	172	ND	103		ttcacaaactgaTCATATGAcattaatgtaat	5'	-339
	Apis_florea	Aflo\Atp6v0a-1				117	1100	306?			13453	86 (113)	9185	101	6221	127	78	177	ND	213	71	769							141	230	691	220	174	172	285	103		ttcacaaactgaTCATATGAcattaacgtaat	5'	>-538
	Bombus_terrestris	Bter\Atp6v0a1 LOC100644609			286	117	2099	306			ND	86 (113)	6891	101	2531	127	101	177	360	213	77	769							75	233	96	217	81	172	144	103	526	ttcacaaactgaTCATATGAccacaaaggagt	5'	-401
	Bombus_impatiens	Bimp\Atp6v0a1				117	2090	306			7195	86 (113)	6896	101	2574	127	102	177	404	213	79	769							75	233	94	217	81	172	144	103		ttcacaaactgaTCATATGAccacaaaggagt	5'	>-678

	Megachile_rotundata	Mrot\Atp6v0a1				117	1596	306			4878	86 (113)	3368	101	2172	127	83	177	80	213	70	769							68	233	79	217	85	172	87	103			tctataagtttaTCATATGAcacgctaatcca	5'	>-697
	Acromyrmex_echinatior	Aech\Atp6v0a1				**117**	**161**	**306**			**7790**	**86 (113)**	12487	101	3250	127	175	177	372	213	91	1379													212	103			tttcggacgtgaTCATGTGAtcatgtcagatg	5'	>-979
	Atta_cephalotes	Acep\Atp6v0a1				117	156	306			9745	86 (113)	12045	101	3471	127	175	177	341	213	97	1379													211	103			tgaacaatttgaTCACATGAtcattgcgattg	5'	>-1615
	Solenopsis_invicta	Sinv\Atp6v0a1				117	963	306			6761	86 (113)	9313	101	3098	127	910	177	207	213	97	1418													860	103			ttttttaattcaTCAATTGAtcaatcgcatta<br>caatgagattgaTCAGTTGAtgaataaaaaaa	5'<br>5'	>-355<br>>-289
	Pogonomyrmex_barbatus	Pbar\Atp6v0a1				117	128	306			4123	86 (113)	5708	101	1762	127	215	177	147	213	74	1382													232	103			tttcagacgcgaTCATGTGAttatgtcagaca<br>atttttgtagcaTCACGTGAtcactgcaccct	5'<br>5'	>-791<br>>-413
	Camponotus_floridanus	Cflo\Atp6v0a1				117	189	306			3265	86 (113)	5672	101	1449	127	143	177	342	213	77	1379													258	103			ND		
	Linepithema_humile	Lhum\Atp6v0a1				117	178	306			3393	86 (113)	4984	101	1620	127	256	177	102	213	93	1394													706	103			caagaagcgctaTCACGTGAccattcgcgtat	5'	>-175
	Harpegnathos_saltator	Hsal\Atp6v0a1				117	346	306			3199	86 (113)	4614	101	1649	127	246	177	160	213	92	1388													332	103			cctcgctcaccaTCACGTGAcctgcgctcgcg	5'	>-206
	Nasonia_vitripennis	Nvit\Atp6v0a1			391	117	604	306			2338	86 (113)	3271	101	3316	127	248	390			80	778							65	224	356	208	80	172	82	112	660		tttttcgtccgaTCATATGAcaccttttgcac	5'	267
	Nasonia_giraulti	Ngir\Atp6v0a1				117	604	306			2350	86 (113)	3222	101	3317	127	249	390			80	778							65	224	355	208	80	172	82	112			tttttcgtccgaTCATATGAcaccttttgcac	5'	>-658
	Nasonia_longicornis	Nlon\Atp6v0a1				117	602	306			2366	86 (113)	3248	101	3338	127	248	390			80	778							65	224	355	208	80	172	82	112			tttttcgtccgaTCATATGAcaccttttgcac	5'	>-658
Odonata	*Ladona_fulva*	Lful\Atp6v0a1				117	150	177	1488	129	2642	86	ND	ND	ND	ND	ND	177	111	213	598	151	572	140	114	237+256+110	1594	125	80	116+354+108	763	259	162	172	ND	103			tatcttcgatatTCATATGAccgacttgagtc	5'	>-76
		Lful\Atp6v0a2				114	509	177	4068	129	465	86	812			127	372	177	ND	213	1289	151	127	140	1882	243+ND+113	122	125	131	116+ND+ND	5688	226	409	172	868	97			ND		
Phthiraptera	*Pediculus_humanus*	Phum\Atp6v0a1				117	1940	177	175	129	ND	86	ND	ND	ND	127	84	177	90	213	88	141	106	150	65	165+131+185	85	128	105	224	77	214	73	172	95	103			aaaataagttgaTCACATGAtgaaataataaa	intron1	>568
		Phum\Atp6v0a2				117	101	177	107	129	315	95	546			127	83	177	73	213	68	151	83	140	68	237+85+113	103	125	71	224	88	226	79	172	78	97			aagtttgttgTCACGTGAaaataagatt	intron 5	> 1726
**Crustacea**	*Daphnia_pulex*	Dpul\Atp6v0a1			136	114	161	306			813	86	1092	92	285	127	75	270+66+120			61	291			69	353	71	131	71	116+77+111	60	123+66+88	77	172	73	97	166		ND		
		Dpul\Atp6v0a2	ND		ND	114	116	231+82+79+117+76					232			65+72+62	61	270+93+120			401	285			68	222+81+146	66	131	76	146+66+111	505	198+139+88	79	175	64	94			ND		
Ixodida	*Ixodes_scapularis*	Isca\Atp6v0a1	ND		ND	117	95	79+ND+98	462	129	ND	86	5004			127	3251	83+97+94	413	213	455	151	1930	140	422	137+1495+79	995	119	4996	223	1932	382		172	3023	97			agaaaggggaaaTCATGTGAcattttcgggtc	intron2	>392
		Isca\Atp6v0a2	ND		ND	117	ND	79+ND+101	ND	129	ND	86	184			127	1850	83+ND+94+567+107+ND+55			ND	181	1965	140	2016	149+1374+82	ND	119	82	51+624+235+4884+111	ND	364	ND	175	ND				gggcgcaattTCACGTGAcaaaagcttt	intron 1	> 1792

Order	Species	Atp6v0a orthologs	5' exon	intron	5'exon		intron	exon	intron	exon	intron	exons	intron	exon	intron	exon	intron	exon	intron	exon	intron	exon	intron	exon	intron	exon	intron	exons	intron	exon	intron	exon	intron	exon3'			Extended CLEAR region	Position	bps from TSS
	Homo_sapiens	ATP6V0A1	120	1884	47	117	5417	79+1502+98	1982	129	7420	104+720+127+2077+83	2288	94	4023	213			3119	151	3696	140	560	155+437+91	3207	119	1664	217+273+108+6215+108	6233	118	310	172	6566	94			GGATCAAATGACAA	5'UTR	303
		ATP6V0A2			248	117	5940	79+3649+98	2205	138	1405	89+1497+127+6016+83	1522	94	1434	213			6513	151	265	137	259	188+97+91	2634	119	849	211+2324+120+1053+120	2017	118	2277	172	940	106	3723		ND		
		ATP6V0A3	104	2148	4	117	364	79+811+221			82	86+339+127+73+83	160	94	224	213			613	145	2330	140	74	158+77+91	906	119	83	214+368+126+173+105	70	118	232	178	76	79	81		ND		
Cnidaria	*Nematostella_vectensis*	Phum\Atp6v0a1				96+89+21	159	79+77+98	2396	129	418	110+699+210	703	94	128	78	176	135	264	151	490	140	511	152+99+97	501	119	354	46+550+84+649+87+105+108+335+114	192	118	362	102+594+70	541	94	423		gcatcagacttgTCACGTGAcaagaggtggtg<br>tgctgtggtgaaTCATGTGAcaaagtgggaat<br>ttcattctcttcTCACATGAattctatttaacaa	5'<br>intron1<br>intron3	>-92<br>>274<br>>901
Placozoa	*Trichoplax_adhaerens*	Tadh\Atp6v0a1				96	1159	79+228+98	259	129	185	116+91+127+192+83	140	94	87	78	75	135	115	151	106	140	77	140+105+88	83	119	93	46+82+84+173+87+98+117+151+126	94	118	108	172	75	106			ttgttgtgatcgTCACATGAtgtttgcgtcat<br>tgatgaacgtcaTCATGTGAacagattccata	5'<br>5'	>-440<br>>-376
		Tadh\Atp6v0a2				96+384+21	116	79+166+98	242	129	139	101+139+127+144+83	95	94	89	78	87	135	243	151	101	137	205	176+64+91	82	119	108	46+218+84+136+106+214+120+141+86	104	118	169	102+138+70	147	91			cacatgcagcaaTCATGTGAtaagaaatcaat	5'	>-160
		Tadh\Atp6v0a3				108+2785+21	449	79+453+98	129	129	215	107+94+127+95+83	77	172			86	135	116	151	87	137	155	140+92+88	132	119	146	46+81+84+73+84+75+114+75+84?	56	151	84	105+101+70	89	91					
