# Supplementary material for: Structural and Comparative Analyses of Insects Suggest the Presence of an Ultra-Conserved Regulatory Element of the Genes Encoding Vacuolar-Type ATPase Subunits and Assembly Factors
Source: Biology (Basel). 2023 Aug 13;12(8):1127. doi: 10.3390/biology12081127 (PMC10452791; doi:10.3390/biology12081127)
Supplement: Supplementary file 1 [file biology-12-01127-s001.zip › Supplementary Files S20-S37/Supp_File_S29_Atp6v0a-2_INS.pdf]

Gene Cluster ID	Atp6v0a
Protein Cluster ID	ATP6V0A2
**Drosophila melanogaster gene**	**Vha100-2 (CG18617)**
FlyBase ID	FBgn0028670
**Predicted function**	**ATPase, H+ transporting, lysosomal V0 subunit a2**

Go to Atp6V0a1 gene structure comparisons in Arthropoda

Atp6v0a2 gene structure comparisons:

Key: UTR region, CDS region

Order	Species	Vha100-2 Atp6v0a orthologs	5' exon	intron	5'exon		intron	exon	intron	exon	intron	exon	intron	exon	intron	exon	intron	exon	intron	exon	intron	exon	intron	exon	intron	exon	intron	exon	intron	exon	intron	exon	intron	exon3'		Position	bps from TSS
Diptera (Drosophilidae)	*Drosophila_melanogaster*	Dmel\Vha100-2	163	5582	21	117	66	1628																			60	259	62	235	71	266			308	intron1	685
Diptera (Muscidae)	*Musca_domestica*	Mdom\Atp6v0a2a	ND	ND	ND	117	ND	1881																					72	229	72	172	ND	94			
		Mdom\Atp6v0a2b	ND	ND	ND	117	69	1881																					131	241	71	172	76	94			
		Mdom\Atp6voa2c	ND	ND	ND	117	105	2098																							64	172	71	91		5'	
Diptera (Tephritidae)	*Ceratitis_capitata*	Ccap\Atp6v0a2a	ND	ND	ND	117	125	1887																					80	232	68	172	74	94			
		Ccap\Atp6v0a2b	ND	ND	ND	117	67	1863																					68	199	84	172	60	94			
		Ccap\Atp6v0a2c	ND	ND	ND	117	78	1866																					63	244	82	172	90	94		5'	
Diptera (Glossinidae)	*Glossina_morsitans*	Gmor\Atp6v0a2a	ND	ND	40	117	66	1890																					730	229	62	172	59	94	95	5'<br>5'UTR	-2172<br>3
		Gmor\Atp6v0a2b	82	2256	21	117	58	1835																					101	149	629	172	75	94			
Diptera (Psychodidae)	*Phlebotomus_papatasi*	Ppap\Atp6v0a2a	ND	ND	ND	117	ND	2125																					730	229	77	263				intron1	>1030
Diptera (Culicidae)	*Anopheles_gambiae*	Agam\Atp6v0a2a AGAP001588	ND	ND	ND	117	84	294			67	207	502	83	72	1460															68	172	77	94	40	5'<br>5'	>-302<br>>-262
	Anopheles_arabiensis	Aara\Atp6v0a2a	ND	ND	ND	117	85	294			67	207	495	83	72	1457															68	172	77	94		5'<br>5'	>-302<br>>-262
	Anopheles_quadriannulatus	Aqan\Atp6v0a2a	ND	ND	ND	117	85	294			65	207	485	83	72	1460															68	172	77	94		5'<br>5'	>-302<br>>-262
	Anopheles_christyi	Achr\Atp6v0a2a	ND	ND	ND	117	80	294			83	207	477	83	70	1454															70	172	73	94		5'<br>5'	>-277<br>>-237
	Anopheles_epiroticus	Aepi\Atp6v0a2a	ND	ND	ND	117	71	294			70	207	445	83	68	1454															77	172	63	94		5'<br>5'	>-317<br>>-277
	Anopheles_minimus	Amin\Atp6v0a2a	ND	ND	ND	117?	77	294			70	207	466	83	64	1454															65	172	67	94		5'<br>5'	>-329<br>>-289
	Anopheles_funestus	Afun\Atp6v0a2a	ND	ND	ND	117?	78	294			79	207	464	83	84	1454															83	172	83	94		5'<br>5'	>-335<br>>-297
	Anopheles_stephensi	Aste\Atp6v0a-2a	ND	ND	ND	117	74	294			77	207	474	83	83	1457															77	172	ND	94		5'<br>5'	>-321<br>>-283
	Anopheles_dirus	Adir\Atp6v0a2a	ND	ND	ND	117	92	294			76	207	501	83	81	1454															68	172	70	94		5'<br>5'	>-302<br>>-263
	Anopheles_nili	Anil\Atp6v0a2a	ND	ND	ND	117?	96	294			73	207	566	83	91	1454															65	172	81	94		5'	>-264
	Anopheles_albimanus	Aalb\Atp6v0a2a	ND	ND	ND	117?	84	294?			74	207	705	83	97	1454															64	172	74	94		5'<br>intron3<br>intron3	>-274<br>>823<br>>1011
	Anopheles_darlingi	Adar\Atp6v0a2a	ND	ND	ND	117	88	501					687	83	98	1454															66	172	71	94		5'<br>intron2	>-264<br>>751
	Aedes_aegypti	Aaeg\Atp6v0a2a AAEL006390	ND	ND	94	117	63	294			65	207	1133	83	21622	1445															65	172	58	97	79	intron1	225
	Culex_pipiens_qui.	Cqui\Atp6v0a2a, CPIJ017010	ND	ND	ND	117	60	294			63	210	120	83	2927	1448															61	172	71	97		intron2	>477
	Anopheles_gambiae	Agam\Atp6v0a2b AGAP001587	196	324	45	117	83	525							177	1377													78	208	81	172	74	97	207	intron1	327
	Anopheles_arabiensis	Aara\Atp6v0a2b	ND	ND	ND	117	85	525							174	1377													81	208	77	172	74	97		5'?	>-233
	Anopheles_quadriannulatus	Aqan\Atp6v0a2b	ND	ND	ND	117	82	525							176	1377													89	208	77	172	81	97		5'?	>-232
	Anopheles_christyi	Achr\Atp6v0a2b	ND	ND	ND	117	73	525							218	1377													71	208	75	172	69	97		5'?	>-210
	Anopheles_epiroticus	Aepi\Atp6v0a2b	ND	ND	ND	117	74	525							150	1377													62	202	69	172	68	97		5'?	>-218
	Anopheles_minimus	Amin\Atp6v0a2b	ND	ND	ND	117	65	525							147	1377													69	205	72	172	64	97		5'	>-199
	Anopheles_funestus	Afun\Atp6v0a2b	ND	ND	ND	117	68	525							145	1377													71	205	84	172	77	97		5'?	>-199
	Anopheles_stephensi	Aste\Atp6v0a2b	ND	ND	ND	117	81	525							136	1376* (C base deletion at 354)													73	205	74	172	75	97		5'?	>-302
	Anopheles_dirus	Adir\Atp6v0a2b	ND	ND	ND	117	70	525							80	1377													67	205	68	172	72	97		5'?	>-159
	Anopheles_nili	Anil\Atp6v0a2b	ND	ND	ND	117	71	>228							ND	1377													71	205	67	172	79	97		5'?	>-238
	Anopheles_albimanus	Aalb\Atp6v0a2b	ND	ND	ND	117	88	525							131	1383													73	190	69	172	76	97		5'?	> -273
	Anopheles_darlingi	Adar\Atp6v0a2b	ND	ND	ND	117	88	525							136	1384* (C base insertion at 190)													63	190	65	172	75	97		5'?	> -263
	Aedes_aegypti	Aaeg\Atp6v0a2b AAEL014053	ND	ND	ND	117	62	522							181	1588															62	172	549	97	247	intron1?	223

	Culex_pipiens_qui.	Cqui\Atp6v0a2b<br>CPIJ003274	52	98	35	117	66	513							66	1587* (A base deletion at 1499)															320	172	59	97	238	intron2?	477
Diptera (Cecidomyiidae)	*Mayetiola_destructor*	Mdes\Atp6v0a2	ND	ND	ND	117	81	774									104	73	93	276	71	260	82	510					89	235	66	172	84	103			
Lepidoptera	*Bombyx_mori*	Bmor\Atp6v0a2	77	16009	27	117	4407	177	1000	161	1089	181	1151			177	1158	213	526	151	ND	374	1362	110	568	128	1143	221	697	235	379	172	526	100	95	intron1	13141
	Manduca_sexta	Msex\Atp6v0a2				117	4168	177	1269	161	563	181	346			177	256	213	369	151	166	374	198	110	272	128		227	279	226	654	172	386	100		5'	>-690
	Danaus_plexippus	Dple\Atp6v0a2				117	2912	177	794	161	452	181	319			177	86	213	86	151?	156	374	71	110	117	128	382	224	161	220?	443	172	335	94			
	Heliconius_melpomene	Hmel\Atp6v0a2a				117	2468	177	792	161	424	181	309			177	226	213	303	151	352	374	234	110	218	128	302	224	561	232	465	172	456	97		intron1	>1359
		Hmel\Atp6v0a2b				108	4717	168	364	324			1604			177	1455	213	548	151	854	377	407	110	425	128	362	218	245	172?	294	172	188	127			
	Plutella_xylostella	Pxyl\Atp6v0a2a				117	2397	177	4393	155	464	181	588			177	ND	213	858	151	416	374	316	110	686	128	ND	227	347	241	470	172	335	97			
		Pxyl\Atp6v0a2b				ND	ND	177	735	155	338	181	459			177	1914	213	900	151	602	374	273	110	413	128	470	227	1057	241	446	172	574	97			
Srepsiptera	*Mengenilla_moldrzyki*	Mmol\Atp6v0a2				117	ND	522							ND	177	2944	213	57	151	ND	371	ND	110	53	128	ND	224	ND	214	66	172	ND	85			
Coleoptera	*Tribolium_castaneum*	Tcas\Atp6v0a2	47	1994	19	117	48	519							49	390 (177+213)			78	151	ND	484			42	131	49	441			56	172	48	100	67	5'	-39
	Dendroctonus_ponderosae	Dpon\Atp6v0a2a	58	527	20	117	ND	177	51	190	51	128	53	217	56	173	53	151	1457	166	52	229	ND	110	65	137	55	239	52	199	58	281			60	5'<br>intron1	-11<br>106
		Dpon\Atp6v0a2b				117	65	177	997	165	190	216	203	174	53	151	54	208	88	190	60			110	64	134	62	236	2236	238	58	272			155		
Hymenoptera	*Apis_mellifera*	Amel\Atp6v0a2	160	>24746	24	117	845	180	1032	129	110	83	654	127	1462	177	206	213	289	214	245	424			343	128	89	233	277	389			568	97	1130	intron1	16102
	Apis_florea	Aflo\Atp6v0a2				117	906	180	380	129	110	83	610	127	1454	177	277	213	387	214	297	424			301	128*	86	233	314	389			594	97		5'	>-1507
	Bombus_terrestris	Bter\Atp6v0a2	619	10596	24	117	1319	180	1493	129	112	83	2814	127	2050	177	956	213	576	214	1114	421			1978	128	103	230	785	389			523	97	1581	intron1	9973
	Bombus_impatiens	Bimp\Atp6v0a2				117	1257	180	800	129	112	83	2530	127	1997	177	1756	213	554	214	1081	421			1986	128	110	230	545	389			542	97		5'<br>intron1	>-1463<br>>331
	Megachile_rotundata	Mrot\Atp6v0a2				117	1756	180	1705	129	110	83	1331	127	2379	177	554	213	190	214	643	421			300	128	58	233	277	389			94	100			
	Acromyrmex_echinatior	Aech\Atp6v0a2				117	1138	180	487	129	119	83	712	127	233	177	240	213	478	214	101	427			365	128	94	233	360	398			359	97		5'<br>5'<br>intron1	-5545<br>-4818<br>658
	Atta_cephalotes	Acep\Atp6v0a2				117	1147	180	494	129	119	83	ND	127	235	177	254	213	ND	214	94	427			347	128	87	233	353	398			352	97		5'<br>5'<br>intron1	>-4813<br>>-1055<br>>537
	Solenopsis_invicta	Sinv\Atp6v0a2				117	733	180	689	129	113	83	1182	127	210	177	298	213	632	214	108	424			315	128	605	230	875	392			720	97			
	Pogonomyrmex_barbatus	Pbar\Atp6v0a2				117	430	180	364	129	116	83	696	127	240	177	245	213	454	214	90	427			352	128	74	233	151	398			383	97			
	Camponotus_floridanus	Cflo\Atp6v0a2				117	349	180	659	129	113	83	ND	ND	ND	177	238	213	279	214	96	430			363	128	76	233	462	395			380	97			
	Linepithema_humile	Lhum\Atp6v0a2				117	257	180	179	129	113	83	692	127	306	177	120	213	385	214	89	430			260	128	70	233	127	401			267	97		5'	>-1921
	Harpegnathos_saltator	Hsal\Atp6v0a2				117	423	180	204	129	143	86	443	127	264	177	228	213	96	214	150	421			381	128	86	239	330	416			415	97			
	Nasonia_vitripennis	Nvit\Atp6v0a2a	352	13600	60	117	76	309			?	86	116	127	73	177	74	213	87	214	111	433			92	128	83	227	88	395			97	106	902		
		Nvit\Atp6v0a2b	96	747	21	117	72	309			131	74	68	127	60	177	85	213	72	214	73	433			70	128	68	224	78	401			76	103	89	5'	-6
	Nasonia_giraulti	Ngir\Atp6v0a2a				117	74	309			110	86	116	127	73	177	74	213	87	214	111	433			92	128	81	227	96	395			96	106			
		Ngir\Atp6v0a2b				117	72	309			134	74	71	127	60	177	85	213	72	214	62	433			70	128	67	224	73	401			76	103		5'	>-870
	Nasonia_longicornis	Nlon\Atp6v0a2a				117	74	309			110	86	116	127	73	177	74	213	87	214	145	433			93	128	81	227	90	395			97	106			
		Nlon\Atp6v0a2b				117	72	309			131	74	71	127	60	177	85	213	72	214	62	433			70	128	67	224	73	401			76	103		5'	>-868

Order	Species	Atp6v0a orthologs	5' exon	intron	5'exon		intron	exon	intron	exon	intron	exon	intron	exon	intron	exon	intron	exon	intron	exon	intron	exon	intron	exon	intron	exon	intron	exon	intron	exon	intron	exon	intron	exon	intron	exon3'		Extended CLEAR region	Position	bps from TSS
Odonata	*Ladona_fulva*	Lful\Atp6v0a1				117	150	177	1488	129	2642	86	ND	ND	ND	ND	ND	177	111	213	598	151	572	140	114	237+256+110	1594	125	80	116+354+108	763	259	162	172	ND	103		tatcttcgatatTCATATGAccgacttgagtc	5'	>-76
		Lful\Atp6v0a2				114	509	177	4068	129	465	86	812			127	372	177	ND	213	1289	151	127	140	1882	243+ND+113	122	125	131	116+ND+ND	5688	226	409	172	868	97				
Phthiraptera	*Pediculus_humanus*	Phum\Atp6v0a1				117	1940	177	175	129	ND	86	ND	ND	ND	127	84	177	90	213	88	141	106	150	65	165+131+185	85	128	105	224	77	214	73	172	95	103		aaaataagttgaTCACATGAtgaaataataaa	intron1	>568
		Phum\Atp6v0a2				117	101	177	107	129	315	95	546			127	83	177	73	213	68	151	83	140	68	237+85+113	103	125	71	224	88	226	79	172	78	97				
**Crustacea**	*Daphnia_pulex*	Dpul\Atp6v0a1			136	114	161	306			813	86	1092	92	285	127	75	270+66+120			61	291			69	353	71	131	71	116+77+111	60	123+66+88	77	172	73	97	166			
		Dpul\Atp6v0a2				114	116	231+82+79+117+76					232			65+72+62	61	270+93+120			401	285			68	222+81+146	66	131	76	146+66+111	505	198+139+88	79	175	64	94				
Ixodida	*Ixodes_scapularis*	Isca\Atp6v0a1				117	95	79+ND+98	462	129	ND	86	5004			127	3251	83+97+94	413	213	455	151	1930	140	422	137+1495+79	995	119	4996	223	1932	382		172	3023	97		agaaaggggaaaTCATGTGAcattttcgggtc	intron2	>392
		Isca\Atp6v0a2				117	ND	79+ND+101	ND	129	ND	86	184			127	1850	83+ND+94+567+107+ND+55			ND	181	1965	140	2016	149+1374+82	ND	119	82	51+624+235+4884+111	ND	364	ND	175	ND					

Order	Species	Atp6v0a orthologs	5' exon	intron	5'exon		intron	exon	intron	exon	intron	exons/introns	intron	exon	intron	exon	intron	exon	intron	exon	intron	exon	intron	exon	intron	exon	intron	exons/introns	intron	exon	intron	exon	intron	exon3'		Extended CLEAR region	Position	bps from TSS
	Homo_sapiens	ATP6V0A1	120	1884	47	117	5417	79+1502+98	1982	129	7420	104+720+127+2077+83	2288	94	4023	213			3119	151	3696	140	560	155+437+91	3207	119	1664	217+273+108+6215+108	6233	118	310	172	6566	94				
		ATP6V0A2			248	117	5940	79+3649+98	2205	138	1405	89+1497+127+6016+83	1522	94	1434	213			6513	151	265	137	259	188+97+91	2634	119	849	211+2324+120+1053+120	2017	118	2277	172	940	106	3723			
		ATP6V0A3	104	2148	4	117	364	79+811+221			82	86+339+127+73+83	160	94	224	213			613	145	2330	140	74	158+77+91	906	119	83	214+368+126+173+105	70	118	232	178	76	79	81			

Cnidaria	*Nematostella_vectensis*	Nvec\Atp6v0a				96+89+21	159	79+77+98	2396	129	418	110+699+210	703	94	128	78	176	135	264	151	490	140	511	152+99+97	501	119	354	46+550+84+649+87+105+108+335+114	192	118	362	102+594+70	541	94	423		gcatcagacttgTCACGTGAcaagaggtggtg<br>tgctgtggtgaaTCATGTGAcaaagtgggaat<br>ttcattctcttcTCACATGAattctatttaacaa	5'<br>intron1<br>intron3	>-92<br>>274<br>>901
Placozoa	*Trichoplaz_adhaerens*	Tadh\Atp6v0a1				96	1159	79+228+98	259	129	185	116+91+127+192+83	140	94	87	78	75	135	115	151	106	140	77	140+105+88	83	119	93	46+82+84+173+87+98+117+151+126	94	118	108	172	75	106			ttgttgtgatcgTCACATGAtgtttgcgtcat<br>tgatgaacgtcaTCATGTGAacagattccata	5'<br>5'	>-440<br>>-376
		Tadh\Atp6v0a2				96+384+21	116	79+166+98	242	129	139	101+139+127+144+83	95	94	89	78	87	135	243	151	101	137	205	176+64+91	82	119	108	46+218+84+136+106+214+120+141+86	104	118	169	102+138+70	147	91			cacatgcagcaaTCATGTGAtaagaaatcaat	5'	>-160
		Tadh\Atp6v0a3				108+2785+21	449	79+453+98	129	129	215	107+94+127+95+83	77	172			86	135	116	151	87	137	155	140+92+88	132	119	146	46+81+84+73+84+75+114+75+84?	56	151	84	105+101+70	89	91					
