# Supplementary material for: Structural and Comparative Analyses of Insects Suggest the Presence of an Ultra-Conserved Regulatory Element of the Genes Encoding Vacuolar-Type ATPase Subunits and Assembly Factors
Source: Biology (Basel). 2023 Aug 13;12(8):1127. doi: 10.3390/biology12081127 (PMC10452791; doi:10.3390/biology12081127)
Supplement: Supplementary file 1 [file biology-12-01127-s001.zip › Supplementary Files S20-S37/Supp_File_S30_Atp6v0b_INS.pdf]

Gene Cluster ID	Atp6v0b
**Drosophila melanogaster gene**	**VhaPPA1-1 (CG7007)**
FlyBase ID	FBgn0028662
**Predicted function**	**ATPase, H+ transporting, lysosomal 21kDa, V0 subunit b**

Atp6v0c CLEAR element conservation:

```
        ___________5'/5'UTR_____________
Mdom\   AGCAGCACTTGATCATGTGATTAAGGCTGAAA
Ccap\   GAAATTGTTTCATCAGCTGATGCAAGGCGAAT
Ccap\   GTTGTTTTTCTGTCATGTGATGAGTAAAGTTT
Gmor\   TGCAACACTTCATCATGTGATTAAATGAAGAA
Llon\   GACTTCACACCATCACGTGAAAATTCTTCGAT
Ppap\   AGCTGACATCAATCACGTGAAAAGTGGCCGTC

Agam\   TTCTTTGCTCGGTCATATGACGTCTTGATCTT
Aara\   TTCTTTGCTCGGTCATATGACGTCTTGATCTT
Aqan\   TTCTTTGCTCGGTCATATGACGTCTTGATCTT
Achr\   TTCTTTTCTCGGTCATATGACGTCTTGATCTT
Aepi\   CTCTTTTCTTGGTCATATGACGTCTTGATCTT
Amin\   CTCTTTTCTCGGTCATATGACGTCTTGATCTT
Afun\   CTCTTTTCTCGGTCATATGACGTCTTGATCTT
Aste\   CTCTTTTCTCGGTCATATGACGTCCAGATCTT
Adir\   TCTCTTTTTCGGTCATATGACATCGAGAGATC
Anil\   TATCTTTTCTGGTCATATGACATCGGGAACAT
Aalb\   CTTCTTTGCGGGTCATATGACATCGGGATCTT
Adar\   CTTCTTTGCGGGTCATATGACATCGGGATCTT
Aaeg\   TCTCTCTGTGGGTCATATGACATCAAAGGATC
Cqui\   CAGTGAGGAGGGTCATATGATACACGGGGGAT

Mdes\   TGCGCAATCTCGTCACATGATTTTCATATCAT

Bmor\   GACATCTTGTGATCACAAGACCTCTCCGTTTT
Msex\   GACATCTTGTGATCACAAGACCTCTCCGTTTT
Hmel\   GACATCTTGTGATCACAAGACTTCTCCGTTTT
Dple\   GACGTCTTGTGATCACAAGACTTCTCGGTTTT
Pxyl\   GACATCTTGTGATCACAAGACTTCTTCGTCTA

Tcas\   gcaaggttgtacTCACGTGAgtccaattatta
Dpon\   ttttgacatttaTCATGTGAttattgtcatca

Amel\   TAAGAGTAAACGTCACATGATAAAAGCGGGTG
Aflo\   TAAGAGTAAACGTCACATGATAAAAGCGGGTG
Bter\   GAATACTAAACGTCACATGATAAAAACGGGTG
Bimp\   GAATACTAAACGTCACATGATAAAAACGGGTG
Mrot\   GATTGCTAGACGTCACATGATAGACGCGGGTG
Aech\   TACGAGCAGTCGTCACATGACAGCGACGGGTG
Pbar\   CTCGAGCAGTCGTCACATGACAGCGACGGGTG
Cflo\   AGCCAGCAGTCGTCACATGACGGCGACGGGTG
Lhum\   CGCCATCAGTCGTCACATGATATCGACGGGTG
Hsal\   AGACCTTGGTTGTCACATGATAACGACGGGTG
Acep\   GTGACATGACAGTCATATGATTTTAGAAGAGG
Sinv\   CCGAAGTGGTCGTCATATGACCACACATGACG
```

```
Ngir\  CAGAGATGGTCGTCACATGACAGCGACGGGTG
Nlon\  CAGAGATGGTCGTCACATGACAGCGACGGGTG
Nvit\  CAGAGATGGTCGTCACATGACAGCGACGGGTG

Lful\  caagtcttaaagTCACATGAttatttagagca
Phum\  ggttgctcgaagTCATATGActtctgcttaaa
Dpul\  gtgagaccaaaaTCACATGAcagatcccttcc
Isca\  gggtgtgacttgTCATGGGAcctcatctgtct
```

Atp6v0b gene structure comparisons:
(Click on orthologs for genomic, mRNA and protein sequence informations)
Key: UTR region, CDS region

Order	Species	Atp6v0b orthologs	5' exon	intron	exon3'			Extended CLEAR region	Position	bps from TSS
Diptera (Drosophilidae)	*Drosophila_melanogaster*	Dmel\VhaPPA1-1	124	59	19	639	502	agaaaatgtacgTCATGTGAttgtggattctt	5'UTR	47
Diptera (Muscidae)	*Musca_domestica*	Mdom\Atp6v0b				636		agcagcacttgaTCATGTGAttaaggctgaaa	5'UTR?	>-190
Diptera (Tephritidae)	*Ceratitis_capitata*	Ccap\Atp6v0b	26	92	22	639	159	gaaattgtttcaTCAGCTGAtgcaaggcgaat<br>gttgtttttctgTCATGTGAtgagtaaagttt	5'<br>5'	-507<br>-25
Diptera (Glossinidae)	*Glossina_morsitans*	Gmor\ATP6V0b-1<br>TMP003354*			113	636	21+70+160	tgcaacacttcaTCATGTGAttaaatgaagaa	5'	-66
		Gmor\ATP6V0b-2<br>TMP003353				648		ND		
Diptera (Psychodidae)	*Lutzomyia_longipalpis*	Llon\Atp6v0b			191	630	175	gacttcacaccaTCACGTGAaaattcttcgat	5'	-8
	Phlebotomus_papatasi	Ppap\Atp6v0b				630		agctgacatcaaTCACGTGAaaagtggccgtc	5'	>-256
Diptera (Culicidae)	*Anopheles_gambiae*	Agam\Atp6v0b<br>AGAP009334	77	83	12	627	151+75+47	ttctttgctcggTCATATGAcgtcttgatctt	5'	-1
	Anopheles_arabiensis	Aara\Atp6v0b				627		ttctttgctcggTCATATGAcgtcttgatctt	5'	>-173
	Anopheles_quadriannulatus	Aqan\Atp6v0b				627		ttctttgctcggTCATATGAcgtcttgatctt	5'	>-173
	Anopheles_christyi	Achr\Atp6v0b				627		ttcttttctcggTCATATGAcgtcttgatctt	5'	>-163
	Anopheles_epiroticus	Aepi\Atp6v0b				627		ctcttttcttggTCATATGAcgtcttgatctt	5'	>-157
	Anopheles_minimus	Amin\Atp6v0b				627		ctcttttctcggTCATATGAcgtcttgatctt	5'	>-148
	Anopheles_funestus	Afun\Atp6v0b				627		ctcttttctcggTCATATGAcgtcttgatctt	5'	>-147
	Anopheles_stephensi	Aste\Atp6v0b			10	627		ctcttttctcggTCATATGAcgtccagatctt	5'	>-152
	Anopheles_dirus A	Adir\Atp6v0b				627		tctctttttcggTCATATGAcatcgagagatc	5'	>-160
	Anopheles_nili	Anil\Atp6v0b				627		tatcttttctggTCATATGAcatcgggaacat	5'	>-157
	Anopheles_albimanus	Aalb\Atp6v0b				627		cttctttgcgggTCATATGAcatcgggatctt	5'	>-121

	Anopheles_darlingi	Adar\Atp6v0b				627												cttctttgcgggTCATATGAcatcggatctt	5'	>-119
	Aedes_aegypti	Aaeg\Atp6v0b<br>AAEL012113	54	77	15	627											55+62+179	tctctctgtgggTCATATGAcatcaaaggatc	5'	-16
	Culex_pipiens_qui.	Cqui\Atp6v0b<br>CPIJ004794				627											59+67+441	cagtgaggagggTCATATGAtacacgggggat	5'	>-155
Diptera (Cecidomyiidae)	*Mayetiola_destructor*	Mdes\Atp6v0b				624												tgcgcaatctcgTCACATGAttttcatatcat<br>attttgctgctgTCACATGAaataacatcaga	5'<br>5'	>-241<br>>-211
Lepidoptera	*Bombyx_mori*	Bmor\Atp6v0b			132	618												gacatcttgtgaTCACAAGAcctctccgtttt	5'UTR	12
	Manduca_sexta	Msex\Atp6v0b				618												gacatcttgtgaTCACAAGAcctctccgtttt	5'	>-110
	Danaus_plexippus	Dple\Atp6v0b				618												gacgtcttgtgaTCACAAGActtctcggtttt	5'	>-107
	Heliconius_melpomene	Hmel\Atp6v0b				618												gacatcttgtgaTCACAAGActtctccgtttt	5'	>-109
	Plutella_xylostella	Pxyl\Atp6v0b			93	618											31	gacatcttgtgaTCACAAGActtcttcgtcta	5'	>-14
Strepsiptera	*Mengenilla_moldrzyki*	Mmol\Atp6v0b				61	49	49	1722	84	50	148	52	243		ND		ND		
Coleoptera	*Tribolium_castaneum*	Tcas\Atp6v0b<br>TC000524	138	54	11	630												gcaaggttgtacTCACGTGAgtccaattatta	5'UTR	26
	Dendroctonus_ponderosae	Dponl\Atp6v0b			91	61	85	49	79	84	671	148	204	246	139	33	50	ttttgacatttaTCATGTGAttattgtcatca<br>cgtgatcaacagTCATATGAttccatttattt	5'UTR<br>intron1	-5<br>233
Hymenoptera	*Apis_mellifera*	Amel\Atp6vob<br>GB17836			181	61	151	49	79	84	66	397			64	33	65	taagagtaaacgTCACATGAtaaaagcgggtg	5'UTR	7
	Apis_florea	Aflo\Atp6v0b				61	151	49	83	84	62	397			63	33		taagagtaaacgTCACATGAtaaaagcgggtg	5'UTR?	>-170
	Bombus_terrestris	Bter\Atp6v0b<br>LOC100646695				61	200	49	109	84	81	394			64	33		gaatactaaacgTCACATGAtaaaaacgggtg	5'	>-173
	Bombus_impatiens	Bimp\Atp6v0b				61	201	49	117	84	97	394			64	33		gaatactaaacgTCACATGAtaaaaacgggtg	5'	>-173
	Megachile_rotundata	Mrot\Atp6v0b				61	167	49	94	84	75	397			82	33		gattgctagacgTCACATGAtagagacgggtg	5'	>-168
	Acromyrmex_echinatior	Aech\Atp6v0b				61	292	49	95	84	212	400			103	36		tacgagcagtcgTCACATGAcagcgacgggtg	5'	>-182
	Atta_cephalotes	Acep\Atp6v0b				61	246	49	95	84	220	400			100	36		gtgacatgacagTCATATGAttttagaagagg	5'	>-181
	Solenopsis_invicta	Sinv\Atp6v0b			132	73	192	49	89	84	65	400			151	36		ccgaagtggtcgTCATATGAccacacatgacg	5'	-47
	Pogonomyrmex_barbatus	Pbar\Atp6v0b				61	267	49	93	84	80	400			130	33		ctcgagcagtcgTCACATGAcagcgacgggtg	5'	>-152
	Camponotus_floridanus	Cflo\Atp6v0b				61	216	49	101	84	79	400			676	33		agccagcagtcgTCACATGAcggcgacgggtg	5'	>-159
	Linepithema_humile	Lhum\Atp6v0b				61	173	49	100	84	77	400			322	33		cgccatcagtcgTCACATGAtatcgacgggtg	5'	>-163
	Harpegnathos_saltator	Hsal\Atp6v0b				61	163	49	90	84	74	403			89	30		agaccttggttgTCACATGAtaacgacgggtg	5'	>-167
	Nasonia_vitripennis	Nvit\Atp6v0b<br>NV12316			118	67	99	49	117	84	84	400			82	36	661	cagagatggtcgTCACATGAcagcgacgggtg	5'	-52
	Nasonia_giraulti	Ngir\Atp6v0b				67	99	49	115	84	84	400			82	36		cagagatggtcgTCACATGAcagcgacgggtg	5'	>-170
	Nasonia_longicornis	Nlon\Atp6v0b				67	99	49	115	84	84	400			82	36		cagagatggtcgTCACATGAcagcgacgggtg	5'	>-172

Hemiptera	*Acyrthosiphon_pisum*	Apis\Atp6v0b ACYPI006833			113	627																			693		ND		
	Rhodnius_prolixus	Rpro\Atp6v0b				633																					ND		
Phthiraptera	*Pediculus_humanus*	Phum\Atp6v0b				211			114	410																	ggttgctcgaagTCATATGActtctgcttaaa	5'	>-78
Odonata	*Ladona_fulva*	Lful\Atp6v0b				61	995	49	87	84	1022	148			249	133?			1182	116			101	30?			aataagctctcaTCAGCTGAaatgttctatga<br>caagtcttaaagTCACATGAttatttagagca	5'<br>5'	>-394<br>>-72
**Crustacea**	*Daphnia_pulex*	Dpul\Atp6v0b			73	73	86	49	63	84	72	148(78+70)			64	130			64	116			68	30	220		gtgagaccaaaaTCACATGAcagatcccttcc	5'	-33
Ixodida	*Ixodes_scapularis*	Isca\Atp6v0b ISCW018464*			70	67	134	49	ND	84	2804	203(78+70+55)					113	191 (79+46+66)					1876	39	302		gggtgtgacttgTCATGGGAcctcatctgtct	5'	26
	Homo_sapiens	ATP6V0B			111	67	692	49	241	84	128	78	210	70	113	52	201	191 (79+46+66)					764	27	293		ggTCACGTGGtaccggcgcaTCACGTGGgc		
Cnidaria	*Nematostella_vectensis*	_v1g200737			87	79	268	49	110	84	881	78	7205	70	329	58	244	79	2104	46	169	66	73	30			taagcacaagtgTCACATGAgcaagggagaaa	5'UTR	1
Placozoa	*Trichoplax_adhaerens*	Tadh\Atp6v0b				70	229	49	879	84	412	78	112	70	87	52	109	79	111	46	74	66	ND	ND			acgctgtcatcgTCACATGAttgtactctgct<br>gaagcgtttctaTCATGTGActctcacatttt	5'<br>5'	>-759<br>>-404
