# Supplementary material for: Structural and Comparative Analyses of Insects Suggest the Presence of an Ultra-Conserved Regulatory Element of the Genes Encoding Vacuolar-Type ATPase Subunits and Assembly Factors
Source: Biology (Basel). 2023 Aug 13;12(8):1127. doi: 10.3390/biology12081127 (PMC10452791; doi:10.3390/biology12081127)
Supplement: Supplementary file 1 [file biology-12-01127-s001.zip › Supplementary Files S20-S37/Supp_File_S31_Atp6v0c_INS.pdf]

Gene Cluster ID	Atp6v0c
**Drosophila melanogaster gene**	**Vha16-1 (CG3161)**
FlyBase ID	FBgn0262736
**Predicted function**	**ATPase, H+ transporting, lysosomal 16kDa, V0 subunit c**

Atp6v0c CLEAR element conservation:

```
       ____5'_____       ____5'__          ____5'_           __intron_         __intron_         __intron_         __intron_
Ccap\  TGCTTCACGTGAGCTG  ATTTTCATATGAATCT  TTGGTCATATGACCAA  CCAGTCACATGAGTAA  CGTGTCACGTGACATT  GAAATCACATGACTGT  CTTGTCATGTGATTAA

       ___5'/5'UTR_      __intron_         __intron_         __intron_
Mdom\  TAAATCATATGACTTT  AGTTTCATGTGAACTC  TTAATCATGTGACTGA  AGTATCATGTGACCAG
Gmor\  ----------------  TAAGTCATATGACCAA  ----------------  ----------------
Llon\  GTGGTCATGTGACGAA  TCTGTCATATGACTTC  CGAATCATGTGACTCC  GAATTCATGTGATTTT
Ppap\  ATGGTCATGTGACAGC  TCTGTCATATGACCCT  GCAATCATGTGACTCC  AAATTCATGTGATTTT

       ___5'/5'UTR_      __intron_         __intron_         __intron_         __intron_         __intron_         __intron_
Agam\  CTGGTCATATGATGAT  CGTGTCATGTGAGCCC  GCTGTCACATGATCGA  TGTGTCATATGACCGA  TTGATCATGTGAACGG  ATCTTCACGTGATCGC  GTCGTCATGTGACCGC
Aara\  CTGGTCATATGATGGT  CGTGTCATGTGAGCCC  GCTGTCACATGATCGA  CGTGTCATATGACCGA  TTGATCATGTGAACGG  ATCTTCACGTGATCGC  GTCGTCATGTGACCGC
Aqan\  CTGGTCATATGATGGT  CGTGTCATGTGAGCCC  GCTGTCACATGATCGA  TGTGTCATATGACCGA  TTGATCATGTGAACGG  ATCTTCACGTGATCGC  GTCGTCATGTGACCGC
Achr\  CAGGTCATATGACGGT  CGTGTCATGTGAGCCC  GCTGTCACATGACTGA  GGAGTCATATGACCGC  ATGATCATGTGAACGC  ATCTTCACGTGATCGC  GTCGTCATGTGACCGC
Aepi\  CAGGTCATATGATGGC  CGTGTCATGTGAGCCC  GCTGTCACATGATCGG  GGAGTCATATGACCGC  TTGATCATGTGAGAAC  ATCTTCACGTGATCGC  GTCGTCATGTGACTAC
Amin\  CAGGTCATATGATGCC  CGTGTCATGTGAGCCC  TCTGTCACATGATCGG  GGAGTCATATGACCGA  ATGATCATGTGAACGC  ATCTTCACGTGATCGT  GTCGTCATGTGACCCC
Afun\  CAGGTCATATGATGGT  CGTGTCATGTGAGCCC  TCTGTCACATGATCGA  TCAGTCATATGACCGA  GTGATCATGTGAACGC  ATCTTCACGTGATCAT  GCCGTCATGTGACCCC
Aste\  CAGGTCATATGATGGT  CGTGTCATGTGAGCCC  TCTGTCACATGATCGG  GAAGTCATATGACCGA  ATGATCATGTGAACGC  ATCTTCACGTGATCGC  GTCATCATGTGACCCG
Adir\  CAGGTCATATGATAGC  GGTGTCATGTGAGCCC  TCTGTCACATGATCGA  ACCATCATCTGACCAC  GTGATCATGTGAACGC  ATCTTCACGTGATCGG  GTCATCATGTGACCTG
Anil\  CAGGTCATATGATGGT  ACTGTCATGTGAGCGC  TCTGTCACATGACCCA  AAAATCATATGACCGA  GATATCATGTGACGAG  ATCTTCACGTGATCGC  GCCATCATGTGACCCT
Aalb\  CTGGTCATATGACGCT  ATCGTCATGTGACGCC  TGGGTCACATGATGAT  ATCCTCACGTGAGGTC  GTGATCATGTGAACGC  ATCTTCACGTGATACT  ACCGTCATGTGACACA
Adar\  ----------------  ATCGTCATGTGAGCGC  TGGGTCACATGATGAT  ----------------  GTGATCATGTGAACGC  ATCTTCACGTGATACT  ATCATCATGTGACACA

Aaeg\  ACAGTCATGTGATTAC  ----------------  CGAATCATGTGACGTC  GTGTTCATGTGACATA  GCTTTCACATGACAAT  AGAGTCATGTGAGCGT  ----------------
Cqui\  CAAGTCATGTGACCAG  AACGTCATATGATCAC  TCAGTCATGTGACGTC  AATCTCACATGAGACA  CAAGTCATATGACTTG  CTGGTCATGTGAGAGT  CAAGTCATGTGATACG
Mdes\  TGTGTCATATGACCAT  ACAATCATATGAATGA  AACGTCACATGATTAA  ATGCTCATGTGATTGA  ----------------  AGATTCATGTGATCAT  ATACTCACGTGAGATA  TCTGTCATGTGAAAGA

       ___________5'/5'UTR_              __intron_         ___________intron___________________   __intron_         __intron_         __intron_
Bmor\  CAACATTCTTCGTCATATGACCCATTCTGTTG  ATTGTCATGTGATAGA  ATTGTCACATGA-CAT-TACT--TGTATCATGTGACTTG
Msex\  GAACATCAATCGTCATATGACCCATTCTATCG  GGAGTCATGTGATTGA  TTAGTCACATGA-CAG-TACA-----ATCATGTGACCAA   ataaTCATGTGAttct  caagTCACATGAttgt
Hmel\  GAACATCAATCGTCATATGACCCATTCTACCG  TCAATCATGTGATTGA  AAAATCATGTGA-CT--TCAC--TTAATCATGTGATGAA   taggTCACATGAtttt  gctgTCACATGAcaaa  tacaTCACATGAttt
Dple\  GAACATCAATCGTCATATGACCCATTCTATTG  GATATCATGTGATTGA  ATCATCACATGA-CTGATAAT--TTGGTCATGTGATTAA   tacaTCATGTGAtgta
Pxyl\  GAACATCAATCGTCATATGACCCATTCTACCG  TTAATCATGTGACTAA  TGACTCACATGATCAGCTGATGACAGGTCATGTGACCTA

       ___________5'/5'UTR_              __intron_         __intron_         __intron_         __intron_         __intron_
Tcas\  AATTTCTAATCGTCATATGACCCATTCCTGCT  GCCGTCACGTGAATTC  cgagTCATGTGAtcca  aaagTCACATGAcggt  tgtgTCATATGAccgc  caagTCACGTGAcatt
Dpon\  ATTTTCTTATCGTCATATGACCCATTCTTGCT  GGAATCAGCTGATGCT  ATGCTCATGTGATTTA  aggtTCATATGAtgaa  ctttTCACGTGActtc  gtttTCACATGActta

       ___________5'/5'UTR_              _______________________intron1____________________________
Amel\  ACTCGTCACTCGTCACATGACCCGCACTCCTG  CTTACATGATTATCATGTGACTGC------CAACGTGAGAATCATGTGACTACGATTTCA
Aflo\  ACTCGTCACTCGTCACATGACCCGCACTCCTG  CTTACATGATTATCATGTGACTGC------CAACGTGAGAATCATGTGACTACGATTTCA
Bter\  ACTCGTCACTCGTCACATGACCCGCACTCCTG  CTTACATGATTATCATGTGACTGC------CAACGTGAGAATCATGTGAC---GATTTCCA
Bimp\  ACTCGTCACTCGTCACATGACCCGCACTCCTG  CTTACATGATTATCATGTGACTGC------CAACGTGAGAATCATGTGAC---GATTTCCA
Mrot\  GGCCGTCACTCGTCACATGACCCGCACTCCTG  CTTACATGATAATCATGTGACTGC------CAACGTGAAAATCATGTGACTACGATTTCCA
Aech\  GCTCGTCACTCATCACATGACCCGCGCCACTG  CTTGTGCGATTGTCATGTGACTGC------CAACGTGAGAATCATGTGACTGCGATTTCCA
Acep\  GCTCGTCACTCATCACATGACCCGCGCCACTG  CTTGTGCGATTGTCATGTGACTGC------CAACGTGAGAATCATGTGACTGCGATTTCCA
```

```
Sinv\  GCTCGTCACTCATCACATGACCCGCGCCACTG  CTTGTGCGATTGTCATGTGACTGC------CAACGTGAGAATCATGTGACTGCAATTTCCA
Cflo\  GCTCGTCACTCATCACATGACCCGCGCCACCG  GGGACTTGATTGTCATGTGACTGC------CAACGTGAGAATCATGTGACTGCGACTTCCA
Pbar\  ACTCGTCACTCATCACATGACCCGCGCCACTG  CTTGTACGATTGTCATGTGACTGC------CAACGTGAGAATCATGTGACTGCGATTACCA
Lhum\  GCTCGTCACTCATCACATGACCCGCGCCACAG  CTTGTACGATTGTCATGTGACTGC------CAACGTGAGAATCATGTGACTGTAATTTCCA
Hsal\  GCTCGTCACTCATCACATGACCCGTGCCCGTG  CTTGAACGATTGTCATGTGACTGC------CAACGTGAGAATCATGTGACTGCGATCTCCA
Ngir\  CAGCTCGGCTCGTCACATGACCGCGCGCAGCC  TACATGCGATTGTCATGTGACTCGTGAGCGCAACGTGAGAGTCATGTGACTGGCTGTGGGG
Nlon\  CAGCTCGGCTCGTCACATGACCGCGCGCAGCC  TACATGCGATTGTCATGTGACTCGTGAGCGCAACGTGAGAGTCATGTGACTGGCTGTGGGG
Nvit\  CAGCTCGGCTCGTCACATGACCGCGCGCAGCC  TACATGCGATTGTCATGTGACTCGAGAGCGCAACGTGAGAGTCATGTGACTGGCTGTGGGG

Lful\  AATCTTCTCACATCACATGACCCTCTCTCACA

Rpro\  gctaaccctcaaTCACATGAtcatatctacct
Phum\  aatttcgattcaTCACATGAccctctgctact
Dpul\  aggtgctttcggTCACGTGAccacgagtcacg
Isca\  aaaacgctcccaTCACGTGAtataaaaataaa  cgggtcgtgcaaTCATGTGActgagattccgc  aatggggtgtttTCACATGAcgtcgatctgcc
```

Atpv0c gene structure comparisons:

(Click on orthologs for genomic, mRNA and protein sequence informations)

Key: UTR region, CDS region

Order	Species	Atp6v0c orthologs	5' exon	intron	exon		intron	exon	intron	exon3'			Extended CLEAR region	Position	bps from TSS
Diptera (Drosophilidae)	*Drosophila_melanogaster*	Dmel\Vha16-1	134		29	85	4105	190	57	205	2228		ttagatttctaaTCACGTGAgtgaaaaagcag (8 CLEAR elements)	exon/int intron1	130
Diptera (Muscidae)	*Musca_domestica*	Mdom\Atp6v0c				76	2950	190	102	205			atatgacttaaaTCATATGActttgactgcaa (4 CLEAR elements)	5' intron1	>-1056
Diptera (Tephritidae)	*Ceratitis_capitata*	Ccap\Atp6v0c	99	1115	43	82	6761	190	125	205	278		tacaaccctgctTCACGTGAgctgtcaataaa (7 CLEAR elements)	5' intron	-681
Diptera (Glossinidae)	*Glossina_morsitans*	Gmor\Atp6v0c	94	750	124	82	1722	190	68	205	180		aacttttgtaagTCATATGAccaaatgaaaaa	5'	-168
Diptera (Psychodidae)	*Lutzomyia_longipalpis*	Llon\Atp6v0c				79	8538	190	88	205	180		ctgtcttttctgTCATATGActtcgtaaaatt (7 CLEAR elements)	5'	>-289
	Phlebotomus_papatasi	Ppap\Atp6v0c				79	5505?	190	ND	205			ctgagctttctgTCATATGAccctcgtccaag (5 CLEAR elements)	5'	>-296
Diptera (Culicidae)	*Anopheles_gambiae*	Agam\atp6v0c AGAP028154			195	79	4335	190	1143	205	470		accgttcgctggTCATATGAtgataaccgcta (7 CLEAR elemnts)	5' intron1	1080
	Anopheles_arabiensis	Aara\Atp6v0c				79	4377	190	1167	205			accgttcgctggTCATATGAtggtaaccgcta (7 CLEAR elemnts)	5' intron1	>-1273
	Anopheles_quadriannulatus	Aqan\Atp6v0c				79	4346	190	1144	205			accgttcgctggTCATATGAtggtaaccgcta (7 CLEAR elemnts)	5' intron1	>-1272
	Anopheles_christyi	Achr\Atp6v0c				79	3957?	190	>483	205			acaatttgcaggTCATATGAcggtagccgcta (7 CLEAR elemnts)	5' intron1	>-1127
	Anopheles_epiroticus	Aepi\Atp6v0c				79	3936	190	740	205			caaacacgcaggTCATATGAtggccagcaaac (7 CLEAR elemnts)	5' intron1	>-1267
	Anopheles_minimus	Amin\Atp6v0c				79	3587	190	572	205			acaattcgcaggTCATATGAtgccaaagctaa (8 CLEAR elemnts)	5' intron1	>-1052
	Anopheles_funestus	Afun\Atp6v0c				79	3832	190	575	205			acaattcgcaggTCATATGAtggtaaagctaa (7 CLEAR elemnts)	5' intron1	>-1114

	Anopheles_stephensi	Aste\Atp6v0c				79	3944	190	673	205			gcgatacgcaggTCATATGAtggtgaggctag (9 CLEAR elemnts)	5' intron1	>-1269
	Anopheles_dirus	Adir\Atp6v0c				79	4584	190	901	205			gtgctgagcaggTCATGTGAtagcggtgcaaa (6 CLEAR elemnts)	5' intron1	>-1081
	Anopheles_nili	Anil\Atp6v0c				79	3459	190	843	205			tcggtacgcaggTCATATGAtggtaaagctaa (7 CLEAR elemnts)	5' intron1	>-1422
	Anopheles_albimanus	Aalb\Atp6v0c				79	3308	190	444	205			ggacagggctggTCATATGAcgctagctacgt (7 CLEAR elemnts)	intron1	>-956
	Anopheles_darlingi	Adar\Atp6v0c				79	3187	190	480	205	421		aaatcgTCATGTGAaatcgTCATGTGAgcgcaa (7 CLEAR elemnts)	intron1	>-270
	Aedes_aegypti	Aaeg\Atp6v0c AAEL000291			176	79	14503	190	7422	205	421		tttgccagacagTCATGTGAttacattcgtgt (5 CLEAR elements)	5'	-260
	Culex_pipiens_qui.	Cqui\Atp6v0c CPIJ011018			139	79	9830	190	3506	205	100		ccacacgcaagTCATGTGAccagcactcgtg (7 CLEAR elemnts)	5' intron1	-164 370
Diptera (Cecidomyiidae)	*Mayetiola_destructor*	Mdes\Atp6v0c				88	4932	190	79	205			catagccgtgtgTCATATGAccattttatatc (7 CLEAR elements)	5' intron1	>-471
Lepidoptera	*Bombyx_mori*	Bmor\Atp6v0c				73	1501	190	1221	205	275		caacattcttcgTCATATGAcccattctgttg (4 CLEAR elements)	5' intron1	>-130
	Manduca_sexta	Msex\Atp6v0c				70	2514	187	1163	208			gaacatcaatcgTCATATGAcccattctatcg (6 CLEAR elements)	5' intron1	>-129
	Danaus_plexippus	Dple\Atp6v0c				73	1511	190	221	211			gaacatcaatcgTCATATGAcccattctattg (5 CLEAR elements)	5' intron1	>-130
	Heliconius_melpomene	Hmel\Atp6v0c				73	1931	190	991	211			gaacatcaatcgTCATATGAcccattctaccg (7 CLEAR elements)	5' intron1	>-135
	Plutella_xylostella	Pxyl\Atp6v0c			66	73	1933	190	779	208	255		gaacatcaatcgTCATATGAcccattctaccg (5 CLEAR elements)	5' intron1	>-135
Strepsiptera	*Mengenilla_moldrzyki*	Mmol\Atp6v0c				477							ND		
Coleoptera	*Tribolium_castaneum*	Tcas\Atp6v0c LOC656327			99	82	2459	193	ND	205	273		aatttctaatcgTCATATGAcccattcctgct (8 CLEAR elements)	5'	-16
	Dendroctonus_ponderosae	Dpon\Atp6v0c			120	85	ND	193	678	208	217		attttcttatcgTCATATGAcccattcttgct (7 CLEAR elements)	5'	-4
Hymenoptera	*Apis_mellifera*	Amel\Atp6v0c GB19405			58	76	1840	190	556	205	53		actcgtcactcgTCACATGAcccgcactcctg (3 CLEAR elements)	5' intron1	-91
	Apis_florea	Aflo\Atp6v0c				76	1810	190	588	205			actcgtcactcgTCACATGAcccgcactcctg (3 CLEAR elements)	5' intron1	>-149
	Bombus_terrestris	Bter\Atp6v0c				85	2086	190	539	205			actcgtcactcgTCACATGAcccgcactcctg (3 CLEAR elements)	5' intron1	>-150
	Bombus_impatiens	Bimp\Atp6v0c				85	2091	190	538	205			actcgtcactcgTCACATGAcccgcactcctg (3 CLEAR elements)	5' intron1	>-150
	Megachile_rotundata	Mrot\Atp6v0c				76	2472	190	787	205			ggccgtcactcgTCACATGAcccgcactcctg (3 CLEAR elements)	5' intron1	>-150
	Acromyrmex_echinatior	Aech\Atp6a0c				79	3543	190	186	205			gctcgtcactcaTCACATGAcccgcgccactg (3 CLEAR elements)	5' intron1	>-180
	Atta_cephalotes	Acep\Atp6v0c				76	3488	190	182	205			gctcgtcactcaTCACATGAcccgcgccactg (3 CLEAR elements)	5' intron1	>-182

	Solenopsis_invicta	Sinv\Atp6v0c				79	3010	190			191	205			gctcgtcactcaTCACATGAcccgcgccactg (3 CLEAR elements)	5' intron1	>-177
	Pogonomyrmex_barbatus	Pbar\Atp6v0c				76	2937	187			256	208			actcgtcactcaTCACATGAcccgcgccactg (3 CLEAR elements)	5' intron1	>-173
	Camponotus_floridanus	Cflo\Atp6v0c				82	2659	190			224	205			gctcgtcactcaTCACATGAcccgcgccaccg (3 CLEAR elements)	5' intron1	>-176
	Linepithema_humile	Lhum\Atp6v0c				79	2561	190			166	205			gctcgtcactcaTCACATGAcccgcgccacag (3 CLEAR elements)	5' intron1	>-173
	Harpegnathos_saltator	Hsal\Atp6v0c				82	2639	187			446	205			gctcgtcactcaTCACATGAcccgtgcccgtg (3 CLEAR elements)	5' intron1	>-174
	Nasonia_vitripennis	Nvit\Atp6v0c			254	88	2051	190			88	211	447		cagctcggctcgTCACATGAccgcgcgcagcc (3 CLEAR elements)	5' intron1	-64
	Nasonia_giraulti	Ngir\Atp6v0c				88	ND	190			88	211			cagctcggctcgTCACATGAccgcgcgcagcc (3 CLEAR elements)	5' intron1	>-334
	Nasonia_longicornis	Nlon\Atp6v0c				88	ND	190			88	211			cagctcggctcgTCACATGAccgcgcgcagcc (3 CLEAR elements)	5' intron1	>-331
Hemiptera	*Acyrthosiphon_pisum*	Apis\Atp6v0c			376	82	2440	190			109	205			ND		
Hemiptera	*Rhodnius_prolixus*	Rpro\Atp6a0c				79	2505	190			1608	205			gctaaccctcaaTCACATGAtcatatctacct	5'	>-152
Phthiraptera	*Pediculus_humanus*	Phum\Atp6a0c				ND	ND	184			105	205			aatttcgattcaTCACATGAccctctgctact	5'	>-139
Odonata	*Ladona_fulva*	Lful\Atp6v0c				82	372	190			1279	205			aatcttctcacaTCACATGAccctctctcaca	5'	>-116
**Crustacea**	*Daphnia_pulex*	Dpul\Atp6a0c			72	91	332	190			59	205	181		aggtgctttcggTCACGTGAccacgagtcacg	5'	-79
Ixodida	*Ixodes_scapularis*	Isca\Atp6v0c-1 ISCW018346			152	76	2174	184			1782	208	1126		aaaacgctcccaTCACGTGAtataaaaataaa (3 CLEAR elements)	5' intron1	-26
Ixodida	*Ixodes_scapularis*	Isca\Atp6v0c-2 ISCW012283				471									ND		
	Homo_sapiens	ATP6V0C			234	79	5035	184			139	205	478		gcggggcccaggTCATGTGAcgcggccgcggc	5'UTR	53
Cnidaria	*Nematostella_vectensis*	v1g162114			92	76	74	159	1262	25	612	211	826		ND		
Placozoa	*Trichoplax_adhaerens*	Tadh\Atp6v0c			98	79	211	159	141	28	904	211	47		ND		
