# Supplementary material for: Structural and Comparative Analyses of Insects Suggest the Presence of an Ultra-Conserved Regulatory Element of the Genes Encoding Vacuolar-Type ATPase Subunits and Assembly Factors
Source: Biology (Basel). 2023 Aug 13;12(8):1127. doi: 10.3390/biology12081127 (PMC10452791; doi:10.3390/biology12081127)
Supplement: Supplementary file 1 [file biology-12-01127-s001.zip › Supplementary Files S20-S37/Supp_File_S32_Atp6v0d_INS.pdf]

Gene Cluster ID	Atp6v0d
**Drosophila melanogaster gene**	**VhaAC39-1 (CG2934)**
FlyBase ID	FBgn0028665
**Predicted function**	**ATPase, H+ transporting, lysosomal 38kDa, V0 subunit d**

Atp6v0d CLEAR element conservation:

```
               5'/5'UTR                          intron1                          intron1
Dmel\   AAGAGTTGAAAATCATATGACTTGTCGATCGA  CACGCCCCTGGGTCACATGACCAGCGATAACA
Mdom\   CAACTGCTATAGTCATATGACAGTCGATAAAG  TACAGGGGCAAGTCATGTGACATTTGTATGTG
Ccap\   TACCAATGACAGTCATGTGATGACTTATGACA  GGGTGGTGTTGGTCATGTGACACACCATTAAA
Gmor\   ACTCTGAGAGAGTCATATGACAGTAAGAATGA  ACAAGGGCCTGGTCATGTGATGCGTACTATGT  TAGTAATAATTTTCATATGAATGTATGTATGA
Ppap\   CTCAACTACGGGTCATATGACATTTTGATAAT  TTGAGAAATTATTCACGTGATCTTGATTAACC  --------------------------------
Llon\   --------------------------------  TTTTTGATTTATTCACATGATTCACAGGTTTC  AAACTCTTGGAATCATATGACCACGGAAAATT

Agam\   AAAACTAGGTAGTCATATGATGCGTTTTTGCC  GTGTAAAGGCGGTCATATGACCAGCCTTCAGC
Aara\   AAAACTAGGTAGTCATATGATGCGTTTTTGCC  GTGTAAAGGCGGTCATATGACCAGCCATCAGC
Aqan\   AAAACTAGGCAGTCATATGATGCGTTTTTGCC  GTGTAAAGGCGGTCATATGACCAGCCATCAGC
Achr\   AAAAATAGGTAGTCATATGATGCGTTTTTGCC  AGTAAAGGCGGGTCATATGACCTGCCTTCAAA
Aepi\   GCAAAATTGTAGTCATATGATGCGTTTTTGCC  GGTATTGACGGGTCATATGACCTGCCTTCGGT
Amin\   ACACAATTGTAGTCATATGATGCGTTTTAGCT  ACGGCAGGCGGGTCATATGACCTGCTCAATAC
Afun\   CAAAAATTGTAGTCATATGATGCGTTTTACCT  ACGGCAGGCAGGTCATATGACCTGCTCAACAC
Aste\   ACAAAATTGTAGTCATATGATACATTTTGCCC  CGGGTAGGCGGGTCATATGACCTACTCAACCG
Adir\                                     ACATAAGGCAGGTCATGTGACCCCTTTGCATG
Aalb\   AAGACTGAGTAGTCATATGATGCACATTCTGC  TCACCCACCGGGTCATATGACCGATCTTTTCA
Adar\   AGTCAGGGGTAGTCATATGATGCGCTTTCAGC  TCACATACTGGGTCATATGACCCGTCTTTTCA
Aaeg\   TCCGAGTGCGAGTCATATGACACTTTTCAGAG  CATCTTGGTTGATCATATGACCTTATCACACG
Cqui\   CCCATGCGCGAGTCATATGACACTTTTCAGAG  CGATTTGGTTGATCATATGACCTTATCATATA

Mdes\   TTCGCATTGTTATCACATGAATTGTCAATGTT  TGTTGTTGTTATTCATATGATGAAAAAGTGAC

Bmor\   TCTATGTTCACATCATATGATCAATTTTCTTT
Msex\   TCTATGATCGCATCATATGATCAATGCAACTC
Dple\   TCTATGGAGCTATCATATGATCAATGATCTAC
Hmel\   TCTATGGAGTTATCATATGATCAATGATCTAT
Pxyl\   TCTATGGTCAAATCATATGATCAATGAATGAT

Tcas\   ctgTCACATGAttgtcactaaTCATATGAcaa
Dpon\   caaTCACATGAt..gtcaaaaTCATATGAcgc

Amel\   CTCACAAGATAGTCATATGATCGACACGAAAA
Aflo\   CTCATAAGATAGTCATATGATCGACACGAAAA
Bter\   CTCGCTAGATGGTCATATGATCGACACGAAAA
Bimp\   CTCGCTAGATGGTCATATGATCGACACGAAAA
Mrot\   CTCACTGTACGGTCATATGATCGACACGAAAA
Ngir\   GTTAGACTTTAGTCATATGATCGACTCCTGAC
Nvit\   GTTAGACTTTAGTCATATGATCGACTCCTGAC
Nlon\   GTTAGACTTTAGTCATATGATCGACTCCTGAC
Aech\   ATGACATGACAGTCATATGATTTTAGAAGAGG
Acep\   GTGACATGACAGTCATATGATTTTAGAAGAGG
Sinv\   CGGCTAGCTCGGTCATATGATTTTGTGGAAAA
```

Pbar\ CTGCTCGTTTAGTCATATGATTTTGAGAAAGG
Cflo\ AGTCCATATTGGTCATATGATTTTCACGAAGG
Lhum\ GGTCATATGCTGTCATATGATCTTCGTGAAAA
Hsal\ CCGTGCGCTCGGTCATATGATCTCGGTGGAAA
Lful\ CACATCCTCGAGTCATATGATCTGCTATCCCT

Rpro\ cagactgaattgTCATATGAatacaccctgcc
Phum\ tcattaatgtatTCATATGAatcattaaaaat
Phum\ cactcttctctgTCATATGAcaacagattggg
Dpul\ ccgtcccaagtgTCATGTGAttctgatccgtc
Isca\ taactgccggagTCATATGAtcaatacggccc
Hsap\ gccccttatggTCACGTGAggcgcttgcgcg
Nvec\ ttcctgcgccggTCATGTGAttcttacatgta
Tadh\ gtatctcgttgcTCACATGAtcctcttgtgag

Atp6v0d gene structure comparisons:
(Click on orthologs for genomic, mRNA and protein sequence informations)
Key: UTR region, CDS region

Order	Species	Atp6v0d orthologs	5'exon		intron	exon	intron	exon3'		Extended CLEAR region	Position	bps from TSS
Diptera (Drosophilidae)	*Drosophila_melanogaster*	Dmel\Vha39-1	249	127	338	509	90	417	502	aagagttgaaaaTCATATGActtgtcgatcga<br>cacgcccctgggTCACATGAccagcgataaca	5'UTR<br>intron1	47<br>437
Diptera (Muscidae)	*Musca_domestica*	Mdom\Vha39-1		127	357	509	948	417		caactgctatagTCATATGAcagtcgataaag<br>tacaggggcaagTCATGTGAcatttgtatgtg	5'UTR?<br>intron1	>-213<br>>223
Diptera (Tephritidae)	*Ceratitis_capitata*	Ccap\Vha39-1	197	124	292	509	70	417	138	taccaatgacagTCATGTGAtgacttatgaca<br>gggtggtgttggTCATGTGAcacaccattaaa	5'UTR?<br>intron1	-33<br>425
Diptera (Glossinidae)	*Glossina_morsitans*	Gmor\Vha39-1	199	127	213	509	58	417	158	actctgagagagTCATATGAcagtaagaatga<br>acaagggcctggTCATGTGAtgcgtactatgt<br>tagtaataatttTCATATGAatgtatgtatga	5'UTR<br>intron1<br>intron1	39<br>382<br>419
Diptera (Psychodidae)	*Lutzomyia_longipalpis*	Llon\Atp6v0d	94	121	418	764 (509+255)	67	162	333	tttttgatttatTCACATGAttcacaggtttc<br>aaactcttggaaTCATATGAccacggaaaatt	5'<br>5'UTR	-617<br>12
	Phlebotomus_papatasi	Ppap\Atp6v0d		121	3295	764 (509+255)	ND	162		ctcaactacgggTCATATGAcattttgataat<br>ttgagaaattatTCACGTGAtcttgattaacc	5'<br>intron1	>-93<br>>552
-												
Diptera (Culicidae)	*Anopheles_gambiae*	Agam\Atp6v0d<br>AGAP000721	370	121	517	509	1037	417	219	aaaactaggtagTCATATGAtgcgtttttgcc<br>gtgtaaaggcggTCATATGAccagccttcagc<br>gcaaaatgTCATGTGAaTCACGTGAtagtcacc	5'UTR<br>intron1<br>intron2	5<br>799<br>2033
	Anopheles_arabiensis	Aara\Atp6v0d-1		121	500	509	1314	417		aaaactaggtagTCATATGAtgcgtttttgcc<br>gtgtaaaggcggTCATATGAccagccatcagc<br>gcgaaatgTCATGTGAaTCACGTGAtaatcacc	5'UTR?<br>intron1<br>intron2	>-368<br>>438<br>>1603
	Anopheles_quadriannulatus	Aqan\Atp6v0d-1		121	511	509	1305	417		aaaactaggcagTCATATGAtgcgtttttgcc<br>gtgtaaaggcggTCATATGAccagccatcagc<br>gcaaaatgTCATGTGAaTCACGTGAtaatcacc	5'UTR<br>intron1<br>intron2	>-368<br>>451<br>>1617
	Anopheles_christyi	Achr\Atp6v0d-1		121	495	509	450	417		aaaaataggtagTCATATGAtgcgtttttgcc<br>agtaaaggcgggTCATATGAcctgccttcaaa	5'UTR<br>intron1	>-317<br>>421
	Anopheles_epiroticus	Aepi\Atp6v0d-1		121	1287	509	792	417		gcaaaattgtagTCATATGAtgcgtttttgcc<br>ggtattgacgggTCATATGAcctgccttcggt	5'UTR<br>intron1	>-326<br>>445

	Anopheles_minimus	Amin\Atp6v0d-1		121	365	509	72	417					acacaattgtagTCATATGAtgcgttttagct<br>acggcaggcgggTCATATGAcctgctcaatac	5'UTR<br>intron1	>-370<br>>309
	Anopheles_funestus	Afun\Atp6v0d-1		121	418	509	77	417					caaaaattgtagTCATATGAtgcgttttacct<br>acggcaggcaggTCATATGAcctgctcaacac	5'UTR<br>intron1	>-378<br>>304
	Anopheles_stephensi	Aste\Atp6v0d-1		121	413	509	79	>392					acaaaattgtagTCATATGAtacattttgccc<br>cgggtaggcgggTCATATGAcctactcaaccg	5'UTR<br>intron1	>-436<br>>372
	Anopheles_dirus	Adir\Atp6v0d-1		121	314	509	62	417					acataaggcaggTCATGTGAcccctttgcatg	5'UTR?<br>intron1	>293
	Anopheles_nili	Anil\Atp6v0d-1													
	Anopheles_albimanus	Aalb\Atp6v0d-1		121	467	509	73	417					aagactgagtagTCATATGAtgcacattctgc<br>tcacccaccgggTCATATGAccgatcttttca	5'UTR?<br>intron1	>-254<br>>385
	Anopheles_darlingi	Adar\Atp6v0d-1		121	633	509	92	417			421		agtcaggggtagTCATATGAtgcgctttcagc<br>tcacatactgggTCATATGAcccgtcttttca	5'UTR?<br>intron1	>-244<br>>450
	Aedes_aegypti	Aaeg\Atp6v0d<br>AAEL011025*	143	121	ND	509	337	417 (255 + 162)			445		tccgagtgcgagTCATATGAcacttttcagag<br>catcttggttgaTCATATGAccttatcacacg	5'<br>intron1	-20<br>409
	Culex_pipiens_qui.	Cqui\Atp6v0d<br>CPIJ009947		121	ND	926 (509+417)					110		cccatgcgcgagTCATATGAcacttttcagag<br>cgatttggttgaTCATATGAccttatcatata	5'<br>intron1	>-154<br>>255
	Anopheles_gambiae	Agam\Atp6v0d-2<br>AGAP013199		1044									ND		
	Anopheles_arabiensis	Aara\Atp6v0d-2		1044									ND		
	Anopheles_quadriannulatus	Aqan\Atp6v0d-2		1044									ND		
	Anopheles_christyi	Achr\Atp6v0d-2		1044									ND		
	Anopheles_epiroticus	Aepi\Atp6v0d-2		1044									ND		
	Anopheles_minimus	Amin\Atp6v0d-2		1044									ND		
	Anopheles_funestus	Afun\Atp6v0d-2		1044									ND		
	Anopheles_stephensi	Aste\Atp6v0d-2		1044									ND		
	Anopheles_dirus	Adir\Atp6v0d-2		1044									ND		
	Anopheles_nili	Anil\Atp6v0d-2		1044									ND		
	Anopheles_albimanus	Aalb\Atp6v0d-2		1068									ND		
	Anopheles_darlingi	Adar\Atp6v0d-2		1071									ND		
-															
Diptera (Cecidomyiidae)	*Mayetiola_destructor*	Mdes\Atp6v0d		121	332	509	76	255	81	162			ttcgcattgttaTCACATGAattgtcaatgtt<br>tgttgttgttatTCATATGAtgaaaaagtgac	5'<br>intron1	>-171<br>>310
Lepidoptera	*Bombyx_mori*	Bmor\Atp6v0d		121	786	509	213	255	262	162			tctatgttcacaTCATATGAtcaattttcttt	5'	>-117
	Manduca_sexta	Msex\Atp6v0d		121	272	509	75	255	335	162			tctatgatcgcaTCATATGAtcaatgcaactc	5'	>-126
	Danaus_plexippus	Dple\Atp6v0d	110	121	176	509	86	255	82	162	59		tctatggagctaTCATATGAtcaatgatctac	5'UTR	5
	Heliconius_melpomene	Hmel\Atp6v0d		121	157	509	408	255	332	162			tctatggagttaTCATATGAtcaatgatctat	5'	>-97

	Plutella_xylostella	Pxyl\Atp6v0d	129	121	102	509					78	255			141	162	334		tctatggtcaaaTCATATGAtcaatgaatgat	5'UTR	26
Strepsiptera	*Mengenilla_moldrzyki*	Mmol\Atp6v0d		121	ND	509?					ND	408							ND		
Coleoptera	*Tribolium_castaneum*	Tcas\Atp6v0d<br>LOC663777	73	121	50	509 (351+158)					43	255(177+178)			54	162	192		TCACATGAttgtcactaaTCATATGAcaatttagttg	5'/5'UTR	-4/6
	Dendroctonus_ponderosae	Dpon\Atp6v0d	73	121	801	926 (351+158+177+78+162)											267		tgcacatgacaaTCACATGAtctgccaaggat<br>ggattgtcaaaaTCATATGAcgctcgggcgag	5'<br>5'UTR	-9<br>13
Hymenoptera	*Apis_mellifera*	Amel\Atp6v0d<br>GB12698	112	121	121	351			125	158	94	177	155	78	102	162	411		ctcacaagatagTCATATGAtcgacacgaaaa	5'UTR	20
	Apis_florea	Aflo\Atp6v0d		121	126	351			119	158	115	177	133	78	97	162			ctcataagatagTCATATGAtcgacacgaaaa	5'	>-84
	Bombus_terrestris	Bter\Atp6v0d		121	126	351			129	158	209	177	118	78	76	162			ctcgctagatggTCATATGAtcgacacgaaaa	5'	>-84
	Bombus_impatiens	Bimp\Atp6v0d		121	126	351			142	158	209	177	118	78	77	162			ctcgctagatggTCATATGAtcgacacgaaaa	5'	>-84
	Megachile_rotundata	Mrot\Atp6v0d		121	90	351			123	158	77	177	79	78	77	162			ctcactgtacggTCATATGAtcgacacgaaaa	5'	>-92
	Acromyrmex_echinatior	Aech\Atp6v0d		121	258	351			145	158	76	177	1075	78	190	162			atgacatgacagTCATATGAttttagaagagg	5'	>-81
	Atta_cephalotes	Acep\Atp6v0d		121	249	351			132	158	73	177	1121	78	ND	162			gtgacatgacagTCATATGAttttagaagagg	5'	>-81
	Solenopsis_invicta	Sinv\Atp6v0d	76	121	389	351			2226	158	281	177	ND	78	136	162			cggctagctcggTCATATGAttttgtggaaaa	5'	>-86
	Camponotus_floridanus	Cflo\Atp6v0d		121	789	351			524	158	86	177	141	78	140	162			agtccatattggTCATATGAttttcacgaagg	5'	>-105
	Harpegnathos_saltator	Hsal\Atp6v0d		121	242	351			310	158	76	177	119	78	110	162			ccgtgcgctcggTCATATGAtctcggtggaaa	5'	>-95
	Pogonomyrmex_barbatus	Pbar\Atp6v0d		121	263	351			117	158	67	177	135	78	163	162			ctgctcgtttagTCATATGAttttgagaaagg	5'	>-84
	Linepithema_humile	Lhum\Atp6v0d		121	239	351			102	158	102	255(177+178)			110	162			ggtcatatgctgTCATATGAtcttcgtgaaaa	5'	>-81
	Nasonia_vitripennis	Nvit\Atp6v0d<br>NV13431		121	82	351			90	158	80	177	89	78	89	162	433		gttagactttagTCATATGAtcgactcctgac	5'UTR?	>-151
	Nasonia_giraulti	Ngir\Atp6v0d		121	82	351			90	158	88	177	88	78	89	162			gttagactttagTCATATGAtcgactcctgac	5'UTR?	>-151
	Nasonia_longicornis	Nlon\Atp6v0d		121	82	351(172+179)			90	158	80	177	88	78	89	162			gttagactttagTCATATGAtcgactcctgac	5'UTR?	>-151
Hemiptera	*Acyrthosiphon_pisum*	Apis\Atp6v0d<br>ACYPI003170	197+16	786									85	270							ND		
	Rhodnius_prolixus	Rpro\Atp6v0d		121	116	172	1506	179	81	158	143	177	520	78	233	162			cagactgaattgTCATATGAatacaccctgcc	5'	>-740
Phthiraptera	*Pediculus_humanus*	Phum\Atp6v0d<br>PHUM410380		121	100	172	67	179	177	158	90	177	80	78	86	>144			tcattaatgtatTCATATGAatcattaaaaat<br>cactcttctctgTCATATGAcaacagattggg	5'<br>5'	>-228<br>>-71
Odonata	*Ladona_fulva*	Lful\Atp6v0d		121	72	172	79	179	111	158	477	177	1429	78	84	162			cacatcctcgagTCATATGAtctgctatccct	5'	>-95
**Crustacea**	*Daphnia_pulex*	Dpul\Atp6v0d		121	64	172	76	337 (179 + 158)			65	177	59	78	60	162	167		ccgtcccaagtgTCATGTGAttctgatccgtc	5'	>-108
Ixodida	*Ixodes_scapularis*	Isca\Atp6v0d<br>ISCW002101	53	121	ND	172	3991	179	127	158(80+78)	814	177	74	78	1886	162			taactgccggagTCATATGAtcaatacggccc	5'UTR	29

	Homo_sapiens	ATP6V0D1	100	130	27241	172	8837	179	1349	80	3747	78	126	177	100	78	103	162			gcccccttatggTCACGTGAggcgcttgcgcg	5'	-23
Cnidaria	*Nematostella_vectensis*	v1g128006		130	2206	172	456	179	880	80	357	78	513	177	1100	78	385	162			ttcctgcgccggTCATGTGAttcttacatgta	5'	>-81
Placozoa	*Trichoplax_adhaerens*	TRIADDRAFT_37067		127	287	172	126	179	122	80	211	78	144	177	79	78	79	162	712		atctcgttgcTCACATGAtcctcttgtgagtc	5'	>-324
