# Supplementary material for: Structural and Comparative Analyses of Insects Suggest the Presence of an Ultra-Conserved Regulatory Element of the Genes Encoding Vacuolar-Type ATPase Subunits and Assembly Factors
Source: Biology (Basel). 2023 Aug 13;12(8):1127. doi: 10.3390/biology12081127 (PMC10452791; doi:10.3390/biology12081127)
Supplement: Supplementary file 1 [file biology-12-01127-s001.zip › Supplementary Files S20-S37/Supp_File_S33_Atp6v0e_INS.pdf]

Gene Cluster ID	Atp6v0e
**Drosophila melanogaster gene**	**VhaM9.7-b (CG7625)**
FlyBase ID	FBgn0028663
**Predicted function**	**ATPase, H+ transporting, V0 subunit e**

Atp6v0e CLEAR element conservation:

```
                 5'/5'UTR                            5'/5'UTR                              intron2
Dmel\  GGGACATGTCCGTCATGTGATATTCGTTGCTC
Mdom\  cattatatttaaTCATGTGAcacacgacgaca
Ccap\  tcccatgggtgaTCATGTGAcatttgcgtagc
Gmor\  gctctttacgcaTCATGTGAtaattagtaatt
Llon\  aacgatttcgggGCACATGAcgtgtgacgtca
Ppap\  gaaacaaaatagTCATATGAttaatcgaccga

Agam\  --------------------------------  CCTGTACGCCTGTCATGTGATATTTTTCTCAT
Aara\  --------------------------------  CCTGTACGCCTGTCATGTGATATTTTTCTCAT
Aqan\  --------------------------------  CCTGTACGCCTGTCATGTGATATTTTTCTCAT
Achr\  --------------------------------  TGCTTGTGACTGTCATGTGATATTTTTCTCTT
Aepi\  --------------------------------  TGTTCTGCTCTGTCATGTGATATTTTTCTCTT
Amin\  --------------------------------  TGTTCTGGTCTGTCATGTGATATTTTTCTCTT
Afun\  --------------------------------  TGTTCTGGTCTGTCATGTGATATTTTTCTCTT
Aste\  --------------------------------  GTCCGTGGTCTGTCATGTGATATTTTTCTCTT
Adir\  --------------------------------  TGTTCTTGTCTGTCATGTGATATTTTTCTGCC
Anil\  --------------------------------  TGGTACAAGCTGTCATGTGATGTTTTTCTCTG
Aalb\  --------------------------------  ACGTCACTGCTGTCATGTGATGTTTTCTCTTC
Adar\  --------------------------------  ACGTCACGGCTGTCATGTGATGTTTTCTCTTC
Cqui\  GCGTTTGAAACGTCACATGATGAGTGACGCGT  CACCCGAACCTGTCATGTGATATTTTTCTCAC
Aaeg\  CCACCAAGGACGTCACATGAGTGACTCACTCA  CACCGAGACCTGTCATGTGATTTATTATTTTCT
Mdes\  --------------------------------  GCGCGCTCACTGTCATGTGATTGTCATGAGAA

Bmor\  TATCTATGACAGTCATATGACTCTCGGTGACA
Msex\  --------------------------------
Hmel\  ATCCGATAATTGTCATATGACTACGACATTCA
Dple\  CCGATTCTCATGTCATATGACTGCCAATGTTC
Pxyl\  ATTCTACGAATGTCATATGACTTAAAACAGTG

Tcas\  GTCCTGTGCGCATCATATGACTTTGACACTTC                                    GTTCAATCGGAATCACATGACAACAAACACTA
Dpon\  TCCCCTACCTAGTCATATGATTTTGACGTCTA                                    TTCTGTTACTAGTCATGTGATTAAATTATGGG

Amel\  AAATCGAAATATTCACGTGATTCACATTTACT  --------------------------------
Aflo\  AAATCGAGATATTCACGTGATTCACGATTACT  --------------------------------
Bter\  AAATCGAGATATTCACGTGACTCTCATTTACA  --------------------------------
Bimp\  AAATCGAGATATTCACGTGACTCCCATTTACA  --------------------------------
```

```
Mrot\  GAACTGAGCGCTTCACGTGATCTGTTTGTGTC  --------------------------------
Aech\  CAATCGATGTG-TCACGTGAATAGTACAAAGA  ACAAAGAATCTCTCATGTGATCCTTTCGATAC
Acep\  CAATCGATGTG-TCACGTGAATAGTACAAAGA  ACAAAGAATCTCTCATGTGATCCTTTCAATAC
Cflo\  TAATCGATGTA-TCACGTGAACAATACGAAGA  ACGAAGATTCTCTCATGTGATCCTCCCAGTTT
Pbar\  AGATCAATGTG-TCACGTGAACAGTACGAAGA  ACGAAGATTCTCTCATGTGATCCTTCCAGTTT
Sinv\  TGATCGATGTG-TCACGTGAATGGTACGAGGA  ACGAGGAGTCTCTCATGTGATTCTTTCGTTAT
Lhum\  GAATCGATATG-TCACGTGAATCAGGCGAAGG  GCGAAGGTACTCTCATGTGATCCTCCCAGTTT
Hsal\  GAATCGATATA-TCACGTGAAGATCTCTCATG  CGTGAAGATCTCTCATGTGATCCTTCCCGTTT

Ngir\  --------------------------------  TGCGCATGTTCATCATGTGACAGAAAGCAGAC
Nlon\  --------------------------------  TGCGCATGTTCATCATGTGACAGAAAGCAGAC
Nvit\  --------------------------------  TGCGCATGTTCATCATGTGACAGAAAGCAGAC

Lful\  --------------------------------  tttctagtaataTCATGTGAtccgttcgcaga  tgtaattatcgtTCATATGAaagttttaatta

Phum\  aaatatttttgaaTCACATGAtttcgtctgctt
Dpul\  caaagttagctgTCACATGAcacaagatatct
```

Atp6v0e gene structure comparisons:

(Click on orthologs for genomic, mRNA and protein sequence informations)

Key: UTR region, CDS region

Order	Species	Atp6v0e orthologs	5'exon		intron	exon	intron	exon3'		Extended CLEAR region	Position	bps from TSS
Diptera (Drosophilidae)	*Drosophila_melanogaster*	Dmel\VhaM9.7-b	142	95	70	48	68	127	303	gggacatgtccgTCATGTGAtattcgttgctc	5'UTR	38
Diptera (Muscidae)	*Musca_domestica*	Mdom\Atp6v0e		95	96	48	74	130		cattatatttaaTCATGTGAcacacgacgaca	5'UTR?	>-144
Diptera (Tephritidae)	*Ceratitis_capitata*	Ccap\Atp6v0e	134	95	81	48	61	130	363	tcccatgggtgaTCATGTGAcatttgcgtagc	5'UTR?	-24
Diptera (Glossinidae)	*Glossina_morsitans*	Gmor\Atp6v0e TMP002992		95	57	48	77	145		gctctttacgcaTCATGTGAtaattagtaatt	5'UTR?	>-83
Diptera (Psychodidae)	*Lutzomyia_longipalpis*	Llon\Atp6v0e	116	98	90	48	66	148		aacgatttcgggGCACATGAcgtgtgacgtca	5'UTR	22
	Phlebotomus_papatasi	Ppap\Atp6v0e	26	98	71	48	59	145		gaaacaaaatagTCATATGAttaatcgaccga<br>aattcttcacctTCACGTGAtttttcttacag	5'<br>intron1	>-32<br>>176
Diptera (Culicidae)	*Anopheles_gambiae*	Agam\Atp6v0e AGAP003588	129	98	91	48	76	142	440	cctgtacgcctgTCATGTGAtatttttctcat	5'UTR	6
	Anopheles_arabiensis	Aara\Atp6v0e		98	85	48	77	142		cctgtacgcctgTCATGTGAtatttttctcat	5'UTR?	>-123
	Anopheles_quadriannulatus	Aqan\Atp6v0e		98	86	48	76	142		cctgtacgcctgTCATGTGAtatttttctcat	5'UTR?	>-124

	Anopheles_christyi	Achr\Atp6v0e		98	64	48	77	142				tgcttgtgactgTCATGTGAtatttttctctt	5'UTR?	>-123
	Anopheles_epiroticus	Aepi\Atp6v0e		98	74	48	78	142				tgttctgctctgTCATGTGAtatttttctctt	5'UTR?	>-105
	Anopheles_minimus	Amin\Atp6v0e		98	78	48	90	142				tgttctggtctgTCATGTGAtatttttctctt	5'UTR?	>-112
	Anopheles_funestus	Afun\Atp6v0e		98	74	48	87	142				tgttctggtctgTCATGTGAtatttttctctt	5'UTR?	>-110
	Anopheles_stephensi	Aste\Atp6v0e		98	79	48	75	142				gtccgtggtctgTCATGTGAtatttttctctt	5'UTR?	>-119
	Anopheles_dirus	Adir\Atp6v0e		98	70	48	80	145				tgttcttgtctgTCATGTGAtatttttctgcc	5'UTR?	>-120
	Anopheles_nili	Anil\Atp6v0e		98	74	48	74	142				tggtacaagctgTCATGTGAtgtttttctctg	5'UTR?	>-119
	Anopheles_albimanus	Aalb\Atp6v0e		98	91	48	91	136				acgtcactgctgTCATGTGAtgttttctcttc	5'UTR?	>-116
	Anopheles_darlingi	Adar\Atp6v0e		98	86	48	85	133				acgtcacggctgTCATGTGAtgttttctcttc	5'UTR?	>-122
	Aedes_aegypti	Aaeg\Atp6v0e<br>AAEL010819	48+82+5	98	70	48	4734	151			674	ccaccaaggacgTCACATGAgtgactcactca<br>caccgagacctgTCATGTGAttattattttct	5'<br>5'UTR	-26<br>4
	Culex_pipiens_qui.	Cqui\Atp6v0e<br>CPIJ013144		98	91	48	71	148			422	gcgtttgaaacgTCACATGAtgagtgacgcgt<br>cacccgaacctgTCATGTGAtatttttctcac	5'<br>5'	>-129<br>>-173
Diptera (Cecidomyiidae)	*Mayetiola_destructor*	Mdes\Atp6v0e		92	82	48	90	145				gcgcgctcactgTCATGTGAttgtcatgagaa	5'UTR?	>-164
Lepidoptera	*Bombyx_mori*	Bmor\Atp6v0e	84	98	83	48	1579	88	ND	30	449	tatctatgacagTCATATGActctcggtgaca	5'	>-26
	Manduca_sexta	Msex\Atp6v0e		ND	ND	ND	ND	88	7377	33	351	ND		
	Danaus_plexippus	Dple\Atp6v0e		98	68	48	476	88	ND	ND		ccgattctcatgTCATATGActgccaatgttc	5'	>-109
	Heliconius_melpomene	Hmel\Atp6v0e		98	74	48	873	88	ND	ND		atccgataattgTCATATGActacgacattca	5'	>-102
	Plutella_xylostella	Pxyl\Atp6v0e	35	98	88	48	965	88	3949	24		attctacgaatgTCATATGActtaaaacagtg<br>tagttaatgtgcTCACATGAtggtcataagtc	5'<br>intron2	-67<br>447
Strepsiptera	*Mengenilla_moldrzyki*	Mmol\Atp6v0e		98	45	48	49	88	57	21		ND		
Coleoptera	*Tribolium_castaneum*	Tcas\Atp6v0e<br>LOC662403	79	98	61	48	46	88	53	18	140	gtcctgtgcgcaTCATATGActttgacacttc<br>gttcaatcggaaTCACATGAcaacaaacacta	5'<br>intron2	-17<br>322
	Dendroctonus_ponderosae	Dpon\Atp6v0e	81	98	61	48	678	88	133	27	272	tcgttgaatcgaTCACGTGAatgacaactccc<br>tcccctacctagTCATATGAttttgacgtcta<br>ttctgttactagTCATGTGAttaaattatggg	5'<br>5'<br>intron2	-27<br>-6<br>326
Hymenoptera	*Apis_mellifera*	Amel\Atp6v0e<br>GB18948	103	98	154	48	86	88	105	36	585	aaatcgaaatatTCACGTGAttcacatttact	5'UTR	20
	Apis_florea	Aflo\Atp6v0e		98	151	48	77	88	101	42		aaatcgagatatTCACGTGAttcacgattact	5'	>-74

	Bombus_terrestris	Bter\Atp6v0e	192	98	128	48	81	88	107	24	375		aaatcgagatatTCACGTGActctcatttaca	5'UTR	100
	Bombus_impatiens	Bimp\Atp6v0e		98	123	48	80	88	103	24			aaatcgagatatTCACGTGActcccatttaca	5'UTR?	>-84
	Megachile_rotundata	Mrot\Atp6v0e		98	173	48	74	88	94	21			gaactgagcgctTCACGTGAtctgtttgtgtc	5'	>-82
	Acromyrmex_echinatior	Aech\Atp6v0e		107	261	48	76	88	181	18			tcaatcgatgtgTCACGTGAatagtacaaaga<br>acaaagaatctcTCATGTGAtcctttcgatac	5'<br>5'	>-121<br>>-96
	Atta_cephalotes	Acep\Atp6v0e		107	268	48	82	88	169	21			tcaatcgatgtgTCACGTGAatagtacaaaga<br>acaaagaatctcTCATGTGAtcctttcaatac	5'<br>5'	>-113<br>>-88
	Solenopsis_invicta	Sinv\Atp6v0e	122	107	254	48	82	88	285	21	139		gtgatcgatgtgTCACGTGAatggtacgagga<br>acgaggagtctcTCATGTGAttctttcgttat	5'<br>5'UTR	-14<br>13
	Camponotus_floridanus	Cflo\Atp6v0e		107	244	48	84	88	64	21			ttaatcgatgtaTCACGTGAacaatacgaaga<br>acgaagattctcTCATGTGAtcctcccagttt	5'<br>5'	>-132<br>>-107
	Harpegnathos_saltator	Hsal\Atp6v0e		98	283	48	81	88	161	24			cgaatcgatataTCACGTGAagatctcTCATGTGAtccttcccgttt	5'	>-108
	Pogonomyrmex_barbatus	Pbar\Atp6v0e		107	474	48	83	88	165	21			cagatcaatgtgTCACGTGAacagtacgaaga<br>acgaagattctcTCATGTGAtccttccagttt	5'<br>5'	>-131<br>>-106
	Linepithema_humile	Lhum\Atp6v0e		107	261	48	87	88	161	21			cgaatcgatatgTCACGTGAatcaggcgaagg<br>gcgaaggtactcTCATGTGAtcctcccagttt	5'<br>5'	>-136<br>>-111
	Nasonia_giraulti	Ngir\Atp6v0e		98	99	48	76	88	222	33			tgcgcatgttcaTCATGTGAcagaaagcagac	5'	>-83
	Nasonia_longicornis	Nlon\Atp6v0e		98	99	48	76	88	221	33			tgcgcatgttcaTCATGTGAcagaaagcagac	5'	>-83
	Nasonia_vitripennis	Nvit\Atp6v0e<br>NV10516	25	98	99	48	73	88	223	33			tgcgcatgttcaTCATGTGAcagaaagcagac	5'	>-59
Hemiptera	*Acyrthosiphon_pisum*	Apis\Atp6v0e<br>LOC100571036	440	98	67	48	2880	88	65	18			tactaccacgTCATATGAttgccgtcaa	intron 2	2344
	Rhodnius_prolixus	Rpro\Atp6v0e		98	71	48	140	88	1483	30			ND		
Phthiraptera	*Pediculus_humanus*	Phum1Atp6v0e<br>PHUM238330		98	70	48	124	94					aaatattttgaaTCACATGAtttcgtctgctt	5'	>-99
Odonata	*Ladona_fulva*	Lful\Atp6v0e		98	69	48	286	88	63	33			tttctagtaataTCATGTGAtccgttcgcaga<br>tgtaattatcgtTCATATGAaagttttaatta	5'<br>intron2	>-49<br>>260
**Crustacea**	*Daphnia_pulex*	Dpul\Atp6v0e	107	98	70	48	210	109			108		caaagttagctgTCACATGAcacaagatatct	5'	-1
Aracnida	*Ixodes_scapularis*	Isca\Atp6v0e<br>ISCW018653		98	57	48	63	94					ND		
	Homo_sapiens	ATP6V0E1		104	10810	48	24415	94			36				
Cnidaria	*Nematostella_vectensis*	NEMVEDRAFT_v1g192225	18	113	318	48	1144	106			17				
Placozoa	*Trichoplax_adhaerens*	TRIADDRAFT_63123	16	98	989	48	148	106			15				
