# Supplementary material for: Structural and Comparative Analyses of Insects Suggest the Presence of an Ultra-Conserved Regulatory Element of the Genes Encoding Vacuolar-Type ATPase Subunits and Assembly Factors
Source: Biology (Basel). 2023 Aug 13;12(8):1127. doi: 10.3390/biology12081127 (PMC10452791; doi:10.3390/biology12081127)
Supplement: Supplementary file 1 [file biology-12-01127-s001.zip › Supplementary Files S20-S37/Supp_File_S34_Atp6ap1_INS.pdf]

Gene Cluster ID	Atp6ap1
**Drosophila melanogaster gene**	**VhaAC45 (CG8029)**
FlyBase ID	[FBgn0262515]()
**Predicted function**	**ATPase, H+ transporting, lysosomal accessory protein 1**

Atp6ap1 CLEAR element conservation:

```
               5'/5'UTR
Dmel\   AGCTGGTGCGCATCACATGACCGTCATAAGGT
Mdom\   ATCGCTCTGTCATCACATGACTGATTCAAGAT

Ccap\   AATTCGAATATATCACATGACTGTCATGAGCA

Gmor\   TATTTTTTCTAATCACATGACAGTCATACGCG
Llon\   GATTCATCGCAATCACATGACAAACATCGAAA
Ppap\   AAAAACATCGAATCATATGACACAGAGAAATC

               5'/5'UTR                          intron1                            exon2
Agam\   GTTCGTAAACA-GTCAGGTGACAAAAAAAGTG  --------------------------------  GATGACTTTGCGTCACATGATGCGCTCATCGA
Aqan\   GTTCGTAAACA-GTCAGGTGACAAAAAAAGTG  --------------------------------  GATGACTTTGCGTCACATGATGCTCTCATCGA
Aara\   GTTCGTAAACA-GTCAGGTGACAAAAAAAAGT  --------------------------------  GATGACTTTGCGTCACATGATGCTCTCATCGA
Achr\   GTTCGTAAACA-GTCAGGTGATAGAAAAAAAC  --------------------------------  GGTGACTTTGCGTCACATGATGCGCTCATCGA
Aepi\   GTTCGTAAACA-GTCAGGTGACAGAGAAAGGG  --------------------------------  GATGACTTTGCGTCACATGACGCCTTGATCGA
Amin\   TTTCGTAAACA-GTCAGGTGATAGCTAGAGCG  --------------------------------  GGTGATTTTGCGTCACATGACGCCATCATCGA
Aste\   TTTCGTAAACA-GTCAGGTGATAGCCCACATT  --------------------------------  GGTGATTTTGCATCACATGACGCCATCATCGA
Afun\   TTTCGTAAACA-GTCAGGTGATTAGCAAAAGC  --------------------------------  GGTGATTTCGCGTCACATGATGCCGTCATCGA
Adir\   TTTCGTAAACAAGTCAGGTGATAGGAAGAAAA  --------------------------------  GGTGACTTTGCATCACATGACACCCTCATCGG
Anil\   CTGCTGACCGTGGTCAGGTGATTCGTCTCGCG  TTGCTGCAAGTTTCACATGACGACGTGCTCGA  GGTGACTTTGCGTCACATGATGCGCTCATCGA
Aalb\   --------------------------------  --------------------------------  GGCGATTACGCATCACATGACGCTCTGATCGA
Adar\   --------------------------------  --------------------------------  GGCGATTACGCATCACATGATGCCCTGATCGA
Aaeg\   AATCGAATCGTATCACGTGAAGGAAGCAAGTG  GGCGGGACGAGGTCATGTGATGTGCTTTACGT  AATGATTTTGCGTCACATGATGCGCTTATCGA
Cqui\   ATCGTCATTGCGTCAGCTGAGTCAGTCACGAG  GGAGTGAGTGGGTCATGTGATGTTGTTTTTC   GACGACTTTGCGTCACATGACGCTTTGATCGA

Mdes\   gcattgtcaaaaTCACTTGActtttgggcccc

                      5'/5'UTR
Bmor\   CGTCAATCGTAATCATATGATCACTTTTCACATGACCGATGGTCACT
Dple\   CGTCAATCGTAATCATATGATCACTTTTCACATGACCGATGGTCACT
Hmel\   GCGTCAAAGTAATCATATGATCACTTTTCACATGACCAATGGTTCTT
Msex\   GCGTCAAAGTAATCATATGATCATTTTTCACATGACCGATGGTTAGT
Pxyl\   CAAGCGTCAAAATCATATGATCATTTTTCACATGACCCCTGTCAAAT

               5'/5'UTR                         intron1
Tcas\   tactcccaaaatTCACGTGAtcttgtaaaagt  gggcatctgaagTCATATGActtggtttcagg

                     5'/5'UTR
Amel\   GAAACAATGTTATCACGTGACGTTTGTCATATGATTCTAGTCTAGG
Aflo\   GAAACAATGTTATCACGTGACGTTTGTCATATGATTCTAGTCTAGG
Bter\   GAAACAAAGTTATCACGTGACGTCTGTCATATGATTCTAGCCTAGT
Bimp\   GAAACAAAGTTATCACGTGACGTCTGTCATATGATTCTAGCCTAGT
```

```
Mrot\   GAAAGGAAGTTGTCACGTGACGTCTGTCATATGATTCTAGTCAGGT
Hsal\   GAAATGTACCAATCACGTGATATATATCATATGATCGCGAGAAGAT
Lhum\   GAAGTGCATCGGCCACGTGACATGTGTCATATGATCGCGGCAGTC
Cflo\   GAAATGCATCGACCATGTGACTCACATCATATGATCACGAGGTGGT
Aech\   GAAGTGCATCGGCCACGTGATGCGAATCAGCGTCACGAGGTAATAG
Acep\   GAAGTGCATCGGTCACGTGATGCGAATCGGCGTCACGAGGTAACAG
Sinv\   GAAGTGTATCGGCCACGTGACACACGAGTCGGCGTCACGACTGTCA
Pbar\   GAAATCAGCCCGCCACGTGATACGAGACGTCACACGATCACGAGGG

                     5'/5'UTR
Phum\   ttcctttgaacaTCATGTGAcaatttacttct
Lful\   cgattgtactgaTCACATGAttgcatgtgatg
Dpul\   gcaccgtctgtgTCATGTGAtgaaatttctg
```

Atp6ap1 gene structure comparisons:

(Click on orthologs for genomic, mRNA and protein sequence informations)

Key: UTR region, CDS region

Order	Species	Atp6ap1 orthologs	5'exon		intron	exon	intron	exon	intron	exon3'		Extended CLEAR region	Position	bps from TSS
Diptera (Drosophilidae)	*Drosophila_melanogaster*	Dmel\VhaAC45	203	86	63	103	66	262	72	689	559	agctggtgcgcaTCACATGAccgtcataaggt	5'	-4
Diptera (Muscidae)	*Musca_domestica*	Mdom\Atp6ap1		86	69	103	343	265	63	698		atcgctctgtcaTCACATGActgattcaagat<br>actgattcaagaTCATATGAatacgaagaaat	5'<br>5'	>-230<br>>-211
Diptera (Tephritidae)	*Ceratitis_capitata*	Ccap\Atp6ap1		80	58	103	66	271	63	710	250	aattcgaatataTCACATGActgtcatgagca	5'	>-187
Diptera (Glossinidae)	*Glossina_morsitans*	Gmor\Atp6ap1	124	89	53	103	56	265	58	698	475	tattttttctaaTCACATGAcagtcatacgcg	5'	-57
Diptera (Psychodidae)	*Lutzomyia_longipalpis*	Llon\Atp6ap1	111	98	72	1087						gattcatcgcaaTCACATGAcaaacatcgaaa	5'	-4
	Phlebotomus_papatasi	Ppap\Atp6ap1	48	98	55	1087					132	aaaaacatcgaaTCATATGAcacagagaaatc	5'	-59
Diptera (Culicidae)	*Anopheles_gambiae*	Agam\Atp6ap1<br>AGAP003879	117	101	67	1066					161	gttcgtaaacagTCAGGTGAcaaaaaaagtgc<br>gatgactttgcgTCACATGAtgcgctcatcga	5'UTR<br>exon2	16<br>626
	Anopheles_arabiensis	Aara\Atp6ap1		101	67	1066						gttcgtaaacagTCAGGTGAcaaaaaaaagtg<br>gatgactttgcgTCACATGAtgctctcatcga	5'<br>exon2	>-96<br>>509
	Anopheles_quadriannulatus	Aqan\Atp6ap1		101	67	1066						gttcgtaaacagTCAGGTGAcaaaaaaagtgc<br>gatgactttgcgTCACATGAtgctctcatcga	5'<br>exon2	>-91<br>>509
	Anopheles_christyi	Achr\Atp6ap1		101	85	1066						gttcgtaaacagTCAGGTGAtagaaaaaaacg<br>ggtgactttgcgTCACATGAtgcgctcatcga	5'<br>exon2	>-96<br>>527
	Anopheles_epiroticus	Aepi\Atp6ap1		104	111	1060						gttcgtaaacagTCAGGTGAcagagaaagggc<br>gatgactttgcgTCACATGAcgccttgatcga	5'<br>exon2	>-95<br>>556
	Anopheles_minimus	Amin\Atp6ap1		104	72	1066						tacgtaccagttTCATATGAttttgtattttt<br>tttcgtaaacagTCAGGTGAtagctagagcga	5'<br>5'	>-524<br>>-91

												ggtgattttgcgTCACATGAcgccatcatga	exon2	>517
	Anopheles_funestus	Afun\Atp6ap1		104	74	1066						tttcgtaaacagTCAGGTGAttagcaaaagcg<br>ggtgatttcgcgTCACATGAtgccgtcatcga	5'<br>exon2	>-90<br>>519
	Anopheles_stephensi	Aste\Atp6ap1	51	104	67	1063					115	tttcgtaaacagTCAGGTGAtagcccacattc<br>ggtgattttgcaTCACATGAcgccatcatcga	5'<br>exon2	-42<br>586
	Anopheles_dirus	Adir\Atp6ap1		101	94	1060						ttcgtaaacaagTCAGGTGAtaggaagaaaaa<br>ggtgactttgcaTCACATGAcaccctcatcgg	5'<br>exon2	>-91<br>>536
	Anopheles_nili	Anil\Atp6ap1		101	82	1072						tgctgaccgtggTCAGGTGAttcgtctcgcga<br>ttgctgcaagttTCACATGAcgacgtgctcga<br>ggtgactttgcgTCACATGAtgcgctcatcga	5'<br>intron1<br>exon2	>-94<br>>145<br>>524
	Anopheles_albimanus	Aalb\Atp6ap1		98	79	1093						ggcgattacgcaTCACATGAcgctctgatcga	exon2	>518
	Anopheles_darlingi	Adar\Atp6ap1		98	78	1093						ggcgattacgcaTCACATGAtgccctgatcga	exon2	>517
	Aedes_aegypti	Aaeg\Atp6ap1<br>AAEL007777	164	92	127	109	324	966			139	aatcgaatcgtaTCACGTGAaggaagcaagtg<br>ggcgggacgaggTCATGTGAtgtgctttacgt<br>aatgattttgcgTCACATGAtgcgcttatcga	5'UTR<br>intron1<br>exon2	135<br>335<br>1054
	Culex_pipiens_qui.	Cpip\Atp6ap1		110	59	1066						atcgtcattgcgTCAGCTGAgtcagtcacgag<br>ggagtgagtgggTCATGTGAtgtttgttttc<br>gacgactttgcgTCACATGAcgctttgatcga	5'<br>intron1<br>exon2	>-130<br>>144<br>>516
Diptera (Cecidomyiidae)	*Mayetiola_destructor*	Mdes\Atp6ap1		1224								gcattgtcaaaaTCACTTGActtttgggcccc	5'	>-110
Lepidoptera	*Bombyx_mori*	Bmor\Atp6ap1	132	826	2037	164	955	219			99	cgtcaatcgtaaTCATATGAtcactttTCACATGAccgatggtcact	5UTR?	-15
	Manduca_sexta	Msex\Atp6ap1	77	808	1403	161	347	219				gcgtcaaagtaaTCATATGAtcattttTCACATGAccgatggttagt	5'	-24
	Danaus_plexippus	Dple\Atp6ap1		817	897	167	139	219				cgtcaatcgtaaTCATATGAtcactttTCACATGAccgatggtcact	5'	>-101
	Heliconius_melpomene	Hmel\Atp6ap1		817	522	155	323	219				gcgtcaaagtaaTCATATGAtcactttTCACATGAccaatggttctt	5'	>-109
	Plutella_xylostella	Pxyl\Atp6ap1	86	691	ND	138	610	128	ND	213		caagcgtcaaaaTCATATGAtcattttTCACATGAcccctgtcaaat	5'	>-111
Coleoptera	*Tribolium_castaneum*	Tcas\Atp6ap1<br>LOC663029		17+156+112+50+271+49+86+51+263+53+193	45	207					273	tactcccaaaatTCACGTGAtcttgtaaaagt<br>gggcatctgaagTCATATGActtggtttcagg	5'<br>intron1	36<br>323
Hymenoptera	*Apis_mellifera*	Amel\Atp6ap1<br>GB16678	138	273	87	351	80	549			372	gaaacaatgttaTCACGTGAcgtttgTCATATGAttctagtctagg	5'UTR	40/54
	Apis_florea	Aflo\Atp6ap1		273	87	351	74	558				gaaacaatgttaTCACGTGAcgtttgTCATATGAttctagtctagg	5'	>-89
	Bombus_terrestris	Bter\Atp6ap1<br>LOC100646562		267	87	357	87	573				gaaacaaagttaTCACGTGAcgtctgTCATATGAttctagcctagt	5'	>-98
	Bombus_impatiens	Bimp\Atp6ap1		267	87	357	87	573				gaaacaaagttaTCACGTGAcgtctgTCATATGAttctagcctagt	5'	>-98
	Megachile_rotundata	Mrot\Atp6ap1		270	84	360	67	552				gaaaggaagttgTCACGTGAcgtctgTCATATGAttctagtcaggt	5'	>-98
	Acromyrmex_echinatior	Aech\Atp6ap1		276	1148	291	204	513				gaagtgcatcggCCACGTGAtgcgaatcagcg	5'	>-130
	Atta_cephalotes	Acep\Atp6ap1		285	1104	303	203	525				gaagtgcatcggTCACGTGAtgcgaatcggcg	5'	>-130

	Solenopsis_invicta	Sinv\Atp6ap1		279			498	303	391	528					gaagtgtatcggCCACGTGAcacacgagtcgg	5'	>-131
	Camponotus_floridanus	Cflo\Atp6ap1		282			130	306	202	534					attaaagattatTCATGTGActtttataacaa<br>atgtgactcacaTCATATGAtcacgaggtggt	5'<br>5'	>-193<br>>-107
	Harpegnathos_saltator	Hsal\Atp6ap1		285			414	309	217	528					gaaatgtaccaaTCACGTGAtatataTCATATGAtcgcgagaagat	5'	>-100
	Pogonomyrmex_barbatus	Pbar\Atp6ap1		279			493	303	102	528					gaaatcagcccgCCACGTGAtacgagacgtca	5'	>-117
	Linepithema_humile	Lhum\Atp6ap1		288			345	306	120	528					gaagtgcatcggCCACGTGAcatgtgTCATATGAtcgcgggcagtc	5'	>-93
	Nasonia_vitripennis																
	Nasonia_giraulti																
	Nasonia_longicornis																
Odonata	*Ladona_fulva*	Lful\Atp6ap1		960					375	198	75	225			cgattgtactgaTCACATGAttgcatgtgatg	5'UTR?	>-10
Hemiptera	*Acyrthosiphon_pisum*	Apis\Atp6ap1<br>LOC100169264	146	241			1030	518	60	267	ND	213			ccttgacgatTCACGTGAatcattgctt	exon3	2010
	Rhodnius_prolixus											210					
Phthiraptera	*Pediculus_humanus*	Phum\Atp6ap1<br>PHUM457150		238	95	318	76	200	74	195	114	210			ttcctttgaacaTCATGTGAcaatttacttct	5'	>-113
**Crustacea**	*Daphnia_pulex*	Dpul\Atp6ap1		104	104	106	60	265	63	86	58	176+63+153+84+288+71+169			gcaccgtctgtgTCATGTGAtgaaaatttctg	5'	>-79
Aracnida	*Ixodes_scapularis*																
