# Supplementary material for: Structural and Comparative Analyses of Insects Suggest the Presence of an Ultra-Conserved Regulatory Element of the Genes Encoding Vacuolar-Type ATPase Subunits and Assembly Factors
Source: Biology (Basel). 2023 Aug 13;12(8):1127. doi: 10.3390/biology12081127 (PMC10452791; doi:10.3390/biology12081127)
Supplement: Supplementary file 1 [file biology-12-01127-s001.zip › Supplementary Files S20-S37/Supp_File_S35_Atp6ap2_INS.pdf]

Gene Cluster ID	Atp6ap2
**Drosophila melanogaster gene**	**VhaM8.9 (CG8444)**
FlyBase ID	FBgn0037671
**Predicted function**	**ATPase, H+ transporting, lysosomal accessory protein 2**

Atp6v1H CLEAR element conservation:

```
                5'/5'UTR                         5'/5'UTR
Dmel\   CAACCCTGTTCATCACATGATAGTCAGGGTGA   TGCAGTCGTTCGTCATATGATTAAAAGTCCAG
Mdom\   TCTCTCTTTACATCATGTGACCAAATGCAACC   GTTTGTCGATTGTCATATGATCACGAACAAAA
                5'/5'UTR
Ccap\   CCGTCGCAGTTGTCATATGATCAATTTTCATT
Gmor\   TCTAGCCGTTTGTCATATGATCAATTTTTGAT
Llon\   AGACACCTTTGGTCATATGATTTTAGTGTGAG
Ppap\   CTTGGGCGATTGTCATATGATTTTACTGTGAA

                5'/5'UTR                         5'/5'UTR                         intron1
Agam\   CTGCGCAAAGTGTCACGTGAGGAACGGGACGG   ACCACAATAACGTCATATGATCACAAGTGCAA   TCTCATCG-CTGTTCACATGACATGAAGTATC
Aara\   CTGCGCAAAGTGTCACGTGAGGAACGGGACGG   ACCACAATAACGTCATATGATCACAAGTGCAA   TCTCATCG-CTTTTCACATGACATGAAGTATC
Aqan\   CTGCGCAAAGTGTCACGTGAGGAACGGGACGG   ACCACAATAACGTCATATGATCACAAGTGCAA   TCTCATCG-CTGTTCACATGACATGAAGTATC
Achr\   CTGCGCAAAGTGTCACGTGAGGAACGGAGTCG   ACCACAATAACGTCATATGATCACAAGTGCAA   TCCCTGTG-CAGTTCACATGACATAAAAATAC
Amin\   CTGCGCAAAGTGTCACGTGAGGAACGGAATGG   AACACAATAACGTCATATGATCACAAGTGCAA   TCTCATCGTCTGTTCACATGACAACGAAAAGG
Afun\   CTGCACAAAGTGTCACGTGAGGAACGGAATGG   AACACAATAACGTCATATGATCACAAGTGCAA   --------------------------------
Aste\   CTGCGCAAAGTGTCACGTGAGGAACGCAGTGG   AACACAATAACGTCATATGATCACAAGTGCAA   --------------------------------
Aepi\   CTGCAGTAAGTGTCACGTGAGGAACAAGGTGG   ACCACAATAACGTCATATGATCACAAGTGCAA   --------------------------------
Adir\   --------------------------------   AACAGAAAAAAGTCATATGACCAACTCGGTCA   --------------------------------
Anil\   --------------------------------   ACGACAAAAAACGTCATATGATCACAAGTGCAA   --------------------------------
Aalb\   CCCGTCCAAACGTCACGTGAGGCGCTCCAAAC   GCGCTCCAAACGTCATATGATCAAAATTCAGA   --------------------------------
Adar\   CCCGTTCAAACGTCACGTGAGGCGCTCCAAAC   GCGCTCCAAACGTCATATGATCAAAAATCAGA   --------------------------------
Aaeg\   AAACTCGAAAAGTCACGTGAGGGACCATATCG   AAATCGGGAACGTCATATGATCACTGGTGCAA   --------------------------------
Cqui\   --------------------------------   AAAGTGAAAACGTCATATGATCACTGGGTGCA   --------------------------------

Mdes\   TGTGCTGTGATGTCACGTGAATGTTTCGAAAC   AATCATAGTACATCATATGATCTGGCTTTAGT

Bmor\   GTATGAATTTAATCATATGACATTGTGATTGT
Dple\   GTAACACTTTAATCATATGACAGTAAGCCACC
Hmel\   GCAATACTTTAATCATATGACAGTCAGCTACT
Msex\   GTAAGACTTTAATCATATGACAGTCAGTCATT
Pxyl\   GCAACACTGAAATCATATGACAGTCTGCCATT
                5'/5'UTR                         5'/5'UTR
Tcas\   GTCACAAGACTGTCATATGATTATGACATTTG   GTTAGTTTAATGTCATATGACTTCAAATTTCC
                5'/5'UTR
Amel\   ATTACATTAATGTCATATGATCAGTTTGTGAA
Aflo\   ATTACGTTAATGTCATATGATCAGTTTGTGAA
Bter\   ACTCCTTTGTGGTCATATGATCAGTTTGTGAA
Bimp\   ACTCCTTTGTGGTCATATGATCAGTTTGTGAA
Mrot\   TGGATTAGCGTGTCATATGATAAACTTATAGA
Nvit\   GTGCAAAGGTGTCATATGATCGGACGAAAAA
Nlon\   GTGCAAAGGTGTCATATGATCGGACGAAAAA
Ngir\   GTGCAAAGGTGTCATATGATCGGACGAAAAA
```

```
Aech\   CATCTGACATGATCACATGATCACGTCCGAAA
Lhum\   CATCTGATGTGATCACATGATCGCGTCCGAAA
Sinv\   TACCTGACATGATCATATGATCGCGTCTGAAA
Pbar\   TGTCTGACATAATCACATGATCGCGTCTGAAA
Cflo\   GATCTGAAGTTATCACATGATCATGTCCGAAA
Hsal\   GACCACAGGTGATCACATGATCATGTCCGAAT
Acep\   TATCTGACATGATCACATGATCCGAAATTGAT

Lful\   catatcgtctggTCATATGAttcttaagcgaa
Dpul\   agcacgaacacaTCACCTGActttagctgtca
```

Atp6ap2 gene structure comparisons:

(Click on Species for genomic, mRNA and protein sequence informations)

Key: UTR region, CDS region

Order	Species	Atp6ap2 orthologs	5'exon		intron	exon			intron	exon3'							Extended CLEAR region	Position	bps from TSS
Diptera (Drosophilidae)	*Drosophila_melanogaster*	Dmel\VhaM8.9	84	43	223	353			262	567					421		caaccctgttcaTCACATGAtagtcagggtga<br>tgcagtcgttcgTCATATGAttaaaagtccag	5'<br>5'UTR	-1<br>52
Diptera (Muscidae)	*Musca_domestica*	Mdom\Atp6ap2		43	260	731							747	183			tctctctttacaTCATGTGAccaaatgcaacc<br>gtttgtcgattgTCATATGAtcacgaacaaaa	5'<br>5'	>-93<br>>-13
Diptera (Tephritidae)	*Ceratitis_capitata*	Ccap\Atp6ap2		43	218	353			64	-	-	249	56	186	285		ccgtcgcagttgTCATATGAtcaattttcatt	5'UTR?	>-37
Diptera (Glossinidae)	*Glossina_morsitans*	Gmor\Atp6ap2	60	43	149	356			1905	378			60	186	236		tctagccgtttgTCATATGAtcaatttttgat	5'	-3
Diptera (Psychodidae)	*Lutzomyia_longipalpis*	Llon\Atp6ap2	52	43	141	341			58	138	59	246	433	189	246		agacacctttggTCATATGAttttagtgtgag	5'UTR	3
	Phlebotomus_papatasi	Ppap\Atp6ap2	56	43[-]?	ND	344			58	138	55	246	ND	189	226		cttgggcgattgTCATATGAttttactgtgaa	5'UTR	14
Diptera (Culicidae)	*Anopheles_gambiae*	Agam\Atp6ap2<br>AGAP003430		43	214	224	81	519					80	189	584		ctgcgcaaagtgTCACGTGAggaacgggacgg<br>accacaataacgTCATATGAtcacaagtgcaa<br>tctcatcgctgtTCACATGAcatgaagtatcg	5'<br>5UTR?<br>intron1	-35<br>18<br>278
	Anopheles_arabiensis	Aara\Atp6ap2		43	214	224	87	519					80	189			ctgcgcaaagtgTCACGTGAggaacgggacgg<br>accacaataacgTCATATGAtcacaagtgcaa<br>tctcatcgctttTCACATGAcatgaagtatcg	5'<br>5UTR?<br>intron1	>-108<br>>-47<br>>206
	Anopheles_quadriannulatus	Aqan\Atp6ap2		43	214	224	81	519					80	189			ctgcgcaaagtgTCACGTGAggaacgggacgg<br>accacaataacgTCATATGAtcacaagtgcaa<br>tctcatcgctgtTCACATGAcatgaagtatcg	5'<br>5UTR?<br>intron1	>-108<br>>-47<br>>206
	Anopheles_christyi	Achr\Atp6ap2		43	217	224	83	513					76	189			ctgcgcaaagtgTCACGTGAggaacggagtcg<br>accacaataacgTCATATGAtcacaagtgcaa<br>tccctgtgcagtTCACATGAcataaaaatacg	5'<br>5UTR?<br>intron1	>-109<br>>-47<br>>211
	Anopheles_epiroticus	Aepi\Atp6ap2		43	234	224	75	516					64	189			ctgcagtaagtgTCACGTGAggaacaaggtgg<br>accacaataacgTCATATGAtcacaagtgcaa<br>ctcatcgtctgtTCACATGAcaacgaaaagga	5'<br>5UTR?<br>intron1	>-108<br>>-47<br>>218
	Anopheles_minimus	Amin\Atp6ap2		43	196	224	59	513					74	189			ctgcgcaaagtgTCACGTGAggaacggaatgg<br>aacacaataacgTCATATGAtcacaagtgcaa	5'<br>5UTR?	>-108<br>>-47

	Anopheles_funestus	Afun\Atp6ap2		43	216	224			65	516							79	189					ctgcacaaagtgTCACGTGAggaacggaatgg<br>aacacaataacgTCATATGAtcacaagtgcaa	5'<br>5UTR?	>-107<br>>-47
	Anopheles_stephensi	Aste\Atp6ap2		43	217	224			62	516							71	189					ctgcgcaaagtgTCACGTGAggaacgcagtgg<br>aacacaataacgTCATATGAtcacaagtgcaa	5'<br>5UTR?	>-107<br>>-47
	Anopheles_dirus A	Adir\Atp6ap2		43	237	224			73	516							78	180					aacagaaaaaagTCATATGAccaactcggtca<br>tgaccaactcggTCATATGAcaagtgcaaaaa	5'<br>5UTR?	>-67<br>>-50
	Anopheles_nili	Anil\Atp6ap2		43	212	224			65	513							82	189					acgacaaaaacgTCATATGAtcacaagtgcaa	5UTR?	>-50
	Anopheles_albimanus	Aalb\Atp6ap2		43	185	224			67	513							74	180					cccgtccaaacgTCACGTGAggcgctccaaac<br>gcgctccaaacgTCATATGAtcaaaattcaga	5'<br>5UTR?	>-63<br>>-42
	Anopheles_darlingi	Adar\Atp6ap2		43	189	224			60	513?							72	180					cccgttcaaacgTCACGTGAggcgctccaaac<br>gcgctccaaacgTCATATGAtcaaaaatcaga	5'<br>5UTR?	
	Aedes_aegypti	Aaeg\Atp6ap2<br>AAEL002411		43	197	224			74	114	16650	399					63	180			421		aaactcgaaaagTCACGTGAgggaccatatcg<br>aaatcgggaacgTCATATGAtcactggtgcaa	5'<br>5UTR	-73<br>15
	Culex_pipiens_qui.	Cqui\Atp6ap2<br>CPIJ009739		43	205	224			80	519							66	180					aaagtgaaaacgTCATATGAtcactgggtgca	5UTR?	-30
Diptera (Cecidomyiidae)	*Mayetiola_destructor*	Mdes\Atp6ap2	54	43	170	350					95	363					104	207					tgtgctgtgatgTCACGTGAatgtttcgaaac<br>aatcatagtacaTCATATGAtctggctttagt	5'<br>5UTR	24<br>-64
Lepidoptera	*Bombyx_mori*	Bmor\Atp6ap2		52	77	131	96	99	165	144	91	141	827	130	422	140	1453	177			275		gtatgaatttaaTCATATGAcattgtgattgt	5UTR?	-15
	Manduca_sexta	Msex\Atp6ap2		52	80	131	142	99	321	144	248	141	78	130	360	140	216	174					gtaagactttaaTCATATGAcagtcagtcatt	5UTR?	>-27
	Danaus_plexippus	Dple\Atp6ap2		52	70	131	126	99	178	144	88	141	174	130	353	137	441	174					gtaacactttaaTCATATGAcagtaagccacc	5UTR?	>-26
	Heliconius_melpomene	Hmel\Atp6ap2		52	71	131	83	99	60	144	77	144	105	130	2632	137	705	174					gcaatactttaaTCATATGAcagtcagctact	5UTR?	>-37
	Plutella _xylostella	Pxyl\Atp6ap2	15	52	94	131	126	99	334	144	387	135	511	130	378	131	1417	180			497		gcaacactgaaaTCATATGAcagtctgccatt	5UTR?	>-11
Coleoptera	*Tribolium_castaneum*	LOC662403		43	43	365					46	135	1537	285			50	180			273		gtcacaagactgTCATATGAttatgacatttg<br>gttagtttaatgTCATATGActtcaaatttcc	5'<br>5UTR	-20<br>14
Hymenoptera	*Apis_mellifera*	GB17311		43	117	128	175	231			380	315			100	93	7467	103	82	80			attacattaatgTCATATGAtcagtttgtgaa	5UTR?	1
	Apis_florea	Aflo\Atp6ap2		43	108	128	180	231			253	315			111	93	7469	103	82	80			attacgttaatgTCATATGAtcagtttgtgaa	5UTR?	>-78
	Bombus_terrestris	Bter\Atp6ap2		43	101	128	180	231			117	315			111	93	4720	112	90	80			actcctttgtggTCATATGAtcagtttgtgaa	5UTR?	-7
	Bombus_impatiens	Bimp\Atp6ap2		43	101	128	178	231			117	315			111	93	4731	112	90	80			actcctttgtggTCATATGAtcagtttgtgaa	5UTR?	>-69
	Megachile_rotundata	Mrot\Atp6ap2		43	111	128	128	231			135	315			71	105	3446	106	74	80			tggattagcgtgTCATATGAtaaacttataga	5UTR?	
	Acromyrmex_echinatior	AECH19445*		43	280	131	346	240			119	309			1285	87	6402	109	2353	80			catctgacatgaTCACATGAtcacgtccgaaa	5'	>-65
	Atta_cephalotes	ACEP24633*		43	280	134	352	240			120	309			1336	87	5463	109	ND	80			tatctgacatgaTCACATGAtccgaaattgat	5UTR?	>-60
	Solenopsis_invicta	Sinv\Atp6ap2		43	284	128	394	240			297	309			799	84	5699	109	1056	80	14		tacctgacatgaTCATATGAtcgcgtctgaaa	5UTR?	-81
	Camponotus_floridanus	CFLO15335*		43	282	128	263	237			356	309			155	87	2862	109	433	80			gatctgaagttaTCACATGAtcatgtccgaaa	5UTR?	>-65
	Harpegnathos_saltator	Hsal\Atp6ap2		43	427	128	177	240			116	309			177	93	3736	109	648	80			gaccacaggtgaTCACATGAtcatgtccgaat	5UTR?	>-70

	Pogonomyrmex_barbatus	Pbar\Atp6ap2		43	320	128	208	240			126	309			485	87	2782	109	ND	80			tgtctgacataaTCACATGAtcgcgtctgaaa	5UTR?	>-66
	Linepithema_humile	Lhum\Atp6ap2		43	303	128	288	234			430	306			102	84	2685	112	307	80			catctgatgtgaTCACATGAtcgcgtccgaaa	5UTR?	>-60
	Nasonia_vitripennis	NV14085		46	108	128	82	231			81	315			72	108	1095	103	88	80			gtgcaaaaggtgTCATATGAtcggacgaaaaa	5UTR?	>-84
	Nasonia_giraulti	Ngir\Atp6ap2		46	139	128	82	231			81	315			72	108	1102	103	88	80			gtgcaaaaggtgTCATATGAtcggacgaaaaa	5UTR?	>-84
	Nasonia_longicornis	Nlon\Atp6ap2		46	139	128	82	231			81	315			72	108	ND	103	88	80			gtgcaaaaggtgTCATATGAtcggacgaaaaa	5UTR?	>-84
Odonata	*Ladona_fulva*	Lful\Atp6ap2		43	108	128	166	241			1031	152			83	108	289	109	73	80			catatcgtctggTCATATGAttcttaagcgaa	5UTR?	>-40
Hemiptera	*Acyrthosiphon_pisum*	ACYPI007940	176	52	148	137	441	240			62	132	224	180	694	75	82	103	1872	80	1224		ND		
Phthiraptera	*Pediculus_humanus*	PHUM376100		135	66	423									165	141	105	109	253	80			ND		
**Crustacea**	*Daphnia_pulex*	Dpul\Atp6ap2		46	59	128	126	309+61+168							62	81	69	115	76	80			agcacgaacacaTCACCTGActttagctgtca	5'	>-80
Ixodida	*Ixodes_scapularis*																								
