# Supplementary material for: Structural and Comparative Analyses of Insects Suggest the Presence of an Ultra-Conserved Regulatory Element of the Genes Encoding Vacuolar-Type ATPase Subunits and Assembly Factors
Source: Biology (Basel). 2023 Aug 13;12(8):1127. doi: 10.3390/biology12081127 (PMC10452791; doi:10.3390/biology12081127)
Supplement: Supplementary file 1 [file biology-12-01127-s001.zip › Supplementary Files S20-S37/Supp_File_S36_Tmem199_INS.pdf]

Gene Cluster ID	Tmem199
**Drosophila melanogaster gene**	**CG7071**
FlyBase ID	FBgn0260467
**Predicted function**	**ATPase, H+ transporting, vacuolar ER assembly factor, Vma12**
**Comment on Gene model**	comparative data suggest that this is a dicistronic gene. Gene with dicistronic mRNA.

Tmem199 CLEAR element conservation:

```
          _________5'/5'UTR/intron______
 Dmel\    CGCTACGATTTCTCATGTGACAGCTGTGGGCG
 Mdom\    gcaaccctagtaTCATGTGAcaaccattgaca
 Ccap\    aaatgacaattcTCATGTGAcaagaaaaatgt
 Gmor\    ttataaattttcTCACGTGActttcatctttt
 Llon\    tggagtgttaaaTCACGTGAagaatgacagct
 Ppap\    ggacgtcacacaTCACGTGAgcaatgacagct

          ____________5'/5'UTR__________
 Agam\    CCCCGCGAGTGGTCACATGAGCCCTGATATTA
 Aara\    CCCCGCGAGTGGTCACATGAGCCCTGATACTA
 Aqan\    CCTCGCTAGTGGTCACATGAGCCCTGATACTA
 Achr\    CTCCGCAAGTGGTCACATGAGCCTTGCTACTT
 Aepi\    TTTCCCGAGTGGTCACATGAGCTTTGCTAGCT
 Amin\    CATCTGATCTGGTCACATGAGGTGGTGCACCT
 Afun\    CATCTGGTTTGGTCACATGAGGTGGTTCTTGT
 Aste\    TGTTGGTGTCGGTCACATGAGCCAGTGATATA
 Adir\    TCTGCATAGAGATCACATGAGCCGAAACGCAT
 Aalb\    TCGTACGGTGGGTCACATGAGCAACTGAGTCG
 Adar\    GTGGAACGAGGGTCACATGAGCAAATTGAGTC
 Aaeg\    AATATTTGACAATCACATGATACCCCAAACAA
 Cqui\    AAACTCTGACAATCACATGATTGAAAATAATG

 Mdes\    aagcgtatttggTCATGTGAtcatatgctaat
 Pxyl\    cggggctacgggTCACATGAtaaatgtcagat
 Tcas\    actacaccccgaTCATATGAtcaaactgaaaa
 Dpon\    actgtcacttagTCAGCTGActgtcatgtggc

 Aech\    TTGTACAGCCAGTCACGTGACAGGTATGACAT
 Acep\    TAGTACAGCCAATCACGTGACAGGTATATTGG
 Sinv\    TAGTACAGTCGGTCACGTGACGGCACACCGTC
 Pbar\    TGATAAACCAGTCACGTGACGACGCATCGCT
 Lhum\    TGATATAACTTATCATGTGACAAAGTGTTGCC
 Cflo\    GGCACAACCTAGTCATGTGACGACGAAGGAGT
```

Tmem199 gene structure comparisons:
(Click on Species for genomic, mRNA and protein sequence informations)
Key: UTR region, CDS region

Order	Species	Tmem199 orthologs		muted (CG34131)						Tmem199(CG7071)					Extended CLEAR region	Position	bps from TSS
				5'exon	int	exon	int	ex		5'exon	intron	exon3'					
Diptera (Drosophilidae)	*Drosophila_melanogaster*	Dmel\CG7071	28	22	60	281	66	171	5	598	69	311	77		cgctacgatttcTCATGTGAcagctgtgggcg	5'UTR	6
Diptera (Muscidae)	*Musca_domestica*	Mdom\Tmem199		19	77	272	59	168	24	574	461	302			gcaaccctagtaTCATGTGAcaaccattgaca	5'UTR?	>-33
Diptera (Tephritidae)	*Ceratitis_capitata*	Ccap\Tmem199		22	54	272	54	171	14	574	58	281			aaatgacaattcTCATGTGAcaagaaaaatgt	5'UTR?	>-9
Diptera (Glossinidae)	*Glossina_morsitans*	Gmor\Tmem199	7	22	70	272	56	171	9	607	57	284	90		ttataaattttcTCACGTGActttcatctttt	intron1	75
Diptera (Psychodidae)	*Lutzomyia_longipalpis*	Llon\Tmem199		25	74	83	65	357	18	846					tggagtgttaaaTCACGTGAagaatgacagct	5'	>-4

	Phlebotomus_papatasi	Ppap\Tmem199		25	62	83	70	357			4	565	65	296			ggacgtcacacaTCACGTGAgcaatgacagct	5'	>-4
Diptera (Culicidae)	*Anopheles_gambiae*	AGAP013136									63+66+29	634	69	296	75		ccccgcgagtggTCACATGAgccctgatatta	5'UTR?	224
	Anopheles_arabiensis	Aara\Tmem199										634	69	296			ccccgcgagtggTCACATGAgccctgatacta	5'UTR?	>-130
	Anopheles_quadriannulatus	Aqan\Tmem199										634	77	296			cctcgctagtggTCACATGAgccctgatacta	5'UTR?	>-128
	Anopheles_christyi	Achr\Tmem199										634	67	293			ctccgcaagtggTCACATGAgccttgctactt	5'UTR?	>-121
	Anopheles_epiroticus	Aepi\Tmem199										628	69	293			tttcccgagtggTCACATGAgctttgctagct	5'UTR?	>-128
	Anopheles_minimus	Amin\Tmem199										622	69	323			catctgatctggTCACATGAggtggtgcacct	5'UTR?	>-88
	Anopheles_funestus	Afun\Tmem199										622	69	329			catctggtttggTCACATGAggtggttcttgt	5'UTR?	>-91
	Anopheles_stephensi	Aste\Tmem199										631	87	338			tgttggtgtcggTCACATGAgccagtgatata	5'UTR?	>-69
	Anopheles_dirus	Adir\Tmem199										915					tctgcatagagaTCACATGAgccgaaacgcat	5'UTR?	>-72
	Anopheles_nili	Anil\Tmem199										>146	74						
	Anopheles_albimanus	Aalb\Tmem199										607	76	320			tcgtacggtgggTCACATGAgcaactgagtcg	5'UTR?	>-15
	Anopheles_darlingi	Adar\Tmem199										598	68	320			gtggaacgagggTCACATGAgcaaattgagtc	5'UTR?	>-16
	Culex_pipiens_qui.	CPIJ009433*										595	63	335			acatcaaactctTCATATGAcgaagataggtg<br>aaactctgacaaTCACATGAttgaaaataatg	5'UTR?	>-202<br>>-82
	Aedes_aegypti	AAEL011748									91+56+2	598	19614	326			aatatttgacaaTCACATGAtaccccaaacaa	5'UTR	-8
Diptera (Cecidomyiidae)	*Mayetiola_destructor*	Mdes\Tmem199		22	158	83	86	189	82	168	74	688	77	287			aagcgtatttggTCATGTGAtcatatgctaat	intron2	>278
Lepidoptera	*Bombyx_mori*	Bmor\Tmem199		25	202	83	80	189	ND	177	3	712	80	317			ND		
	Manduca_sexta	Msex\Tmem199		19	440	83	321	189	144	177	2	658	1693	311			ND		
	Danaus_plexippus	Dple\Tmem199		19	205	83	138	189	307	177	2	652	68	308			tcttgtaacttgTCAGGTGAttttctttttaa	intron1	>92
	Heliconius_melpomene	Hmel\Tmem199		19	213	83	209	189	392	177	3	661	531	290			ND		
	Plutella_xylostella	Pxyl\Tmem199		19	292	83	116	189	392	174	6	685	279	338			cggggctacgggTCACATGAtaaatgtcagat	5'	>-56
Strepsiptera	*Mengenilla_moldrzyki*	Mmol\Tmem199										801					ND		
Coleoptera	*Dendroctonus_ponderosae*	Dpon\Tmem199									112	813			151		actgtcacttagTCAGCTGActgtcatgtggc	5'	-7
	Tribolium_castaneum	Tcas\Tmem199<br>LOC656791*		19	55	83	56	189	ND	ND	ND	550	47	254			actacaccccgaTCATATGAtcaaactgaaaa	5'UTR?	>-11
Hymenoptera	*Apis_florea*	Aflo\Tmem199		19	95	83	190	189	92	168	179	418	ND	254			ND		
	Apis_mellifera	Amel\Tmem199	7	19	103	83	199	189	90	168	183	418	116	254	196		ND		
	Bombus_impatiens	Bimp\Tmem199		19	103	83	169	189	87	168	151	418	65	254			ND		
	Bombus_terrestris	Bter\Tmem199		19	103	83	169	189	87	168	150	418	65	254			ND		
	Megachile_rotundata	Mrot\Tmem199		19	238	83	238	189	77	168	123	418	64	251			atatttgtagttTCATGTAAgaaaaaataata	intron2	>242
	Acromyrmex_echinatior	Aech\Tmem199		19	128	83	684	189	77	168	106	430	93	245			ttgtacagccagTCACGTGAcaggtatgacat	5'	>-60
	Atta_cephalotes	Acep\Tmem199		19	128	83	680	189	76	168	109	430	93	245			tagtacagccaaTCACGTGAcaggtatattgg	5'	>-66
	Solenopsis_invicta	Sinv\Tmem199		19	119	83	453	189	84	168	137	427	283	245			tagtacagtcggTCACGTGAcggcacaccgtc	5'	>-67
	Pogonomyrmex_barbatus	Pbar\Tmem199		19	115	83	430	189	82	168	118	427	102	242			tgataaaaccagTCACGTGAcgacgcatcgct	5'	>-50
	Harpegnathos_saltator	Hsal\Tmem199		ND	ND	83	113	189	249	168	101	424	90	251			ND		
	Linepithema_humile	Lhum\Tmem199		19	114	83	380	189	87	168	129	427	91	245			tgatataacttaTCATGTGAcaaagtgttgcc	5'	>-46

	Camponotus_floridanus	Cflo\Tmem199		19	121	83	384	189	84	168	115	427	87	239			ggcacaacctagTCATGTGAcgacgaaggagt	5'	>-58
	Nasonia_giraulti	Ngir\Tmem199		19	151	83	194	189	76	168	19	418	81	251			aaactttttTCAATTGAaaagattttt<br>atgtgtactaTCAGATGAattaccattt	3'UTR	
	Nasonia_longicornis	Nlon\Tmem199		19	151	83	194	189	76	168	19	418	81	251			aaactttttTCAATTGAaaagatttgt<br>atgtgtactaTCAGATGAattaccattt	3'UTR	1034<br>1351
	Nasonia_vitripennis	NV17772		19	151	83	195	189	76	168	19	418	81	251			aaactttttTCAATTGAaaagattttt<br>atgtgtactaTCAGATGAattac cattt	3'UTR	1034<br>1347
Hemiptera	*Acyrthosiphon_pisum*																		
	Rhodnius_prolixus																		
Phthiraptera	*Pediculus_humanus*																		
**Crustacea**	*Daphnia_pulex*																		
Ixodida	*Ixodes_scapularis*																		
	Homo_sapiens																		
	Nematostella_vectensis																		
	Trichoplax_adhaerens																		
