# Supplementary material for: Structural and Comparative Analyses of Insects Suggest the Presence of an Ultra-Conserved Regulatory Element of the Genes Encoding Vacuolar-Type ATPase Subunits and Assembly Factors
Source: Biology (Basel). 2023 Aug 13;12(8):1127. doi: 10.3390/biology12081127 (PMC10452791; doi:10.3390/biology12081127)
Supplement: Supplementary file 1 [file biology-12-01127-s001.zip › Supplementary Files S20-S37/Supp_File_S37_Vma21_INS.pdf]

Gene Cluster ID	Vma21
**Drosophila melanogaster gene**	**CG5969**
FlyBase ID	FBgn0036998
**Predicted function**	**Vacuolar ATPase assembly integral membrane protein VMA21-like domain**

Vma21 gene structure comparison:

Key: UTR region, CDS region

Order	Species	Vma21 orthologs	5'exon		intron	exon	intron	exon3'			Extended CLEAR region	Position	bps from TSS
Diptera (Drosophilidae)	*Drosophila_melanogaster*	Dmel\CG5969	101	72	62	246			517		actaccactttaTCATGTGAtttgtgatttgt	5'	-11
Diptera (Muscidae)	*Musca_domestica*	Mdom\Vma21		78	69	240					ctgtataactaaTCATGTGAcgttgattggac	5'	>-120
Diptera (Tephritidae)	*Ceratitis_capitata*	Ccap\Vma21	96	51	60	255			298		ggtgaaatgtcaTCACGTGAcgttttcttcat	5'	-31
Diptera (Glossinidae)	*Glossina_morsitans*	Gmor\Vma21		318							ND		
Diptera (Psychodidae)	*Phlebotomus_papatasi*	Ppap\Vma21	115	27	94	270			423		aatagagaaaagTCACATGAcgccgaattgag	intron1	172
Diptera (Culicidae)	*Anopheles_stephensi*	Aste\Vma21		42	110	285					ND		
	Anopheles_gambiae	AGAP01354	55	42	81	273			369		ND		
	Anopheles_darlingi	Adar\Vma21		42	64	279					ND		
	Aedes_aegypti	AAEL011546		42	142	279					atcgctcattggTCAGCTGAttcccttcagaa	5'	
		AAEL013448		42	142	279					atcgctcattggTCAGCTGAttcccttcagaa	5'	
	Culex_pipiens_qui.	CPIJ002927		39	136	264					ND		
Diptera (Cecidomyiidae)	*Mayetiola_destructor*	Mdes\Vma21		ND	ND	282					gtaatttgtttaTCATGTGAtttgtgtacact	5'	ND
Lepidoptera	*Bombyx_mori*	Bmor\Vma21	76	21	87	249			192		ataggatgtgTCATATGAatcatcattt	5'	1012
	Danaus_plexippus	Dple\Vma21		21	88	252					ND		
	Heliconius_melpomene	Hmel\Vma21		21	86	252					ND		
	Manduca_sexta	Msex\Vma21		21	80	249					acattcattcatTCATGTGAtcgatcattgat	5'	-86
Coleoptera	*Dendroctonus_ponderosae*	Dpon\Vma21	64	109			55	149	244		aaagtggactagTCATGTGAtttcagttcaat	3'UTR	378
	Tribolium_castaneum	Tcas\Vma21									NA		
Hymenoptera	*Apis_mellifera*	GB11041	396	15	90	106	528	140	1688		ND		
	Apis_florea	Aflo\Vma21		15	112	106*	729	140			ND		
	Bombus_terrestris	Bter\Vma21	631	15	90	106	401	140			ND		
	Bombus_impatiens	Bimp\Vma21		15	90	106	648	140			ND		
	Megachile_rotundata	Mrot\Vma21		15	97	106	402	140			ND		
	Acromyrmex_echinatior	Aech\Vma21		15	92	106	173	143			ND		
	Atta_cephalotes	Acep\Vma21		15	98	106	ND	143			ND		

	Solenopsis_invicta	Sinv\Vma21	127	18	108	106	142	143	284		ND		
	Pogonomyrmex_barbatus	Pbar\Vma21		15	110	106	197	143			ND		
	Linepithema_humile	Lhum\Vma21		15	108	106	129	143			ND		
	Harpegnathos_saltator	Hsal\Vma21		15	99	106	160	140			ND		
	Camponotus_floridanus	Cflo\Vma21		15	99	106	171	143			ND		
	Nasonia_giraulti	Ngir\Vma21		15	166	106	83	140			ND		
	Nasonia_longicornis	Nlon\Vma21		15	166	106	83	140			ND		
	Nasonia_vitripennis	NV14065	399	15	166	106	83	140	951		ND		
Hemiptera	*Acyrthosiphon_pisum*	Apis\Vma21									NA		
	Rhodnius_prolixus	Rpro\Vma21									NA		
Phthiraptera	*Pediculus_humanus*	Phum\Vma21									NA		
	Ladona Fulva	Lful\Vma21									NA		
**Crustacea**	*Daphnia_pulex*	Dpul\Vma21	30	36	80	39+64+73	56	137	49		actgtcgaccACACGTGAcaacgccgtc	5'	24
Ixodida	*Ixodes_scapularis*	Isca\Vma21	161+2927+14	264					305				
	Homo_sapiens	VMA21	124	53	6269	110	1175	143	4306		caccgccggtaagTCATGTGAgcgcccgccccc	5'	114
Cnidaria	*Nematostella_vectensis*	Nvec\Vma21	112	38	102	104	1627	134	131		ctgaagtatTCACACGAgcggatcgcaa	5'	214
Placozoa	*Trichoplax_adhaerens*	Tadh\Vma21		ND	ND	104	459	131			gttgaaagtaTCATACGAtcaattaaag	5'	1228
