# Supplementary material for: Structural and Comparative Analyses of Insects Suggest the Presence of an Ultra-Conserved Regulatory Element of the Genes Encoding Vacuolar-Type ATPase Subunits and Assembly Factors
Source: Biology (Basel). 2023 Aug 13;12(8):1127. doi: 10.3390/biology12081127 (PMC10452791; doi:10.3390/biology12081127)
Supplement: Supplementary file 1 [file biology-12-01127-s001.zip › Supplementary Files S3-S19/Supp_File_S10_Atp6v1H_DROS.pdf]

Gene Cluster ID	Atp6v1H
**Drosophila melanogaster gene**	**VhaSFD (CG17332)**
FlyBase ID	FBgn0027779
**Predicted function**	**V-type ATPase, H+ transporting, V1 subunit H**
Cytogenetic map	2L:6A12-36A12
Gene sequence location	2L:16723138..16727825[-]
**Dmel CLEAR sequence**	**TCACAAGAccagTCAGCTGA**
CLEAR sequence location	2L:16727801..16727782

FlyBase VhaSFD (CG17332) gene model & CLEAR element (red) positions:

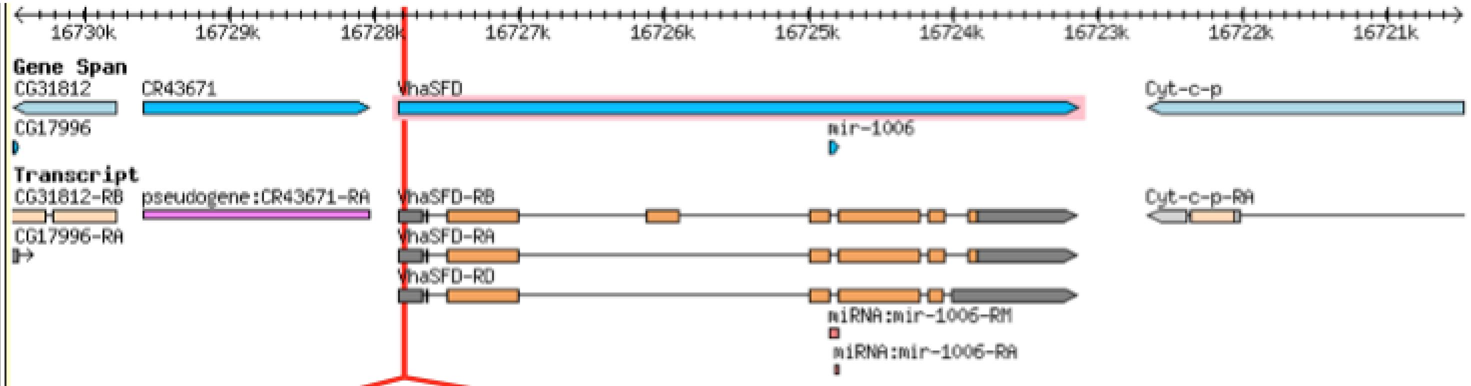


```
Dmel\  GCTGCAGAAAAGTCACAAGACC--AGTCAGCTGAATTT-CGGTGCTG
Dsim\  GCTGCAGAAAAGTCACAAGACC--AGTCAGCTGAATTT-CGGTGCTG
Dsec\  GCTGCAGAAAAGTCACAAGACC--AGTCAGCTGAATTT-CGGTGCTG
Dyak\  GCTGCAGAAAAGTCACAAGACC--AGTCAGCTGAATTT-CGGTGCTG
Dere\  GCTGCAGAAAAGTCACAAGACC--AGTCAGCTGAATTT-CGGTGCTG
Dbia\  CCTGCAGAAAAGTCACAAGACC--AGTCAGCTGAATTT-CGGTCGTG
Dsuz\  GCTGCAGAAAAGTCACAAGACC--AGTCAGCTGAATTT-CGGTCGTG
Dtak\  GACACAAAAAAGTCACAAGACC-AAGTCAGCTGAATTCTCGTCGTGA
Deug\  GACTGCAGAAAATCACAAGACC--AGTCAGCTGAATTT-CGGTGCTG
Dele\  TATGCAGAAAAGTCACAAGACC--AGTCAGCTGAATTT-CGGTGCGT
Drho\  GCTGCAGAAAAGTCACAAGACC--AGTCAGCTGAATTT-CGGTGCTG
Dfic\  ACTGAAGAAAAGTCACAAGACC--AGTCAGCTGAATTT-CGGTGCTT
Dkik\  AGCCCCAAAAAGTCACAAGACCCAAGTCAGCTGAATTTCGGTGAATC
Dbip\  GCGGCGAAAAAGTCACAAGACC--AGTCAGCTGAATTT-CTGTCTTT
Dana\  GCGGCGAAAAAGTCACAAGACC--AGTCAGCTGAATTT-CTGTCTTT
Dpse\  ACACTGGGCCAGTCACAAGACCAAAGTCAGCTGAATTTTCTGTTCGT
Dper\  ACACTGGGCCAGTCACAAGACCAAAGTCAGCTGAATTTTCTGTTCGT
Dmir\  ACACTGGGCCAGTCACAAGACCAAAGTCAGCTGAATTTTCTGTTCGT
Dwil\  ACTCTAAAGAAGTCACAAGACC--AGTCAGCTGAATTTTCGGTGCTG
Dalb\  ACGGCGAATAAGTCACAAGACC--AGTCAGCTGAATTT-CGGTGCTG
Dmoj\  ACGGCGAATAAGTCACAAGACC--AGTCAGCTGAATTT-CGGTGCTG
Dvir\  CACGGCGAATAATCACAAGACC--AGTCAGCTGAATTT-CGGTGCTG
Dgri\  ACGGCGAATAAGTCACAAGACC--AGTCAGCTGAATTT-CGGTGCTG
```

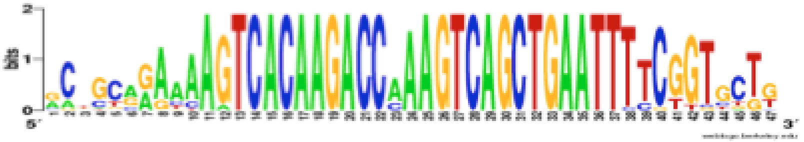

Atp6v1H (VhaSFD) gene structure comparison:

Key: UTR region, CDS region, intron, alternatively spliced exon

Species	VhaSFD Atp6v1H orthologs	5'exon		intron	exon	intron	exon	intron	exon	intron	exon	intron	exon	intron	exon3'		Extended CLEAR region	Position	bps from TSS
Drosophila_melanogaster	Dmel\VhaSFD	159	37	141	479	896	222	898	135	64	563	60	114	160	79	681	gaaaagTCACAAGAccagTCAGCTGAatttcg	5'UTR	36
Drosophila_simulans	Dsim\GD21903		37	145	479	919	231	882	135	64	563	57	114	158	79		gaaaagTCACAAGAccagTCAGCTGAatttcg	5'UTR?	
Drosophila_sechellia	Dsec\GM17165		37	145	479	905	231	869	135	64	563	55	114	160	79		gaaaagTCACAAGAccagTCAGCTGAatttcg	5'UTR?	
Drosophila_yakuba	Dyak\GE13171		37	145	479	900	231	891	135	64	563	65	114	160	79		gaaaagTCACAAGAccagTCAGCTGAatttcg	5'UTR?	
Drosophila_erecta	Dere\GG20115		37	147	479	901	231	885	135	64	563	65	114	160	79		gaaaagTCACAAGAccagTCAGCTGAatttcg	5'UTR?	
Drosophila_biarmipes	Dbia\VhaSFD		37	160	479	1075	231	741	135	64	563	103	114	153	79		gaaaagTCACAAGAccagTCAGCTGAatttcg	5'UTR?	
Drosophila_suzukii	Dsuz\VhaSFD		37	158	479	1098	222	845	135	64	563	109	114	153	79		gaaaagTCACAAGAccagTCAGCTGAatttcg	5'UTR?	
Drosophila_takahashii	Dtak\VhaSFD		37	169	479	1500	231	795	135	64	563	61	114	151	79		aaaaagTCACAAGAccaagTCAGCTGAattctcg	5'UTR?	
Drosophila_eugracilis	Deug\VhaSFD		37	154	479	983	231	845	135	64	563	95	114	162	79		agaaaaTCACAAGAccagTCAGCTGAatttcg	5'UTR?	
Drosophila_elegans	Dele\VhaSFD		37	157	479	1057	231	767	135	64	563	181	114	160	79		gaaaagTCACAAGAccagTCAGCTGAatttcg	5'UTR?	
Drosophila_rhopaloa	Drho\VhaSFD		37	156	479	1071	231	779	135	64	563	215	114	159	79		gaaaagTCACAAGAccagTCAGCTGAatttcg	5'UTR?	
Drosophila_ficusphila	Dfic\VhaSFD		37	150	479	1022	231	830	135	64	563	67	114	160	79		gaaaagTCACAAGAccagTCAGCTGAatttcg	5'UTR?	
Drosophila_kikkawai	Dkik\VhaSFD		37	146	479	1199	222	767	135	64	563	65	114	158	79		aaaaagTCACAAGAcccaagTCAGCTGAatttcg	5'UTR?	
Drosophila_bipectinata	Dbip\VhaSFD		37	154	479	854	222	524	135	64	563	68	114	142	79		aaaaagTCACAAGAccagTCAGCTGAatttct	5'UTR?	
Drosophila_ananassae	Dana\GF24362		37	156	479	821	231	557	135	64	563	60	114	138	79		aaaaagTCACAAGAccagTCAGCTGAatttct	5'UTR?	
Drosophila_pseudoobscura	Dpse\GA14464		37	125	479	1063	240	1088	135	64	563	60	114	146	79		gccagTCACAAGAccaaagTCAGCTGAattttc	5'UTR?	
Drosophila_persimilis	Dper\GL19340		37	130	479	1066	240	1081	135	64	563	60	114	146	79		gccagTCACAAGAccaaagTCAGCTGAattttc	5'UTR?	
Drosophila_miranda	Dmir\VhaSFD		37	130	479	1037	240	1112	135	64	563	60	114	146	79		gccagTCACAAGAccaaagTCAGCTGAattttc	5'UTR?	
Drosophila_willistoni	Dwil\GK15126		37	173	479	1595	219	1052	135	66	563	98	114	194	79		aagaagTCACAAGAccagTCAGCTGAattttc	5'UTR?	
Drosophila_albomicans	Dalb\VhaSFD		37	121	479	ND	ND	ND	135	66	563	63	114	196	79		aataagTCACAAGAccagTCAGCTGAatttcg	5'UTR?	
Drosophila_mojavensis	Dmoj\GI21354		37	136	479	1823	231	1150	135	64	563	63	114	200	79		aataagTCACAAGAccagTCAGCTGAatttcg	5'UTR?	
Drosophila_virilis	Dvir\GJ11146		37	129	479	2101	219	1227	135	64	563	61	114	174	79		gaataaTCACAAGAccagTCAGCTGAatttcg	5'UTR?	
Drosophila_grimshawi	Dgri\GH10362		37	122	479	1351	234	911	135	64	563	62	114	185	79		aataagTCACAAGAccagTCAGCTGAatttcg	5'UTR?	

Key: UTR region, CDS region, intron, alternatively spliced exon
