# Supplementary material for: Structural and Comparative Analyses of Insects Suggest the Presence of an Ultra-Conserved Regulatory Element of the Genes Encoding Vacuolar-Type ATPase Subunits and Assembly Factors
Source: Biology (Basel). 2023 Aug 13;12(8):1127. doi: 10.3390/biology12081127 (PMC10452791; doi:10.3390/biology12081127)
Supplement: Supplementary file 1 [file biology-12-01127-s001.zip › Supplementary Files S3-S19/Supp_File_S11_Atp6v0a_DROS.pdf]

Gene Cluster ID	Atp6v0a
**Drosophila melanogaster gene**	**Vha100-2 (CG18617)**
FlyBase ID	FBgn0028670
**Predicted function**	**ATPase, H+ transporting, lysosomal V0 subunit a**
Protein size	834 amino acids
Cytogenetic map	91A5-91A5
Gene sequence location	3R:14216039..14224876[-]
**Dmel CLEAR sequence**	**TCATGTGA**
CLEAR sequence location	3R:14224185..14224192
Drosophila Atp6v0a paralogs	**Vha100-1 (CG1709); Vha100-3 (CG30329); Vha100-4 (CG7678); Vha100-5 (CG12602)**

FlyBase Vha100-2 (CG18617) gene model & CLEAR element (red) position:

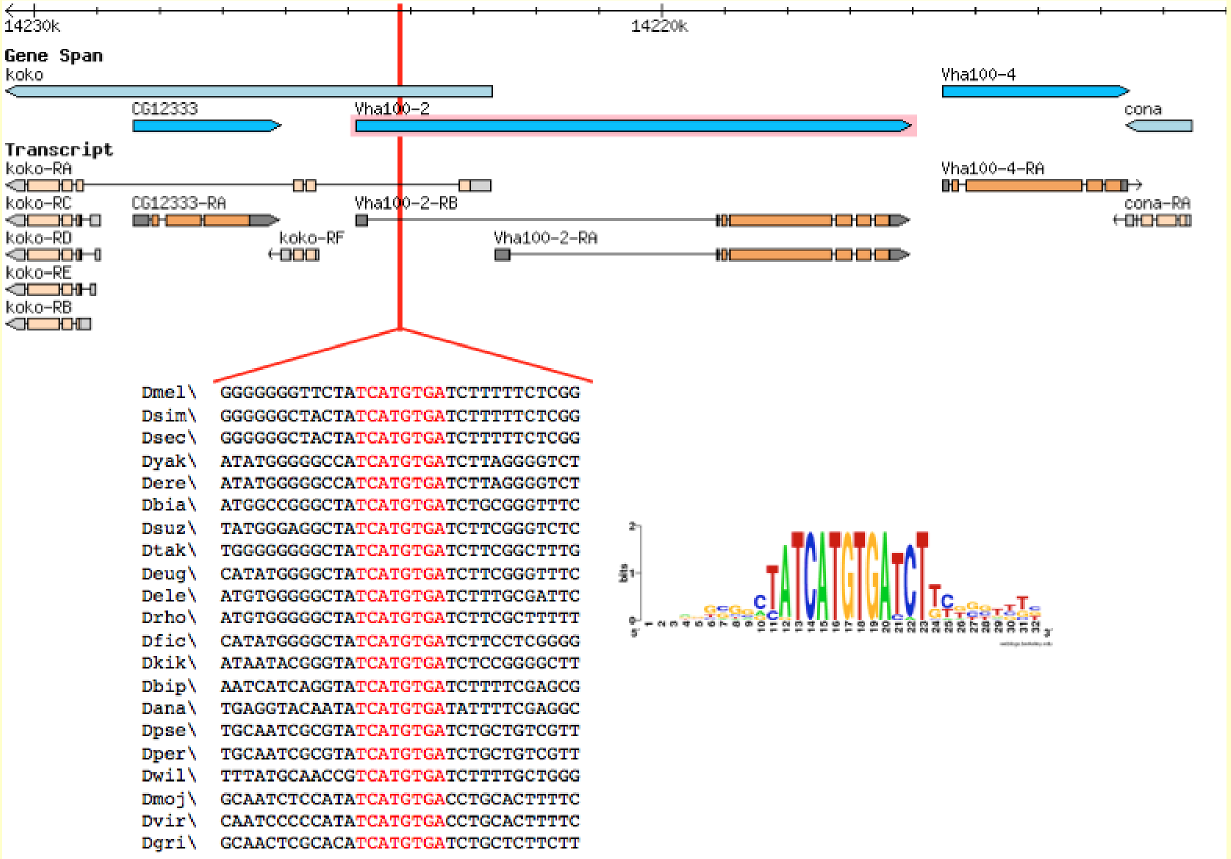

Vha100-2 (CG18617) gene structure comparison in Drosophila species:

Key: UTR region, CDS region

Species	Vha100-2 Atp6v0a orthologs	5'exon	intron	exon		intron	exon	intron	exon	intron	exon	intron	exon3'			Extended CLEAR region	Position	bps from TSS
Drosophila_melanogaster	Dmel\Vha100-2	163	5582	21	117	66	1628	60	259	62	235	71	266	308		ggggggggttctaTCATGTGAtctttttctcgg	intron1	685
Drosophila_simulans	Dsim\GD20170				117	66	1628	59	259	62	235	60	266			ggggggctactaTCATGTGAtctttttctcgg	intron1?	
Drosophila_sechellia	Dsec\GM18705	162	3187	21	117	66	1628	60	259	62	235	60	266			ggggggctactaTCATGTGAtctttttctcgg	intron1?	
Drosophila_yakuba	Dyak\GE25199				117	66	1628	60	259	62	235	57	266			atatgggggccaTCATGTGAtcttaggggtct	intron1?	
Drosophila_erecta	Dere\Vha100-2	114	ND	21	117	66	1628	56	259	62	235	58	266			atatgggggccaTCATGTGAtcttaggggtct	intron1?	
Drosophila_biarmipes	Dbia\Vha100-2				117	67	1628	61	259	62	235	59	266			atggccgggctaTCATGTGAtctgcgggtttc	intron1?	
Drosophila_suzukii	Dsuz\Vha100-2				117	67	1628	61	259	69	235	59	266			tatgggaggctaTCATGTGAtcttcgggtctc	intron1?	
Drosophila_takahashii	Dtak\Vha100-2				117	65	1631	325	259	472	235	58	266			tgggggggggctaTCATGTGAtcttcggctttg	intron1?	
Drosophila_eugracilis	Deug\Vha100-2				117	68	1628	60	494 (259+235)			56	266			catatggggctaTCATGTGAtcttcgggtttc	intron1?	
Drosophila_elegans	Dele\Vha100-2				117	60	1625	65	259	71	235	58	266			atgtgggggctaTCATGTGAtctttgcgattc	intron1?	
Drosophila_rhopaloa	Drho\Vha100-2				117	60	1625	66	259	71	235	58	266			atgtgggggctaTCATGTGAtcttcgcttttt	intron1?	
Drosophila_ficusphila	Dfic\Vha100-2				117	60	1628	60	259	58	235	57	266			catatggggctaTCATGTGAtcttcctcgggg	intron1?	
Drosophila_kikkawai	Dkik\Vha100-2				117	59	1628	58	259	74	235	58	266			ataatacgggtaTCATGTGAtctccggggctt	intron1?	
Drosophila_bipectinata	Dbip\Vha100-2				117	62	1628	69	259	57	238	61	266			aatcatcaggtaTCATGTGAtcttttcgagcg	intron1?	
Drosophila_ananassae	Dana\GF18112				117	60	1887 (1628+259)			61	238	61	266			tgaggtacaataTCATGTGAtattttcgaggc	intron1?	
Drosophila_pseudoobscura	Dpse\GA15015	92	3069	21	117	67	1628	70	259	67	235	65	266			tgcaatcgcgtaTCATGTGAtctgctgtcgtt	intron1?	
Drosophila_persimilis	Dper\GL23189			21	117	67	1628	70	259	67	235	65	266			tgcaatcgcgtaTCATGTGAtctgctgtcgtt	intron1?	
Drosophila_miranda	Dmir\Vha100-2																	
Drosophila_willistoni	Dwil\GK11490				117	68	1634	73	259	64	226	61	266			tttatgcaaccgTCATGTGAtcttttgctggg	intron1?	
Drosophila_albomicans	Dalb\Vha100-2																	
Drosophila_mojavensis	Dmoj\GI24258	122	5377	21	117	60	1631	58	262	67	232	62	266			gcaatctccataTCATGTGAcctgcacttttc	intron1?	
Drosophila_virilis	Dvir\GJ10832	161	5187	21	117	69	1631	64	262	65	235	60	266			caatcccccataTCATGTGAcctgcacttttc	intron1?	
Drosophila_grimshawi	Dgri\GH19224	65		27	117	85	1631	70	262	74	235	67	269			gcaactcgcacaTCATGTGAtctgctcttctt	intron1?	

Vha100-1 (CG1709) gene structure comparison in Drosophila species:

Key: UTR region, CDS region, alternatively spliced exons

Species	Vha100-1 Atp6v0a orthologs	5'exon	intron	exon		intron	exon	intron	exon	intron	exon	intron	exon	intron	exon	intron	exon	intron	exon	intron	exon	intron	exon	intron	exon	intron	exon3'	
Drosophila_melanogaster	Dmel\Vha100-1	129	414	23	117	300	177	73	129	833 (767)	92 (158)	431	101	595	42	450	127	86	1156	64	218	376	211	65	172	60	103	294
Drosophila_simulans	Dsim\GD21418	88	413	23	117	304	177	73	129	819 (753)	92 (158)	436	101	591	42	443	127	86	1156	63	218	377	124 64 88		172		103	294
Drosophila_sechellia	Dsec\GM12768*				117	305	177	73	129	833 (767)	92 (158)	446	101	591	42	427	127	86	1156	63	218	375	211	64	172	60	103	

Drosophila_yakuba	Dyak\GE23836				117	299	177	68	129	831 (765)	92 (158)	472	101	640	42	436	127	85	1156	59	218	375	211	62	172	60	103	
Drosophila_erecta	Dere\GG11646				117	329	177	73	129	819 (753)	92 (158)	419	101	650	42	454	127	100	1156	64	218	372	211	62	172	59	103	
Drosophila_biarmipes	Dbia\Vha100-1				117	322	177	67	129	966 (900)	92 (158)	417	101	707	42	400	127	391	1156	58	218	375	211	64	172	59	103	
Drosophila_suzukii	Dsuz\Vha100-1				117	326	177	98	129	886 (820)	92 (158)	419	101	655	42	384	127	363	1156	55	218	399	211	60	172	66	103	
Drosophila_takahashii	Dtak\Vha100-1				117	289	177	157	129	989 (923)	92 (158)	411	101	547	42	406	127	135	1156	59	218	335	211	63	172	66	103	
Drosophila_eugracilis	Deug\Vha100-1				117	332	177	111	129	911 (845)	92 (158)	397	101	549	42	432	127	122	1156	58	218	445	211	62	172	67	103	
Drosophila_elegans	Dele\Vha100-1				117	390	177	173	129	885 (819)	92 (158)	431	101	734	42	437	127	325	1156	53	218	412	211	62	172	58	103	
Drosophila_rhopaloa	Drho\Vha100-1				117	310	177	119	129	972 (906)	92 (158)	401	101	708	42	412	127	311	1156	59	218	397	211	67	172	66	103	
Drosophila_ficusphila	Dfic\Vha100-1				117	421	177	99	129	809 (743)	92 (158)	419	101	595	42	434	127	246	1156	63	218	383	211	63	172	65	103	
Drosophila_kikkawai	Dkik\Vha100-1				117	161	177	96	129	1294 (1225)	92 (161)	411	101	1086	42	437	127	59	1156	60	218	576	211	68	172	61	106	
Drosophila_bipectinata	Dbip\Vha100-1				117	64	177	71	129	859 (793)	92 (158)	394	101	450	42	427	127	59	1156	61	218	406	214	61	172	58	106	
Drosophila_ananassae	Dana\GF23290				117	55	177	57	129	792 (726)	92 (158)	405	101*	458	42	401	127	58	1156	62	218	435	214	62	172	62	106	
Drosophila_pseudoobscura	Dpse\GA14320*				117	62	177	69	129*	892 (826)	92 (158)	871	101	471	42	502	127	63	1156	59	218	305	211	60	172	63	103	
Drosophila_persimilis	Dper\GL23912				117	62	177	69	129	887 (821)	92 (158)	949	101	475	42	490	127	63	1156	58	218	305	211	59	172	63	103	
Drosophila_willistoni	Dwil\GK11891				117	96	177	81	129	775 (712)	92 (155)	1052	101	425	42	440	127	67	1159	70	218	360	217	59	172	68	109	
Drosophila_mojavensis	Dmoj\GI22777				117	65	177	70	129	856 (790)	92 (158)	628	101	439	42	343	127	63	1159	62	218	361	211	70	172	56	106	
Drosophila_virilis	Dvir\GJ22780				117*	56	177	61	129	799 (736)	92 (155)	626	101	456	42	289	127	62	1159	54	218	344	199	57	172	56	109	
Drosophila_grimshawi	Dgri\GH17453				117	66	177	329	129	827 (752)	92 (167)	676	101	423	42	317	127	75	1159	73	215	364	196	62	172	58	109	

Vha100-3 (CG30329) gene structure comparison in Drosophila species:

Key: UTR region, CDS region

Species	Vha100-3 Atp6v0a orthologs	5'exon	intron	exon3'		
Drosophila_melanogaster	Dmel\Vha100-3		2715			
Drosophila_sechellia	Dsec\GM19860		2715			
Drosophila_yakuba	Dyak\GE13869		2736			
Drosophila_erecta	Dere\GG20932		2688			
Drosophila_biarmipes	Dbia\Vha100-3					

Drosophila_suzukii	Dsuz\Vha100-3		2760			
Drosophila_takahashii	Dtak\Vha100-3					
Drosophila_eugracilis	Deug\Vha100-3					
Drosophila_elegans	Dele\Vha100-3					
Drosophila_rhopaloa	Drho\Vha100-3					
Drosophila_ficusphila	Dfic\Vha100-3					
Drosophila_kikkawai	Dkik\Vha100-3					
Drosophila_bipectinata	Dbip\Vha100-3					
Drosophila_ananassae	Dana\GF13738		980	64	1897?	
Drosophila_pseudoobscura	Dpse\GA15764		2706			
Drosophila_persimilis	Dper\GL10577		2706			
Drosophila_willistoni	Dwil\GK20688		2622			
Drosophila_mojavensis	Dmoj\GI20149		2613			
Drosophila_virilis	Dvir\GJ22427		2613			
Drosophila_grimshawi	Dgri\GH19727		2610			

Vha100-4 (CG7678) gene structure comparison:

Key: UTR region, CDS region

Species	Vha100-4 Atp6v0a orthologs	exon		intron	exon	intron	exon	intron	exon	intron	exon3'	
Drosophila_melanogaster	Dmel\Vha100-4	85	153	119	1872			62	244	56	266	114
Drosophila_simulans	Dsim\GD20171		153	127	1872			62	244	56	266	
Drosophila_sechellia	Dsec\Vha100-4		153	127	1872*			62	244	56	266	
Drosophila_yakuba	Dyak\GE25200		144	111	1872			58	244	56	266	
Drosophila_erecta	Dere\GG16473*		ND	ND	>1713			57	244	56	266	
Drosophila_biarmipes	Dbia\Vha100-4		153	121	1872			63	244	56	266	
Drosophila_suzukii	Dsuz\Vha100-4		153	117	1872			59	244	55	266	
Drosophila_takahashii	Dtak\Vha100-4		144	1461	1872			403	244	56	266	
Drosophila_eugracilis	Deug\Vha100-4		153	132	1872			60	244	54	266	
Drosophila_elegans	Dele\Vha100-4		153	97	1872			62	244	56	266	
Drosophila_rhopaloa	Drho\Vha100-4		153	396	1872			66	244	56	>206	
Drosophila_ficusphila	Dfic\Vha100-4		153	101	1872			53	244	56	266	
Drosophila_kikkawai	Dkik\Vha100-4		153	79	1872			65	235	59	266	
Drosophila_bipectinata	Dbip\Vha100-4		153	66	1872			50	244	55	266	
Drosophila_ananassae	Dana\GF18113		153	66	1872			50	244	62	266	
Drosophila_pseudoobscura	Dpse\GA20518		153	62	1872			67	238	59	266	
Drosophila_persimilis	Dper\GL24346		153	62	1872			67	238	59	266	
Drosophila_miranda	Dmir\Vha100-4		153	62	1872			67	238	59	266	

Drosophila_willistoni	Dwil\GK11491		174	70	1872			66	241	59	266	
Drosophila_mojavensis	Dmoj\GI24259		159	62	417	62	1455	55	247	56	266	
Drosophila_virilis	Dvir\GJ10833		159	56	417	64	1455	51	259	56	266	
Drosophila_grimshawi	Dgri\GH19225		159	63	417	59	1455	54	247	316	269	

Vha100-5 (CG12602) gene structure comparison in Drosophila species:

Key: UTR region, CDS region

Species	Vha100-5 Atp6v0a orthologs	5' exon	intron	exon		intron	exon	intron	exon3'	
Drosophila_melanogaster	Dmel\Vha100-5	85	2493	21	2179	104	172	62	94	
Drosophila_simulans	Dsim\GD23787*				2179	105	172	63	94	
Drosophila_sechellia	Dsec\GM19181				2179	96	172	63	94	
Drosophila_yakuba	Dyak\GE18538				2179	97	172	61	94	
Drosophila_erecta	Dere\GG23732				2179	115	172	61	94	
Drosophila_biarmipes	Dbia\Vha100-5				2179	357	172	58	94	
Drosophila_suzukii	Dsuz\Vha100-5				ND	ND	172	62	94	
Drosophila_takahashii	Dtak\Vha100-5				2179	389	172	64	94	
Drosophila_eugracilis	Deug\Vha100-5									
Drosophila_elegans	Dele\Vha100-5				2179	819	172	67	94	
Drosophila_rhopaloa	Drho\Vha100-5				2179	1201	172	63	94	
Drosophila_ficusphila	Dfic\Vha100-5				2188	576	172	795	94	
Drosophila_kikkawai	Dkik\Vha100-5				2182	73	172	64	94	
Drosophila_bipectinata	Dbip\Vha100-5				2167	302	172	58	94	
Drosophila_ananassae	Dana\GF14569				2167	311	172	59	94	
Drosophila_pseudoobscura	Dpse\GA11714				2206	64	172	63	94	
Drosophila_persimilis	Dper\GL19314				2194	64	172	63	94	
Drosophila_miranda	Dmir\Vha100-5				2191	71	172	68	94	
Drosophila_willistoni	Dwil\GK24362*				2197*	65	172	67	91	
Drosophila_mojavensis	Dmoj\GI18075				2191	355	172	68	94	
Drosophila_virilis	Dvir\GJ16665				2191	70	172	67	94	
Drosophila_grimshawi	Dgri\GH11370				2185	63	172	71	94	
