# Supplementary material for: Structural and Comparative Analyses of Insects Suggest the Presence of an Ultra-Conserved Regulatory Element of the Genes Encoding Vacuolar-Type ATPase Subunits and Assembly Factors
Source: Biology (Basel). 2023 Aug 13;12(8):1127. doi: 10.3390/biology12081127 (PMC10452791; doi:10.3390/biology12081127)
Supplement: Supplementary file 1 [file biology-12-01127-s001.zip › Supplementary Files S3-S19/Supp_File_S12_Atp6v0b_DROS.pdf]

Gene Cluster ID	Atp6v0b
**Drosophila melanogaster gene**	**VhaPPA1-1 (CG7007)**
FlyBase ID	FBgn0028662
**Predicted function**	**ATPase, H+ transporting, V0 subunit b**
Protein size	212 amino acids
Cytogenetic map	3R:88D6-88D6
Gene sequence location	3R:10728048..10729796[-]
**Dmel CLEAR sequence**	**TCATGTGA**
CLEAR sequence location	3R: 10729742.. 10729749[-]
Drosophila Atp6v0b paralog	VhaPPA1-2 (CG7026)

FlyBase VhaPPA1-1 (CG7007)gene model & CLEAR element (red) position:

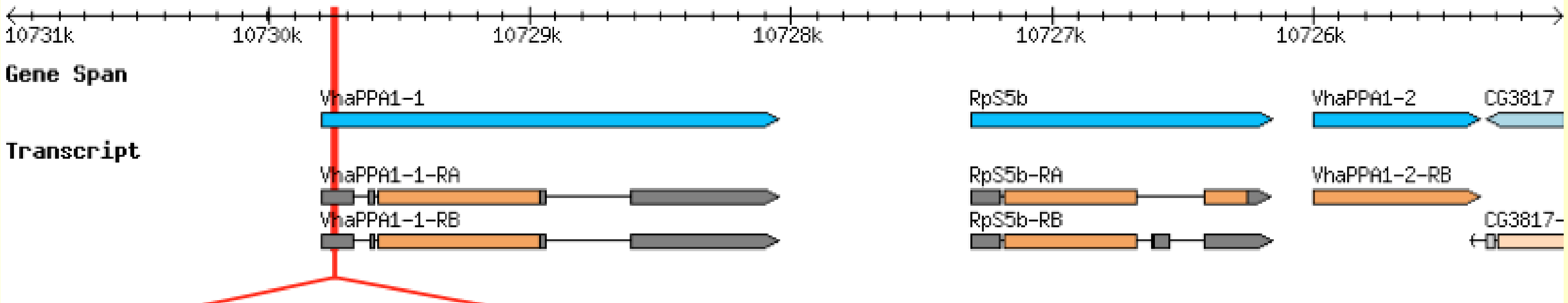


```
Dmel\  AGAAAATGTACGTCATGTGATTGTGGATTCTT
Dsim\  AGAAAATGTACGTCATGTGATTGTGGATTCTT
Dsec\  AGAAAATGTACGTCATGTGATTGTGGATTCTT
Dyak\  AGAAAATGTACGTCATGTGATTGTGGATTCTT
Dere\  AGAAAATGTACGTCATGTGATTGTGGATTCTT
Dbia\  AGAAAATGTACGTCATGTGATTGTGGATTCTT
Dsuz\  AGAAAATGTACGTCATGTGATTGTGGATTTTT
Dtak\  AGAAAATGTACGTCATGTGATTGTGGATTCTT
Deug\  AGAAAATGTACGTCATGTGATTGTGGATTCTT
Dele\  AGAAAATGTACGTCATGTGATTGTGGATTCTT
Drho\  AGAAAATGTACGTCATGTGATTGTGGATTCTT
Dfic\  AGAAAATGTACGTCATGTGATTGTGGATTCTT
Dkik\  AGAAAATGTACGTCATGTGATTGTGGATTCTT
Dbip\  AGAAAATGTACGTCATGTGATTGTGGATTCTT
Dana\  AGAAAATGTACGTCATGTGATTGTGGATTCTT
Dpse\  AATAGAGAAACGTCATGTGATTGTGATTTTTT
Dper\  AATAGAGAAACGTCATGTGATTGTGATTTTTT
Dmir\  AATAGAGAAACGTCATGTGATTGTGATTTTTT
Dwil\  TGATAGAGAAAATCATGTGATTGTGGTCATTT
Dalb\  AGAAAATGTACGTCATGTGATTGTGGATTCTT
Dmoj\  AGAAAATGTACGTCATGTGATTGTGGATTCTT
Dvir\  AGAAAATGTACGTCATGTGATTGTGGATTCTT
Dgri\  AGAAAATGTACGTCATGTGATTGTGGATTCTT
```

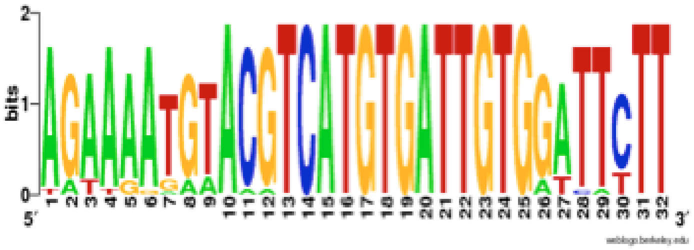

Atp6v0b gene structure comparison:

Key: UTR region, CDS region

Species	Atp6v0b orthologs	5'exon	intron	exon3'				Extended CLEAR region	Position	bps from TSS
Drosophila_melanogaster	Dmel\VhaPPA1-1	124	59	19	639	502		agaaaatgtacgTCATGTGAttgtggattctt	5'UTR	47

Drosophila_simulans	Dsim\GD20390				639			agaaaatgtacgTCATGTGAttgtggattctt	5'UTR?	
Drosophila_sechellia	Dsec\GM25814				639			agaaaatgtacgTCATGTGAttgtggattctt	5'UTR?	
Drosophila_yakuba	Dyak\GE26419				639			agaaaatgtacgTCATGTGAttgtggattctt	5'UTR?	
Drosophila_erecta	Dere\GG20968				639			agaaaatgtacgTCATGTGAttgtggattctt	5'UTR?	
Drosophila_biarmipes	Dbia\VhaPPA1-1				639			agaaaatgtacgTCATGTGAttgtggattctt	5'UTR?	
Drosophila_suzukii	Dsuz\VhaPPA1-1				639			agaaaatgtacgTCATGTGAttgtggattttt	5'UTR?	
Drosophila_takahashii	Dtak\VhaPPA1-1				639			agaaaatgtacgTCATGTGAttgtggattctt	5'UTR?	
Drosophila_eugracilis	Deug\VhaPPA1-1				639			agaaaatgtacgTCATGTGAttgtggattctt	5'UTR?	
Drosophila_elegans	Dele\VhaPPA1-1				639			agaaaatgtacgTCATGTGAttgtggattctt	5'UTR?	
Drosophila_rhopaloa	Drho\VhaPPA1-1				639			agaaaatgtacgTCATGTGAttgtggattctt	5'UTR?	
Drosophila_ficusphila	Dfic\VhaPPA1-1				639			agaaaatgtacgTCATGTGAttgtggattctt	5'UTR?	
Drosophila_kikkawai	Dkik\VhaPPA1-1				639			agaaaatgtacgTCATGTGAttgtggattctt	5'UTR?	
Drosophila_bipectinata	Dbip\VhaPPA1-1				639			agaaaatgtacgTCATGTGAttgtggattctt	5'UTR?	
Drosophila_ananassae	Dana\GF17169				639			agaaaatgtacgTCATGTGAttgtggattctt	5'UTR?	
Drosophila_pseudoobscura	Dpse\GA20025				639			aatagagaaacgTCATGTGAttgtgatttttt	5'UTR?	
Drosophila_persimilis	Dper\GL21698				639			aatagagaaacgTCATGTGAttgtgatttttt	5'UTR?	
Drosophila_miranda	Dmir\VhaPPA1-1				639			aatagagaaacgTCATGTGAttgtgatttttt	5'UTR?	
Drosophila_willistoni	Dwil\GK11008				639			tgatagagaaaaTCATGTGAttgtggtcattt	5'UTR?	
Drosophila_albomicans	Dalb\VhaPPA1-1				639			agaaaatgtacgTCATGTGAttgtggattctt	5'UTR?	
Drosophila_mojavensis	Dmoj\GI22994				639			agaaaatgtacgTCATGTGAttgtggattctt	5'UTR?	
Drosophila_virilis	Dvir\GJ24692				639			agaaaatgtacgTCATGTGAttgtggattctt	5'UTR'	
Drosophila_grimshawi	Dgri\GH19517				639			agaaaatgtacgTCATGTGAttgtggattctt	5'UTR?	

Paralog: VhaPPA1-2 (CG7026)

Drosophila_melanogaster	Dmel\VhaPPA1-2				639					
Drosophila_simulans	Dsim\GD20392				627					
Drosophila_sechellia	Dsec\GM25816				627					
Drosophila_yakuba	Dyak\GE26421				639					
Drosophila_erecta	Dere\GG20990				627					
Drosophila_biarmipes	Dbia\VhaPPA1-2				627					
Drosophila_suzukii	Dsuz\VhaPPA1-2				627					
Drosophila_takahashii	Dtak\VhaPPA1-2				627					
Drosophila_eugracilis	Deug\VhaPPA1-2				627					
Drosophila_elegans	Dele\VhaPPA1-2				627					
Drosophila_rhopaloa	Drho\VhaPPA1-2				627					
Drosophila_ficusphila	Dfic\VhaPPA1-2				627					
Drosophila_kikkawai	Dkik\VhaPPA1-2				627					
Drosophila_bipectinata	Dbip\VhaPPA1-2				627					
Drosophila_ananassae	Dana\GF17172*				627					
Drosophila_pseudoobscura	Dpse\GA26375				624					
Drosophila_persimilis	Dper\GL21700				624					
Drosophila_miranda	Dmir\VhaPPA1-2				624					
Drosophila_willistoni	Dwil\GK13787				630					
Drosophila_albomicans	Dalb\VhaPPA1-2				621					
Drosophila_mojavensis	Dmoj\GI22996				624					
Drosophila_virilis	Dvir\GJ24694				624					

Drosophila_grimshawi	Dgri\GH19519				624					

Key: UTR region, CDS region
