# Supplementary material for: Structural and Comparative Analyses of Insects Suggest the Presence of an Ultra-Conserved Regulatory Element of the Genes Encoding Vacuolar-Type ATPase Subunits and Assembly Factors
Source: Biology (Basel). 2023 Aug 13;12(8):1127. doi: 10.3390/biology12081127 (PMC10452791; doi:10.3390/biology12081127)
Supplement: Supplementary file 1 [file biology-12-01127-s001.zip › Supplementary Files S3-S19/Supp_File_S13_Atp6v0c_DROS.pdf]

Gene Cluster ID	Atp6v0c
**Drosophila melanogaster gene**	**Vha16-1 (CG3161)**
FlyBase ID	FBgn0262736
**Predicted function**	**ATPase, H+ transporting, V0 subunit c**
Protein size	159 amino acids
Cytogenetic map	2R:42B2-42B2
Gene sequence location	2R:2512747..2520633[-]
**Dmel CLEAR sequence(s)**	**TCACGTGA; TCATGTGA; TCACAAGA; TCACATGC; TCACATGG; TCACATGA; TCACATGA; TCATGTGA**
CLEAR sequence location(s)	2R:2520496..2520503; 2R:2520461..2520468; 2R:2518990..2518997; 2R:2518647..2518654; 2R:2518597..2518604; 2R:2518099..2518106; 2R:2516246..2516253; 2R:2515721..2515728
Drosophila Atp6v0c paralogs	Vha16-2 (CG32089); Vha16-3 (CG32090); Vha16-4 (CG9013); Vha16-5 (CG6737)

FlyBase Vha16-1 (CG3161) gene model & CLEAR element (red) positions:

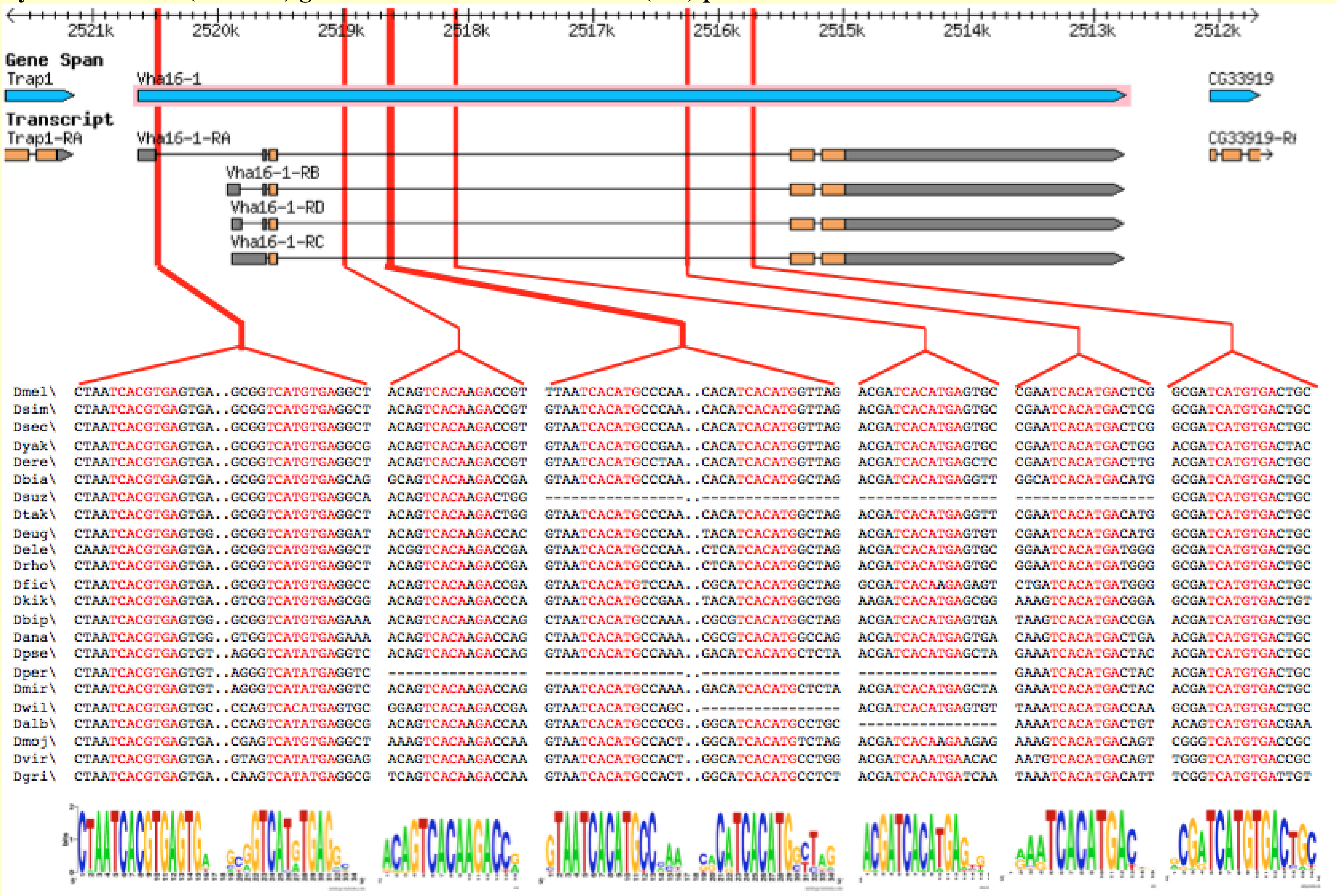

Atpv0c gene structure comparison:

Key: UTR region, CDS region

Species	Atp6v0c orthologs	5'exon	intron	exon	intron	exon3'	Extended CLEAR region	Position	bps from

												TSS
Drosophila_melanogaster	Dmel\Vha16-1	96+854+29	85	4105	190	57	205	2228		ttagatttctaaTCACGTGAgtgaaaaagcag<br>aaagataggcggTCATGTGAggctagccctgc	exon/int<br>intron1	93<br>128
Drosophila_simulans	Dsim\GD10321		85	4121	190	60	205			ttagatttctaaTCACGTGAgtgaaaaagcag<br>aaagataggcggTCATGTGAggctagccctgc	exon/int?<br>intron1	
Drosophila_sechellia	Dsec\GM20869		85	4106	190	60	205			ttagatttctaaTCACGTGAgtgaaaaagcag<br>aaagataggcggTCATGTGAggctagccctgc	exon/int?<br>intron1	
Drosophila_yakuba	Dyak\Vha16		85	3891	190	61	205			ttagatttctaaTCACGTGAgtgaaaaagcag<br>aaagataggcggTCATGTGAggcgagcactgc	exon/int?<br>intron1	
Drosophila_erecta	Dere\GG10820		85	4097	190	57	205			ttagaattctaaTCACGTGAgtgaaaaagcag<br>aaagataggcggTCATGTGAggctagccctgc	exon/int?<br>intron1	
Drosophila_biarmipes	Dbia\Vha16-1		85	4328	190	68	205			ttagatttctaaTCACGTGAgtgaaaaggcaa<br>aaagataggcggTCATGTGAgcaggccagccc	exon/int?<br>intron1	
Drosophila_suzukii	DsuzVha16-1		85	ND	190	60	205			ttagatttctaaTCACGTGAgtgaaaaagcaa<br>aaagataggcggTCATGTGAggcaagccctgc	exon/int?<br>intron1	
Drosophila_takahashii	Dtak\Vha16-1		85	4179	190	71	205			ttagatttctaaTCACGTGAgtgaaaaagcca<br>aaagataggcggTCATGTGAggctagccctgc	exon/int?<br>intron1	
Drosophila_eugracilis	Deug\Vha16-1		85	4018	190	57	205			ttagatttctaaTCACGTGAgtggaaaagcat<br>gccgatcggcggTCATGTGAggatagcctgcc	exon/int?<br>intron1	
Drosophila_elegans	Dele\Vha16-1		85	4592	190	57	205			ttagatttcaaaTCACGTGAgtgaaaaaaaga<br>aaagataggcggTCATGTGAggctcgcgctgc	exon/int?<br>intron1	
Drosophila_rhopaloa	Drho\Vha16-1		85	4326	190	58	205			ttagatttctaaTCACGTGAgtgaaaagctaa<br>aaagattggcggTCATGTGAggctcgcgctgc	exon/int?<br>intron1	
Drosophila_ficusphila	Dfic\Vha16-1		85	4020	190	57	205			ttagatttctaaTCACGTGAgtgaaaaagcag<br>aaagataggcggTCATGTGAggcccagcggaa	exon/int?<br>intron1	
Drosophila_kikkawai	Dkik\Vha16-1		85	3795	190	70	205			ttagatttctaaTCACGTGAgtgaagaagagt<br>catagttagtcgTCATGTGAgcggaaaagttt	exon/int?<br>intron1	
Drosophila_bipectinata	Dbip\Vha16-1		85	3476	190	68	205			ttagatttctaaTCACGTGAgtggaaaaaaac<br>gtaaataagcggTCATGTGAgaaaaagttcct	exon/int?<br>intron1	
Drosophila_ananassae	Dana\GF13808		85	3429	190	70	205			ttagatttctaaTCACGTGAgtggaaaaaaca<br>gtaaataagtggTCATGTGAgaaaaagttcct	exon/int?<br>intron1	
Drosophila_pseudoobscura	Dpse\GA16335		85	4293	190	72	205			ttagatttctaaTCACGTGAgtgtgaacaaat<br>acgaccagagggTCATATGAggtccaataaaa	exon/int?<br>intron1	
Drosophila_persimilis	Dper\GL20293		85	3204?	190	72	205			ttagatttctaaTCACGTGAgtgtgaacaaat<br>acgaccagagggTCATATGAggtccaataaaa	exon/int?<br>intron1	
Drosophila_miranda	Dmir\Vha16-1		85	3204?	190	72	205			ttagatttctaaTCACGTGAgtgtgaacaaat<br>acggccagagggTCATATGAggtccaataaaa	exon/int?<br>intron1	
Drosophila_willistoni	Dwil\GK19648		85	5070	190	76	205			ttaaatttctaaTCACGTGAgtgccgagatac<br>aaaactctccagTCACATGAgtgcgaggtctt	exon/int?<br>intron1	
Drosophila_albomicans	Dalb\Vha16-1		85	>5215	190	65	205			ttagatttctaaTCACGTGAgtgaaaaaaaaa<br>aaaaacaaccagTCATATGAggcgcaatcaaa	exon/int?<br>intron1	

Drosophila_mojavensis	Dmoj\GI21278		85	5825	190	71	205			ttagatttctaaTCACGTGAgtgaaaaaaaag gaaacaatcgagTCATGTGAggctccatcgaa	exon/int? intron1	
Drosophila_virilis	Dvir\GJ20880		82	5409	190	59	205			ttagatttctaaTCACGTGAgtgaagaaaaaa aaaaaacagtagTCATATGAggagcaatcgaa	exon/int? intron1	
Drosophila_grimshawi	Dgri\GH22892		91	5471	190	72	205			ttagatttctaaTCACGTGAgtgaaaaaaata ttcacaatcaagTCATATGAggcgcaatcgta	exon/int? intron1	
Vha16-2 (CG32089)												
Drosophila_melanogaster	Dmel\Vha16-2	40	477					78				
Drosophila_simulans	Dsim\Vha16-2		477									
Drosophila_sechellia	Dsec\GM25274		477									
Drosophila_yakuba	Dyak\GE21813		477									
Drosophila_erecta	Dere\GG15505		477									
Drosophila_biarmipes	Dbia\Vha16-2		477									
Drosophila_suzukii	DsuzVha16-2		477									
Drosophila_takahashii	Dtak\Vha16-2		477									
Drosophila_eugracilis	Deug\Vha16-2		480									
Drosophila_elegans	Dele\Vha16-2		480									
Drosophila_rhopaloa	Drho\Vha16-2		480									
Drosophila_ficusphila	Dfic\Vha16-2		477									
Drosophila_kikkawai	Dkik\Vha16-2		477									
Drosophila_bipectinata	Dbip\Vha16-2		480									
Drosophila_ananassae	Dana\GF25266		480									
Drosophila_pseudoobscura	Dpse\GA26306		480									
Drosophila_persimilis	Dper\GL21512		480									
Drosophila_miranda												
Drosophila_willistoni	Dwil\GK17171		483									
Drosophila_albomicans	Dalb\Vha16-2		477									
Drosophila_mojavensis	Dmoj\GI11613		477									
Drosophila_virilis	Dvir\GJ11292		480									

Drosophila_grimshawi	Dgri\GH16653		480					
Vha16-3 (CG32090)								
Drosophila_melanogaster	Dmel\Vha16-3		477					
Drosophila_simulans	Dsim\GD14307		477					
Drosophila_sechellia	Dsec\GM25273		477					
Drosophila_yakuba	Dyak\GE21812		477					
Drosophila_erecta	Dere\GG15504		477					
Drosophila_biarmipes	Dbia\Vha16-3		477					
Drosophila_suzukii	DsuzVha16-3		477					
Drosophila_takahashii	Dtak\Vha16-3		477					
Drosophila_eugracilis	Deug\Vha16-3		477					
Drosophila_elegans	Dele\Vha16-3		477					
Drosophila_rhopaloa	Drho\Vha16-3		477					
Drosophila_ficusphila	Dfic\Vha16-3		477					
Drosophila_kikkawai	Dkik\Vha16-3		477					
Drosophila_bipectinata	Dbip\Vha16-3		474					
Drosophila_ananassae	Dana\GF25265		474					
Drosophila_pseudoobscura	Dpse\GA26304		489					
Drosophila_persimilis	Dper\GL21511		489					
Drosophila_miranda	Dmir\Vha16-3		489					
Drosophila_willistoni	Dwil\GK17170		483					
Drosophila_albomicans	Dalb\Vha16-3		486					
Drosophila_mojavensis	Dmoj\GI11612		477					
Drosophila_virilis	Dvir\GJ11291		480					
Drosophila_grimshawi	Dgri\GH16652		486					
Vha16-4 (CG9013)								
Drosophila_melanogaster	Dmel\Vha16-4	46	468	53				

Drosophila_simulans	Dsim\GD25509		477					
Drosophila_sechellia	Dsec\GM20023		477					
Drosophila_yakuba	Dyak\GE14233		477					
Drosophila_erecta	Dere\GG22236		477					
Drosophila_biarmipes	Dbia\Vha16-4		468					
Drosophila_suzukii	Dsuz\Vha16-4		477					
Drosophila_takahashii	Dtak\Vha16-4		480					
Drosophila_eugracilis	Deug\Vha16-4		480					
Drosophila_elegans	Dele\Vha16-4		477					
Drosophila_rhopaloa	Drho\Vha16-4		477					
Drosophila_ficusphila	Dfic\Vha16-4		468					
Drosophila_kikkawai	Dkik\Vha16-4		471					
Drosophila_bipectinata	Dbip\Vha16-4		495					
Drosophila_ananassae	Dana\GF13441*		495					
Drosophila_pseudoobscura	Dpse\GA21477		498					
Drosophila_persimilis	Dper\GL10498		498					
Drosophila_miranda	Dmir\Vha16-4		498					
Drosophila_willistoni	Dwil\GK22034		483					
Drosophila_albomicans	Dalb\Vha16-4		483					
Vha16-5 (CG6737)								
Drosophila_melanogaster	Dmel\Vha16-5		582					
Drosophila_simulans	Dsim\GD22230		576					
Drosophila_sechellia	Dsec\GM11483		576					
Drosophila_yakuba	Dyak\GE13384		576					
Drosophila_erecta	Dere\GG10362		576					
Drosophila_biarmipes	Dbia\Vha16-5		579					
Drosophila_suzukii	DsuzVha16-5		576					

Drosophila_takahashii								
Drosophila_eugracilis								
Drosophila_elegans	Dele\Vha16-5		576					
Drosophila_rhopaloa	Drho\Vha16-5		576					
Drosophila_ficusphila	Dfic\Vha16-5		567					
Drosophila_kikkawai	Dkik\Vha16-5		558					
Drosophila_bipectinata	Dbip\Vha16-5		558					
Drosophila_ananassae	Dana\GF19671		558					
Drosophila_pseudoobscura	Dpse\GA25245		549					
Drosophila_persimilis	Dper\GL26032		549					
Drosophila_miranda	Dmir\Vha16-5		549					
Drosophila_willistoni	Dwil\GK18316		555					
Drosophila_albomicans	Dalb\Vha16-5		510					
Drosophila_mojavensis	Dmoj\GI13313		528					
Drosophila_virilis	Dvir\GJ11943		540					
Drosophila_grimshawi	Dgri\GH10920		522					
