# Supplementary material for: Structural and Comparative Analyses of Insects Suggest the Presence of an Ultra-Conserved Regulatory Element of the Genes Encoding Vacuolar-Type ATPase Subunits and Assembly Factors
Source: Biology (Basel). 2023 Aug 13;12(8):1127. doi: 10.3390/biology12081127 (PMC10452791; doi:10.3390/biology12081127)
Supplement: Supplementary file 1 [file biology-12-01127-s001.zip › Supplementary Files S3-S19/Supp_File_S14_Atp6v0d_DROS.pdf]

Gene Cluster ID	Atp6v0d
**Drosophila melanogaster gene**	**VhaAC39-1 (CG2934)**
FlyBase ID	FBgn0028665
**Predicted function**	**ATPase, H+ transporting, lysosomal 38kDa, V0 subunit d**
Protein size	350 amino acids
Cytogenetic map	X:3F4-3F4
Gene sequence location	X:3774773..3777004[+]
**Dmel CLEAR sequence**	**TCATATGA; TCACATGA**
CLEAR sequence locations	X:3774820..3774827; X:3775210..3775217
Drosophila Atp6v0d paralog	VhaAC39-2

FlyBase Vha39-1 (CG2934) gene model & CLEAR element (red) positions:

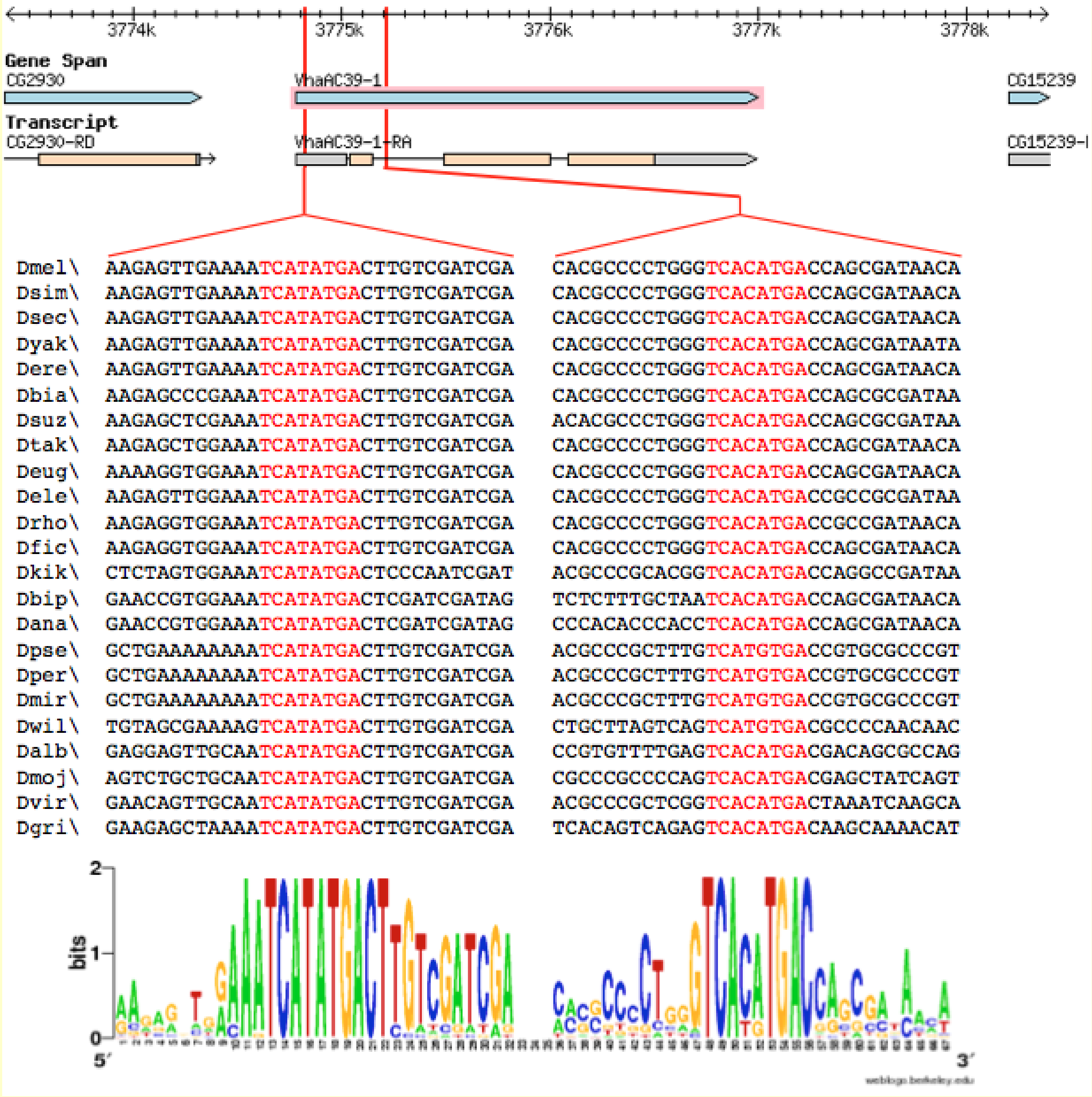

Drosophila Atp6v0d gene structure comparison:

Key: UTR region, CDS region

Species	Atp6v0d orthologs	5'exon		intron	exon	intron	exon3'			Extended CLEAR region	Position	bps from TSS
Drosophila_melanogaster	Dmel\VhaAC39-1	249	127	338	509	90	417	502		aagagttgaaaaTCATATGActtgtcgatcga<br>cacgcccctgggTCACATGAccagcgataaca	5'UTR<br>intron1	47<br>437
Drosophila_simulans	Dsim\GD16660*		127	348	509	90	417			aagagttgaaaaTCATATGActtgtcgatcga<br>cacgcccctgggTCACATGAccagcgataaca	5'UTR?<br>intron1	
Drosophila_sechellia	Dsec\GM12314		127	348	509	90	417			aagagttgaaaaTCATATGActtgtcgatcga<br>cacgcccctgggTCACATGAccagcgataaca	5'UTR?<br>intron1	
Drosophila_yakuba	Dyak\GE16319		127	384	509	70	417			aagagttgaaaaTCATATGActtgtcgatcga<br>cacgcccctgggTCACATGAccagcgataata	5'UTR?<br>intron1	
Drosophila_erecta	Dere\GG18679		127	369	509	90	417			aagagttgaaaaTCATATGActtgtcgatcga<br>cacgcccctgggTCACATGAccagcgataaca	5'UTR?<br>intron1	
Drosophila_biarmipes	Dbia\VhaAC39-1		127	658	509	357	417			aagagcccgaaaTCATATGActtgtcgatcga<br>cacgcccctgggTCACATGAccagcgcgataa	5'UTR?<br>intron1	
Drosophila_suzukii	Dsuz\VhaAC39-1		127	671	509?	ND	417?			aagagctcgaaaTCATATGActtgtcgatcga<br>acacgccctgggTCACATGAccagcgcgataa	5'UTR?<br>intron1	
Drosophila_takahashii	Dtak\VhaAC39-1		127	660	509	357	417			aagagctggaaaTCATATGActtgtcgatcga<br>cacgcccctgggTCACATGAccagcgataaca	5'UTR?<br>intron1	
Drosophila_eugracilis	Deug\VhaAC39-1		127	536	509	162	417			aaaaggtggaaaTCATATGActtgtcgatcga<br>cacgcccctgggTCACATGAccagcgataaca	5'UTR?<br>intron1	
Drosophila_elegans	Dele\VhaAC39-1		127	899	509	731	417			aagagttggaaaTCATATGActtgtcgatcga<br>cacgcccctgggTCACATGAccgccgcgataa	5'UTR?<br>intron1	
Drosophila_rhopaloa	Drho\VhaAC39-1		127	734	509	350	417			aagaggtggaaaTCATATGActtgtcgatcga<br>cacgcccctgggTCACATGAccgccgataaca	5'UTR?<br>intron1	
Drosophila_ficusphila	Dfic\VhaAC39-1		127	579	509	279	417			aagaggtggaaaTCATATGActtgtcgatcga<br>cacgcccctgggTCACATGAccagcgataaca	5'UTR?<br>intron1	
Drosophila_kikkawai	Dkik\VhaAC39-1		127	539	509	76	417			ctctagtggaaaTCATATGActcccaatcgat<br>acgcccgcacggTCACATGAccaggccgataa	5'UTR?<br>intron1	
Drosophila_bipectinata	Dbip\VhaAC39-1		127	431	509	60	417			gaaccgtggaaaTCATATGActcgatcgatag<br>tctctttgctaaTCACATGAccagcgataaca	5'UTR?<br>intron1	
Drosophila_ananassae	Dana\GF21182		127	471	509	81	417			gaaccgtggaaaTCATATGActcgatcgatag<br>cccacacccaccTCACATGAccagcgataaca	5'UTR?<br>intron1	
Drosophila_pseudoobscura	Dpse\GA15530		127	237	509	68	417			gctgaaaaaaaaTCATATGActtgtcgatcga<br>acgcccgctttgTCATGTGAccgtgcgcccgt	5'UTR?<br>intron1	
Drosophila_persimilis	Dper\GL14761		127	237	509	68	417			gctgaaaaaaaaTCATATGActtgtcgatcga<br>acgcccgctttgTCATGTGAccgtgcgcccgt	5'UTR?<br>intron1	
Drosophila_miranda	Dmir\Vha39-1		127	235	509	68	417			gctgaaaaaaaaTCATATGActtgtcgatcga<br>acgcccgctttgTCATGTGAccgtgcgcccgt	5'UTR?<br>intron1	

Drosophila_willistoni	Dwil\GK10228		127	409	509	71	417			tgtagcgaaaagTCATATGActtgtggatcga<br>ctgcttagtcagTCATGTGAcgccccaacaac	5'UTR?<br>intron1	
Drosophila_albomicans	Dalb\Vha39-1		127	409	509	71	417			gaggagttgcaaTCATATGActtgtcgatcga<br>ccgtgttttgagTCACATGAcgacagcgccag	5'UTR?<br>intron1	
Drosophila_mojavensis	Dmoj\GI16480		127	733	509	61	417			agtctgctgcaaTCATATGActtgtcgatcga<br>cgcccgccccagTCACATGAcgagctatcagt	5'UTR?<br>intron1	
Drosophila_virilis	Dvir\GJ16024		127	244	509	190	417			gaacagttgcaaTCATATGActtgtcgatcga<br>acgcccgctcggTCACATGActaaatcaagca	5'UTR'<br>intron1	
Drosophila_grimshawi	Dgri\GH17612		127	811	509	63	423			gaagagctaaaaTCATATGActtgtcgatcga<br>tcacagtcagagTCACATGAcaagcaaaacat	5'UTR?<br>intron1	
Paralogs:VhaAC39-2 (CG4624)												
Drosophila_melanogaster	Dmel\VhaAC39-2	77	686			67	367	103				
Drosophila_simulans	Dsim\GD18401		686			59	ND					
Drosophila_sechellia	Dsec\GM23587		686			59	367					
Drosophila_yakuba	Dyak\GE23978		686			58	367					
Drosophila_erecta	Dere\GG12455		686			66	367					
Drosophila_biarmipes	Dbia\VhaAC39-2		686			57	367					
Drosophila_takahashii	Dtak\VhaAC39-2		686			192	367					
Drosophila_suzukii	Dsuz\VhaAC39-2		686			60	367					
Drosophila_eugracilis	Deug\VhaAC39-2		686			57	367					
Drosophila_ficusphila	Dfic\VhaAC39-2		686			59	367					
Drosophila_elegans	Dele\VhaAC39-2		686			84	367					
Drosophila_rhopaloa	Drho\VhaAC39-2		686			121	367					
Drosophila_kikkawai	Dkik\VhaAC39-2		686			57	370					
Drosophila_bipectinata	Dbip\VhaAC39-2		683			62	370					
Drosophila_ananassae	Dana\GF18027		683			51	370					

Drosophila_pseudoobscura	Dpse\GA18311		1047		
Drosophila_persimilis	Dper\GL23045		1047		
Drosophila_miranda	Dmir\VhaAC39-2		1047		
Drosophila_willistoni	Dwil\GK22588		1062		
Drosophila_albomicans	Dalb\VhaAC39-2		ND		
Drosophila_mojavensis	Dmoj\GI10510		1056		
Drosophila_virilis	Dvir\GJ23190		1059		
Drosophila_grimshawi	Dgri\GH21449		1056		

Key: UTR region, CDS region
