# Supplementary material for: Structural and Comparative Analyses of Insects Suggest the Presence of an Ultra-Conserved Regulatory Element of the Genes Encoding Vacuolar-Type ATPase Subunits and Assembly Factors
Source: Biology (Basel). 2023 Aug 13;12(8):1127. doi: 10.3390/biology12081127 (PMC10452791; doi:10.3390/biology12081127)
Supplement: Supplementary file 1 [file biology-12-01127-s001.zip › Supplementary Files S3-S19/Supp_File_S15_Atp6v0e_DROS.pdf]

Gene Cluster ID	Atp6v0e
**Drosophila melanogaster gene**	**VhaM9.7-b (CG7625)**
FlyBase ID	FBgn0028663
**Predicted function**	**ATPase, H+ transporting, lysosomal 9kDa, V0 subunit e**
Protein size	89 amino acids
Cytogenetic map	3L:78D5-78D5
Gene sequence location	3L:21532946..21533798[+]
**Dmel CLEAR sequence**	**TCATGTGA**
CLEAR sequence location	3L:21532984..21532991
Drosophila Atp6v0e paralogs	VhaM9.7-a (CG1268); VhaM9.7-c (CG11589); VhaM9.7-d (CG14909)

FlyBaseVhaM9.7-b (CG7625) gene model & CLEAR element (red) position:

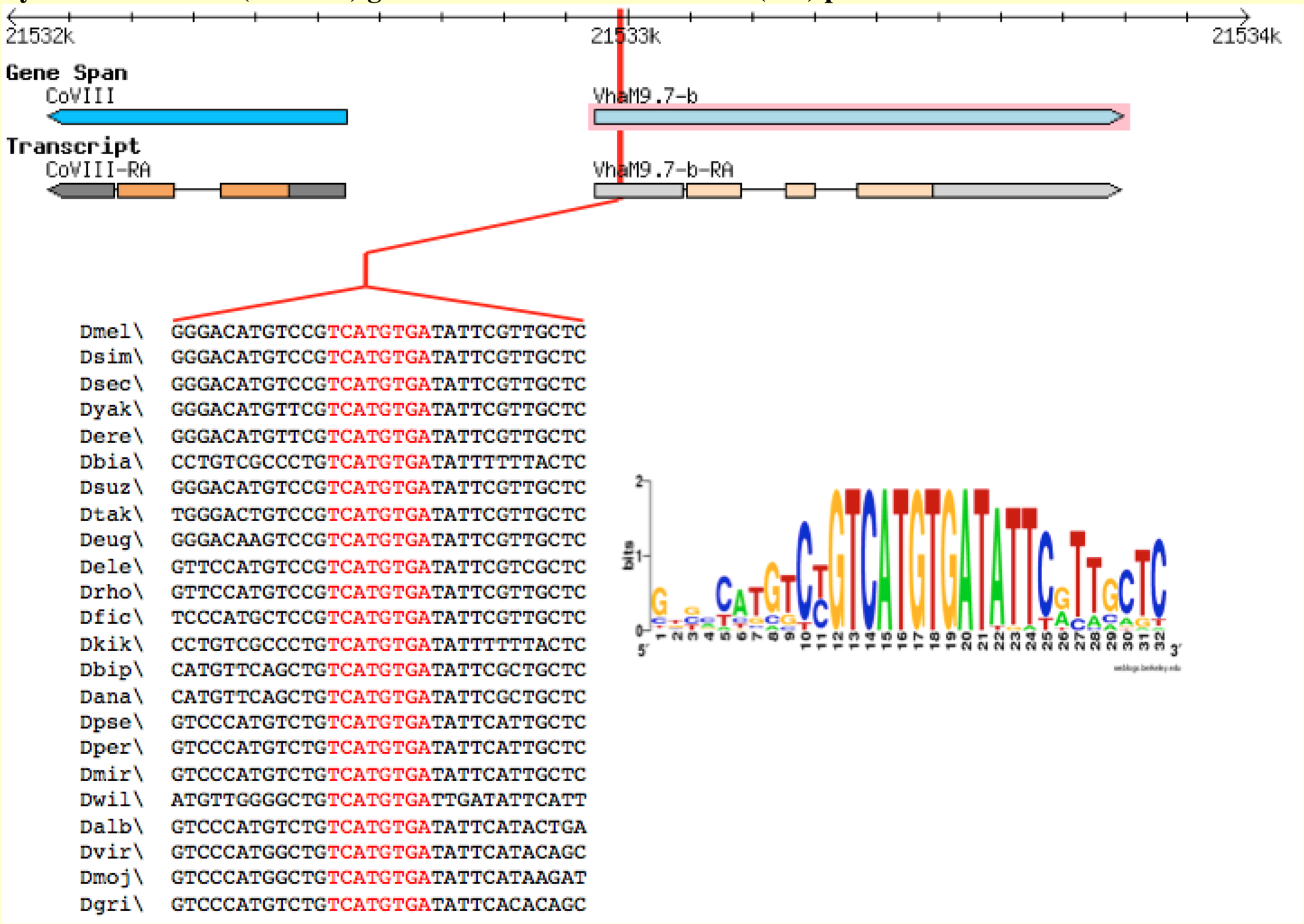

Atp6v0e gene structure comparison:

Key: UTR region, CDS region

Species	Atp6v0e orthologs	5'exon		intron	exon	intron	exon3'			Extended CLEAR region	Position	bps from TSS
Drosophila_melanogaster	Dmel\VhaM9.7-b	142	95	70	48	68	127	303		gggacatgtccgTCATGTGAtattcgttgctc	5'UTR	38

Drosophila_simulans	Dsim\GD14971		95	70	48	105	127			gggacatgtccgTCATGTGAtattcgttgctc	5'UTR?	
Drosophila_sechellia	Dsec\GM22381		95	70	48	105	127			gggacatgtccgTCATGTGAtattcgttgctc	5'UTR?	
Drosophila_yakuba	Dyak\GE19768		95	63	48	101	127			gggacatgttcgTCATGTGAtattcgttgctc	5'UTR?	
Drosophila_erecta	Dere\GG16199		95	58	48	100	127			gggacatgttcgTCATGTGAtattcgttgctc	5'UTR?	
Drosophila_biarmipes	Dbia\VhaM9.7-b		95	53	48	111	127			gggacatgtccgTCATGTGAtattcgttgctc	5'UTR?	
Drosophila_suzukii	Dsuz\VhaM9.7-b		95	51	48	106	127			gggacatgtccgTCATGTGAtattcgttgctc	5'UTR?	
Drosophila_takahashii	Dtak\VhaM9.7-b		95	70	48	102	127			tgggactgtccgTCATGTGAtattcgttgctc	5'UTR?	
Drosophila_eugracilis	Deug\VhaM9.7-b		95	52	48	59	127			gggacaagtccgTCATGTGAtattcgttgctc	5'UTR?	
Drosophila_elegans	Dele\VhaM9.7-b		95	56	48	84	127			gttccatgtccgTCATGTGAtattcgtcgctc	5'UTR?	
Drosophila_rhopaloa	Drho\VhaM9.7-b		95	56	48	78	127			gttccatgtccgTCATGTGAtattcgttgctc	5'UTR?	
Drosophila_ficusphila	Dfic\VhaM9.7-b		95	56	48	78	127			tcccatgctccgTCATGTGAtattcgttgctc	5'UTR?	
Drosophila_kikkawai	Dkik\VhaM9.7-b		95	54	48	72	127			cctgtcgccctgTCATGTGAtattttttactc	5'UTR?	
Drosophila_bipectinata	Dbip\VhaM9.7-b		95	59	48	92	127			catgttcagctgTCATGTGAtattcgctgctc	5'UTR?	
	Dbip\VhaM9.7-b2		270									
Drosophila_ananassae	Dana\GF23725		95	59	48	92	127			catgttcagctgTCATGTGAtattcgctgctc	5'UTR?	
Drosophila_pseudoobscura	Dpse\GA20488		98	54	48	105	127			gtcccatgtctgTCATGTGAtattcattgctc	5'UTR?	
Drosophila_persimilis	Dper\GL11891		98	54	48	105	127			gtcccatgtctgTCATGTGAtattcattgctc	5'UTR?	
Drosophila_miranda	Dmir\VhaM9.7-b		98	54	48	105	127			gtcccatgtctgTCATGTGAtattcattgctc	5'UTR?	
Drosophila_willistoni	Dwil\GK17834		95	81	48	348	127			atgttggggctgTCATGTGAttgatattcatt	5'UTR?	
Drosophila_albomicans	Dalb\VhaM9.7-b		95	65	48	102	127			gtcccatgtctgTCATGTGAtattcatactga	5'UTR?	
Drosophila_mojavensis	Dmoj\Tes122		95	75	48	118	127			gtcccatggctgTCATGTGAtattcataagat	5'UTR?	
Drosophila_virilis	Dvir\GJ13542		95	58	48	123	127			gtcccatggctgTCATGTGAtattcatacagc	5'UTR?	

Drosophila_grimshawi	Dgri\GH14789		95	71	48	114	127			gtcccatgtctgTCATGTGAtattcacacagc	5'UTR?	
Paralog:VhaM9.7-a (CG1268)												
Drosophila_melanogaster	Dmel\VhaM9.7-a	20	258					162				
Drosophila_simulans	Dsim\VhaM9.7-a		258									
Drosophila_sechellia	Dsec\GM14641		258									
Drosophila_yakuba	Dyak\GE21432		258									
Drosophila_erecta	Dere\GG15212		258									
Drosophila_biarmipes	Dbia\VhaM9.7-a		258									
Drosophila_suzukii	Dsuz\VhaM9.7-a		258									
Drosophila_takahashii	Dtak\VhaM9.7-a		258									
Drosophila_eugracilis	Deug\VhaM9.7-a		258									
Drosophila_elegans	Dele\VhaM9.7-a		258									
Drosophila_rhopaloa	Drho\VhaM9.7-a		258									
Drosophila_ficusphila	Dfic\VhaM9.7-a		258									
Drosophila_kikkawai	Dkik\VhaM9.7-a		258									
Drosophila_bipectinata	Dbip\VhaM9.7-a		258									
Drosophila_ananassae	Dana\GF20098		258									
Drosophila_pseudoobscura	Dpse\GA11753		258									
Drosophila_persimilis	Dper\GL12843		258									
Drosophila_miranda	Dmir\VhaM9.7-a		258									
Drosophila_willistoni	Dwil\GK19005		258									
Drosophila_albomicans	Dalb\VhaM9.7-a		261									

Drosophila_mojavensis	Dmoj\GI16589		258					
Drosophila_virilis	Dvir\GJ12841		258					
Drosophila_grimshawi	Dgri\GH15937		258					
Paralog: VhaM9.7-c (CG11589)								
Drosophila_melanogaster	Dmel\VhaM9.7-c		255					
Drosophila_simulans	Dsim\GD13283		255					
Drosophila_sechellia	Dsec\GM14003		255					
Drosophila_yakuba	Dyak\GE20639		255					
Drosophila_erecta	Dere\GG14211		255					
Drosophila_biarmipes	Dbia\VhaM9.7-c		255					
Drosophila_suzukii	Dsuz\VhaM9.7-c		255					
Drosophila_takahashii	Dtak\VhaM9.7-c		255					
Drosophila_eugracilis	Deug\VhaM9.7-c		255					
Drosophila_elegans	Dele\VhaM9.7-c		255					
Drosophila_rhopaloa	Drho\VhaM9.7-c		255					
Drosophila_ficusphila	Dfic\VhaM9.7-c		255					
Drosophila_kikkawai	Dkik\VhaM9.7-c		255					
Drosophila_bipectinata	Dbip\VhaM9.7-c		252					
Drosophila_ananassae	Dana\GF20059		252					
Drosophila_pseudoobscura	Dpse\GA11084		255					
Drosophila_persimilis	Dper\GL12731		255					
Drosophila_miranda	Dmir\VhaM9.7-c		255					
Drosophila_willistoni								

Drosophila_albomicans	Dalb\VhaM9.7-c		258					
Drosophila_mojavensis	Dmoj\GI16823		258					
Drosophila_virilis	Dvir\GJ12571		258					
Drosophila_grimshawi	Dgri\GH15548		279					
Paralog: VhaM9.7-d (CG14909)								
Drosophila_melanogaster	Dmel\VhaM9.7-d	14	267	213				
Drosophila_simulans	Dsim\GD19123		267					
Drosophila_sechellia	Dsec\GM15188		267					
Drosophila_yakuba	Dyak\GE24594		267					
Drosophila_erecta	Dere\GG21840		267					
Drosophila_biarmipes	Dbia\VhaM9.7-d		267					
Drosophila_suzukii	Dsuz\VhaM9.7-d		267					
Drosophila_takahashii	Dtak\VhaM9.7-d		267					
Drosophila_eugracilis	Deug\VhaM9.7-d		267					
Drosophila_elegans	Dele\VhaM9.7-d		267					
Drosophila_rhopaloa	Drho\VhaM9.7-d		267					
Drosophila_ficusphila	Dfic\VhaM9.7-d		267					
Drosophila_kikkawai	Dkik\VhaM9.7-d		267					
Drosophila_bipectinata	Dbip\VhaM9.7-d		267					
Drosophila_ananassae	Dana\GF17072		267					
Drosophila_pseudoobscura	Dpse\GA13345		267					
Drosophila_persimilis	Dper\GL24534		267					

Drosophila_miranda	Dmir\VhaM9.7-d		267					
Drosophila_willistoni	Dwil\GK18963		267					
Drosophila_albomicans								
Drosophila_mojavensis	Dmoj\GI23846		267					
Drosophila_virilis	Dvir\GJ10560		267					
Drosophila_grimshawi	Dgri\GH19550		267					
