# Supplementary material for: Structural and Comparative Analyses of Insects Suggest the Presence of an Ultra-Conserved Regulatory Element of the Genes Encoding Vacuolar-Type ATPase Subunits and Assembly Factors
Source: Biology (Basel). 2023 Aug 13;12(8):1127. doi: 10.3390/biology12081127 (PMC10452791; doi:10.3390/biology12081127)
Supplement: Supplementary file 1 [file biology-12-01127-s001.zip › Supplementary Files S3-S19/Supp_File_S16_Atp6ap1_DROS.pdf]

Gene Cluster ID	Atp6ap1
**Drosophila melanogaster gene**	**VhaAC45 (CG8029)**
FlyBase ID	FBgn0262515
**Predicted function**	**ATPase, H+ transporting, lysosomal accessory protein 1**
Protein size	379 amino acids
Cytogenetic map	2R:45B4-45B4
Gene sequence location	2R:5095067..5097169[+]
**Dmel CLEAR sequence**	**TCACATGA**
CLEAR sequence location	2R:5095055..5095062

FlyBase VhaAC45 (CG8029) gene model & CLEAR element (red) position:

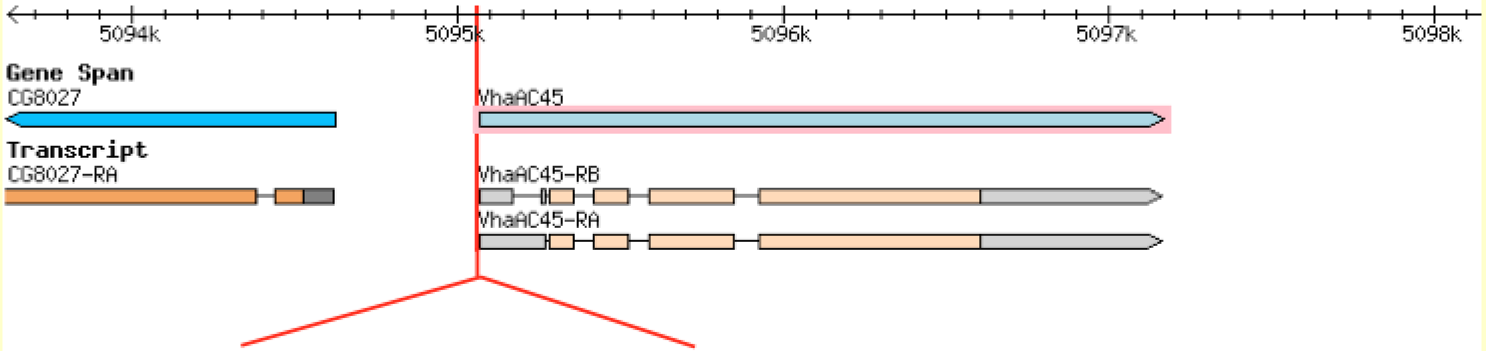


```
Dmel\   AGCTGGTGCGCATCACATGACCGTCATAAGGT
Dsim\   AGCTGGTGCGCATCACATGACCGTCATAAGGT
Dsec\   AGCTGGTGCGCATCACATGACCGTCATAAGGT
Dyak\   AGCTGGTGCGCATCACATGACCGTCATAAGGT
Dere\   AGCTGGTCCGCATCACATGACCGTCATAAGGT
Dbia\   AGCTGGTGCGCATCACATGACCGTCATAAGGT
Dsuz\   AGCTGGTGCGCATCACATGACCGTCATAAGGT
Dtak\   AGCTGGTGCGCATCACATGACCGTCATAAGGT
Deug\   AGCTGGTGTACATCACATGACCGTCATAAGGT
Dele\   AGCTGGTGCGCATCACATGACCGTCATAAGGT
Drho\   AGCTGGTGCGCATCACATGACCGTCATAAGGT
Dfic\   AGCTGGTGCGCATCACATGACCGTCATAAGGT
Dkik\   AGCTGCTGCGCATCACATGACCGTCACAAGTT
Dbip\   GCTGGTCGTCCATCACATGACCGTCATAAGGT
Dana\   GCTGGTCGTCCATCACATGACCGTCATAAGGT
Dpse\   GCGGCCACACGATCACATGATTTGGTCGGTCA
Dper\   GCGGCCACACGATCACATGATTTGGTCGGTCA
Dmir\   GCGGTCACACGATCACATGATTTAGTCGGTCA
Dwil\   TGCCACCTCTAATCACATGACCGTACCGTCAT
Dmoj\   GCTGCTTGGCCATCACATGACCTGTCATATGA
Dvir\   GCTGGTCGCCCATCACATGACCTGTCATATGA
Dgri\   GCTGGTCGGCCATCACATGACATGTCATATGA
```

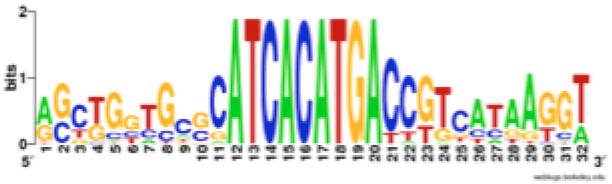

VhaAC45 (CG8029) gene structure comparison in Drosophila species:

Key: UTR region, CDS region

Species	VhaAC45 Atp6ap1 orthologs	5'exon		intron	exon	intron	exon	intron	exon3'			Extended CLEAR region	Position	bps from TSS
Drosophila_melanogaster	Dmel\VhaAC45	203	86	63	103	66	262	72	689	559		agctggtgcgcaTCACATGAccgtcataaggt	5'	-4
Drosophila_simulans	Dsim\GD10644		86	63	103	65	262	73	689			agctggtgcgcaTCACATGAccgtcataaggt	5'	>-206
Drosophila_sechellia	Dsec\GM21109		86	63	103	65	262	73	689			agctggtgcgcaTCACATGAccgtcataaggt	5'	>-206

Drosophila_yakuba	Dyak\GE19265		86	63	103	63	262	73	689			agctggtgcgcaTCACATGAccgtcataaggt	5'	>-206
Drosophila_erecta	Dere\GG23426		86	62	103	63	262	62	689			agctggtccgcaTCACATGAccgtcataaggt	5'	>-206
Drosophila_biarmipes	Dbia\VhaAC45		86	62	103	63	262	61	695			agctggtgcgcaTCACATGAccgtcataaggt	5'	>-208
Drosophila_suzukii	Dsuz\VhaAC45		86	71	103	71	262	74	695			agctggtgcgcaTCACATGAccgtcataaggt	5'	>-208
Drosophila_takahashii	Dtak\VhaAC45		86	61	103	80	262	71	695			agctggtgcgcaTCACATGAccgtcataaggt	5'	>-202
Drosophila_ficusphila	Dfic\VhaAC45		86	63	103	66	262	64	695			agctggtgcgcaTCACATGAccgtcataaggt	5'	>-205
Drosophila_eugracilis	Deug\VhaAC45		86	72	103	82	262	64	677			agctggtgtacaTCACATGAccgtcataaggt	5'	>-203
Drosophila_elegans	Dele\VhaAC45		86	74	103	71	262	71	695			agctggtgcgcaTCACATGAccgtcataaggt	5'	>-205
Drosophila_rhopaloa	Drho\VhaAC45		86	68	103	68	262	63	695			agctggtgcgcaTCACATGAccgtcataaggt	5'	>-203
Drosophila_kikkawai	Dkik\VhaAC45		86	68	103	63	262	67	695			agctgctgcgcaTCACATGAccgtcacaagtt	5'	>-189
Drosophila_bipectinata	Dbip\VhaAC45		86	64	103	55	262	65	686			gctggtcgtccaTCACATGAccgtcataaggt	5'	>-207
Drosophila_ananassae	Dana\GF11814		86	67	103	55	262	65	686			gctggtcgtccaTCACATGAccgtcataaggt	5'	>-207
Drosophila_pseudoobscura	Dpse\GA20777		86	62	103	67	262	62	701			gcggccacacgaTCACATGAtttggtcggtca	5'	>-197
Drosophila_persimilis	Dper\GL17578		86	62	103	67	262	62	701			gcggccacacgaTCACATGAtttggtcggtca	5'	>-197
Drosophila_miranda	Dmir\VhaAC45		86	62	103	67	262	62	701			gcggtcacacgaTCACATGAtttagtcggtca	5'	>-237
Drosophila_willistoni	Dwil\GK17837		89	74	103	376	274	55	704			tgccacctctaaTCACATGAccgtaccgtcat	5'	>-223
Drosophila_albomicans														
Drosophila_mojavensis	Dmoj\GI20366		86	71	103	69	262	70	713			gctgcttggccaTCACATGAcctgtcatatga	5'	>-212
Drosophila_virilis	Dvir\GJ22093		86	58	103	66	262	66	719			gctggtcgcccaTCACATGAcctgtcatatga	5'	>-216
Drosophila_grimshawi	Dgri\GH21865		86	68	103	132	265	436	695			gctggtcggccaTCACATGAcatgtcatatga	5'	>-216
