# Supplementary material for: Structural and Comparative Analyses of Insects Suggest the Presence of an Ultra-Conserved Regulatory Element of the Genes Encoding Vacuolar-Type ATPase Subunits and Assembly Factors
Source: Biology (Basel). 2023 Aug 13;12(8):1127. doi: 10.3390/biology12081127 (PMC10452791; doi:10.3390/biology12081127)
Supplement: Supplementary file 1 [file biology-12-01127-s001.zip › Supplementary Files S3-S19/Supp_File_S17_Atp6ap2_DROS.pdf]

Gene Cluster ID	Atp6ap2
**Drosophila melanogaster gene**	**VhaM8.9 (CG8444)**
FlyBase ID	FBgn0037671
**Predicted function**	**ATPase, H+ transporting, lysosomal accessory protein 2**
Protein size	320 amino acids
Cytogenetic map	3R:85D6-85D7
Gene sequence location	3R:5086363..5088315[+]
**Dmel CLEAR sequence**	**TCACATGA; TCATATGA**
CLEAR sequence location(s)	3R:5086356..5086363; 3R:5086414..5086421

FlyBase VhaM8.9 (CG8444) gene model & CLEAR element position:

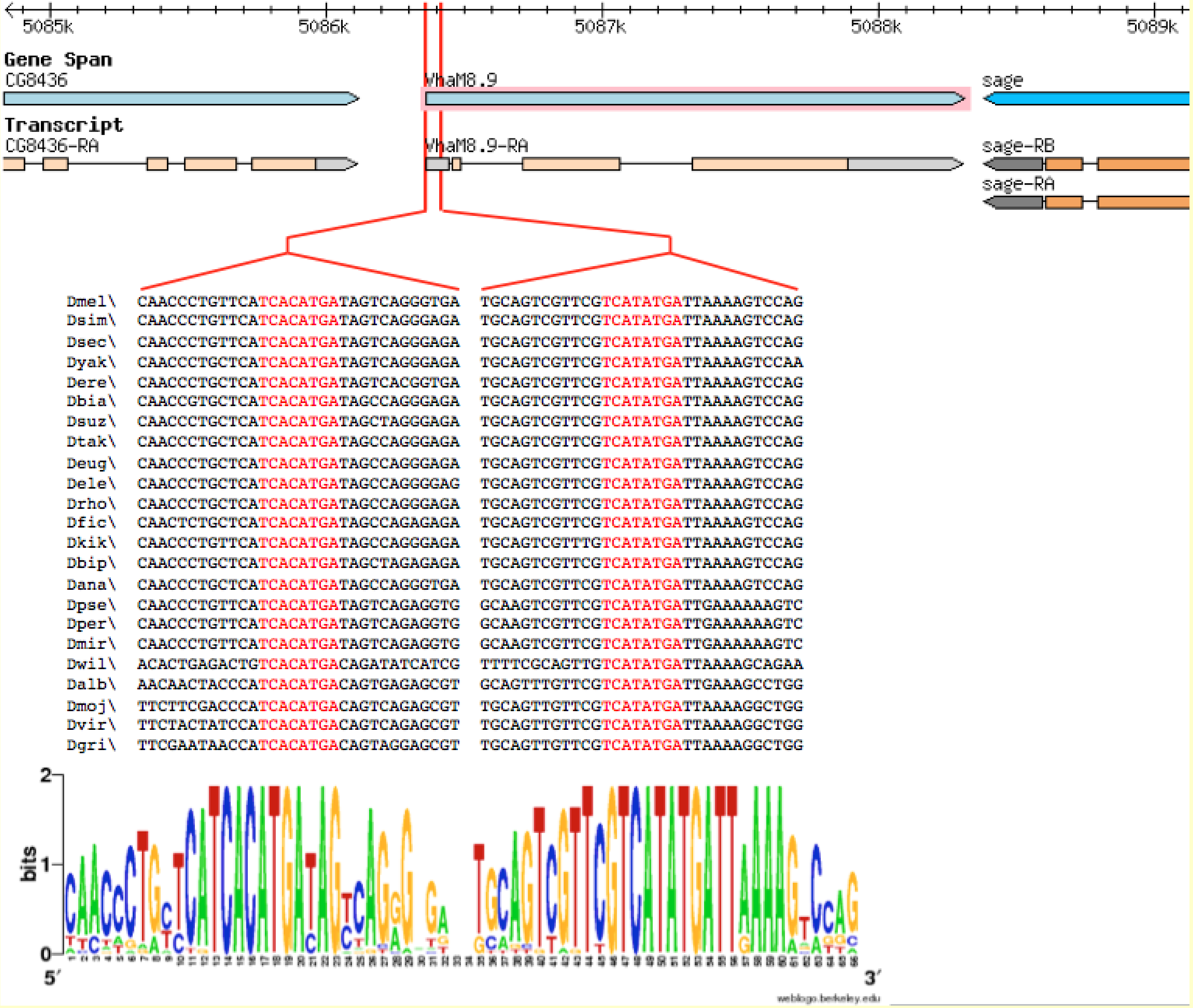

VhaM8.9 (CG8444) gene structure comparison in Drosophila species:

Key: UTR region, CDS region

Species	VhaM8.9 Atp6ap2 orthologs	5'exon		intron	exon	intron	exon3'			Extended CLEAR region	Position	bps from TSS
Drosophila_melanogaster	Dmel\VhaM8.9	84	43	223	353	262	567	421		caaccctgttcaTCACATGAtagtcagggtga<br>tgcagtcgttcgTCATATGAttaaaagtccag	5'<br>5UTR	-1<br>52
Drosophila_simulans	Dsim\GD18602		43	223	353	251	567			caaccctgttcaTCACATGAtagtcagggaga<br>tgcagtcgttcgTCATATGAttaaaagtccag	5'<br>5UTR?	
Drosophila_sechellia	Dsec\GM23791		43	223	353	265	567			caaccctgttcaTCACATGAtagtcagggaga<br>tgcagtcgttcgTCATATGAttaaaagtccag	5'<br>5UTR?	
Drosophila_yakuba	Dyak\GE25938		43	226	353	359	567			caaccctgctcaTCACATGAtagtcagggaga<br>tgcagtcgttcgTCATATGAttaaaagtccaa	5'<br>5UTR?	
Drosophila_erecta	Dere\GG15974		43	217	353	230	567			caaccctgctcaTCACATGAtagtcacggtga<br>tgcagtcgttcgTCATATGAttaaaagtccag	5'<br>5UTR?	
Drosophila_biarmipes	Dbia\VhaM8.9		43	224	353	436	567			caaccgtgctcaTCACATGAtagccagggaga<br>tgcagtcgttcgTCATATGAttaaaagtccag	5'<br>5UTR?	
Drosophila_suzukii	Dsuz\VhaM8.9		43	223	353	541	567			caaccctgctcaTCACATGAtagctagggaga<br>tgcagtcgttcgTCATATGAttaaaagtccag	5'<br>5UTR?	
Drosophila_takahashii	Dtak\VhaM8.9		43	218	353	1418	567			caaccctgctcaTCACATGAtagccagggaga<br>tgcagtcgttcgTCATATGAttaaaagtccag	5'<br>5UTR?	
Drosophila_eugracilis	Deug\VhaM8.9		43	187	353	260	567			caaccctgctcaTCACATGAtagccagggaga<br>tgcagtcgttcgTCATATGAttaaaagtccag	5'<br>5UTR?	
Drosophila_elegans	Dele\VhaM8.9		43	214	353	913	567			caaccctgctcaTCACATGAtagccaggggag<br>tgcagtcgttcgTCATATGAttaaaagtccag	5'<br>5UTR?	
Drosophila_rhopaloa	Drho\VhaM8.9		43	215	353	485	567			caaccctgctcaTCACATGAtagccagggaga<br>tgcagtcgttcgTCATATGAttaaaagtccag	5'<br>5UTR?	
Drosophila_ficusphila	Dfic\VhaM8.9		43	222	353	232	570			caactctgctcaTCACATGAtagccagagaga<br>tgcagtcgttcgTCATATGAttaaaagtccag	5'<br>5UTR?	
Drosophila_kikkawai	Dkik\VhaM8.9		43	235	353	65	570			caaccctgttcaTCACATGAtagccagggaga<br>tgcagtcgtttgTCATATGAttaaaagtccag	5'<br>5UTR?	
Drosophila_bipectinata	Dbip\VhaM8.9		43	207	353	67	567			caaccctgctcaTCACATGAtagctagagaga<br>tgcagtcgttcgTCATATGAttaaaagtccag	5'<br>5UTR?	
Drosophila_ananassae	Dana\GF16587		43	216	353	59	567			caaccctgctcaTCACATGAtagccagggtga<br>tgcagtcgttcgTCATATGAttaaaagtccag	5'<br>5UTR?	
Drosophila_pseudoobscura	Dpse\GA21083		43	226	353	71	567	386		caaccctgttcaTCACATGAtagtcagaggtg<br>gcaagtcgttcgTCATATGAttgaaaaaagtc	5'<br>5UTR?	
Drosophila_persimilis	Dper\GL13947		43	226	353	71	567			caaccctgttcaTCACATGAtagtcagaggtg<br>gcaagtcgttcgTCATATGAttgaaaaaagtc	5'<br>5UTR?	
Drosophila_miranda	Dmir\VhaM8.9		43	226	353	71	567			caaccctgttcaTCACATGAtagtcagaggtg<br>gcaagtcgttcgTCATATGAttgaaaaaagtc	5'<br>5UTR?	

Drosophila_willistoni	Dwil\GK14261		43	225	353	63	573			acactgagactgTCACATGAcagatatcatcg<br>ttttcgcagttgTCATATGAttaaaagcagaa	5'<br>5UTR?	
Drosophila_albomicans	Dalb\VhaM8.9		43	237	353	68	567			aacaactacccaTCACATGAcagtgagagcgt<br>gcagtttgttcgTCATATGAttgaaagcctgg	5'<br>5UTR?	
Drosophila_mojavensis	Dmoj\GI10428		43	237	353	58	564			ttcttcgacccaTCACATGAcagtcagagcgt<br>tgcagttgttcgTCATATGAttaaaaggctgg	5'<br>5UTR?	
Drosophila_virilis	Dvir\GJ10644		43	245	353	64	564			ttctactatccaTCACATGAcagtcagagcgt<br>tgcagttgttcgTCATATGAttaaaaggctgg	5'<br>5UTR?	
Drosophila_grimshawi	Dgri\GH14333		43	231	353	57	567			ttcgaataaccaTCACATGAcagtaggagcgt<br>tgcagttgttcgTCATATGAttaaaaggctgg	5'<br>5UTR?	
