# Supplementary material for: Structural and Comparative Analyses of Insects Suggest the Presence of an Ultra-Conserved Regulatory Element of the Genes Encoding Vacuolar-Type ATPase Subunits and Assembly Factors
Source: Biology (Basel). 2023 Aug 13;12(8):1127. doi: 10.3390/biology12081127 (PMC10452791; doi:10.3390/biology12081127)
Supplement: Supplementary file 1 [file biology-12-01127-s001.zip › Supplementary Files S3-S19/Supp_File_S18_Tmem199_DROS.pdf]

Gene Cluster ID	Tmem199
**Drosophila melanogaster dicistronic gene**	**CG7071**
FlyBase ID	FBgn0260467
**Predicted function**	**ATPase, H+ transporting, vacuolar ER assembly factor, Vma12**
Protein size	302 amino acids
Cytogenetic map	3R:94A6-94A6
Gene sequence location	3R:18188619..18190306 [+]
**Dmel CLEAR sequence**	**TCATGTGA**
CLEAR sequence location	3R:18188617..18188624

NOTE: Dicistronic gene with dicistronic transcript.

FlyBase gene model & CLEAR element (red) position:

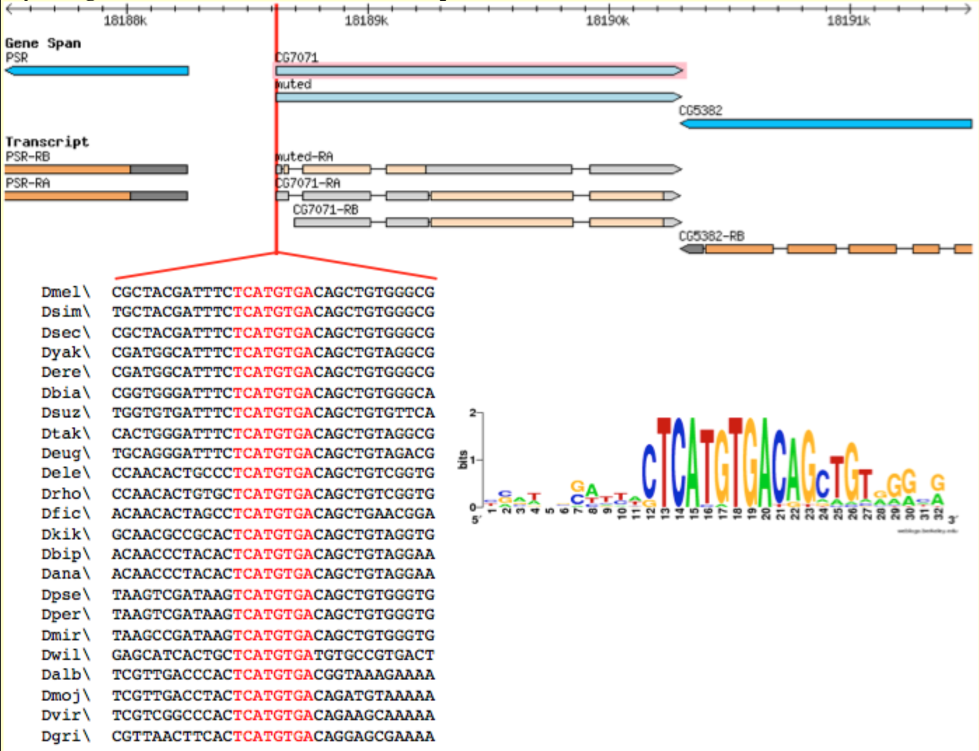

muted/Tmem199 (CG7071) gene structure comparison:

Key: UTR region, CDS region

Species	CG7071 Tmem199 orthologs		muted (CG34131)						CG7071					Extended CLEAR region	Position	bps from TSS
			5'exon	intron	exon	intron	exon3'		5'exon	intron	exon3'					
Drosophila_melanogaster	Dmel\CG7071	28	22	60	281	66	171	5	598	69	311	77		cgctacgatttcTCATGTGAcagctgtgggcg	5'UTR	6
Drosophila_simulans	Dsim\GD20938		22	60	281	66	171	5	598	68	311			tgctacgatttcTCATGTGAcagctgtgggcg	5'UTR?	
Drosophila_sechellia	Dsec\GM26419		22	60	281	66	171	5	598	58	311			cgctacgatttcTCATGTGAcagctgtgggcg	5'UTR?	

Drosophila_yakuba	Dyak\GE10288		22	60	281	66	171	5	604	60	323			cgatggcatttcTCATGTGAcagctgtaggcg	5'UTR?	
Drosophila_erecta	Dere\GG11121		22	60	287	66	171	5	568	61	320			cgatggcatttcTCATGTGAcagctgtgggcg	5'UTR?	
Drosophila_biarmipes	Dbia\CG7071		22	63	272	69	171	5	601	60	323			cggtgggatttcTCATGTGAcagctgtgggca	5'UTR?	
Drosophila_suzukii	Dsuz\CG7071		22	65	272	69	171	5	598	60	323			tggtgtgatttcTCATGTGAcagctgtgttca	5'UTR?	
Drosophila_takahashii	Dtak\CG7071		22	63	272	66	171	5	583	60	326			cactgggatttcTCATGTGAcagctgtaggcg	5'UTR?	
Drosophila_eugracilis	Deug\CG7071		22	60	272	64	171	5	592	60	323			tgcagggatttcTCATGTGAcagctgtagacg	5'UTR?	
Drosophila_elegans	Dele\CG7071		22	59	272	64	171	5	589	63	323			ccaacactgcccTCATGTGAcagctgtcggtg	5'UTR?	
Drosophila_rhopaloa	Drho\CG7071		22	61	272	65	171	4	592	68	323			ccaacactgtgcTCATGTGAcagctgtcggtg	5'UTR?	
Drosophila_ficusphila	Dfic\CG7071		22	61	272	62	165	5	592	57	323			acaacactagccTCATGTGAcagctgaacgga	5'UTR?	
Drosophila_kikkawai	Dkik\CG7071		22	61	272	65	171	8	604	68	317			gcaacgccgcacTCATGTGAcagctgtaggtg	5'UTR?	
Drosophila_bipectinata	Dbip\CG7071		22	63	272	60	171	6	583	63	314			acaaccctacacTCATGTGAcagctgtaggaa	5'UTR?	
Drosophila_ananassae	Dana\GF18081		22	63	272	72	171	6	583	60	314			acaaccctacacTCATGTGAcagctgtaggaa	5'UTR?	
Drosophila_pseudoobscura	Dpse\GA20078		22	61	272	72	171	13	625	63	317			taagtcgataagTCATGTGAcagctgtgggtg	5'UTR?	
Drosophila_persimilis	Dper\GL23377		22	61	272	72	171	13	625	63	317			taagtcgataagTCATGTGAcagctgtgggtg	5'UTR?	
Drosophila_miranda	Dmir\CG7071		22	61	272	72	171	14	625	63	317			taagccgataagTCATGTGAcagctgtgggtg	5'UTR?	
Drosophila_willistoni	Dwil\GK12992		22	61	272	58	171	14	613	61	299			gagcatcactgcTCATGTGAtgtgccgtgact	5'UTR?	
Drosophila_albomicans	Dalb\CG7071		22	177	ND	ND	171	27	589	62	290			tcgttgacccacTCACATGAcggtaaagaaaa	5'UTR?	
Drosophila_mojavensis	Dmoj\GI10478		22	60	272	59	171	13	586	73	290			tcgttgacctacTCATGTGAcagatgtaaaaa	5'UTR?	
Drosophila_virilis	Dvir\GJ23158		22	58	272	68	171	13	577	64	287			tcgtcggcccacTCATGTGAcagaagcaaaaa	5'UTR?	
Drosophila_grimshawi	Dgri\GH21152		22	62	272	69	171	23	589	54	287			cgttaacttcacTCATGTGAcaggagcgaaaa	5'UTR?	
