# Supplementary material for: Structural and Comparative Analyses of Insects Suggest the Presence of an Ultra-Conserved Regulatory Element of the Genes Encoding Vacuolar-Type ATPase Subunits and Assembly Factors
Source: Biology (Basel). 2023 Aug 13;12(8):1127. doi: 10.3390/biology12081127 (PMC10452791; doi:10.3390/biology12081127)
Supplement: Supplementary file 1 [file biology-12-01127-s001.zip › Supplementary Files S3-S19/Supp_File_S19_Vma21_DROS.pdf]

Gene Cluster ID	Vma21
**Drosophila melanogaster gene**	**CG5969**
FlyBase ID	FBgn0036998
**Predicted function**	**Vacuolar ATPase assembly integral membrane protein VMA21-like domain**
Protein size	105 amino acids
Cytogenetic map	3L:77C4-77C4
Gene sequence location	3L:20466897..20467891[+]
**Dmel CLEAR sequence**	**TCATGTGA**[+]
CLEAR sequence location	3L:20466879..20466886

FlyBase CG5969 gene model & CLEAR element (red) position:

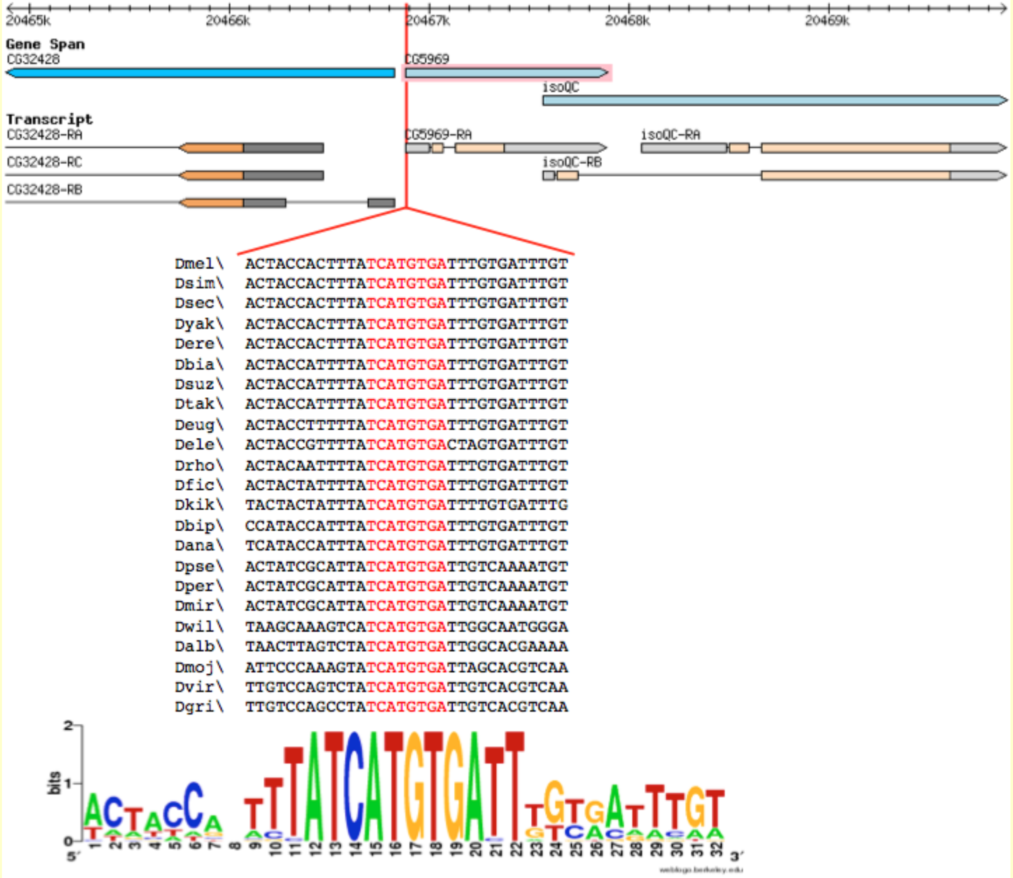

Vma21(CG5969) gene structure comparison in Drosophila species:

Key: UTR region, CDS region

Species	Vma21 orthologs	5'exon	intron	exon3'		Extended CLEAR region	Position	bps from TSS

Drosophila_melanogaster	Dmel\CG5969	101	72	62	246	517		actaccactttaTCATGTGAtttgtgatttgt	5'	-11
Drosophila_simulans	Dsim\GD14890		72	62	246			actaccactttaTCATGTGAtttgtgatttgt	5'	
Drosophila_sechellia	Dsec\GM22297		72	63	246			actaccactttaTCATGTGAtttgtgatttgt	5'	
Drosophila_yakuba	Dyak\GE19686		72	63	246			actaccactttaTCATGTGAtttgtgatttgt	5'	
Drosophila_erecta	Dere\GG16118		72	62	246			actaccactttaTCATGTGAtttgtgatttgt	5'	
Drosophila_biarmipes	Dbia\CG5969		72	62	246			actaccattttaTCATGTGAtttgtgatttgt	5'	
Drosophila_suzukii	Dsuz\CG5969		72	62	246			actaccattttaTCATGTGAtttgtgatttgt	5'	
Drosophila_takahashii	Dtak\CG5969		72	63	246			actaccattttaTCATGTGAtttgtgatttgt	5'	
Drosophila_ficusphila	Dfic\CG5969		72	63	246			actactattttaTCATGTGAtttgtgatttgt	5'	
Drosophila_eugracilis	Deug\CG5969		69	65	246			actaccttttttaTCATGTGAtttgtgatttgt	5'	
Drosophila_elegans	Dele\CG5969		72	70	246			actaccgttttaTCATGTGActagtgatttgt	5'	
Drosophila_rhopaloa	Drho\CG5969		72	62	246			actacaattttaTCATGTGAtttgtgatttgt	5'	
Drosophila_kikkawai	Dkik\CG5969		81	63	246			tactactatttaTCATGTGAttttgtgatttg	5'	
Drosophila_bipectinata	Dbip\CG5969		75	56	246			ccataccatttaTCATGTGAtttgtgatttgt	5'	
Drosophila_ananassae	Dana\GF10347		75	57	246			tcataccatttaTCATGTGAtttgtgatttgt	5'	
Drosophila_pseudoobscura	Dpse\GA19267		81	63	246			actatcgcattaTCATGTGAttgtcaaaatgt	5'	
Drosophila_persimilis	Dper\GL12791		81	63	246			actatcgcattaTCATGTGAttgtcaaaatgt	5'	
Drosophila_miranda	Dmir\CG5969		81	64	246			actatcgcattaTCATGTGAttgtcaaaatgt	5'	
Drosophila_willistoni	Dwil\GK12294		87	62	246			taagcaaagtcaTCATGTGAttggcaatggga	5'	
Drosophila_albomicans	Dalb\CG5969		72	90	252			taacttagtctaTCATGTGAttggcacgaaaa	5'	
Drosophila_mojavensis	Dmoj\GI13399		78	65	240			attcccaaagtaTCATGTGAttagcacgtcaa	5	
Drosophila_virilis	Dvir\GJ11524		78	65	246			ttgtccagtctaTCATGTGAttgtcacgtcaa	5'	
Drosophila_grimshawi	Dgri\GH14552		78	85	243			ttgtccagcctaTCATGTGAttgtcacgtcaa	5'	
