# Supplementary material for: Structural and Comparative Analyses of Insects Suggest the Presence of an Ultra-Conserved Regulatory Element of the Genes Encoding Vacuolar-Type ATPase Subunits and Assembly Factors
Source: Biology (Basel). 2023 Aug 13;12(8):1127. doi: 10.3390/biology12081127 (PMC10452791; doi:10.3390/biology12081127)
Supplement: Supplementary file 1 [file biology-12-01127-s001.zip › Supplementary Files S3-S19/Supp_File_S3_Atp6v1A_DROS.pdf]

Gene Cluster ID	Atp6v1A
**Drosophila melanogaster gene**	**Vha68-2 (CG3762)**
FlyBase ID	FBgn0020367
**Predicted function**	**ATPase, H+ transporting, lysosomal 70kDa, V1 subunit A**
Protein size	614 amino acids
Cytogenetic map	2L:34A3-34A3
Gene sequence location	2L:12,974,322..12,978,607 [+]
**Dmel CLEAR sequence**	**TCAGCTGA; TCAGCTGA; TCACGTGA; TCAGCTGA**
CLEAR sequence location	2L:12974792..12974799; 2L:12975013..12975020; 2L:12975303..12975310; 2L:12975499..12975506
Drosophila Atp6v1A paralogs	Vha68-1 (CG12403); Vha68-3 (CG5075)

FlyBase gene model & CLEAR element (red) positions:

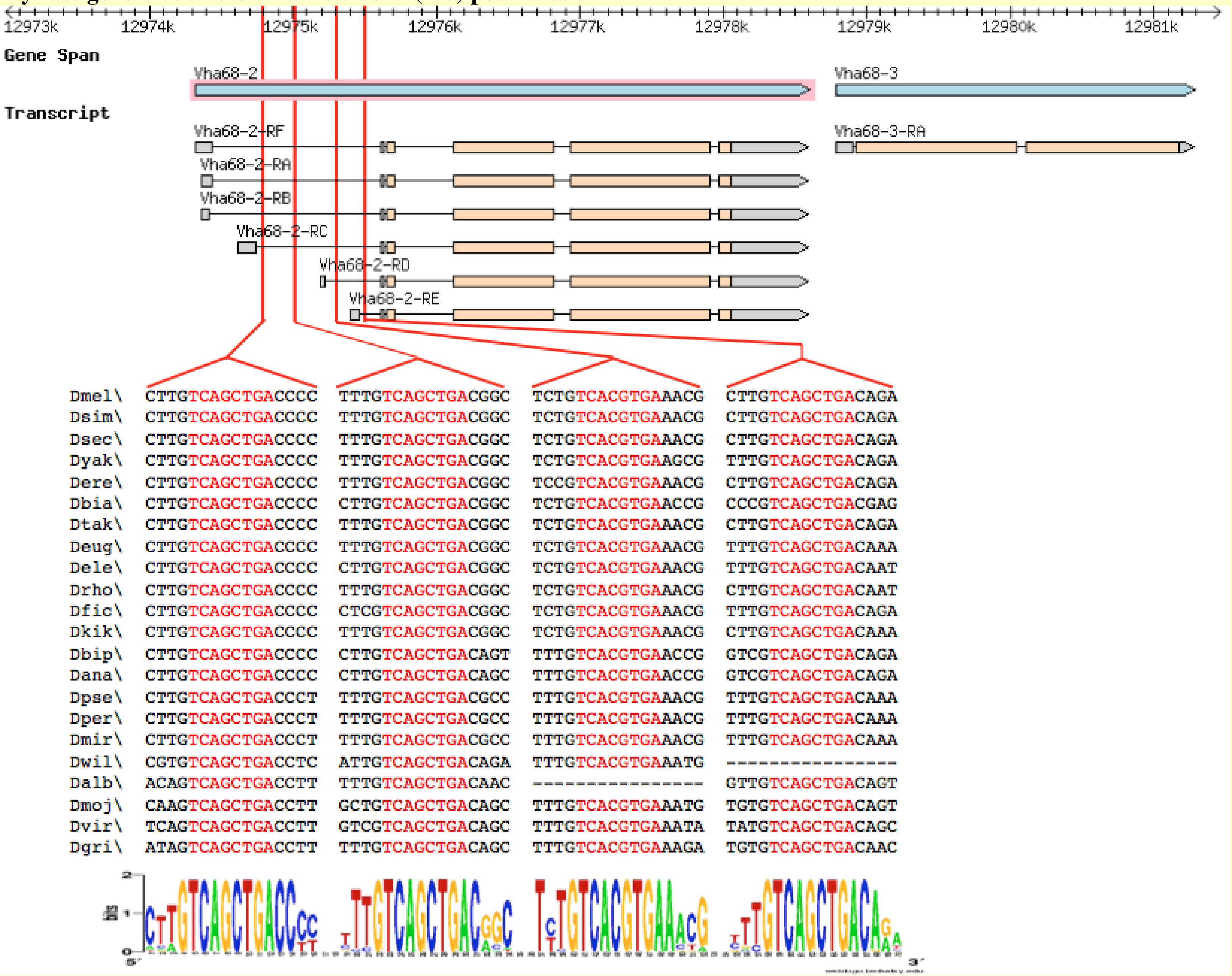

Atp6v1A gene structure comparison:

Key: UTR region, CDS region

Species	Atp6v1A orthologs	5'exon	intron	exon		intron	exon	intron	exon	intron	exon3'			Extended CLEAR region	Position	bps from TSS
Drosophila_melanogaster	Dmel\Vha68-2	124	1170	23	76	413	698	108	978	67	93	546		tattttgccttgTCAGCTGAccccccttccccg<br>ttttttcatttgTCAGCTGAcggcaatgacag<br>ttcaaagctctgTCACGTGAaacgaaagctct<br>acctgcccccttgTCAGCTGAcagagggctgag	intron1<br>intron1<br>intron1<br>intron1	470<br>691<br>981<br>1177
Drosophila_simulans	Dsim\Vha68-2				76	346	698	104	978	67	93			tattttgccttgTCAGCTGAccccccttccccg<br>ttttttcatttgTCAGCTGAcggcaatgacag<br>ttcaaagctctgTCACGTGAaacgaaagctct<br>acctgcccccttgTCAGCTGAcagagggctgag	intron1<br>intron1<br>intron1<br>intron1	
Drosophila_sechellia	Dsec\GM10085				76	357	698	108	978	67	93			tattttgccttgTCAGCTGAccccccttccccg<br>ttttttcatttgTCAGCTGAcggcaatgacag<br>ttcaaagctctgTCACGTGAaacgaaagctct<br>acctgcccccttgTCAGCTGAcagagggctgag	intron1<br>intron1<br>intron1<br>intron1	
Drosophila_yakuba	Dyak\GE18610				76	352	698	111	978	67	93			tattttgccttgTCAGCTGAccccccttccccg<br>ttttttcatttgTCAGCTGAcggcaatgacag<br>ttcaaagctctgTCACGTGAagcgaaagctct<br>acctgccctttgTCAGCTGAcagagggccaag	intron1<br>intron1<br>intron1<br>intron1	
Drosophila_erecta	Dere\GG23804				76	378	698	111	978	69	93			tattttgccttgTCAGCTGAccccccttccccg<br>ttttttcatttgTCAGCTGAcggcaatgacag<br>ttcaaagctccgTCACGTGAaacgaaagctct<br>acctgtcccttgTCAGCTGAcagagggccaag	intron1<br>intron1<br>intron1<br>intron1	
Drosophila_biarmipes	Dbia\Vha68-2				76	440	698	66	978	63	93			tatttcgccttgTCAGCTGAccccccttccccg<br>ctttttcacttgTCAGCTGAcggcaatgacag<br>ttcaaagctctgTCACGTGAaccgaaagctct<br>tcacctgccccgTCAGCTGAcgaggggctaac	intron1<br>intron1<br>intron1<br>intron1	
Drosophila_suzukii	Dsuz\Vha68-2	98	1132	22	76	634	698	67	978	66	93			aattttgccttgTCAGCTGAccccccttccccg<br>ttttttcatttgTCAGCTGAcggcaatgacag<br>ttcaaagctctgTCACGTGAaacgaaagctct<br>cctgcccccctcgTCAGCTGAcgaagggctaac	intron1<br>intron1<br>intron1<br>intron1	430<br>636<br>919<br>1118
Drosophila_takahashii	Dtak\Vha68-2				76	546	698	332	978	65	93			tattttgccttgTCAGCTGAccccccttccccg<br>ttttttcatttgTCAGCTGAcggcaatgacag<br>ttcaaagctctgTCACGTGAaacgaaagctcc<br>cctgcccccttgTCAGCTGAcagagggctaac	intron1<br>intron1<br>intron1<br>intron1	
Drosophila_eugracilis	Deug\Vha68-2				76	427	698	63	978	65	93			tattttgccttgTCAGCTGAcccctttccccg<br>ttttttcatttgTCAGCTGAcggcaatgacag<br>ttcaaagctctgTCACGTGAaacgaaagctct<br>ctgctctctttgTCAGCTGAcaaaactaaacc	intron1<br>intron1<br>intron1<br>intron1	
Drosophila_elegans	Dele\Vha68-2				76	503	698	66	978	66	93			tattttaccttgTCAGCTGAccccccttccccg<br>ttttttcacttgTCAGCTGAcggcaatgacag<br>ttcaaagctctgTCACGTGAaacgaaagctct<br>cctgccctttttgTCAGCTGAcaatggcccaac	intron1<br>intron1<br>intron1<br>intron1	

Drosophila_rhopaloa	Drho\Vha68-2				76	718	698	60	978	67	93			tattttgccttgTCAGCTGAcccccttccccg<br>tttttcatttgTCAGCTGAcggcaatgacag<br>ttaaaagctctgTCACGTGAaacgaaagctct<br>cctgcgctcttgTCAGCTGAcaatgggccaac	intron1<br>intron1<br>intron1<br>intron1	
Drosophila_ficusphila	Dfic\Vha68-2				76	452	698	65	978	67	93			tattttgccttgTCAGCTGAcccccttccccg<br>tttttcgctcgTCAGCTGAcggcaatgacag<br>ttcaaagctctgTCACGTGAaacgaaagctct<br>cctgtttttttgTCAGCTGAcagaagagccaa	intron1<br>intron1<br>intron1<br>intron1	
Drosophila_kikkawai	Dkik\Vha68-2				76	403	1676			68	93			tattttgccttgTCAGCTGAccccgctccccg<br>tttttcatttgTCAGCTGAcggcaatgacag<br>cgcaaagctctgTCACGTGAaacgaaagctct<br>acctgttgcttgTCAGCTGAcaaaagggccaa	intron1<br>intron1<br>intron1<br>intron1	
Drosophila_bipectinata	Dbip\Vha68-2				76	162	698	159	978	65	93			tattttgtcttgTCAGCTGAcccctccctctc<br>tttttccacttgTCAGCTGAcagtggtcttgt<br>cgcaaagctttgTCACGTGAaccgaaagctct<br>tctcgcgtgtcgTCAGCTGAcagaggcccaac	intron1<br>intron1<br>intron1<br>intron1	
Drosophila_ananassae	Dana\GF14214				76	109	698	109	978	63	93			tattttgccttgTCAGCTGAcccctctccctc<br>tttttccacttgTCAGCTGAcagcggtcttgt<br>cgcaaagctttgTCACGTGAaccgaaagctct<br>tctggcccgtcgTCAGCTGAcagaggcgcaac	intron1<br>intron1<br>intron1<br>intron1	
Drosophila_pseudoobscura	Dpse\GA28953				76	62	698	73	978	68	93			tattttgccttgTCAGCTGAccctcccctcgc<br>tttttccatttgTCAGCTGAcgccaatgacag<br>gccaaagctttgTCACGTGAaacgaaagctct<br>cacctcgatttgTCAGCTGAcaaaagggccag	intron1<br>intron1<br>intron1<br>intron1	
Drosophila_persimilis	Dper\Vha68-2				76	62	700?	73	978	68	93			tattttgccttgTCAGCTGAccctcccctcgc<br>tttttccatttgTCAGCTGAcgccaatgacag<br>gccaaagctttgTCACGTGAaacgaaagctct<br>cacctcgatttgTCAGCTGAcaaaagggccag	intron1<br>intron1<br>intron1<br>intron1	
Drosophila_miranda	Dmir\Vha68-2				76	62	698	65	978	68	93			tattttgccttgTCAGCTGAccctcccctcgc<br>tttttcatttgTCAGCTGAcgccaatgacag<br>gccaaagctttgTCACGTGAaacgaaagctct<br>cacttcgatttgTCAGCTGAcaaaagggccag	intron1<br>intron1<br>intron1<br>intron1	
Drosophila_willistoni	Dwil\GK15307				76	70	698	57	978	72	93			ttttctgccgtgTCAGCTGAcctccccctcgc<br>ttcttccaattgTCAGCTGAcagaaaatgaca<br>cgcaaagctttgTCACGTGAaatgaaagctct	intron1<br>intron1<br>intron1<br>intron1	
Drosophila_albomicans	Dalb\Vha68-2				76	68	698?	62	978	170	93			atagttgcacagTCAGCTGAccttccgctcgc<br>tttttccatttgTCAGCTGAcaacatgacagt<br><br>attaacctgttgTCAGCTGAcagtgataagaa	intron1<br>intron1<br>intron1<br>intron1	
Drosophila_mojavensis	Dmoj\GI18088				76	78	698	62	978	68	93			atatttgtcaagTCAGCTGAccttacgcccgt<br>ccagttgtgctgTCAGCTGAcagcatgacagt<br>tgcaaagctttgTCACGTGAaatgaaagctct<br>gcgctctatgtgTCAGCTGAcagtgataagaa	intron1<br>intron1<br>intron1<br>intron1	
Drosophila_virilis	Dvir\GJ16804				76	74	698	58	978	83	93			ataattgttcagTCAGCTGAccttccgcccgc<br>ttccagtcgtcgTCAGCTGAcagcatgacagt<br>tgaaaagctttgTCACGTGAaataaaagctct<br>gccttctatatgTCAGCTGAcagcgataagaa	intron1<br>intron1<br>intron1<br>intron1	

Drosophila_grimshawi	Dgri\GH11385				76	60	698	59	978	61	93			ataattggatagTCAGCTGAccttcccactgc<br>tttttccatttgTCAGCTGAcagcatgacagc<br>cttaaagctttgTCACGTGAaagaaaagctct<br>ctcttcggtgtgTCAGCTGAcaacgataagaa	intron1<br>intron1<br>intron1<br>intron1	
Vha68-1 (CG12403)																
Drosophila_melanogaster	Dmel\Vha68-1				76	263	698	85	978	55	93			-		
Drosophila_simulans	Dsim\GD22093				76	240	698	85	978	55	93					
Drosophila_sechellia	Dsec\GM25943				76	187	698	85	978	55	93					
Drosophila_yakuba	Dyak\GE11978				76	334	698	95	978	58	93			-		
Drosophila_erecta	Dere\GG10233				76	240	698	85	978	58	93			-		
Drosophila_biarmipes	Dbia\Vha68-1				76	217	698	285	978	99	93					
Drosophila_suzukii	Dsuz\Vha68-1			56	76	220	698	423	978	97	93					
Drosophila_takahashii	Dtak\Vha68-1				76	612	698	364	978	55	93			-		
Drosophila_eugracilis	Deug\Vha68-1				76	236	698	87	978	96	93					
Drosophila_elegans	Dele\Vha68-1				76	101	698	679	978	318	93					
Drosophila_rhopaloa	Drho\Vha68-1				76	146	698	141	978	54	93			-		
Drosophila_ficusphila	Dfic\Vha68-1				76	469	698	364	978	83	93					
Drosophila_kikkawai	Dkik\Vha68-1				76	86	698	1178	978	55	93			-		
Drosophila_bipectinata	Dbip\Vha68-1				76	71	698	74	978	80	93			-		
Drosophila_ananassae	Dana\GF15651				76	76	698	66	978	58	93					
Drosophila_pseudoobscura	Dpse\GA11612				76	77	698	70	978	66	93					
Drosophila_persimilis	Dper\GL24783				76	77	698	70	978	66	93			-		
Drosophila_miranda	Dmir\Vha68-1				76	74	698	70	978	66	93					
Drosophila_willistoni	Dwil\GK14815				76	87	698	57	978	59	93			-		
Drosophila_albomicans	Dalb\Vha68-1				76	ND	698	66	978	59	93					
Drosophila_mojavensis	Dmoj\GI21237				76	84	698	62	978	68	93					
Drosophila_virilis	Dvir\GJ11069				76	70	698	58	978	70	93			-		
Drosophila_grimshawi	Dgri\GH10350				76	79	698	59	978	70	93			-		
Vha68-3 (CG5075)																

Drosophila_melanogaster	Dmel\Vha68-3				1149	63	1083					
Drosophila_simulans	Dsim\GD23861*				1149	96	1083					
Drosophila_sechellia	Dsec\GM10088				1149	94	1083					
Drosophila_yakuba	Dyak\GE18611				1149	99	1083					
Drosophila_erecta	Dere\GG23805				1122	165	1083					
Drosophila_eugracilis												
Drosophila_suzukii	Dsuz\Vha-3				1170	1787	1083					
Drosophila_takahashii												
Drosophila_ficusphila	Dfic\Vha68-3				1251	632	1083					
Drosophila_elegans	-											
Drosophila_rhopaloa	Drho\Vha68-3				783	1210	1083					
Drosophila_kikkawai												
Drosophila_biarmipes	-											
Drosophila_bipectinata	Dbip\Vha68-3				1158	108	1083					
Drosophila_ananassae												
Drosophila_pseudoobscura	Dpse\GA18641				1083	65	1089					
Drosophila_persimilis	Dper\GL24733*				1080	65	1089					
Drosophila_miranda	Dmir\Vha68-3				1083	65	1089					
Drosophila_willistoni	Dwil\GK15309				1104	57	1086					
Drosophila_mojavensis												
Drosophila_virilis												
Drosophila_grimshawi	Dgri\GH11386				1161	502	1086					
