# Supplementary material for: Structural and Comparative Analyses of Insects Suggest the Presence of an Ultra-Conserved Regulatory Element of the Genes Encoding Vacuolar-Type ATPase Subunits and Assembly Factors
Source: Biology (Basel). 2023 Aug 13;12(8):1127. doi: 10.3390/biology12081127 (PMC10452791; doi:10.3390/biology12081127)
Supplement: Supplementary file 1 [file biology-12-01127-s001.zip › Supplementary Files S3-S19/Supp_File_S4_Atp6v1B_DROS.pdf]

Gene Cluster ID	Atp6v1B
**Drosophila melanogaster gene**	**Vha55 (CG17369)**
FlyBase ID	FBgn0005671
**Predicted function**	**ATPase, H+ transporting, lysosomal 56/58kDa, V1 subunit B**
Protein size	490 amino acids
Cytogenetic map	3R:87C2-87C3
Gene sequence location	3R:8449484..8453551[-]
**Dmel CLEAR sequence(s)**	**TCATGTGA; TCACATGA; TCAGCTGA; TCATGTGA**
CLEAR sequence location(s)	3R:8453499..8453506; 3R:8452843..8452850; 3R:8452492..8452499; 3R:8452403..8452410

FlyBase Vha55 (CG17369) gene model & CLEAR element (red) positions:

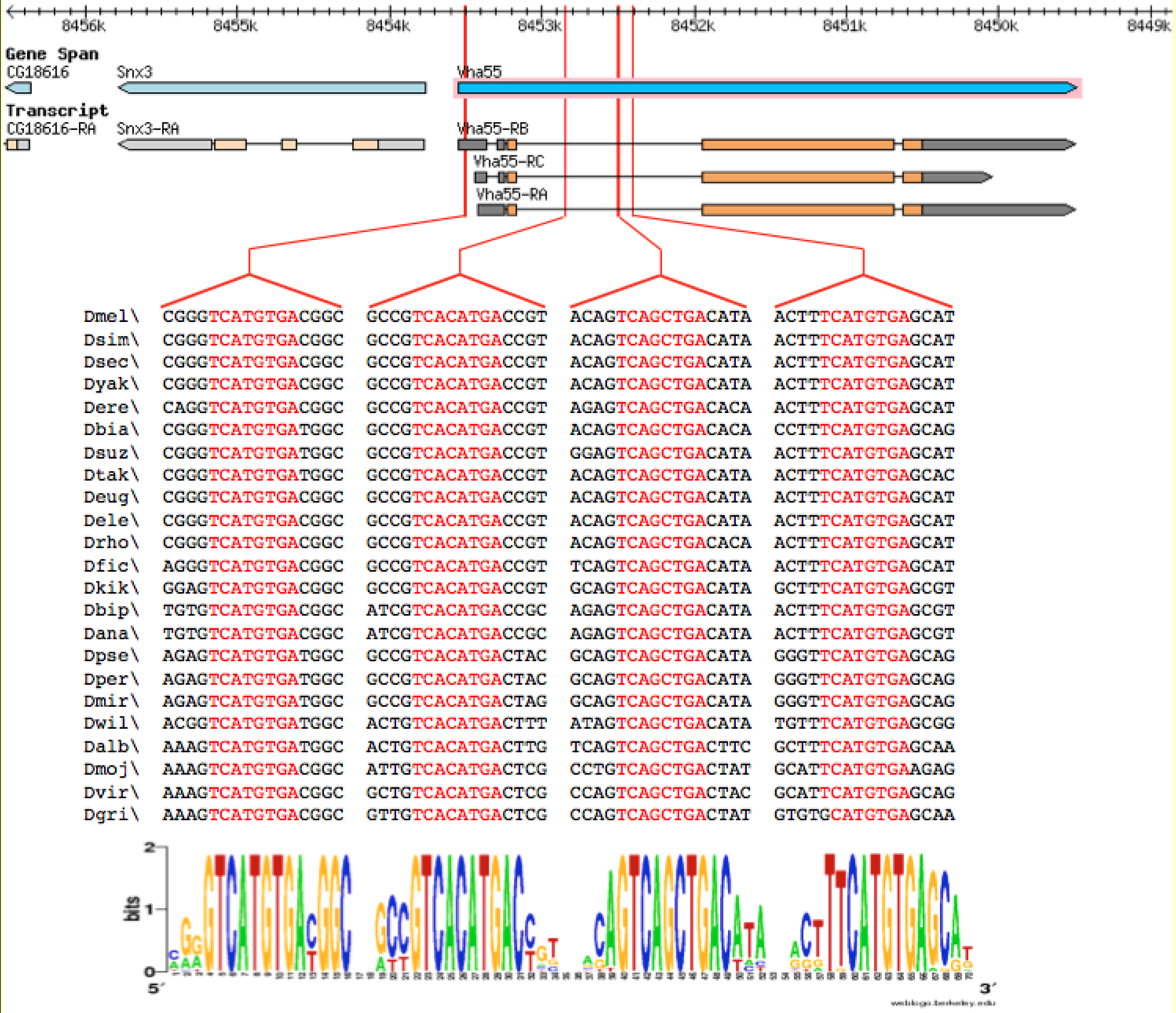

Atp6v1B gene structure comparison:

Key: UTR region, CDS region

Species	Atp6v1B orthologs	5'exon		intron	exon	intron	exon3'			Extended CLEAR region	Position	bps from TSS
Drosophila_melanogaster	Dmel\Vha55	308	73	1221	1262	61	138	1005		acgcgtgacgggTCATGTGAcggcacgtgacg<br>aaattttcgccgTCACATGAccgttcgctgct<br>ccagttggacagTCAGCTGAcataatttagcc<br>agattccgacttTCATGTGAgcattgcgcgac	5'UTR<br>intron1<br>intron1<br>intron1	45<br>701<br>1052<br>1141
Drosophila_simulans	Dsim\GD20555		73	1224	1262	67	138			acgcgtggcgggTCATGTGAcggcacgtgacg<br>aaattttcgccgTCACATGAccgttcgctgct<br>ccagttggacagTCAGCTGAcataatttaccc<br>agattccgacttTCATGTGAgcattgcgcgac	5'UTR?<br>intron1<br>intron1<br>intron1	
Drosophila_sechellia	Dsec\GM25995		73	1243	1262	64	138			acgcgtggcgggTCATGTGAcggcacgtgacg<br>aaattttcgccgTCACATGAccgttcgctgct<br>ccagttggacagTCAGCTGAcataatttaccc<br>agattccgacttTCATGTGAgcattgcgcgac	5'UTR?<br>intron1<br>intron1<br>intron1	
Drosophila_yakuba	Dyak\GE24501		73	1346	1262	60	138			acgcgtggcgggTCATGTGAcggcacgtgacg<br>aaattttcgccgTCACATGAccgttcgctgct<br>ccagttagacagTCAGCTGAcataatttaccc<br>agattccgacttTCATGTGAgcattgcgcgac	5'UTR?<br>intron1<br>intron1<br>intron1	
Drosophila_erecta	Dere\GG17111		73	1320	1262	60	138			acgcgtgtcaggTCATGTGAcggcacgtgacg<br>aaattttcgccgTCACATGAccgttcgctgct<br>ccagttggagagTCAGCTGAcacaatttaccc<br>agattccgacttTCATGTGAgcattgcgcgac	5'UTR?<br>intron1<br>intron1<br>intron1	
Drosophila_biarmipes	Dbia\Vha55		73	1561	1262	60	138			acgcctggcgggTCATGTGAtggcacgtgacg<br>aaattttcgccgTCACATGAccgttcgctgct<br>ccagttggacagTCAGCTGAcacaattaccct<br>agattcccccttTCATGTGAgcagtgcgcgac	5'UTR?<br>intron1<br>intron1<br>intron1	
Drosophila_suzukii	Dsuz\Vha55		73	1525	1262	59	138			acgcctggcgggTCATGTGAtggcacgtgacg<br>aaattttcgccgTCACATGAccgttggctgct<br>aaccagttggagTCAGCTGAcataatttaccc<br>agattcctacttTCATGTGAgcattgcgcgac	5'UTR?<br>intron1<br>intron1<br>intron1	
Drosophila_takahashii	Dtak\Vha55		73	1613	1262	63	138			aacgcctggcgggTCATGTGAtggcacgtgac<br>aaattttcgccgTCACATGAccgttcgctgct<br>ccagttagacagTCAGCTGAcataatttaccc<br>agattcctacttTCATGTGAgcactgcgcgac	5'UTR?<br>intron1<br>intron1<br>intron1	
Drosophila_eugracilis	Deug\Vha55		73	1515	1262	59	138			acgcctgacgggTCATGTGAcggcacgtgacg<br>aaattttcgccgTCACATGAccgttcgctgct<br>ccagttggacagTCAGCTGAcataatttagcc<br>agattcccacttTCATGTGAgcattgcgcgac	5'UTR?<br>intron1<br>intron1<br>intron1	
Drosophila_elegans	Dele\Vha55		73	1645	1262	67	138			gcgcctggcgggTCATGTGAcggcacgtgacg<br>aaattttcgccgTCACATGAccgttcgctgct<br>ccagttagacagTCAGCTGAcataatttttcc<br>agattcttacttTCATGTGAgcattgcgcgac	5'UTR?<br>intron1<br>intron1<br>intron1	

Drosophila_rhopaloa	Drho\Vha55		73	1596	1262	58	138			gcgcctgacgggTCATGTGAcggcacgtgacg<br>aaattttcgccgTCACATGAccgttcgctgct<br>ccagttggacagTCAGCTGAcacaatttaccc<br>agattctaacttTCATGTGAgcattgcgcgac	5'UTR?<br>intron1<br>intron1<br>intron1	
Drosophila_ficusphila	Dfic\Vha55		73	1385	1262	59	138			gcgcctcaagggTCATGTGAcggcacgtgacg<br>aaattttcgccgTCACATGAccgttcgcagct<br>ccagttggtcagTCAGCTGAcataatttacca<br>agattcctacttTCATGTGAgcattgcgagac	5'UTR?<br>intron1<br>intron1<br>intron1	
Drosophila_kikkawai	Dkik\Vha55		73	1272	1400 (1262+138)					acgcctggggagTCATGTGAcggcacgtgacg<br>aaattttcgccgTCACATGAccgttcgctcag<br>ccagttgggcagTCAGCTGAcataatttaccg<br>aaattctcgcttTCATGTGAgcgttgcgcgac	5'UTR?<br>intron1<br>intron1<br>intron1	
Drosophila_bipectinata	Dbip\Vha55		73	1028	1262	68	138			ctgtcgcctgtgTCATGTGAcggcacgtgacg<br>aaattttcatcgTCACATGAccgctagtctct<br>cgagtcggagagTCAGCTGAcatacttctccc<br>agcttcttacttTCATGTGAgcgttgcgcgac	5'UTR?<br>intron1<br>intron1<br>intron1	
Drosophila_ananassae	Dana\GF23001		73	1070	1262	70	138			ctgtcgcctgtgTCATGTGAcggcacgtgacg<br>aaattttcatcgTCACATGAccgctactctct<br>caagtcggagagTCAGCTGAcataatttgccc<br>aacttcttacttTCATGTGAgcgttgcgcgac	5'UTR?<br>intron1<br>intron1<br>intron1	
Drosophila_pseudoobscura	Dpse\GA14484	133+68+86	73	835	1262	67	138	1176		acaaccctagagTCATGTGAtggcacgtgacg<br>aaattttcgccgTCACATGActaccatagatc<br>ccagttgggcagTCAGCTGAcataagttcccc<br>tctccaaggggtTCATGTGAgcaggcggccta	5'UTR<br>intron1<br>intron1<br>intron1	0<br>649<br>960<br>1043
Drosophila_persimilis	Dper\GL13570		73	835	1262	67	138			acaaccctagagTCATGTGAtggcacgtgacg<br>aaattttcgccgTCACATGActaccatagatc<br>ccagttgggcagTCAGCTGAcataagttcccc<br>tctccaaggggtTCATGTGAgcaggcggccta	5'UTR?<br>intron1<br>intron1<br>intron1	
Drosophila_miranda	Dmir\Vha55		73	828	1262	67	138			acaaccctagagTCATGTGAtggcacgtgacg<br>aaattttcgccgTCACATGActagcatagatc<br>ccagttgggcagTCAGCTGAcataagttcccc<br>tctccaaggggtTCATGTGAgcaggcggccta	5'UTR?<br>intron1<br>intron1<br>intron1	
Drosophila_willistoni	Dwil\GK13566		73	789	1262	57	138			gcaaccctacggTCATGTGAtggcacgtgacg<br>aaattttcactgTCACATGActtttgtgttgt<br>gctcgtggatagTCAGCTGAcataatttaccc<br>actggttgtgttTCATGTGAgcggtagaatcc	5'UTR?<br>intron1<br>intron1<br>intron1	
Drosophila_albomicans	Dalb\Vha55		73	959	1262	62	138			tcacacgcaaagTCATGTGAtggcacgtgacg<br>aaattttcactgTCACATGActtgatcgctga<br>ttcgttcgtcagTCAGCTGActtcgttgagtt<br>tggtgtttgcttTCATGTGAgcaacgcttgta	5'UTR?<br>intron1<br>intron1<br>intron1	
Drosophila_mojavensis	Dmoj\GI24752		73	847	1262	68	138			tcacacgtaaagTCATGTGAcggcacgtgacg<br>aaattttcattgTCACATGActcgatcatcaa<br>ccagtttgcctgTCAGCTGActatttaacccc<br>ccacttgtgcatTCATGTGAagagcgctattt	5'UTR?<br>intron1<br>intron1<br>intron1	
Drosophila_virilis	Dvir\GJ14577		73	847	1262	68	138			tcacacgcaaagTCATGTGAcggcacgtgacg<br>aatttttcgctgTCACATGActcgatcgccga<br>gcagtttgccagTCAGCTGActacttaacccc<br>gccgctttgcatTCATGTGAgcagcgttattg	5'UTR?<br>intron1<br>intron1<br>intron1	

Drosophila_grimshawi	Dgri\GH18893		73	862	1262	86	138			tcacacgcaaagTCATGTGAcggcacgtgacg<br>aaattttcgttgTCACATGActcgatcgctga<br>ctggtttgccagTCAGCTGActatttaaccct	5'UTR?<br>intron1<br>intron1<br>intron1	
