# Supplementary material for: Structural and Comparative Analyses of Insects Suggest the Presence of an Ultra-Conserved Regulatory Element of the Genes Encoding Vacuolar-Type ATPase Subunits and Assembly Factors
Source: Biology (Basel). 2023 Aug 13;12(8):1127. doi: 10.3390/biology12081127 (PMC10452791; doi:10.3390/biology12081127)
Supplement: Supplementary file 1 [file biology-12-01127-s001.zip › Supplementary Files S3-S19/Supp_File_S5_Atp6v1C_DROS.pdf]

Gene Cluster ID	Atp6v1C
**Drosophila melanogaster gene**	**Vha44 (CG8048)**
FlyBase ID	FBgn0262511
**Predicted function**	**ATPase, H+ transporting, lysosomal 42kDa, V1 subunit C**
Protein size	388 amino acids (PA)
Cytogenetic map	2R:53B5-53C1
Gene sequence location	2R:12212318..12224142[-]
**Dmel CLEAR sequence**	**TCACGTGA; TCATGTGA**
CLEAR sequence location	2R:12224183..12224190; 2R:12223570..12223577

FlyBase gene model & CLEAR element (red) positions:

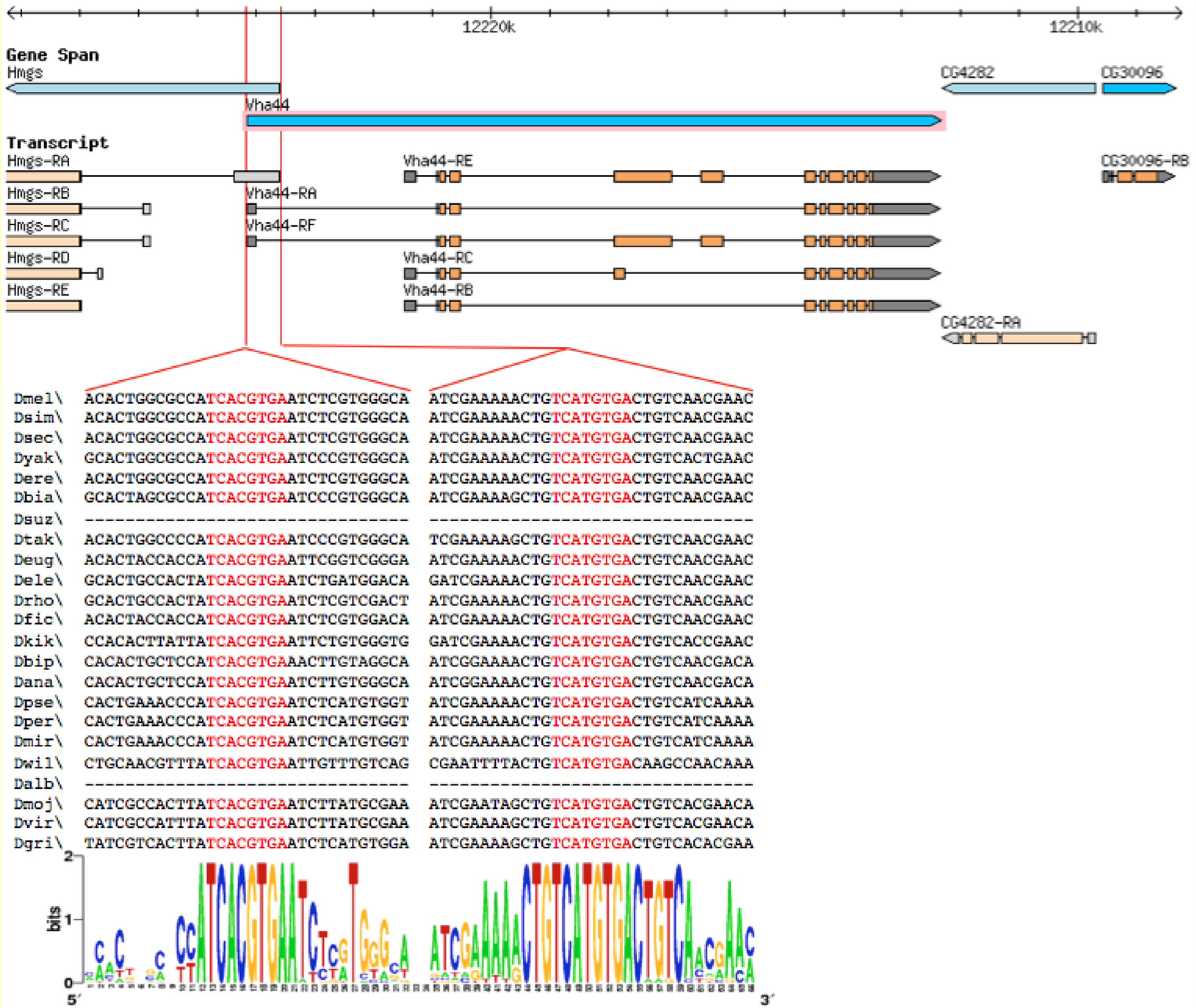

Atp6v1C (Vha44-RA) gene structure comparison:

Key: UTR region, CDS region

Species	Vha44-RA (Atp6v1C) orthologs	5'exon	intron	exon	intron	exon	intron	exon	intron	exon	intron	exon	intron	exon	intron	exon	intron	exon3'		Extended CLEAR region	Position	bps from TSS
Drosophila_melanogaster	Dmel\Vha44	148	3069	8 135	90	154	5873	187	64	99	62	256	62	98	59	139	70	99 1153		acactggcgccaTCACGTGAatctcgtgggca atcgaaaaactgTCATGTGActgtcaacgaac	5' intron1	-40 565

Drosophila_simulans	Dsim\GD25545	ND	ND		135	77	154	5099	187	58	99	63	256	62	98	59	139	67	99	ND		acactggcgccaTCACGTGAatctcgtgggca<br>atcgaaaaactgTCATGTGActgtcaacgaac	5'<br>intron1?	
Drosophila_sechellia	Dsec\GM20063	ND	ND		135	85	154	4304	187	63	99	62	256	63	98	59	139	67	99	ND		acactggcgccaTCACGTGAatctcgtgggca<br>atcgaaaaactgTCATGTGActgtcaacgaac	5'<br>intron1?'	
Drosophila_yakuba	Dyak\GE14069*	ND	ND		135	64	154	5734	187	64	99	62	256	62	98	62	139	67	99	ND		gcactggcgccaTCACGTGAatcccgtgggca<br>atcgaaaaactgTCATGTGActgtcactgaac	5'<br>intron1?	
Drosophila_erecta	Dere\GG22273	ND	ND		135	86	154	5862	187	64	99	60	256	62	98	60	139	67	99	ND		acactggcgccaTCACGTGAatctcgtgggca<br>atcgaaaaactgTCATGTGActgtcaacgaac	5'<br>intron1?	
Drosophila_biarmipes	Dbia\Vha44	ND	ND		135	75	154	5801	187	465	99	60	256	62	98	60	139	65	99	ND		gcactagcgccaTCACGTGAatcccgtgggca<br>atcgaaaagctgTCATGTGActgtcaacgaac	5'<br>intron1?	
Drosophila_suzukii	Dsuz\Vha44	ND	ND		135	118	154	ND	ND	ND	99?	60	256	61	98	63	139	ND	99	ND		ND		
Drosophila_takahashii	Dtak\Vha44	ND	ND		135	344	154	5616	187	579	99	61	256	62	98	62	139	63	99	ND		acactggccccaTCACGTGAatcccgtgggca<br>tcgaaaaagctgTCATGTGActgtcaacgaac	5'<br>intron1?	
Drosophila_eugracilis	Deug\Vha44	ND	ND		135	110	154	5912	187	60	99	59	256	62	98	61	139	67	99	ND		acactaccaccaTCACGTGAattcggtcggga<br>atcgaaaaactgTCATGTGActgtcaacgaac	5'<br>intron1?	
Drosophila_elegans	Dele\Vha44	ND	ND		135	137	154	6020	187	77	99	74	256	70	98	68	139	70	99	ND		gcactgccactaTCACGTGAatctgatggaca<br>gatcgaaaactgTCATGTGActgtcaacgaac	5'<br>intron1?	
Drosophila_rhopaloa	Drho\Vha44	ND	ND		135	181	154	5622	187	68	99	60	256	62	98	64	139	65	99	ND		gcactgccactaTCACGTGAatctcgtcgact<br>atcgaaaaactgTCATGTGActgtcaacgaac	5'<br>intron1?	
Drosophila_ficusphila	Dfic\Vha44	ND	ND		135	134	154	5711	187	59	99	62	256	62	98	61	139	68	99	ND		acactaccaccaTCACGTGAatctcgtggaca<br>atcgaaaaactgTCATGTGActgtcaacgaac	5'<br>intron1?	
Drosophila_kikkawai	Dkik\Vha44	ND	ND		135	57	154	5890	187	67	99	64	256	66	98	65	139	74	99	ND		ccacacttattaTCACGTGAattctgtgggtg<br>gatcgaaaactgTCATGTGActgtcaccgaac	5'<br>intron1?	
Drosophila_bipectinata	Dbip\Vha44	ND	ND		135	131	154	5782	187	62	99	60	256	61	98	63	139	58	99	ND		cacactgctccaTCACGTGAaacttgtaggca<br>atcggaaaactgTCATGTGActgtcaacgaca	5'<br>intron1?	
Drosophila_ananassae	Dana\GF11269	ND	ND		135	60	154	4919	187	61	99	65	256	59	98	61	139	63	99	ND		cacactgctccaTCACGTGAatcttgtgggca<br>atcggaaaactgTCATGTGActgtcaacgaca	5'<br>intron1?	
Drosophila_pseudoobscura	Dpse\GA20788	ND	ND		135	61	154	5678	187	72	99	71	256	67	98	66	139	67	99	ND		cactgaaacccaTCACGTGAatctcatgtggt<br>atcgaaaaactgTCATGTGActgtcatcaaaa	5'<br>intron1?	
Drosophila_persimilis	Dper\GL11349	ND	ND		135	61	154	6037	187	72	99	71	256	67	98	66	139	67	99	ND		cactgaaacccaTCACGTGAatctcatgtggt<br>atcgaaaaactgTCATGTGActgtcatcaaaa	5'<br>intron1?	
Drosophila_miranda	Dmir\Vha44	ND	ND		135	61	154	5925	187	72	99	71	256	67	98	66	139	67	99	ND		cactgaaacccaTCACGTGAatctcatgtggt<br>atcgaaaaactgTCATGTGActgtcatcaaaa	5'<br>intron1?	
Drosophila_willistoni	Dwil\GK21692	ND	ND		135	65	154	8442	187	76	99	58	256	75	237			70	99	ND		ctgcaacgtttaTCACGTGAattgtttgtcag<br>cgaattttactgTCATGTGAcaagccaacaaa	5'<br>intron1?	
Drosophila_albomicans	Dalb\Vha44	ND	ND		132	68	154	6951	187	97	99	71	256	71	98	85	139	65	99	ND		ND		
Drosophila_mojavensi	Dmoj\GI19629	ND	ND		132	63	154	6772	187	77	99	77	256	63	98	268	139	58	99	ND		catcgccacttaTCACGTGAatcttatgcgaa<br>atcgaatagctgTCATGTGActgtcacgaaca	5'<br>intron1?	
Drosophila_virilis	Dvir\GJ14996	ND	ND		135	65	154	6765	187	63	99	137	256	62	98	62	139	56	99	ND		catcgccatttaTCACGTGAatcttatgcgaa<br>atcgaaaagctgTCATGTGActgtcacgaaca	5'<br>intron1?	
Drosophila_grimshawi	Dgri\GH21923	ND	ND		135	70	154	6549	187	84	99	103	256	75	98	61	142	63	99	ND		tatcgtcacttaTCACGTGAatctcatgtgga<br>atcgaaaagctgTCATGTGActgtcacacgaa	5'<br>intron1?	
