# Supplementary material for: Structural and Comparative Analyses of Insects Suggest the Presence of an Ultra-Conserved Regulatory Element of the Genes Encoding Vacuolar-Type ATPase Subunits and Assembly Factors
Source: Biology (Basel). 2023 Aug 13;12(8):1127. doi: 10.3390/biology12081127 (PMC10452791; doi:10.3390/biology12081127)
Supplement: Supplementary file 1 [file biology-12-01127-s001.zip › Supplementary Files S3-S19/Supp_File_S6_Atp6v1D_DROS.pdf]

Gene Cluster ID	Atp6v1D
**Drosophila melanogaster gene**	**Vha36-1 (CG8186)**
FlyBase ID	FBgn0022097
**Predicted function**	**ATPase, H+ transporting, V1 subunit D**
Protein size	246 amino acids
Cytogenetic map	2R:52A10-52A10
Gene sequence location	2R:11428922..11430003[+]
**Dmel CLEAR sequence**	**TCATCTGA; TCATATGA**
CLEAR sequence location	2R:11430063..11430070; 2R:11430114..11430121
Drosophila ATP6v1D paralogs	Vha36-2; Vha36-3

FlyBase Vha36-1 gene model & CLEAR element (red) position:

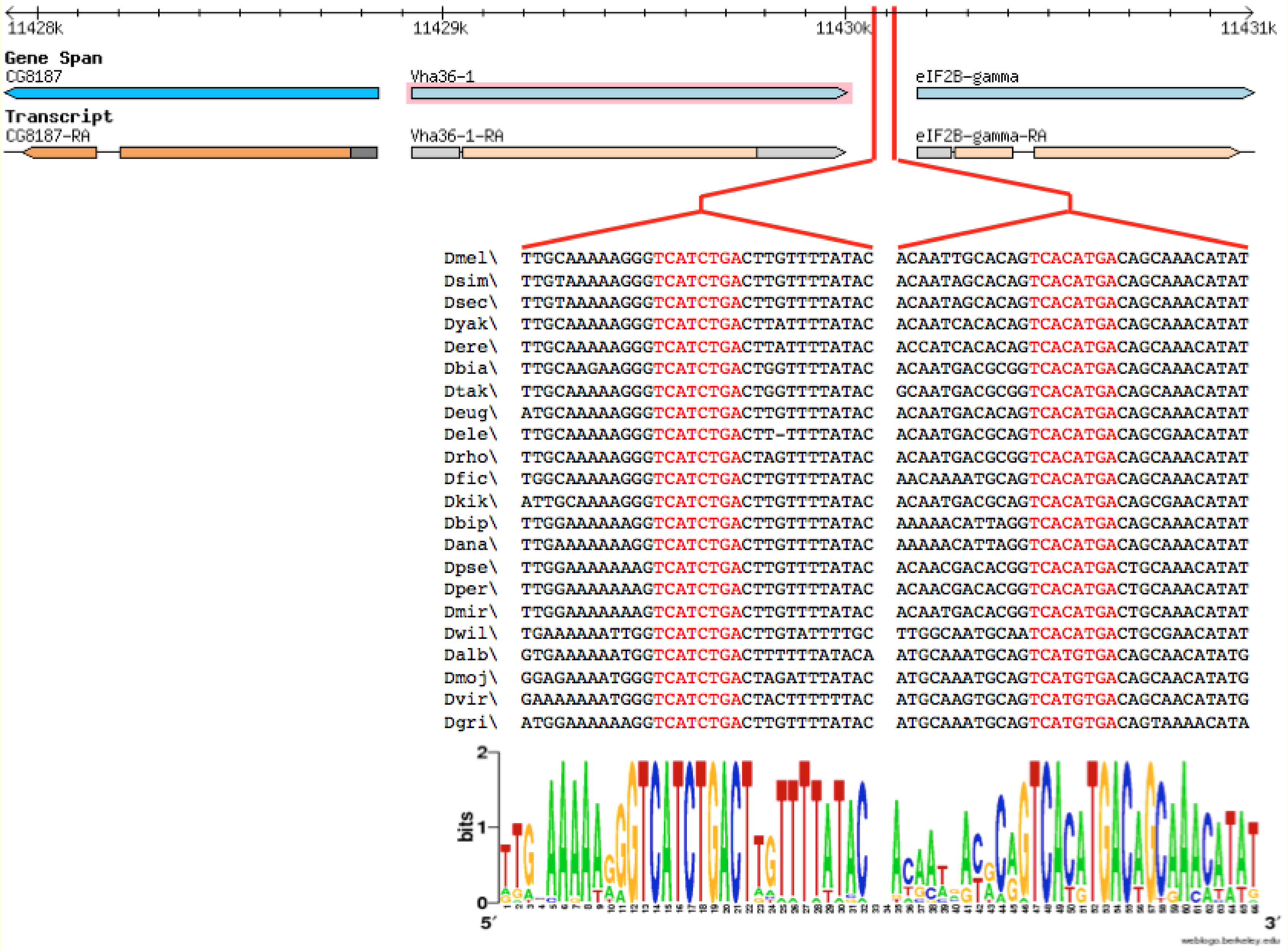

Atp6v1D gene structure comparison:

Key: UTR region, CDS region

Species	Atp6v1D orthologs	5'exon3'			Extended CLEAR region	Position	bps from TSS
Drosophila_melanogaster	Dmel\Vha36-1	120	741	221	ttgcaaaaagggTCATCTGActtgttttatac<br>acaattgcacagTCACATGAcagcaaacatat	3'<br>3'	1142<br>1193
Drosophila_simulans	Dsim\GD11125*	99	741	180	ttgtaaaaagggTCATCTGActtgttttatac<br>acaatagcacagTCACATGAcagcaaacatat	3'<br>3'	1122<br>1173
Drosophila_sechellia	Dsec\GM21623*	97	741	178	ttgtaaaaagggTCATCTGActtgttttatac<br>acaatagcacagTCACATGAcagcaaacatat	3'<br>3'	1119<br>1170
Drosophila_yakuba	Dyak\GE11717		741		ttgcaaaaagggTCATCTGActtattttatac<br>acaatcacacagTCACATGAcagcaaacatat	3'<br>3'	>1023<br>>1073
Drosophila_erecta	Dere\GG20533		741		ttgcaaaaagggTCATCTGActtattttatac<br>accatcacacagTCACATGAcagcaaacatat	3'<br>3'	>1022<br>>1073
Drosophila_biarmipes	Dbia\Vha36-1		741		ttgcaagaagggTCATCTGActggttttatac<br>acaatgacgcggTCACATGAcagcaaacatat	3'<br>3'	>1050<br>>1101
Drosophila_suzukii	Dsuz\Vha36-1		741		ttgcaaaaagggTCATCTGActtgttttatac<br>acaatgacgcggTCACATGAcagcaaacatat	3'<br>3'	>1037<br>>1088
Drosophila_takahashii	Dtak\Vha36-1		741		ttgcaaaaagggTCATCTGActggttttatac<br>gcaatgacgcggTCACATGAcagcaaacatat	3'<br>3'	>1039<br>>1090
Drosophila_eugracilis	Deug\Vha36-1		741		atgcaaaaagggTCATCTGActtgttttatac<br>acaatgacacagTCACATGAcagcaaacatat	3'<br>3'	>1027<br>>1078
Drosophila_elegans	Dele\Vha36-1		741		ttgcaaaaagggTCATCTGActtttttataca<br>acaatgacgcagTCACATGAcagcgaacatat	3'<br>3'	>1023<br>>1073
Drosophila_rhopaloa	Drho\Vha36-1		741		ttgcaaaaagggTCATCTGActagttttatac<br>acaatgacgcggTCACATGAcagcaaacatat	3'<br>3'	>1021<br>>1072
Drosophila_ficusphila	Dfic\Vha36-1		741		tggcaaaaagggTCATCTGActtgttttatac<br>aacaaaatgcagTCACATGAcagcaaacatat	3'<br>3'	>1022<br>>1071
Drosophila_kikkawai	Dkik\Vha36-1		741		attgcaaaagggTCATCTGActtgttttatac<br>acaatgacgcagTCACATGAcagcgaacatat	3'<br>3'	>1027<br>>1078
Drosophila_bipectinata	Dbip\Vha36-1		741		ttggaaaaaaggTCATCTGActtgttttatac<br>aaaaacattaggTCACATGAcagcaaacatat	3'<br>3'	>1030<br>>1070
Drosophila_ananassae	Dana\GF11142	87	741		ttgaaaaaaaggTCATCTGActtgttttatac<br>aaaaacattaggTCACATGAcagcaaacatat	3'<br>3'	1116<br>1157
Drosophila_pseudoobscura	Dpse\GA20878	81	741		ttggaaaaaaagTCATCTGActtgttttatac<br>acaacgacacggTCACATGActgcaaacatat	3'<br>3'	1078<br>1128
Drosophila_persimilis	Dper\GL20077		741		ttggaaaaaaagTCATCTGActtgttttatac<br>acaacgacacggTCACATGActgcaaacatat	3'<br>3'	>999<br>>1048
Drosophila_miranda	Dmir\Vha36-1		741		ttggaaaaaaagTCATCTGActtgttttatac<br>acaatgacacggTCACATGActgcaaacatat	3'<br>3'	>999<br>1048
Drosophila_willistoni	Dwil\GK23028	93	744	148	tgaaaaaattggTCATCTGActtgtattttgc<br>ttggcaatgcaaTCACATGActgcgaacatat	3'<br>3'	1147<br>1180

Drosophila_albomicans	Dalb\Vha36-1		741			gtgaaaaaatggTCATCTGActttttataca<br>atgcaaatgcagTCATGTGAcagcaacatatg	3'<br>3'	>1055<br>>1099
Drosophila_mojavensis	Dmoj\GI20255	65	741			ggagaaaatgggTCATCTGActagatttatac<br>atgcaaatgcagTCATGTGAcagcaacatatg	3'<br>3'	1132<br>1177
Drosophila_virilis	Dvir\GJ20208	84	747			gaaaaaaatgggTCATCTGActactttttac<br>atgcaagtgcagTCATGTGAcagcaacatatg	3'<br>3'	1156<br>1201
Drosophila_grimshawi	Dgri\GH19969	76	741			atggaaaaaaggTCATCTGActtgttttatac<br>atgcaaatgcagTCATGTGAcagtaaaacata	3'<br>3'	1134<br>1179

Paralogs: Vha36-2 (CG13167)

Species	Vha36-2 orthologs	5'exon		intron	exon3'	
Drosophila_melanogaster	Dmel\Vha36-2	73	525	75	597	101
Drosophila_simulans	Dsim\GD10848		525	53	558	
Drosophila_sechellia	Dsec\GM21350		525	53	558	
Drosophila_yakuba	Dyak\GE12423		525	55	594	
Drosophila_erecta	Dere\GG20264		525	63	621	
Drosophila_biarmipes	Dbia\Vha36-2		525	443	597	
Drosophila_suzukii	Dsuz\Vha36-2		525	333	660	
Drosophila_takahashii	Dtak\Vha36-2		525	440	594	
Drosophila_eugracilis	Deug\Vha36-2		525	63	558	
Drosophila_elegans	Dele\Vha36-2		525	1706	564	
Drosophila_rhopaloa	Drho\Vha36-2		525	284	555	
Drosophila_ficusphila	Dfic\Vha36-2		525	233	465	
Drosophila_kikkawai	Dkik\Vha36-2		531	64	441	
Drosophila_bipectinata	Dbip\Vha36-2		1059			
Drosophila_ananassae	Dana\GF12834		1047			
Drosophila_pseudoobscura	Dpse\GA12092		1020			
Drosophila_persimilis	Dper\GL10813		1020			
Drosophila_miranda	Dmir\Vha36-2		1020			
Drosophila_willistoni	Dwil\GK22115		1077			
Drosophila_albomicans	Dalb\Vha36-2		1002			
Drosophila_mojavensis	Dmoj\GI18533		975			
Drosophila_virilis	Dvir\GJ21402		999			

Drosophila_grimshawi	Dgri\GH22755		1023	

Paralogs: Vha36-3 (CG8310)

Species	Vha36-3 orthologs	5'exon		intron	exon	intron	exon	intron	exon3'	
Drosophila_melanogaster	Dmel\Vha36-3	83	41	60	118	55	364	61	227	45
Drosophila_simulans	Dsim\Vha36-3		41	60	118	64	364	58	227	
Drosophila_sechellia	Dsec\Vha36-3		ND	ND	118	64	364	56	227	
Drosophila_yakuba	Dyak\Vha36-3		41	65	118	65	>266	ND	ND	
Drosophila_erecta	Dere\GG12609		41	72	118	61	364	68	227	
Drosophila_biarmipes	Dbia\Vha36-3		41	76	118	66	364	119	227	
Drosophila_suzukii	Dsuz\Vha36-3		41	68	118	64	364	147	227	
Drosophila_takahashii	Dtak\Vha36-3		41	63	118	58	364	299	230	
Drosophila_eugracilis	Deug\Vha36-3		41	59	118	56	364	52	227	
Drosophila_elegans	Dele\Vha36-3		41	58	118	56	364	492	227	
Drosophila_rhopaloa	Drho\Vha36-3		41	65	118	59	364	316	227	
Drosophila_ficusphila	Dfic\Vha36-3		41	62	118	56	364	59	227	
Drosophila_kikkawai	Dkik\Vha36-3		41	66	118	58	364	74	230	
Drosophila_bipectinata	Dbip\Vha36-3		41	53	118	62	364	61	227	
Drosophila_ananassae	Dana\GF21946		41	58	118	57	364	57	227	
Drosophila_pseudoobscura	Dpse\GA20975		41	59	118	54	364	58	227	
Drosophila_persimilis	Dper\GL14446		41	59	118	54	364	58	227	
Drosophila_miranda	Dmir\Vha36-3		41	59	118	54	364	58	227	
Drosophila_willistoni	Dwil\GK24913		41	59	118	72	364	83	230	
Drosophila_albomicans	Dalb\Vha36-3		41	68	118	89	364	69	230	
Drosophila_mojavensis	Dmoj\GI15892		41	78	118	85	364	57	230	
Drosophila_virilis	Dvir\GJ16639		41	74	118	63	364	105	230	
Drosophila_grimshawi	Dgri\GH12748		41	129	118	71	364	95	230	
