# Supplementary material for: Structural and Comparative Analyses of Insects Suggest the Presence of an Ultra-Conserved Regulatory Element of the Genes Encoding Vacuolar-Type ATPase Subunits and Assembly Factors
Source: Biology (Basel). 2023 Aug 13;12(8):1127. doi: 10.3390/biology12081127 (PMC10452791; doi:10.3390/biology12081127)
Supplement: Supplementary file 1 [file biology-12-01127-s001.zip › Supplementary Files S3-S19/Supp_File_S7_Atp6v1E_DROS.pdf]

Gene Cluster ID	Atp6v1E
**Drosophila melanogaster gene**	**Vha26 (CG1088)**
FlyBase ID	FBgn0015324
**Predicted function**	**ATPase, H+ transporting, lysosomal 31kDa, V1 subunit E**
Protein size	226 amino acids
Cytogenetic map	3R:83B4-83B4
Gene sequence location	3R:1417453..1420429[+]
**Dmel CLEAR sequences**	**TCATATGA; TCATGTGA; TCATGTGA; TCACACGA; TCATGTGA**
CLEAR sequence locations	3R:1417501..1417508; 3R:1417899..1417906; 3R:1418066..1418073; 3R:1418145..1418152; 3R:1418246..1418253

FlyBase Vha26 (CG1088) gene model & CLEAR element (red) positions:

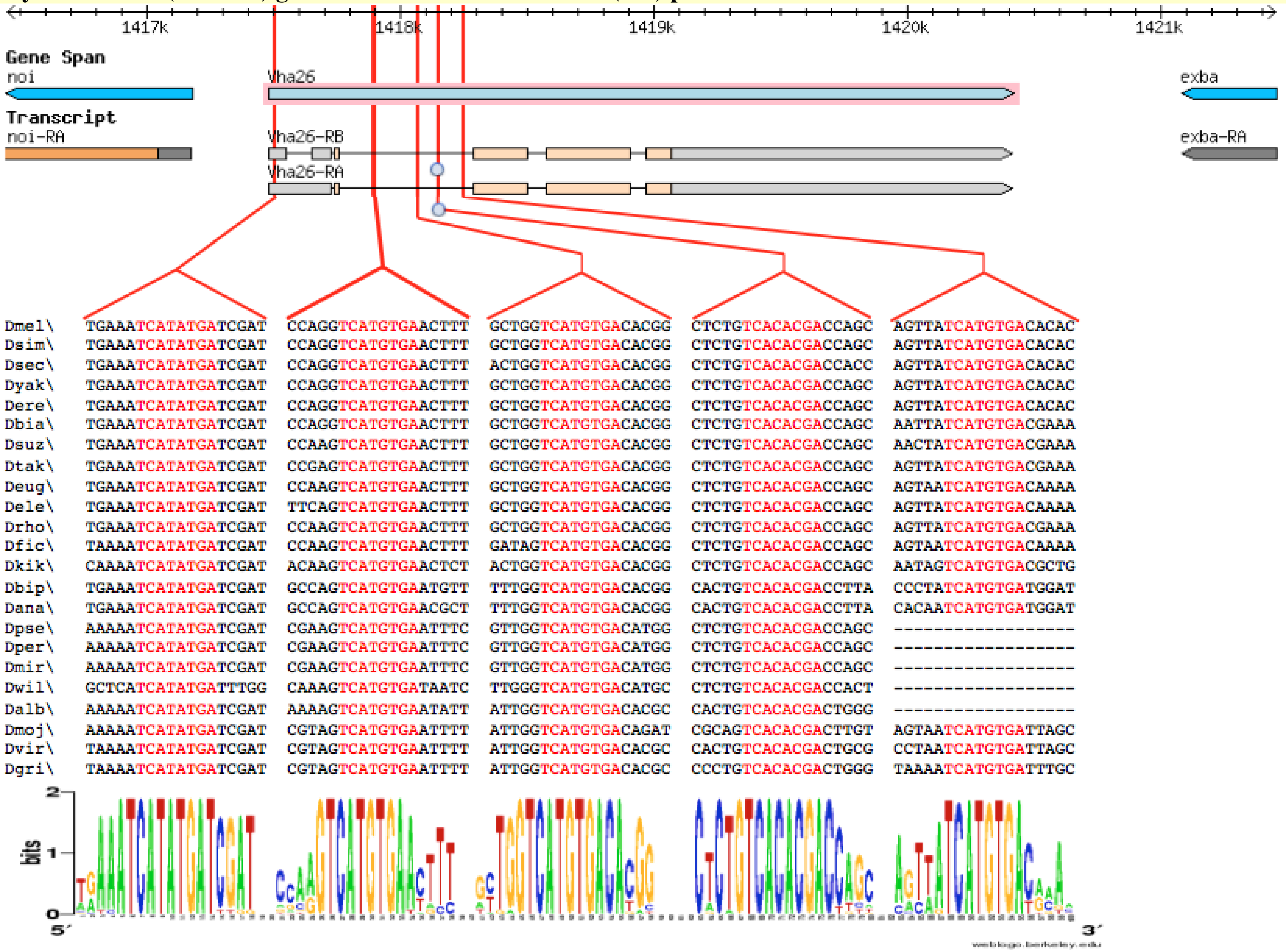

Atp6v1E gene structure comparison:

Key: UTR region, CDS region

Species	Vha26 Atp6v1E orthologs	5'exon		intron	exon	intron	exon	intron	exon3'			Extended CLEAR region	Position	bps from TSS
Drosophila_melanogaster	Dmel\Vha26	275	33	529	213	78	327	61	108	1353		actttcgtgaaaTCATATGAtcgatttgcagt<br>ggatctcccaggTCATGTGAacttttcaccgc<br>aattcgtgctggTCATGTGAcacggcccccgc<br>catgtccagttaTCATGTGAcacacaggcaac	5'UTR?<br>intron1<br>intron1<br>intron1	48<br>447<br>614<br>794
Drosophila_simulans	Dsim\GD19797		33	514	213	76	327	61	108			actttcgtgaaaTCATATGAtcgatttgcagt<br>gtatctcccaggTCATGTGAacttttcaccgc<br>aattcgtgctggTCATGTGAcacggcccccgc<br>catgtccagttaTCATGTGAcacacaggcaac	5'UTR?<br>intron1<br>intron1<br>intron1	
Drosophila_sechellia	Dsec\GM10818		33	520	213	76	327	61	108			actttcgtgaaaTCATATGAtcgatttgcagt<br>ggatctcccaggTCATGTGAacttttcattgc<br>aattcgtactggTCATGTGAcacggcccccgc<br>catgtccagttaTCATGTGAcacacaggcaac	5'UTR?<br>intron1<br>intron1<br>intron1	
Drosophila_yakuba	Dyak\GE10141		33	499	213	69	327	61	108			actttcgtgaaaTCATATGAtcgatttgcagt<br>ggatctcccaggTCATGTGAacttttcaccgc<br>aattcgtgctggTCATGTGAcacggcccccgc<br>taaatgcagttaTCATGTGAcacacaggcaac	5'UTR?<br>intron1<br>intron1<br>intron1	
Drosophila_erecta	Dere\GG12936		33	504	213	67	327	61	108			actttcgtgaaaTCATATGAtcgatttgcagt<br>ggatcttccaggTCATGTGAacttttcaccac<br>aattcgtgctggTCATGTGAcacggcccccgc<br>taagtgcagttaTCATGTGAcacaccggcaac	5'UTR?<br>intron1<br>intron1<br>intron1	
Drosophila_biarmipes	Dbia\Vha26		33	531	213	71	327	62	108			cttcccgtgaaaTCATATGAtcgatttgcagt<br>acgtcccccaggTCATGTGAactttctgtgcc<br>aattcgtgctggTCATGTGAcacggcccccgc<br>aaagtgcaattaTCATGTGAcgaaaggcgcaa	5'UTR?<br>intron1<br>intron1<br>intron1	
Drosophila_suzukii	Dsuz\Vha26		33	530	213	64	327	63	108			cctcccctgaaaTCATATGAtcgatttgcagt<br>atgttcaccaagTCATGTGAactttttatgcc<br>agttcgtgctggTCATGTGAcacggcccccgc<br>gaaatgcaactaTCATGTGAcgaaaggggcaa	5'UTR?<br>intron1<br>intron1<br>intron1	
Drosophila_takahashii	Dtak\Vha26		33	521	213	65	327	61	108			cttcgcgtgaaaTCATATGAtcgatttgcagt<br>atgttccccgagTCATGTGAactttccatccc<br>aattcgtgctggTCATGTGAcacggcccccgc<br>ccaatgcagttaTCATGTGAcgaaatgggcaa	5'UTR?<br>intron1<br>intron1<br>intron1	
Drosophila_eugracilis	Deug\Vha26		33	482	213	56	327	62	108			ctacccgtgaaaTCATATGAtcgatttgcagt<br>atgtcccccaagTCATGTGAactttttacatc<br>aattggtgctggTCATGTGAcacggcccccgc<br>ccaatgcagtaaTCATGTGAcaaaaggggtta	5'UTR?<br>intron1<br>intron1<br>intron1	
Drosophila_elegans	Dele\Vha26		33	517	213	66	327	62	108			cttcccgtgaaaTCATATGAtcgatttgcagt<br>gtcccccttcagTCATGTGAacttttcagcac<br>aattcgtgctggTCATGTGAcacggcccccgc<br>ccaatgcagttaTCATGTGAcaaaaaaaggca	5'UTR?<br>intron1<br>intron1<br>intron1	
Drosophila_rhopaloa	Drho\Vha26		33	516	213	74	327	62	108			cttttcgtgaaaTCATATGAtcgatttgcagt<br>tgttcccccaagTCATGTGAacttttcatccg<br>aattggtgctggTCATGTGAcacggccccgca<br>cgaatgcagttaTCATGTGAcgaaaaggacaa	5'UTR?<br>intron1<br>intron1<br>intron1	

Drosophila_ficusphila	Dfic\Vha26		33	496	213	63	327	64	108			cttcccgtaaaaTCATATGAtcgatttgcagt<br>ggtgcccccaagTCATGTGAacttttcaccac<br>aattcgtgatagTCATGTGAcacgggcccccg<br>ccaatgcagtaaTCATGTGAcaaaatgaggca	5'UTR?<br>intron1<br>intron1<br>intron1	
Drosophila_kikkawai	Dkik\Vha26		33	559	213	67	327	69	108			ctccccccaaaaTCATATGAtcgattgtgaaa<br>attgcccacaagTCATGTGAactctttgctac<br>aatacgtactggTCATGTGAcacggcccccgc<br>caaaggaaatagTCATGTGAcgctgtactaac	5'UTR?<br>intron1<br>intron1<br>intron1	
Drosophila_bipectinata	Dbip\Vha26		33	657	213	66	327	64	108			cttcacctgaaaTCATATGAtcgatttgcagt<br>cacaatagccagTCATGTGAatgttccccctc<br>atttcaatttggTCATGTGAcacggcccccgc<br>ctgaatgccctaTCATGTGAtggataaaggta	5'UTR?<br>intron1<br>intron1<br>intron1	
Drosophila_ananassae	Dana\GF20699		33	638	213	58	327	70	108			cttcacctgaaaTCATATGAtcgatttgcagt<br>cacaatagccagTCATGTGAacgcttcccccct<br>atttcattttggTCATGTGAcacggcccccgc<br>ctgaatacacaaTCATGTGAtggataatacga	5'UTR?<br>intron1<br>intron1<br>intron1	
Drosophila_pseudoobscura	Dpse\GA10614		33	590	213	67	327	64	108			cacactgaaaaaTCATATGAtcgatttgcagt<br>aatgattcgaagTCATGTGAatttctcatggc<br>aattcgtgttggTCATGTGAcatggactccac	5'UTR?<br>intron1<br>intron1	
Drosophila_persimilis	Dper\GL21540		33	591	213	67	327	64	108			cacactgaaaaaTCATATGAtcgatttgcagt<br>aatgattcgaagTCATGTGAatttctcatggc<br>aattcgtgttggTCATGTGAcatgggctccac	5'UTR?<br>intron1<br>intron1	
Drosophila_miranda	Dmir\Vha26		33	589	213	67	327	64	108			cacactaaaaaaTCATATGAtcgatttgctgt<br>aatgattcgaagTCATGTGAatttctcatggc<br>aattcgtgttggTCATGTGAcatgggctccac	5'UTR?<br>intron1<br>intron1	
Drosophila_willistoni	Dwil\GK22511		33	652	213	76	435 (327+108)					attgtgggctcaTCATATGAtttggccagtga<br>aaattgtcaaagTCATGTGAtaatcattagcg<br>attcgtattgggTCATGTGAcatgctccccca	5'UTR?<br>intron1<br>intron1	
Drosophila_albomicans	Dalb\Vha26		33	>725	213	351	327	70	108			acgaggtaaaaaTCATATGAtcgatttgtggt<br>gcgagtgaaaagTCATGTGAatattactcgca<br>aatacttattggTCATGTGAcacgcactacac	5'UTR?<br>intron1<br>intron1	
Drosophila_mojavensis	Dmoj\GI23674		33	704	213	57	327	63	108			acgaagtaaaaaTCATATGAtcgattagtcgt<br>ttctcgccgtagTCATGTGAattttgcgtaca<br>aaagcttattggTCATGTGAcagatattacat<br>agatataagtaaTCATGTGAttagctttgagg	5'UTR?<br>intron1<br>intron1<br>intron1	
Drosophila_virilis	Dvir\GJ23234		33	730	213	94	327	62	108			cacgaagtaaaaTCATATGAtcgattggaggt<br>ttctcatcgtagTCATGTGAattttgcgtgca<br>aatgcttattggTCATGTGAcacgcactacac<br>ataggtgcctaaTCATGTGAttagctttaggt	5'UTR?<br>intron1<br>intron1<br>intron1	
Drosophila_grimshawi	Dgri\GH14669		33	765	213	71	327	72	108			cacgaagtaaaaTCATATGAtcgatttgtggt<br>ttctctccgtagTCATGTGAattttagcatgc<br>attgcttattggTCATGTGAcacgcactagac<br>gagcaactaaaaTCATGTGAtttgcttaagag	5'UTR?<br>intron1<br>intron1<br>intron1	
