# Supplementary material for: Structural and Comparative Analyses of Insects Suggest the Presence of an Ultra-Conserved Regulatory Element of the Genes Encoding Vacuolar-Type ATPase Subunits and Assembly Factors
Source: Biology (Basel). 2023 Aug 13;12(8):1127. doi: 10.3390/biology12081127 (PMC10452791; doi:10.3390/biology12081127)
Supplement: Supplementary file 1 [file biology-12-01127-s001.zip › Supplementary Files S3-S19/Supp_File_S8_Atp6v1F_DROS.pdf]

Gene Cluster ID	Atp6v1F
**Drosophila melanogaster gene**	**Vha14-1 (CG8210)**
FlyBase ID	FBgn0262512
**Predicted function**	**ATPase, H+ transporting, lysosomal 14kDa, V1 subunit F**
Protein size	124 amino acids
Cytogenetic map	2R:52B5-52B2
Gene sequence location	2R:11566931..11567585[-]
**Dmel CLEAR sequence**	**TCATGTGA**
CLEAR sequence location	2R:11567581..11567588
Drosophila paralog	Vha14-2 (CG1076)

FlyBase Vha14-1 (CG8210) gene model & CLEAR element (red) positions:

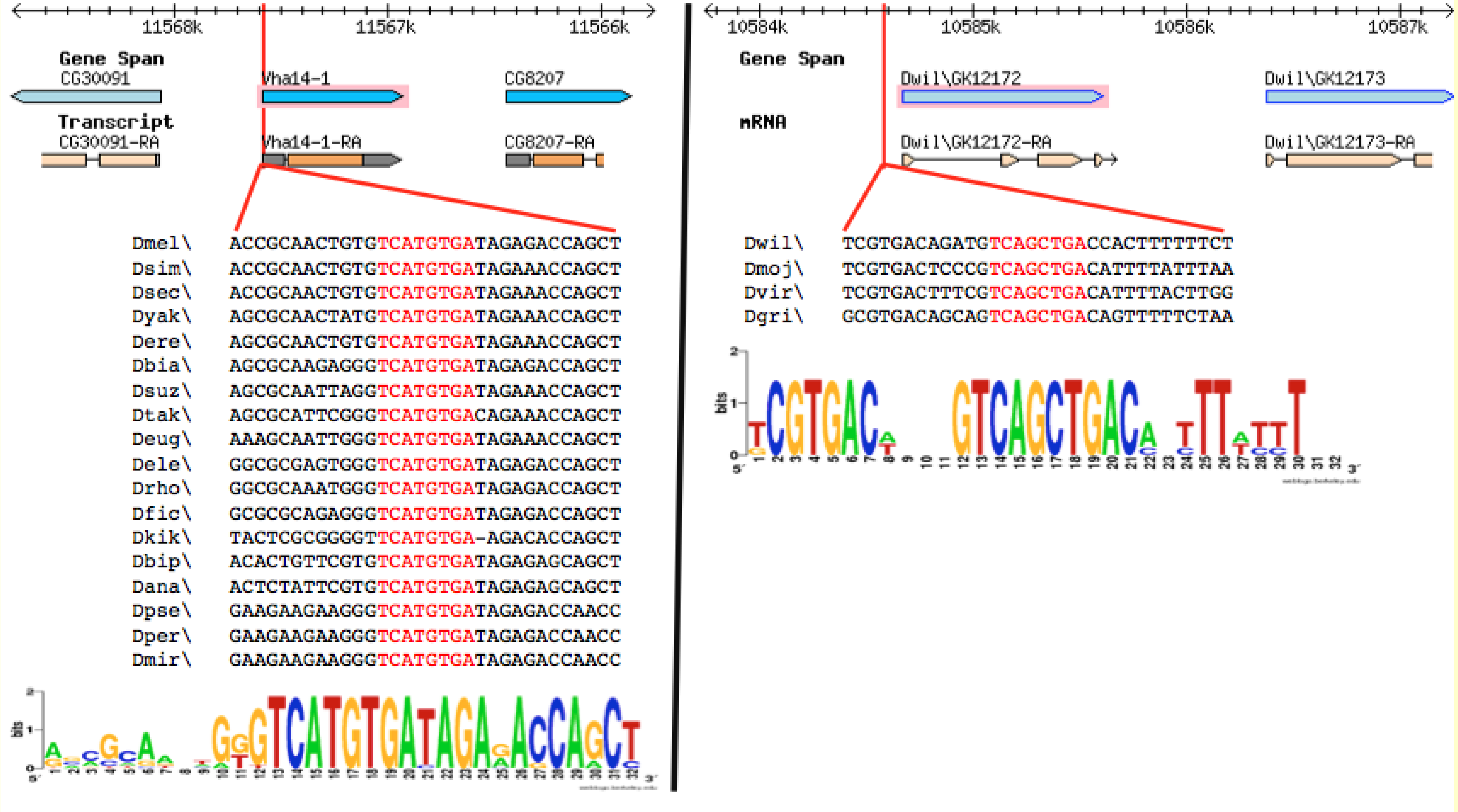

Atp6v1F gene structure comparison:

Key: UTR region, CDS region

Species	Atp6v1F orthologs	5'exon	intron	exon	intron	exon	intron	exon3'		Extended CLEAR region	Position	bps from TSS
Drosophila_melanogaster	Dmel\Vha14-1	98	375					182		accgcaactgtgTCATGTGAtagagaccagct	5'	-3
Drosophila_simulans	Dsim\GD25605	ND	375					ND		accgcaactgtgTCATGTGAtagaaaccagct	5'	

Drosophila_sechellia	Dsec\GM20126	ND	375							ND		accgcaactgtgTCATGTGAtagaaaccagct	5'	
Drosophila_yakuba	Dyak\GE14140	ND	375							ND		agcgcaactatgTCATGTGAtagaaaccagct	5'	
Drosophila_erecta	Dere\GG22339	ND	375							ND		agcgcaactgtgTCATGTGAtagaaaccagct	5'	
Drosophila_biarmipes	Dbia\Vha14-1	ND	375							ND		agcgcaagagggTCATGTGAtagagaccagct	5'	
Drosophila_suzukii	Dsuz\Vha14-1	ND	375							ND		agcgcaattaggTCATGTGAtagaaaccagct	5'	
Drosophila_takahashii	Dtak\Vha14-1	ND	375							ND		agcgcattcgggTCATGTGAcagaaaccagct	5'	
Drosophila_eugracilis	Deug\Vha14-1	ND	375							ND		aaagcaattgggTCATGTGAtagaaaccagct	5'	
Drosophila_elegans	Dele\Vha14-1	ND	375							ND		ggcgcgagtgggTCATGTGAtagagaccagct	5'	
Drosophila_rhopaloa	Drho\Vha14-1	ND	375							ND		ggcgcaaatgggTCATGTGAtagagaccagct	5'	
Drosophila_ficusphila	Dfic\Vha14-1	ND	375							ND		gcgcgcagagggTCATGTGAtagagaccagct	5'	
Drosophila_kikkawai	Dkik\Vha14-1	ND	375							ND		ctactcgcggggtTCATGTGAagacaccagct	5'	
Drosophila_bipectinata	Dbip\Vha14-1	ND	375							ND		acactgttcgtgTCATGTGAtagagagcagct	5'	
Drosophila_ananassae	Dana\GF13376	ND	375							ND		actctattcgtgTCATGTGAtagagagcagct	5'	
Drosophila_pseudoobscura	Dpse\Vha14-1	ND	375							ND		gaagaagaagggTCATGTGAtagagaccaacc	5'	
Drosophila_persimilis	Dper\GL11862	ND	375							ND		gaagaagaagggTCATGTGAtagagaccaacc	5'	
Drosophila_miranda	Dmir\Vha14-1	ND	375							ND		gaagaagaagggTCATGTGAtagagaccaacc	5'	
Drosophila_willistoni	Dwil\GK12172	ND	54	407	82	90	206	60	33	ND		tcgtgacagatgTCAGCTGAccacttttttct	5'	
Drosophila_albomicans	Dalb\Vha14-1	ND	ND	ND	82	320	206	57	33	ND		ND		
Drosophila_mojavensis	Dmoj\GI23969	ND	54	210	82	698	206	64	33	ND		tcgtgactcccgTCAGCTGAcattttatttaa	5'	
Drosophila_virilis	Dvir\GJ22513	ND	54	101	82	8390	206	57	33	ND		tcgtgactttcgTCAGCTGAcattttacttgg	5'	
	Dvir\GJ10919	ND	375							ND		tcgtgactttcgTCAGCTGAcattttgcttgg	5'	
Drosophila_grimshawi	Dgri\GH22329	ND	54	1903	82	385	206	67	33	ND		gcgtgacagcagTCAGCTGAcagtttttctaa	5'	
Paralog:Vha14-2 (CG1076)														
Drosophila_melanogaster	Dmel\Vha14-2(RB)	65	54	62	82	119	206	58	33	152				
Drosophila_simulans	Dsim\GD19837	ND	54	56	82	89	206	58	33	ND				
Drosophila_sechellia	Dsec\GM10855	ND	54	56	82	89	206	58	33	ND				

Drosophila_yakuba	Dyak\GE10180	ND	54	56	82	66	206	58	33	ND				
Drosophila_erecta	Dere\GG13113	ND	54	58	82	110	206	58	33	ND				
Drosophila_biarmipes	Dbia\Vha14-2	ND	54	59	82	181	206	59	33	ND				
Drosophila_suzukii	Dsuz\Vha14-2	ND	54	59	82	164	206	64	33	ND				
Drosophila_takahashii	Dtak\Vha14-2	ND	54	57	82	3977	206	67	33	ND				
Drosophila_eugracilis	Deug\Vha14-2	ND	54	66	82	51	206	59	33	ND				
Drosophila_elegans	Dele\Vha14-2	ND	54	55	82	199	206	55	33	ND				
Drosophila_rhopaloa	Drho\Vha14-2	ND	54	56	82	1049	206	55	33	ND				
Drosophila_ficusphila	Dfic\Vha14-2	ND	54	52	82	133	206	55	33	ND				
Drosophila_kikkawai	Dkik\Vha14-2	ND	75?	48	82	54	206	58	33	ND				
Drosophila_bipectinata	Dbip\Vha14-2	ND	54	51	82	59	206	60	33	ND				
Drosophila_ananassae	Dana\GF16369*	ND	54	53	82	59	206	58	33	ND				
Drosophila_pseudoobscura	Dpse\GA26491	ND	54	51	82	64	206	59	33	ND				
Drosophila_persimilis	Dper\GL24046	ND	ND	ND	ND	64	206	59	33	ND				
Drosophila_miranda	Dmir\Vha14-2	ND	54	51	82	64	206	59	33	ND				
Drosophila_willistoni	Dwil\GK13026*	ND	54	55	82	57	206	63	33	ND				
Drosophila_albomicans	Dalb\Vha14-2	ND	54	53	82	58	206	63	33	ND				
Drosophila_mojavensis	Dmoj\GI24384*	ND	54	62	82	85	206	59	33	ND				
Drosophila_virilis	Dvir\GJ14239*	ND	54	79	82	76	206	62	33	ND				
Drosophila_grimshawi	Dgri\GH14013*	ND	54	67	82	49	206	65	33	ND				

Key: UTR region, CDS region
