# Supplementary material for: Structural and Comparative Analyses of Insects Suggest the Presence of an Ultra-Conserved Regulatory Element of the Genes Encoding Vacuolar-Type ATPase Subunits and Assembly Factors
Source: Biology (Basel). 2023 Aug 13;12(8):1127. doi: 10.3390/biology12081127 (PMC10452791; doi:10.3390/biology12081127)
Supplement: Supplementary file 1 [file biology-12-01127-s001.zip › Supplementary Files S3-S19/Supp_File_S9_Atp6v1G_DROS.pdf]

Gene Cluster ID	Atp6v1G
**Drosophila melanogaster gene**	**Vha13 (CG6213)**
FlyBase ID	FBgn0026753
**Predicted function**	**ATPase, H+ transporting, lysosomal 13kDa, V1 subunit G**
Cytogenetic map	3R:92A11-92A11
Gene sequence location	3R:15468980..15470126[-]
**Dmel CLEAR sequence**	**TCATATGA; TCACATGA**
CLEAR sequence location(s)	3R:15470110..15470117; 3R:15469803..15469810

FlyBase Vha13 (CG6213) gene model & CLEAR element (red) positions:

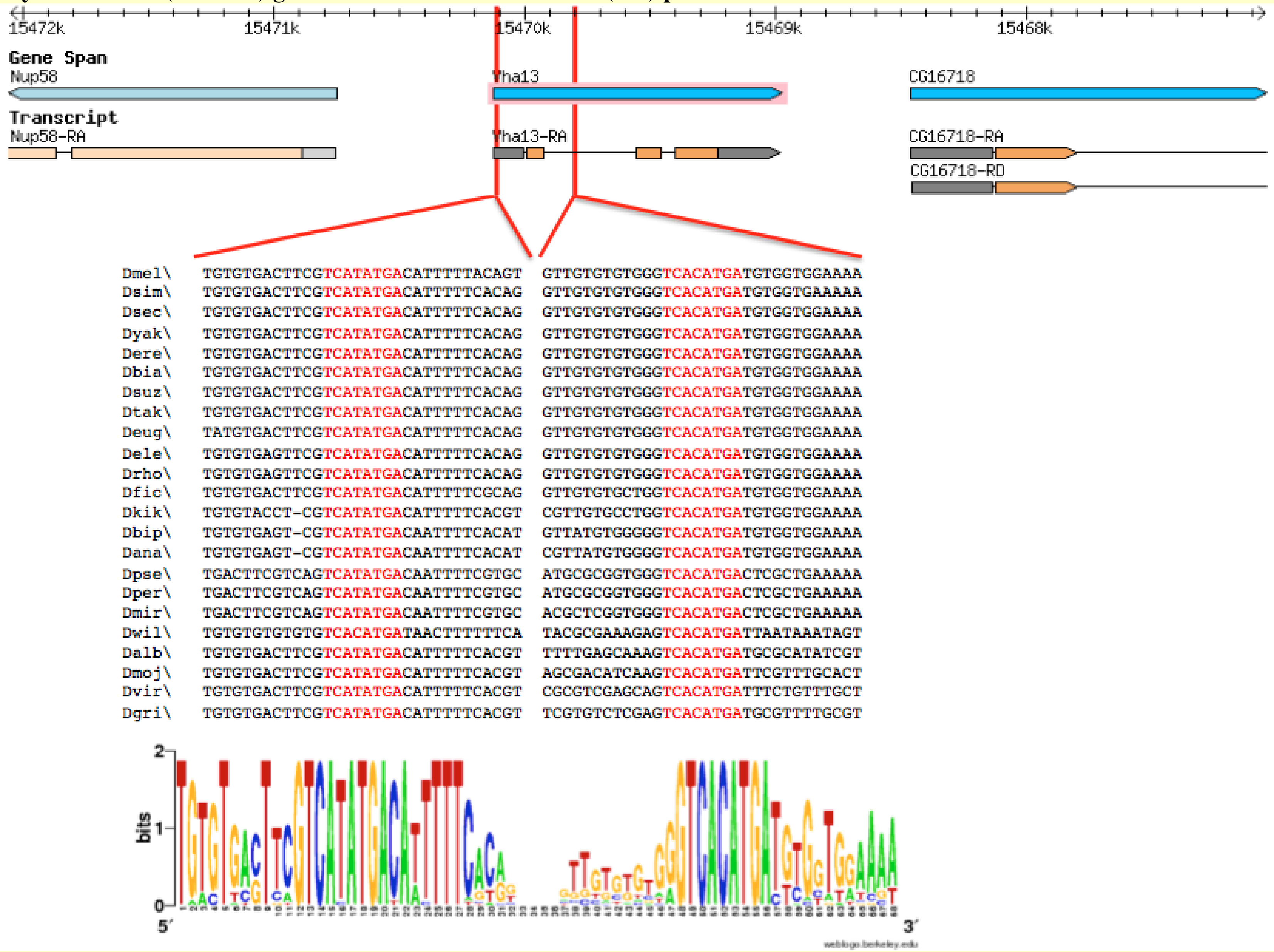

Atp6v1G (Vha13) gene structure comparison:

Key: UTR region, CDS region

Species	Vha13 Atp6v1G orthologs	5'exon		intron	exon	intron	exon3'			Extended CLEAR region	Position	bps from TSS
Drosophila_melanogaster	Dmel\Vha13	117	82	367	101	59	171	250		tgtgtgacttcgTCATATGAcatttttcacag<br>gttgtgtgtgggTCACATGAtgtggtggaaaa	5'UTR<br>intron1	9<br>317
Drosophila_simulans	Dsim\GD20107		82	350	101	59	171			tgtgtgacttcgTCATATGAcatttttcacag<br>gttgtgtgtgggTCACATGAtgtggtgaaaaa	5'UTR?<br>intron1	
Drosophila_sechellia	Dsec\GM26899		82	349	101	59	171			tgtgtgacttcgTCATATGAcatttttcacag<br>gttgtgtgtgggTCACATGAtgtggtggaaaa	5'UTR?<br>intron1	
Drosophila_yakuba	Dyak\Vha13		82	363	101	65	171			tgtgtgacttcgTCATATGAcatttttcacag<br>gttgtgtgtgggTCACATGAtgtggtggaaaa	5'UTR?<br>intron1	
Drosophila_erecta	Dere\GG15897		82	367	101	64	171			tgtgtgacttcgTCATATGAcatttttcacag<br>gttgtgtgtgggTCACATGAtgtggtggaaaa	5'UTR?<br>intron1	
Drosophila_biarmipes	Dbia\Vha13		82	362	101	65	171			tgtgtgacttcgTCATATGAcatttttcacag<br>gttgtgtgtgggTCACATGAtgtggtggaaaa	5'UTR?<br>intron1	
Drosophila_suzukii	Dsuz\Vha13		82	358	101	66	171			tgtgtgacttcgTCATATGAcatttttcacag<br>gttgtgtgtgggTCACATGAtgtggtggaaaa	5'UTR?<br>intron1	
Drosophila_takahashii	Dtak\Vha13		82	360	101	57	171			tgtgtgacttcgTCATATGAcatttttcacag<br>gttgtgtgtgggTCACATGAtgtggtggaaaa	5'UTR?<br>intron1	
Drosophila_eugracilis	Deug\Vha13		82	362	101	64	171			tatgtgacttcgTCATATGAcatttttcacag<br>gttgtgtgtgggTCACATGAtgtggtggaaaa	5'UTR?<br>intron1	
Drosophila_elegans	Dele\Vha13		82	191	101	71	171			tgtgtgagttcgTCATATGAcatttttcacag<br>gttgtgtgtgggTCACATGAtgtggtggaaaa	5'UTR?<br>intron1	
Drosophila_rhopaloa	Drho\Vha13		82	193	101	57	171			tgtgtgagttcgTCATATGAcatttttcacag<br>gttgtgtgtgggTCACATGAtgtggtggaaaa	5'UTR?<br>intron1	
Drosophila_ficusphila	Dfic\Vha13		82	178	101	73	171			tgtgtgacttcgTCATATGAcatttttcgcag<br>gttgtgtgctggTCACATGAtgtggtggaaaa	5'UTR?<br>intron1	
Drosophila_kikkawai	Dkik\Vha13		82	191	101	65	171			gtgtgtacctcgTCATATGAcatttttcacgt<br>cgttgtgcctggTCACATGAtgtggtggaaaa	5'UTR?<br>intron1	
Drosophila_bipectinata	Dbip\Vha13		82	190	101	256	171			ctgtgtgagtcgTCATATGAcaattttcacat<br>gttatgtggggggTCACATGAtgtggtggaaaa	5'UTR?<br>intron1	
Drosophila_ananassae	Dana\GF17364		82	189	101	180	171			ctgtgtgagtcgTCATATGAcaattttcacat<br>cgttatgtggggTCACATGAtgtggtggaaaa	5'UTR?<br>intron1	
Drosophila_pseudoobscura	Dpse\GA19444		82	219	101	68	171			tgacttcgtcagTCATATGAcaattttcgtgc<br>atgcgcggtgggTCACATGActcgctgaaaaa	5'UTR?<br>intron1	
Drosophila_persimilis	Dper\GL11956		82	219	101	67	171			tgacttcgtcagTCATATGAcaattttcgtgc<br>atgcgcggtgggTCACATGActcgctgaaaaa	5'UTR?<br>intron1	
Drosophila_miranda	Dmir\Vha13		82	222	101	68	171			tgacttcgtcagTCATATGAcaattttcgtgc<br>acgctcggtgggTCACATGActcgctgaaaaa	5'UTR?<br>intron1	
Drosophila_willistoni	Dwil\GK22514		82	224	101	65	171			tgtgtgtgtgtgTCACATGAtaactttttca<br>tacgcgaaagagTCACATGAttaataaatagt	5'UTR?<br>intron1	

Drosophila_albomicans	Dalb\Vha13		82	164	101	74	171			tgtgtgacttcgTCATATGAcatttttcacgt<br>ttttgagcaaagTCACATGAtgcgcatatcgt	5'UTR?<br>intron1	
Drosophila_mojavensis	Dmoj\GI24620		82	168	101	61	171			tgtgtgacttcgTCATATGAcatttttcacgt<br>agcgacatcaagTCACATGAttcgtttgcact	5'UTR?<br>intron1	
Drosophila_virilis	Dvir\GJ24533		82	152	101	68	171			tgtgtgacttcgTCATATGAcatttttcacgt<br>cgcgtcgagcagTCACATGAtttctgtttgct	5'UTR?<br>intron1	
Drosophila_grimshawi	Dgri\GH22384		82	222	101	75	174			tgtgtgacttcgTCATATGAcatttttcacgt<br>tcgtgtctcgagTCACATGAtgcgttttgcgt	5'UTR?<br>intron1	
